# Patent Deployment Strategies and Patent Value in LED Industry

**DOI:** 10.1371/journal.pone.0129911

**Published:** 2015-06-22

**Authors:** Ming-Fu Wu, Keng-Wei Chang, Wei Zhou, Juan Hao, Chien-Chung Yuan, Ke-Chiun Chang

**Affiliations:** 1 School of Economics and Management, Wuhan University, Wuhan 430072, China; 2 Department of Business Administration, National Yunlin University of Science and Technology, Yunlin 64002, Taiwan; 3 College of Public Administration and Law, Hunan Agricultural University, Changsha 410128, China; 4 Department of Computer Science and Information Engineering, Fu Jen Catholic University, New Taipei, 24205 Taiwan; East China University of Science and Technology, CHINA

## Abstract

This study applies two variables in the measurement of company patent deployment strategies: patent family depth and earn plan ratio. Patent family depth represents the degree to which certain fields and markets are valued by the patent owner. Earn plan ratio defined as the ratio of the number of patent forward citations to patent family size. Earn plan ratio indicates the degree to which a patent family could be cited by later innovators and competitors. This study applies a logistic regression model in the analysis LED industry data. The results demonstrate that patent value has a positive relationship with the patent family depth, and earn plan ratio.

## Introduction

A light-emitting diode (LED) is a semiconductor that converts electric energy to light energy. It is applied in a broad range of technologies, including substrates, epitaxy, chips, encapsulation, etc. As technology is core to LED companies’ competitiveness, patent applications are used by the industry as an effective mechanism to protect intellectual property. A patent is an intangible asset and can both exclude competitors in the same technological field and enhance company competitiveness and value. Patent litigation is also treated as an important strategic tool, and whether a lawsuit will be filed or not largely depends on patent value [1].

There are two options for patentees to deal with infringement: one is to settle out of court, another is to bring a lawsuit. The cost of litigation is typically much higher than the cost of reaching a settlement. Litigation in general involves not only high legal expense but also lengthy trials. Trials typically stretch companies’ human resources and capital, while posing a series of uncertainties about future outcomes. Therefore, patent litigation can have a negative effect on company operations. For these reasons, patent litigation is not considered if a patent does not offer substantial payoff. This is why patent litigation is a useful standard for measuring patent value [2].

Prior to litigation, patentee and non-patentee hold different expectations on the potential outcomes. A win for the patentee could mean continued profits and payment for damages stemming from the patent being upheld. If the patentee loses in court, the loss of exclusive rights to the patent technology could result in lower profitability and subsequent legal fees. In order to avoid such infringement litigation, patentees can authorize competitors to use a patented technology through license. Both patentees and non-patentees independently evaluate whether a lawsuit is worthwhile. Expectations of winning determine whether litigation takes place or not. When the expectation of winning on both sides tends to be very close, the sides are more likely to reach a settlement [3].

As profit margins are highly correlated to patent value, a patent with higher value can bring larger profits to a company. Patent value has a positive impact on the incentive to pursue patent litigation [4, 5]. Higher patent value means higher probability of infringement and litigation, as opposed to settlement. Patent litigation can, in turn, have an additional impact on patent value. If patent litigation is likely to fail, the prospect of losing can minimize patent value. Conversely, patent value increases if it results in winning and thus protecting and maintaining a company’s market share. Given the rapid rise in globalization of commercial activities, companies take advantage of patents’ legal value and implement legitimate commercial strategies to prevent potential contenders from entering the market. From a legal perspective, patent litigation becomes a measurement of patent value [6].

In prior literature, patent value has often been measured in terms of the number of patent claims and number of backward citations. Patents with more claims and backward citations generally have a higher value than those with fewer claims and backwards citations [2, 7]. However, patent value can also be reflected in company strategy as indicated by the patent family approach. Patent family refers to a cluster of applications for the same patent in different countries, including applications for its continuation, continuation-in-part and divisional applications. It indicates a company’s emphasis on certain fields and technologies. Patent citations show a patent’s appeal to follow-up innovators and competitors, which, to some extent, can reflect patent value. A patent often cited by others implicates that the technological knowledge involved in it is the foundation or core technology of the following patents, which has contributed to many later innovations [8]. Thus, patents with a high level of patent citations have greater appeal and greater market value. Based on this evidence, we derive patent family depth and the earn plan ratio to investigate a company’s market evaluation and the accuracy of its strategies. The structure of this paper is as follows: Section 2 would outline the literature review and hypothesis development; Section 3 described the methodology and measurement of this paper; Section 4 would discuss the empirical results; the final section was conclusions and implications of this study.

## Literature Review and Hypothesis Development

### Patent value determinants

Aiming at identifying the determinants of patent value and finding the most valuable patents, former researchers have developed various models, mainly categorizing independent variables into four different classes, including patent characteristics (number of forward citations, backward patent citations, International Patent Classifications (IPCs) and inventors etc.), patent ownerships (co-applicants, cross-border ownership, portfolio size, market size etc.), insider information (patenting motives and invention context) and filing strategies (number of priorities and claims etc.) [9]. From these variables, we can extract much valuable information about patents such as the technological importance [10], the existing technological background, the linkage between the innovation and basic research [11], the technological scope [12], the legal breadth of the protection [13] and so on.

As for the indicators of patent value, they can be divided into two categories: market-based indicators and patent-based indicators [9]. The most famous market-based indicators are Tobin’s q and stock market values at the firm level [14] and royalties, valuation by inventors and managers and buy-outs at the patent level. Patent-based indicators are much more diverse and can be clustered into five categories: technological importance (forward citations), geographical importance (family size), length (renewals), grant decisions (patent granted) and legal disputes (litigation incidences, opposition incidences). These five categories of indicators are found positively relevant to patent value [9].

Based on the different selections of patent value indicators and patent value determinants, various empirical studies have been conducted to predict the potential patent value. Forward citation counts, patent families, renewals, legal disputes and filing strategy indicators are consistently found positive related to patent value, nevertheless, the relationship of other determinants and patent value are ambiguous [9]. The phenomenon calls forth the need for further study into the determinants of patent value from a new perspective.

### The main effect of patent deployment strategy

To evaluate patent value, patent documents can provide some basic indicators such as patent claims, backward citations, and forward citations. Allison, Lemley [2] found that valuable patents have more patent claims, forward citations, and backward citations than others. In addition to characteristics as a measure, patent value is also reflected in companies’ strategic deployment. Patent family is the basic indicator of companies’ patent strategy [15]. Most define patent family as a set of either patent applications or publications taken in multiple countries to protect a single invention by a common inventor(s) and then patented in more than one country. By applying for protections of the same patent technology across several countries, companies are able to obtain sufficient protection and prevent their competitive advantages and market share from being eroded. Nevertheless, for the reason of the huge costs of apply and maintain a patent and the rigid Principle of Territory, only when a country has a huge market with big potential and wide prospect will companies apply for patents in that country. Thus whatever industry a patent belongs to, a company will not invest in a patent family unless it can surely receive a return on that investment. The more countries that have applied, the greater the value of the family [15–17]. Allison, Lemley [2] showed positively relationships between patent family and litigation possibility. Chang, Yang [18] suggested the deployment of patent family reveals potential markets; thus the size of patent family represents the importance of a technology and direction of future markets.

Patent citation shows the patent’s appeal to follow-on innovators and competitors. It measures the knowledge spillover effect and company market value. A large number of forward citations indicate that the patented technology is unique enough to influence innovators that follow and a high level of appeal to competitors. The more times a patent is cited by others, the higher the innovative value of the patent [19, 20]. Allison, Lemley [2], Marco [21] and Su, Chen [1] showed positive relation of forward citations and litigation possibility. Chang, Yang [18] suggested that patent forward citation is an important indicator to inspect in judging whether a technology is important and appealing to competitors.

According to the function of patent family and patent forward citations, this study derives two indicators to measure company strategic deployment. The first one is patent family depth, defined as the ratio of patent family size to the number of countries that a patent family is applied in. It shows the average patent family size of each patent family country, reflecting the degree to which certain fields and markets are valued by company. The greater the patent family depth, the more a company has valued the patent in question, which implies a high expected return from investing in the fields and markets included in the family. Hence, this study proposes the following hypothesis:

Hypothesis 1 (H_1_): The patent family depth is positively associated with the probability of patent litigation.

The second indicator is earn plan ratio, defined as the ratio of the number of patent forward citations to patent family size. It shows the degree to which a patent family could be cited by later innovators and competitors. A higher earn plan ratio implies increased confidence in company strategy, as it is an indication of rational capital allocation by investing in patents with greater competitiveness and value. Hence, this study proposes the following hypothesis:

Hypothesis 2 (H_2_): The earn plan ratio is positively associated with the probability of patent litigation.

## Methodology and Measurement

### Sample and data collection

The patent data used in this study were collected from the Thomson Innovation database. It includes US authorized patent information up to May 31, 2011. The technologies retrieved in this study include epitaxy, LED chip, and LED encapsulation technologies, excluding applications of end products. Primary searches obtained 40,330 patents, and 7,164 patents fell within the scope of this study after careful manual screening. To locate the precise technological fields of the LED industry, we interviewed 10 leading industry experts with at least 10 years of experiences in R&D. We used the Westlaw patent litigation database to verify litigated patents. Finally, we obtained a sample set of 7,164 total patents (64 litigated patents) (S1 Table).

### Measurement

Dependent variable: Litigated/Non-litigated patents: The dependent variable is a categorical variable and is coded as 1 if a patent has been litigated and 0 if a patent has never been litigated. This study used “litigated patent” as the proxy variable for a “valuable patent” and collected information from Westlaw to ensure whether each patent in the sample used to be litigated.

Independent variables: Patent family depth: the ratio of patent family size to the number of countries that the patent family has applied in. Family size of the patent can be referred to as the number of jurisdictions in which patent protection was sought for the same invention. The variable is a continuous variable, which is defined greater than or equal to 1. The study used patent database from International Patent Documentation Center (INPADOC) in European patent office website esp@cenet.

Earn Plan Ratio: the ratio of the number of patent forward citations to patent family size. The variable is a continuous variable, which is defined greater than or equal to 0. The study used Patent Full-Text and Image Database provided by USPTO and patent database from INPADOC in European patent office website esp@cenet.

Control variables: this study included a number of control variables in the empirical model that may influence probability of patent litigation: number of patent claims and number of backward citations.

Number of patent claims: Patent claim refers to an individual or set of claims proposed under certain conditions, describing the technological characteristics of a patent and stating the patent protection based on the form of patent application. It functions as criteria to clarify the protection range and judge infringement. For patentees, more patent claims mean a wider range of patent protections. However, maximizing patent protections means more than protection to patentees and motivation for invention and creation, but so acts as an obstacle to technology spread and application, which in turn leads to more infringement cases. Nerkar, Paruchuri [22] suggested that the more claims the greater the value of the intellectual property. In addition, Lanjouw and Schankerman [4] found that every 10% rise in the number of claims implies a 1.4% increase in sample litigation. Number of patent claims can be referred to as the sum of independent claims and dependent claims. The variable is a discrete variable, which is defined as an integer greater than or equal to 1. The study used Patent Full-Text and Image Database provided by United States Patent and Trademark Office (USPTO).

Number of backward citations: Backward citations are citations of existing and relevant patent/non-patent references reflecting the foundation of the patented technology [19, 20]. Lanjouw and Schankerman [4] found that each unit increase in the ratio of forward citations and claims is associated with a 22% rise in the probability of litigation. Allison, Lemley [2] showed that litigated patents contain more backward citations and are more likely to be cited by others compared to non-litigated patents. Generally, citation patterns reveal the diversity of technology sources. The more complicated a citation pattern, the more diversified the sources of a given technology. Therefore, patents which contain a great number of backward citations are those which are developed upon or improve existing technology, namely substantial technologies of great market value that entail a high possibility of litigation. Moreover, backward citations reveal the activeness of a company in certain technology fields. Narrow technology fields entail a high rate of infringements and litigations. This can also happen when competing companies recombine existing technologies and thus simulate an invention by studying the patented technology. Number of backward citations is measured by the sum of patent references and non-patent references, where the number of patent references consists of the number of US patent references and foreign patent references. The variable is a discrete variable, which is defined as an integer greater than or equal to 0. The study used Patent Full-Text and Image Database provided by USPTO.

## Results

### Basic patent analysis

Table 1 shows the top 10 companies by number of patents. Each company in this group has been granted more than 150 LED patents in the United States, with an average number near 200. The top company, Osram, owns 356 LED patents in the United States, followed by Philips and Cree, which have 256 and 250 patents respectively. Sony is ranked 10th with only 159 patents. Table 1 shows only minor differences among companies with the exception of the top company, Osram, and the last company, Sony. As the total number of patents reflects the degree of industry innovation, and the concentrative level of patent distribution reveals the severity of the competition, we can observe that there is both a high level of innovation and intense competition in the LED industry.

**Table 1:** Top 10 assignee.

Rank	Company	Number of Patents
1	Osram	356
2	Philips	256
3	Cree	250
4	Toshiba	222
5	Samsung	212
6	Toyoda Gosei	207
7	Matsushita	204
8	Nichia	199
9	Sharp	191
10	Sony	159

Table 2 shows the ranking of first assignee country for non-litigated patents and litigated patents. Japan, the United States, Taiwan, and Germany are included in the top five countries in terms of both non-litigated patents and litigated patents, which indicates that countries with more patents are also more likely to be countries of litigation. The ranking order changes slightly between non-litigated patents and litigated patents. Japan ranks at the top in non-litigated patents with a 41.7% share, but is second in terms of litigated patents with 17.2% share. At the same time, the United States ranks No. 2 in non-litigated patents, with a proportion of 33.3%, while it is No. 1 in litigated patents with 53.1%. The US is the largest market in LED industry and very concerned of the protection of intellectual property, so companies usually treat the US as the most important market and try to apply for patents there. When infringements occur, companies prefer to start litigations to protect their own rights and realize their strategic purpose. Hence, even though Japan has the most patents, US patents are more valuable, which brings about more litigation. The phenomenon is the same as the similar high-tech industry such as biomedicine or smartphone industries.

**Table 2:** Top 10 first assignee country.

Patent				Litigated patent			
Rank	Country	No.	%	Rank	Country	No.	%
1	Japan	2985	41.7%	1	USA	34	53.1%
2	USA	2389	33.3%	2	Japan	11	17.2%
3	Taiwan	582	8.1%	3	Germany	8	12.5%
4	Korea	432	6.0%	4	Taiwan	6	9.4%
5	Germany	342	4.8%	5	Canada	5	7.8%
6	Canada	126	1.8%				
7	Singapore	75	1.0%				
8	France	45	0.6%				
9	China	44	0.6%				
10	UK	31	0.4%				
		7051	98.30%			64	100%

Table 3 shows the top 10 International Patent Classification (IPC) subclass analysis for non-litigated patents and litigated patents. The first three IPC subclass levels in litigated patents are: (1) H01L, (2) C09K, and (3) H05K. Unlike the results shown in Table 2 where top-five countries appear in both cases of litigated patents and non-litigated patents, Table 3 shows that only 6 IPC subclass levels, H01L, C09K, C30B, G02B, F21V and H01J, cover both litigated and non-litigated patents. This suggests that litigation is more sensitive to IPC than country category. In addition, H01L ranks No. 1 in both litigated patents and non-litigated patents, with 65.6% and 56.25% respectively. This indicates the importance of H01L and implies intense competition in terms of that measure.

**Table 3:** Top 10 First IPC subclass analysis.

Patent				Litigated patent			
Rank	IPC code	No.	%	Rank	IPC code	No.	%
1	H01L	4702	65.6%	1	H01L	36	56.25%
2	C30B	465	6.5%	2	C09K	4	6.25%
3	F21V	282	3.9%	3	H05K	4	6.25%
4	H01J	218	3.0%	4	C30B	3	4.69%
5	H01S	165	2.3%	5	G02B	3	4.69%
6	C09K	164	2.3%	6	F21K	2	3.13%
7	G02B	150	2.1%	7	F21L	2	3.13%
8	H05B	135	1.9%	8	F21V	2	3.13%
9	C23C	116	1.6%	9	H01J	2	3.13%
10	F21S	51	0.7%	10	A61B	1	1.56%
		6448	89.90%			59	92.21%

Table 4 shows the top 10 IPC main-group analysis for both non-litigated patents and litigated patents. Table 4 further analyzes IPC. H01L0021 and H01L0023 take up the largest proportion in both non-litigated patents and litigated patents, with a percentage of 44.6% and 44.2% respectively. There are five IPC main groups that subordinated from H01L, ranked in the top five in non-litigated patents and in the top four in litigated patents, which is consistent with the result in Table 3. That means H01L0021 and H01L0023 are the center of in LED industry.

**Table 4:** Top 10 IPC main-group analysis.

Patent				Litigated patent			
Rank	IPC code	No.	%	Rank	IPC code	No.	%
1	H01L0021	1667	23.3%	1	H01L0033	17	26.56%
2	H01L0033	1526	21.3%	2	H01L0021	9	14.06%
3	H01L0029	599	8.4%	3	C09K0011	4	6.25%
4	H01L0023	325	4.5%	4	H01L0025	3	4.69%
5	H01L0027	297	4.1%	5	H01L0031	3	4.69%
6	C30B0025	201	2.8%	6	H05K0001	3	4.69%
7	C09K0011	162	2.3%	7	C30B0025	2	3.13%
8	H01S0005	152	2.1%	8	F21L0004	2	3.13%
9	H01J0001	146	2.0%	9	G02B0003	2	3.13%
10	H01L0031	136	1.9%	10	H01J0001	2	3.13%
		5211	72.70%			47	73.46%

### Descriptive statistics

The descriptive statistics and correlations matrix are shown in Table 5. Table 5 illustrates that the probability of patent litigation has significantly positive correlations with patent family depth, earn plan ratio, patent claims, and backward citations.

**Table 5:** Descriptive statistics and correlations matrix.

Variables	Min	Max	Mean	S. D.	1	2	3	4
1. Litigated/Non-Litigated patents	0	1	0.01	0.09	1			
2. Patent Family Depth	0.5	53	1.83	2.40	0.06**	1		
3. Earn Plan Ratio	0	241	3.80	11.25	0.03**	0.04**	1	
4. Patent Claims	1	228	16.87	13.85	0.05**	0.18**	-0.08**	1
5. Backward Citations	0	747	20.22	37.51	0.05**	0.31**	-0.06**	0.19**

**p<0.01.

*p<0.05.

### Results of logistics regression analysis

This study applied logistic regression analysis, setting dependent variable based on whether a patent is litigated and independent variables as the number of patent claims, number of backward citations, patent family depth and earn plan ratio. The results presented in Table 6 support the study hypothesis that all of the independent variables have positive influences on the dependent variable.

**Table 6:** Results of logistic regression analysis.

Variables	Patent Value
Intercept	-5.299**
Independent variables	
Patent Family Depth	0.066**
Earn Plan Ratio	0.014**
Control variables	
Patent Claims	0.014*
Backward Citations	0.004*
Log Likelihood	-353.3196
Prob > χ^2^	0.0001

**p<0.01.

*p<0.05.

Patent family depth and earn plan ratio have positive influences on the probability of patent litigation with a P value of 1%; number of patent claims and number of backward citations have positive influences on the probability of patent litigation with a P value of 5%. Patent family depth and earn plan ratio play more of a role than number of patent claims and number of backward citations in the probability of patent litigation.

## Conclusion and Discussion

Using samples from the LED industry, this study collected the US granted patent information through May 31, 2011, from the Thomson Innovation database. We found significant differences of number of patent claims, number of backward citations, patent family depth and earn plan ratio between non-litigated and litigated patents. This study applied logistic regression to test the relationship between the aforementioned four variables and the probability of patent litigation.

Based on the results showing a positive correlation between number of patent claims, number of backward citations and the probability of patent litigation, it is suggested that when applying for patents, companies should specialize patent claims, increase the number of patent claims and include as many backward citations as possible to increase patent value.

Patent family depth and earn plan ratio measure company strategies, and the results in this study show positive influences on the probability of patent litigation. Patent family depth represents the degree to which certain fields and markets are valued by a company. Greater patent family depth indicates the company expects higher return from investing in that market and the patent is essential and profitable so that the company is willing to afford huge maintenance fees to expand the patent family, which implies higher patent value. Therefore, it is suggested that companies apply for initial patents or continuation patents that have large market shares and high expected returns to prevent competitors from stepping into relevant technological field and crowding out the market and finally increase patent value.

Earn plan ratio indicates the degree to which a patent family could be cited by later innovators and competitors. A lower earn plan ratio indicates an excessive investment in patents of weaker competitiveness. In other words, patents with small earn plan ratios don’t have star technologies that following patents need to refer to, they have less attraction to external competitors and cannot spread technological knowledge to other patents, which implies the deployment strategy might be wrong and thus the patents have lower comparative values. Accordingly, it is suggested that when applying for patents, companies take patent deployment into consideration to estimate the value of the patent, including factors such as the technology development stage, the life cycle of relevant products, R&D activities of competitors and sales projections in target markets to judge whether the patent family is worth expanding and whether the patent can appeal competitors and improve patent value.

What’s more, in terms of the innovation of LED industry in which companies take technology advantages as their core competitive advantages, patent deployment strategies based on patent family depth and earn plan ratio can exert significant effects. First, these strategies encourage companies to build a more comprehensive patent family to prevent competitors from digging out a piece of technological field. Second, the strategies advise companies to evaluate the technology innovation from both the internal deployment and the external followers. It is preferred that companies maximize their technology status by winning more citations with smaller family size.

Finally, as to competitors, only by commercializing valuable patents can they promote the value of enterprise. So they could target the potential patent according to the patent value evaluated by the model, and negotiate with the patentee to obtain the use right by patent license. What’s more, competitors may as well analyze the patent deployment of two parties to seek potential opportunities of cooperation and realize development on complementary resources by cross licensing.

## Supporting Information

S1 Table The Raw Data of This Paper. (DOCX)
