# Supplementary material for: Patent Deployment Strategies and Patent Value in LED Industry
Source: PLoS One. 2015 Jun 22;10(6):e0129911. doi: 10.1371/journal.pone.0129911 (PMC4476752; doi:10.1371/journal.pone.0129911)
Supplement: S1 Table — (DOCX) [file pone.0129911.s001.docx]

**Supporting Information for:**

**Patent Deployment Strategies and Patent Value in LED Industry**

**S1 Table The Raw Data of This Paper**

| ID | litigated | First Assignee county | First IPC Subclass | First IPC Main-Group | Patent Claims Count | Patent Backward Citations | Patent Forward Citations | Patent Family Depth | Earn Plan Ratio |
| --- | --- | --- | --- | --- | --- | --- | --- | --- | --- |
| 1 | 0 | US | G09F | G09F0009 | 3 | 8 | 19 | 1 | 19.00 |
| 2 | 0 | US | H01L | H01L0033 | 6 | 3 | 61 | 1 | 61.00 |
| 3 | 0 | US | H01L | H01L0021 | 12 | 3 | 1 | 1 | 1.00 |
| 4 | 0 | US | H04N | H04N0009 | 20 | 7 | 126 | 1 | 126.00 |
| 5 | 0 | US | H01L | H01L0033 | 10 | 6 | 2 | 1 | 2.00 |
| 6 | 0 | US | C23C | C23C0018 | 32 | 3 | 8 | 1 | 8.00 |
| 7 | 0 | US | H01L | H01L0021 | 10 | 19 | 5 | 1 | 5.00 |
| 8 | 0 | US | G01N | G01N0021 | 20 | 18 | 8 | 1 | 8.00 |
| 9 | 0 | US | G02B | G02B0006 | 42 | 292 | 87 | 2.6 | 6.69 |
| 10 | 0 | JP | H01L | H01L0033 | 3 | 2 | 9 | 1 | 9.00 |
| 11 | 0 | US | H01L | H01L0033 | 6 | 2 | 23 | 1 | 23.00 |
| 12 | 1 | US | H01L | H01L0033 | 28 | 8 | 19 | 1 | 19.00 |
| 13 | 0 | US | C09K | C09K0011 | 9 | 6 | 6 | 1.25 | 1.20 |
| 14 | 0 | US | B81B | B81B0007 | 12 | 27 | 13 | 1 | 13.00 |
| 15 | 0 | US | H01L | H01L0033 | 4 | 2 | 82 | 1 | 82.00 |
| 16 | 0 | US | H01L | H01L0033 | 15 | 10 | 2 | 1 | 2.00 |
| 17 | 0 | US | H01L | H01L0033 | 16 | 25 | 0 | 1 | 0.00 |
| 18 | 0 | US | H01R | H01R0043 | 17 | 10 | 2 | 1.2 | 0.33 |
| 19 | 0 | US | H01L | H01L0029 | 8 | 292 | 3 | 2.6 | 0.23 |
| 20 | 0 | US | A61L | A61L0002 | 53 | 13 | 0 | 1.142857143 | 0.00 |
| 21 | 0 | US | F21K | F21K0002 | 10 | 8 | 14 | 1 | 14.00 |
| 22 | 0 | US | H01L | H01L0033 | 7 | 0 | 31 | 1 | 31.00 |
| 23 | 0 | US | H01L | H01L0033 | 4 | 11 | 80 | 1.333333333 | 10.00 |
| 24 | 0 | US | H01L | H01L0033 | 7 | 7 | 5 | 1.666666667 | 1.00 |
| 25 | 0 | US | G02F | G02F0001 | 24 | 1 | 12 | 1 | 3.00 |
| 26 | 0 | US | H01L | H01L0033 | 19 | 16 | 6 | 1.25 | 1.20 |
| 27 | 0 | US | C30B | C30B0025 | 12 | 16 | 7 | 2 | 1.17 |
| 28 | 0 | US | H01S | H01S0005 | 33 | 10 | 12 | 2.5 | 2.40 |
| 29 | 0 | US | H01S | H01S0005 | 12 | 8 | 0 | 1.25 | 0.00 |
| 30 | 0 | US | H01L | H01L0033 | 17 | 4 | 46 | 1 | 11.50 |
| 31 | 0 | US | H01L | H01L0033 | 15 | 2 | 48 | 1 | 16.00 |
| 32 | 0 | US | G09F | G09F0009 | 10 | 5 | 70 | 1 | 35.00 |
| 33 | 0 | US | H01L | H01L0033 | 14 | 26 | 15 | 1 | 3.00 |
| 34 | 0 | US | H01L | H01L0021 | 18 | 18 | 8 | 1.25 | 1.60 |
| 35 | 0 | US | C23C | C23C0016 | 17 | 29 | 32 | 1 | 16.00 |
| 36 | 0 | US | H05B | H05B0033 | 38 | 125 | 67 | 2.4 | 2.79 |
| 37 | 0 | US | H01L | H01L0033 | 8 | 5 | 128 | 1.5 | 42.67 |
| 38 | 0 | US | H01L | H01L0033 | 5 | 11 | 49 | 2 | 6.13 |
| 39 | 0 | US | H01L | H01L0033 | 10 | 3 | 8 | 1.333333333 | 2.00 |
| 40 | 0 | US | H01L | H01L0021 | 5 | 13 | 17 | 1 | 8.50 |
| 41 | 0 | US | F28D | F28D0015 | 12 | 14 | 14 | 2 | 1.40 |
| 42 | 0 | US | H01L | H01L0023 | 15 | 412 | 102 | 36.66666667 | 0.31 |
| 43 | 0 | US | H01L | H01L0021 | 7 | 10 | 7 | 1.5 | 0.78 |
| 44 | 0 | US | G02B | G02B0003 | 40 | 8 | 9 | 2 | 4.50 |
| 45 | 0 | US | C23C | C23C0016 | 18 | 14 | 10 | 3 | 3.33 |
| 46 | 0 | US | F28D | F28D0015 | 21 | 6 | 4 | 2 | 0.40 |
| 47 | 0 | US | H01L | H01L0029 | 23 | 11 | 45 | 1 | 7.50 |
| 48 | 0 | US | F21V | F21V0008 | 48 | 101 | 84 | 2.125 | 4.94 |
| 49 | 0 | US | G01N | G01N0021 | 21 | 31 | 2 | 1.363636364 | 0.13 |
| 50 | 0 | US | H01L | H01L0021 | 1 | 5 | 13 | 1 | 13.00 |
| 51 | 1 | US | H05K | H05K0001 | 29 | 22 | 59 | 2.5 | 11.80 |
| 52 | 0 | US | H01L | H01L0025 | 3 | 7 | 0 | 1.25 | 0.00 |
| 53 | 0 | US | H01L | H01L0021 | 69 | 59 | 24 | 1 | 12.00 |
| 54 | 0 | US | H01L | H01L0025 | 15 | 2 | 16 | 1.333333333 | 4.00 |
| 55 | 1 | US | H05K | H05K0001 | 63 | 13 | 49 | 5 | 9.80 |
| 56 | 0 | JP | C30B | C30B0025 | 13 | 9 | 28 | 1 | 28.00 |
| 57 | 0 | US | H01L | H01L0021 | 18 | 16 | 2 | 1.2 | 0.33 |
| 58 | 0 | US | G01N | G01N0021 | 68 | 58 | 11 | 1.333333333 | 1.38 |
| 59 | 0 | US | G01N | G01N0021 | 34 | 39 | 2 | 1.3 | 0.15 |
| 60 | 0 | US | H01L | H01L0033 | 8 | 4 | 7 | 1 | 3.50 |
| 61 | 0 | US | H01L | H01L0021 | 14 | 51 | 0 | 1.5 | 0.00 |
| 62 | 0 | US | C23F | C23F0001 | 100 | 9 | 9 | 1.666666667 | 1.80 |
| 63 | 0 | US | H01L | H01L0023 | 4 | 4 | 9 | 1 | 3.00 |
| 64 | 0 | US | H01L | H01L0021 | 20 | 16 | 4 | 1.2 | 0.67 |
| 65 | 0 | US | C23C | C23C0014 | 32 | 14 | 0 | 2 | 0.00 |
| 66 | 0 | US | B24B | B24B0037 | 9 | 37 | 0 | 17.86666667 | 0.00 |
| 67 | 1 | US | H05K | H05K0001 | 31 | 33 | 26 | 2.5 | 5.20 |
| 68 | 0 | US | G02B | G02B0006 | 44 | 4 | 11 | 20.63636364 | 0.05 |
| 69 | 0 | US | H01L | H01L0035 | 23 | 47 | 11 | 1 | 1.57 |
| 70 | 0 | US | H01L | H01L0023 | 3 | 10 | 25 | 2 | 6.25 |
| 71 | 0 | US | H01S | H01S0005 | 10 | 18 | 2 | 2 | 1.00 |
| 72 | 0 | US | F21K | F21K0099 | 13 | 7 | 20 | 1 | 20.00 |
| 73 | 0 | US | G02B | G02B0006 | 108 | 56 | 9 | 5.25 | 0.43 |
| 74 | 0 | US | B60Q | B60Q0001 | 21 | 8 | 14 | 2 | 7.00 |
| 75 | 0 | US | H01L | H01L0023 | 16 | 154 | 36 | 7.5 | 0.60 |
| 76 | 0 | US | B41J | B41J0002 | 10 | 5 | 0 | 1 | 0.00 |
| 77 | 0 | US | G02B | G02B0006 | 33 | 45 | 9 | 2.2 | 0.82 |
| 78 | 0 | US | H01L | H01L0021 | 15 | 5 | 34 | 2 | 17.00 |
| 79 | 0 | US | B60Q | B60Q0003 | 19 | 8 | 2 | 1 | 1.00 |
| 80 | 0 | US | F21V | F21V0008 | 16 | 5 | 0 | 1 | 0.00 |
| 81 | 0 | US | C09K | C09K0011 | 28 | 4 | 10 | 1 | 5.00 |
| 82 | 0 | US | H01L | H01L0023 | 17 | 14 | 6 | 1.25 | 1.20 |
| 83 | 0 | JP | H01L | H01L0033 | 7 | 21 | 14 | 1.8 | 1.56 |
| 84 | 0 | US | H01L | H01L0021 | 19 | 8 | 0 | 1 | 0.00 |
| 85 | 0 | US | H01L | H01L0021 | 17 | 40 | 4 | 3.285714286 | 0.17 |
| 86 | 0 | US | G02B | G02B0006 | 41 | 20 | 3 | 1 | 3.00 |
| 87 | 0 | US | F21V | F21V0021 | 29 | 106 | 13 | 2.5 | 2.60 |
| 88 | 0 | US | H05K | H05K0001 | 90 | 96 | 19 | 5 | 3.80 |
| 89 | 0 | US | H01L | H01L0029 | 18 | 57 | 5 | 5.25 | 0.24 |
| 90 | 0 | US | F21V | F21Y0101 | 20 | 101 | 14 | 5 | 2.80 |
| 91 | 0 | US | F21V | F21V0021 | 18 | 111 | 10 | 2.5 | 2.00 |
| 92 | 0 | JP | H01L | H01L0033 | 2 | 7 | 13 | 1 | 13.00 |
| 93 | 0 | US | H01L | H01L0021 | 35 | 16 | 2 | 1.5 | 0.67 |
| 94 | 0 | US | H01L | H01L0029 | 23 | 7 | 1 | 2.571428571 | 0.06 |
| 95 | 0 | US | H02B | H02B0001 | 19 | 5 | 0 | 1.285714286 | 0.00 |
| 96 | 0 | US | F21V | F21V0033 | 6 | 139 | 0 | 3.5 | 0.00 |
| 97 | 0 | US | H01L | H01L0033 | 23 | 19 | 0 | 2.571428571 | 0.00 |
| 98 | 0 | US | G02F | G02F0001 | 15 | 10 | 0 | 1.5 | 0.00 |
| 99 | 0 | JP | G02B | G02B0006 | 4 | 19 | 24 | 2.6 | 0.92 |
| 100 | 0 | US | H01L | H01L0033 | 20 | 12 | 0 | 2.571428571 | 0.00 |
| 101 | 0 | US | H01J | H01J0001 | 25 | 24 | 0 | 1 | 0.00 |
| 102 | 0 | CN | H01L | H01L0031 | 41 | 11 | 3 | 1.125 | 0.33 |
| 103 | 0 | JP | H01L | H01L0029 | 9 | 4 | 9 | 1 | 1.80 |
| 104 | 0 | CN | H01L | H01L0033 | 24 | 6 | 4 | 1.428571429 | 0.40 |
| 105 | 0 | JP | G02B | G02B0006 | 19 | 2 | 13 | 2.6 | 0.50 |
| 106 | 0 | CN | H01L | H01L0033 | 4 | 7 | 4 | 1.4 | 0.57 |
| 107 | 0 | CN | H01L | H01L0033 | 45 | 5 | 24 | 1.428571429 | 2.40 |
| 108 | 0 | US | H01L | H01L0021 | 5 | 21 | 16 | 1 | 16.00 |
| 109 | 0 | US | F21K | F21K0099 | 13 | 58 | 17 | 1 | 17.00 |
| 110 | 0 | US | H01L | H01L0029 | 11 | 925 | 2 | 1 | 0.67 |
| 111 | 0 | US | H01L | H01L0029 | 20 | 926 | 0 | 1.333333333 | 0.00 |
| 112 | 0 | US | H01L | H01L0021 | 22 | 17 | 0 | 2.142857143 | 0.00 |
| 113 | 0 | US | H01L | H01L0031 | 22 | 5 | 4 | 1.142857143 | 0.50 |
| 114 | 0 | US | H01S | H01S0005 | 44 | 7 | 8 | 2.25 | 0.89 |
| 115 | 0 | US | H01S | H01S0005 | 14 | 9 | 5 | 2.25 | 0.56 |
| 116 | 0 | US | B23K | B23K0035 | 32 | 43 | 2 | 1 | 0.67 |
| 117 | 0 | US | H01L | H01L0021 | 27 | 13 | 21 | 1 | 5.25 |
| 118 | 0 | JP | H01L | H01L0021 | 9 | 10 | 14 | 1 | 2.80 |
| 119 | 0 | US | H01L | H01L0021 | 15 | 24 | 4 | 1.142857143 | 0.50 |
| 120 | 0 | US | H01L | H01L0021 | 19 | 29 | 2 | 2 | 1.00 |
| 121 | 0 | US | F21V | F21V0008 | 40 | 2 | 80 | 1.666666667 | 16.00 |
| 122 | 0 | US | H01L | H01L0021 | 8 | 35 | 3 | 2 | 1.50 |
| 123 | 0 | US | H01L | H01L0033 | 16 | 28 | 192 | 1 | 192.00 |
| 124 | 0 | US | F21V | F21V0009 | 20 | 3 | 4 | 1.666666667 | 0.80 |
| 125 | 0 | US | F21V | F21V0005 | 14 | 4 | 6 | 1.666666667 | 1.20 |
| 126 | 0 | US | C07K | C07K0014 | 4 | 30 | 0 | 3 | 0.00 |
| 127 | 0 | US | H01L | H01L0023 | 9 | 12 | 0 | 1 | 0.00 |
| 128 | 1 | US | H01L | H01L0033 | 14 | 3 | 97 | 1 | 24.25 |
| 129 | 0 | US | H01L | H01L0021 | 16 | 15 | 0 | 1 | 0.00 |
| 130 | 0 | JP | C23C | C23C0016 | 26 | 12 | 6 | 1 | 6.00 |
| 131 | 0 | US | G02B | G02B0005 | 30 | 21 | 11 | 1 | 3.67 |
| 132 | 0 | JP | H01L | H01L0021 | 23 | 327 | 0 | 1 | 0.00 |
| 133 | 0 | JP | H01L | H01L0021 | 20 | 13 | 0 | 2 | 0.00 |
| 134 | 0 | US | H05B | H05B0033 | 6 | 15 | 0 | 1.2 | 0.00 |
| 135 | 0 | US | G02B | G02B0006 | 20 | 2 | 0 | 1 | 0.00 |
| 136 | 0 | US | H01L | H01L0023 | 37 | 14 | 16 | 3 | 5.33 |
| 137 | 0 | US | H01S | H01S0005 | 7 | 2 | 0 | 1 | 0.00 |
| 138 | 0 | TW | G09F | G09F0009 | 1 | 4 | 88 | 1 | 88.00 |
| 139 | 0 | US | C08L | C08L0053 | 17 | 27 | 44 | 1.375 | 4.00 |
| 140 | 0 | US | H01L | H01L0023 | 9 | 3 | 2 | 1 | 0.67 |
| 141 | 0 | US | H01L | H01L0023 | 6 | 38 | 8 | 1.5 | 1.33 |
| 142 | 0 | US | H01L | H01L0021 | 9 | 7 | 14 | 1 | 4.67 |
| 143 | 0 | US | H01S | H01S0005 | 22 | 19 | 0 | 1 | 0.00 |
| 144 | 0 | JP | H01L | H01L0033 | 10 | 11 | 77 | 3.333333333 | 7.70 |
| 145 | 0 | US | C30B | C30B0025 | 13 | 14 | 26 | 1 | 26.00 |
| 146 | 0 | US | H01L | H01L0021 | 22 | 50 | 3 | 1 | 3.00 |
| 147 | 0 | SG | H01L | H01L0021 | 8 | 13 | 8 | 1.5 | 2.67 |
| 148 | 0 | US | G02B | G02B0005 | 9 | 3 | 8 | 1 | 8.00 |
| 149 | 0 | JP | C30B | C30B0025 | 17 | 20 | 19 | 1.75 | 2.71 |
| 150 | 0 | SG | H01L | H01L0021 | 24 | 16 | 18 | 1 | 9.00 |
| 151 | 0 | SG | H01L | H01L0023 | 11 | 4 | 1 | 2 | 0.25 |
| 152 | 0 | SG | H01L | H01L0021 | 20 | 12 | 16 | 1 | 8.00 |
| 153 | 0 | SG | H01L | H01L0025 | 8 | 11 | 66 | 2 | 16.50 |
| 154 | 0 | SG | H01L | H01L0023 | 20 | 15 | 8 | 1.5 | 2.67 |
| 155 | 0 | US | H01L | H01L0021 | 59 | 7 | 7 | 1 | 7.00 |
| 156 | 0 | US | C30B | C30B0023 | 17 | 15 | 34 | 1 | 11.33 |
| 157 | 0 | US | H01L | H01L0021 | 20 | 11 | 2 | 2 | 1.00 |
| 158 | 0 | US | H01L | H01L0021 | 15 | 12 | 201 | 1 | 201.00 |
| 159 | 0 | US | G02B | G02B0006 | 52 | 27 | 64 | 1 | 64.00 |
| 160 | 0 | BE | H01L | H01L0021 | 15 | 8 | 3 | 1 | 3.00 |
| 161 | 0 | US | C23C | C23C0014 | 19 | 22 | 0 | 1 | 0.00 |
| 162 | 0 | US | C01B | C01B0021 | 5 | 34 | 7 | 1 | 7.00 |
| 163 | 0 | US | H01L | H01L0021 | 22 | 17 | 76 | 1 | 76.00 |
| 164 | 0 | US | H01L | H01L0021 | 12 | 11 | 8 | 1 | 8.00 |
| 165 | 0 | US | H01L | H01L0023 | 8 | 28 | 0 | 1 | 0.00 |
| 166 | 0 | US | F21S | F21S0010 | 24 | 9 | 8 | 1 | 8.00 |
| 167 | 0 | US | H01S | H01S0005 | 47 | 10 | 9 | 1 | 1.50 |
| 168 | 0 | TW | H01L | H01L0025 | 2 | 11 | 35 | 1 | 35.00 |
| 169 | 0 | US | C30B | C30B0025 | 19 | 17 | 4 | 1.8 | 0.44 |
| 170 | 0 | US | G02B | G02B0027 | 27 | 37 | 10 | 2 | 2.50 |
| 171 | 0 | KR | H01L | H01L0021 | 1 | 6 | 1 | 1 | 1.00 |
| 172 | 0 | US | H01L | H01L0023 | 38 | 36 | 0 | 1.333333333 | 0.00 |
| 173 | 0 | JP | H01L | H01L0023 | 8 | 3 | 8 | 1 | 8.00 |
| 174 | 0 | US | C01B | C01B0021 | 9 | 44 | 2 | 1 | 0.33 |
| 175 | 0 | US | H01L | H01L0021 | 25 | 193 | 0 | 17 | 0.00 |
| 176 | 0 | US | H01L | H01L0023 | 18 | 410 | 2 | 2 | 1.00 |
| 177 | 0 | TW | H01L | H01L0033 | 6 | 11 | 16 | 1 | 16.00 |
| 178 | 0 | US | G03F | G03F0001 | 20 | 5 | 5 | 1 | 5.00 |
| 179 | 0 | US | H01L | H01L0033 | 2 | 3 | 29 | 1 | 29.00 |
| 180 | 0 | US | H01L | H01L0033 | 47 | 27 | 140 | 1 | 140.00 |
| 181 | 0 | JP | H01L | H01L0029 | 21 | 16 | 4 | 1 | 0.80 |
| 182 | 0 | US | C04B | C04B0035 | 45 | 38 | 0 | 1 | 0.00 |
| 183 | 0 | TW | H01L | H01L0033 | 18 | 3 | 2 | 1 | 2.00 |
| 184 | 0 | US | H01L | H01L0021 | 64 | 5 | 1 | 1 | 1.00 |
| 185 | 0 | US | C30B | C30B0019 | 5 | 13 | 1 | 2.666666667 | 0.13 |
| 186 | 0 | JP | H01S | H01S0005 | 18 | 10 | 28 | 1 | 14.00 |
| 187 | 0 | US | C30B | C30B0019 | 20 | 11 | 4 | 2.666666667 | 0.50 |
| 188 | 0 | US | C08K | C08K0003 | 19 | 18 | 10 | 1 | 10.00 |
| 189 | 0 | US | C08G | C08G0059 | 25 | 21 | 7 | 1.166666667 | 0.50 |
| 190 | 0 | JP | H01L | H01L0021 | 8 | 12 | 6 | 1.333333333 | 1.50 |
| 191 | 0 | JP | H01L | H01L0033 | 4 | 12 | 7 | 1 | 1.75 |
| 192 | 1 | US | B64D | B64D0047 | 23 | 9 | 74 | 1 | 24.67 |
| 193 | 0 | US | H05B | H05B0033 | 52 | 8 | 7 | 1 | 7.00 |
| 194 | 0 | TW | H01L | H01L0033 | 18 | 5 | 23 | 2 | 11.50 |
| 195 | 0 | US | C30B | C30B0029 | 10 | 17 | 15 | 1 | 5.00 |
| 196 | 0 | US | H01L | H01L0021 | 17 | 17 | 2 | 2 | 1.00 |
| 197 | 0 | US | C30B | C30B0025 | 62 | 28 | 16 | 3 | 5.33 |
| 198 | 0 | US | G03F | G03F0007 | 33 | 217 | 26 | 4.666666667 | 1.86 |
| 199 | 0 | HK | C09K | C09K0011 | 4 | 6 | 2 | 1 | 2.00 |
| 200 | 0 | US | G03C | G03C0005 | 16 | 221 | 5 | 4.666666667 | 0.36 |
| 201 | 0 | US | C30B | C30B0025 | 37 | 33 | 1 | 1 | 0.20 |
| 202 | 0 | TW | H01L | H01L0033 | 4 | 3 | 17 | 1 | 17.00 |
| 203 | 0 | US | G03B | G03B0027 | 76 | 13 | 4 | 4.666666667 | 0.29 |
| 204 | 0 | US | C30B | C30B0025 | 46 | 34 | 2 | 3 | 0.67 |
| 205 | 0 | US | H01L | H01L0021 | 28 | 3 | 10 | 1 | 10.00 |
| 206 | 0 | JP | H01L | H01L0033 | 4 | 4 | 2 | 1.333333333 | 0.50 |
| 207 | 0 | US | H01L | H01L0029 | 18 | 12 | 0 | 1 | 0.00 |
| 208 | 0 | US | H01L | H01L0033 | 17 | 29 | 97 | 1 | 97.00 |
| 209 | 0 | JP | H01L | H01L0029 | 18 | 13 | 23 | 1.5 | 7.67 |
| 210 | 0 | US | B08B | B08B0007 | 18 | 30 | 6 | 3 | 0.29 |
| 211 | 0 | US | G02B | G02B0006 | 10 | 2 | 10 | 1 | 10.00 |
| 212 | 0 | TW | C30B | C30B0025 | 23 | 12 | 29 | 1 | 29.00 |
| 213 | 0 | US | H01L | H01L0029 | 15 | 12 | 0 | 1 | 0.00 |
| 214 | 0 | US | H01L | H01L0023 | 12 | 50 | 1 | 9 | 0.11 |
| 215 | 0 | US | H01L | H01L0027 | 20 | 8 | 0 | 1.166666667 | 0.00 |
| 216 | 0 | JP | H01L | H01L0033 | 6 | 5 | 6 | 2 | 1.50 |
| 217 | 0 | US | H01L | H01L0021 | 14 | 13 | 0 | 1 | 0.00 |
| 218 | 0 | US | C30B | C30B0025 | 12 | 33 | 14 | 12 | 1.17 |
| 219 | 0 | US | C09K | C09K0011 | 24 | 5 | 18 | 1 | 18.00 |
| 220 | 0 | US | C30B | C30B0025 | 37 | 75 | 7 | 12 | 0.58 |
| 221 | 0 | US | C09K | C09K0011 | 14 | 5 | 10 | 1 | 10.00 |
| 222 | 0 | TW | H01L | H01L0033 | 7 | 2 | 20 | 1 | 20.00 |
| 223 | 0 | US | C30B | C30B0025 | 36 | 71 | 2 | 12 | 0.17 |
| 224 | 0 | CH | C23C | C23C0016 | 22 | 13 | 12 | 1 | 12.00 |
| 225 | 0 | JP | C30B | C30B0025 | 8 | 10 | 2 | 1 | 0.40 |
| 226 | 0 | US | C30B | C30B0025 | 33 | 80 | 5 | 12 | 0.42 |
| 227 | 0 | DE | H01L | H01L0033 | 5 | 17 | 2 | 1 | 0.50 |
| 228 | 0 | JP | H01L | H01L0029 | 10 | 20 | 1 | 1 | 0.50 |
| 229 | 0 | US | C30B | C30B0009 | 14 | 3 | 14 | 2 | 7.00 |
| 230 | 0 | US | C30B | C30B0025 | 35 | 4 | 6 | 2 | 0.60 |
| 231 | 0 | US | C30B | C30B0025 | 41 | 81 | 4 | 12 | 0.33 |
| 232 | 0 | US | H01L | H01L0033 | 8 | 16 | 4 | 1 | 1.00 |
| 233 | 0 | US | C30B | C30B0025 | 30 | 4 | 5 | 2 | 0.50 |
| 234 | 0 | US | C30B | C30B0025 | 18 | 29 | 11 | 7.666666667 | 0.48 |
| 235 | 0 | TW | H01L | H01L0033 | 47 | 1 | 75 | 1 | 75.00 |
| 236 | 0 | US | C30B | C30B0025 | 47 | 10 | 5 | 2 | 0.50 |
| 237 | 0 | JP | H01L | H01L0033 | 8 | 5 | 0 | 1 | 0.00 |
| 238 | 0 | JP | H01L | H01L0033 | 5 | 5 | 10 | 1 | 2.50 |
| 239 | 0 | JP | H01L | H01L0027 | 4 | 15 | 3 | 1.333333333 | 0.75 |
| 240 | 0 | JP | H01L | H01L0033 | 6 | 6 | 6 | 2 | 1.50 |
| 241 | 0 | US | C30B | C30B0025 | 51 | 75 | 1 | 12 | 0.08 |
| 242 | 0 | US | C30B | C30B0025 | 50 | 80 | 0 | 7.666666667 | 0.00 |
| 243 | 0 | JP | H01L | H01L0021 | 4 | 14 | 49 | 1 | 49.00 |
| 244 | 0 | US | H01L | H01L0033 | 29 | 13 | 61 | 1 | 61.00 |
| 245 | 0 | US | B22F | B22F0001 | 18 | 60 | 7 | 1 | 7.00 |
| 246 | 0 | TW | H01L | H01L0033 | 8 | 3 | 24 | 1 | 24.00 |
| 247 | 0 | US | H01L | H01L0023 | 74 | 57 | 185 | 13.25 | 1.16 |
| 248 | 0 | US | H01L | H01L0033 | 18 | 3 | 19 | 1 | 19.00 |
| 249 | 0 | US | B60Q | B60Q0001 | 17 | 23 | 51 | 13.25 | 0.32 |
| 250 | 0 | US | F21S | F21S0008 | 36 | 23 | 12 | 1 | 12.00 |
| 251 | 0 | US | H01J | H01J0061 | 17 | 12 | 74 | 1 | 74.00 |
| 252 | 0 | US | H01L | H01L0021 | 13 | 35 | 2 | 13.25 | 0.01 |
| 253 | 0 | JP | G02B | G02B0006 | 32 | 10 | 9 | 1 | 4.50 |
| 254 | 0 | TW | H01L | H01L0033 | 3 | 3 | 4 | 1 | 4.00 |
| 255 | 0 | US | F21S | F21S0008 | 21 | 28 | 2 | 1 | 1.00 |
| 256 | 0 | US | H01L | H01L0029 | 156 | 61 | 2 | 13.25 | 0.01 |
| 257 | 0 | US | F21V | F21V0009 | 6 | 64 | 4 | 13.25 | 0.03 |
| 258 | 0 | AT | H01L | H01L0027 | 14 | 9 | 36 | 1 | 18.00 |
| 259 | 0 | TW | H01L | H01L0027 | 13 | 2 | 79 | 1 | 79.00 |
| 260 | 0 | TW | H01L | H01L0033 | 14 | 4 | 111 | 1 | 111.00 |
| 261 | 0 | US | C23F | C23F0004 | 20 | 4 | 0 | 1 | 0.00 |
| 262 | 0 | US | C23C | C23C0014 | 25 | 26 | 25 | 2.272727273 | 1.00 |
| 263 | 0 | US | H01L | H01L0021 | 15 | 18 | 0 | 2 | 0.00 |
| 264 | 0 | US | F21L | F21L0015 | 7 | 12 | 20 | 1 | 20.00 |
| 265 | 0 | US | H01L | H01L0021 | 18 | 6 | 102 | 1 | 34.00 |
| 266 | 0 | JP | H01L | H01L0021 | 4 | 62 | 5 | 1 | 5.00 |
| 267 | 0 | CH | C23C | C23C0016 | 13 | 26 | 20 | 1 | 20.00 |
| 268 | 0 | TW | H01K | H01K0023 | 19 | 10 | 38 | 1 | 38.00 |
| 269 | 0 | US | H01L | H01L0033 | 19 | 83 | 0 | 2 | 0.00 |
| 270 | 0 | US | C30B | C30B0029 | 70 | 61 | 31 | 1.625 | 2.38 |
| 271 | 0 | US | H01L | H01L0021 | 18 | 129 | 46 | 4 | 11.50 |
| 272 | 0 | JP | H01L | H01L0027 | 8 | 13 | 11 | 1.25 | 2.20 |
| 273 | 0 | US | H01L | H01L0021 | 51 | 41 | 24 | 1.5 | 2.67 |
| 274 | 0 | JP | H04N | H04N0001 | 49 | 29 | 14 | 3.2 | 0.88 |
| 275 | 0 | US | C23C | C23C0014 | 93 | 79 | 41 | 2.2 | 3.73 |
| 276 | 0 | TW | H01L | H01L0033 | 20 | 6 | 72 | 1 | 72.00 |
| 277 | 0 | US | H01L | H01L0031 | 72 | 135 | 2 | 1.75 | 0.29 |
| 278 | 0 | US | H01L | H01L0021 | 47 | 69 | 23 | 1.5 | 2.56 |
| 279 | 0 | US | H01L | H01L0031 | 10 | 106 | 1 | 2.333333333 | 0.14 |
| 280 | 0 | US | H01L | H01L0029 | 12 | 133 | 0 | 1.333333333 | 0.00 |
| 281 | 0 | US | H01L | H01L0021 | 26 | 117 | 0 | 2.333333333 | 0.00 |
| 282 | 0 | US | G02B | G02B0006 | 17 | 65 | 1 | 10.77777778 | 0.01 |
| 283 | 0 | JP | H01L | H01L0033 | 1 | 4 | 17 | 1.5 | 5.67 |
| 284 | 0 | TW | H01L | H01L0033 | 20 | 11 | 17 | 1 | 17.00 |
| 285 | 0 | TW | H01L | H01L0033 | 19 | 11 | 5 | 1 | 5.00 |
| 286 | 0 | US | G02B | G02B0006 | 10 | 66 | 3 | 10.77777778 | 0.03 |
| 287 | 0 | JP | G02B | G02B0006 | 9 | 7 | 5 | 1 | 5.00 |
| 288 | 0 | KR | H01L | H01L0021 | 8 | 13 | 2 | 1 | 1.00 |
| 289 | 0 | US | C30B | C30B0025 | 20 | 49 | 4 | 1.333333333 | 1.00 |
| 290 | 0 | TW | H01L | H01L0025 | 30 | 3 | 13 | 1 | 13.00 |
| 291 | 0 | US | H01L | H01L0021 | 1 | 2 | 25 | 1 | 25.00 |
| 292 | 0 | TW | H01L | H01L0033 | 4 | 5 | 56 | 1 | 56.00 |
| 293 | 0 | TW | H01L | H01L0033 | 19 | 4 | 4 | 1 | 4.00 |
| 294 | 0 | DE | H01L | H01L0021 | 15 | 8 | 14 | 1.142857143 | 1.75 |
| 295 | 0 | US | C30B | C30B0023 | 7 | 5 | 1 | 1 | 1.00 |
| 296 | 0 | US | H01L | H01L0021 | 13 | 4 | 3 | 1 | 3.00 |
| 297 | 0 | US | H01L | H01L0029 | 28 | 22 | 7 | 1 | 7.00 |
| 298 | 0 | US | G02B | G02B0005 | 35 | 173 | 14 | 1.2 | 2.33 |
| 299 | 0 | TW | H01L | H01L0025 | 13 | 1 | 4 | 1 | 4.00 |
| 300 | 0 | TW | H01L | H01L0021 | 15 | 4 | 0 | 1 | 0.00 |
| 301 | 0 | US | H01L | H01L0033 | 17 | 8 | 9 | 1 | 3.00 |
| 302 | 0 | TW | H01L | H01L0033 | 10 | 10 | 27 | 2 | 13.50 |
| 303 | 0 | TW | H01L | H01L0023 | 5 | 4 | 11 | 1 | 11.00 |
| 304 | 0 | TW | H01L | H01L0023 | 6 | 13 | 4 | 1 | 4.00 |
| 305 | 0 | US | H01J | H01J0001 | 25 | 56 | 4 | 1 | 2.00 |
| 306 | 0 | JP | H01L | H01L0027 | 14 | 13 | 2 | 1.25 | 0.40 |
| 307 | 0 | US | H01S | H01S0005 | 5 | 33 | 6 | 5.75 | 0.13 |
| 308 | 0 | US | H01J | H01J0001 | 8 | 38 | 1 | 2.333333333 | 0.07 |
| 309 | 0 | US | H01L | H01L0029 | 31 | 39 | 2 | 1.444444444 | 0.15 |
| 310 | 0 | US | G01J | G01J0005 | 12 | 7 | 2 | 1.333333333 | 0.50 |
| 311 | 1 | TW | H01L | H01L0033 | 13 | 6 | 59 | 1 | 59.00 |
| 312 | 0 | TW | H01L | H01L0033 | 10 | 3 | 0 | 1 | 0.00 |
| 313 | 0 | US | H01L | H01L0021 | 20 | 32 | 8 | 1.25 | 1.60 |
| 314 | 0 | KR | C30B | C30B0025 | 6 | 8 | 4 | 1 | 4.00 |
| 315 | 0 | JP | H01L | H01L0021 | 33 | 6 | 35 | 2.5 | 7.00 |
| 316 | 0 | JP | G02F | G02F0001 | 14 | 10 | 34 | 1 | 17.00 |
| 317 | 0 | TW | F21V | F21V0008 | 9 | 4 | 30 | 1 | 30.00 |
| 318 | 0 | TW | H01L | H01L0033 | 4 | 4 | 3 | 1 | 3.00 |
| 319 | 0 | TW | H01L | H01L0021 | 20 | 9 | 14 | 1 | 14.00 |
| 320 | 0 | KR | H01L | H01L0033 | 3 | 12 | 21 | 1 | 21.00 |
| 321 | 0 | US | C30B | C30B0025 | 20 | 3 | 0 | 1.25 | 0.00 |
| 322 | 1 | US | H01L | H01L0033 | 32 | 28 | 56 | 1 | 11.20 |
| 323 | 0 | US | C30B | C30B0015 | 29 | 21 | 4 | 1.6 | 0.50 |
| 324 | 0 | US | C30B | C30B0025 | 39 | 18 | 7 | 1 | 7.00 |
| 325 | 0 | US | H01L | H01L0051 | 34 | 13 | 36 | 1 | 36.00 |
| 326 | 0 | JP | G02F | G02F0001 | 6 | 11 | 11 | 1 | 11.00 |
| 327 | 0 | US | H01L | H01L0033 | 31 | 14 | 6 | 2 | 3.00 |
| 328 | 0 | JP | H01L | H01L0033 | 19 | 8 | 56 | 1.166666667 | 8.00 |
| 329 | 0 | JP | C30B | C30B0025 | 27 | 26 | 37 | 3 | 6.17 |
| 330 | 0 | JP | H01L | H01L0029 | 16 | 4 | 8 | 1 | 8.00 |
| 331 | 0 | US | F21V | F21V0019 | 9 | 17 | 18 | 1.333333333 | 4.50 |
| 332 | 0 | US | C30B | C30B0015 | 15 | 3 | 2 | 1.6 | 0.25 |
| 333 | 0 | JP | H01L | H01L0027 | 9 | 1 | 10 | 1 | 10.00 |
| 334 | 0 | US | H01L | H01L0033 | 66 | 3 | 36 | 1 | 36.00 |
| 335 | 0 | US | C30B | C30B0029 | 10 | 7 | 0 | 3 | 0.00 |
| 336 | 0 | JP | C30B | C30B0025 | 11 | 10 | 57 | 1 | 28.50 |
| 337 | 0 | US | C23C | C23C0016 | 42 | 3 | 0 | 1.6 | 0.00 |
| 338 | 0 | US | C30B | C30B0025 | 39 | 9 | 3 | 1 | 0.60 |
| 339 | 0 | US | H01L | H01L0033 | 37 | 22 | 10 | 1 | 3.33 |
| 340 | 0 | US | H01J | H01J0009 | 12 | 39 | 3 | 2.333333333 | 0.21 |
| 341 | 0 | US | H01L | H01L0021 | 12 | 8 | 0 | 3 | 0.00 |
| 342 | 0 | US | H05B | H05B0033 | 15 | 25 | 9 | 1.333333333 | 2.25 |
| 343 | 0 | US | H05B | H05B0033 | 17 | 38 | 0 | 2 | 0.00 |
| 344 | 0 | US | H05B | H05B0033 | 18 | 29 | 8 | 1.5 | 2.67 |
| 345 | 0 | US | H05B | H05B0033 | 42 | 25 | 0 | 1 | 0.00 |
| 346 | 0 | JP | H01C | H01G0004 | 9 | 13 | 4 | 1 | 2.00 |
| 347 | 0 | TW | H01L | H01L0021 | 19 | 4 | 11 | 1 | 11.00 |
| 348 | 0 | FR | H01L | H01L0033 | 28 | 15 | 8 | 1 | 8.00 |
| 349 | 0 | DE | H01L | H01L0033 | 25 | 17 | 4 | 1 | 0.67 |
| 350 | 0 | US | H01L | H01L0051 | 47 | 12 | 2 | 1.5 | 0.67 |
| 351 | 0 | TW | H01L | H01L0033 | 10 | 19 | 1 | 1 | 1.00 |
| 352 | 0 | US | H05B | H05B0003 | 20 | 19 | 0 | 1 | 0.00 |
| 353 | 0 | TW | H01L | H01L0033 | 30 | 4 | 8 | 1 | 8.00 |
| 354 | 0 | US | H01L | H01L0051 | 12 | 64 | 0 | 1.666666667 | 0.00 |
| 355 | 0 | US | C09K | C09K0011 | 21 | 61 | 0 | 1.285714286 | 0.00 |
| 356 | 0 | CA | C30B | C30B0029 | 26 | 13 | 0 | 1 | 0.00 |
| 357 | 0 | JP | H01L | H01L0033 | 9 | 7 | 12 | 1.5 | 2.00 |
| 358 | 0 | TW | H01L | H01L0033 | 6 | 5 | 114 | 1 | 114.00 |
| 359 | 0 | US | G03F | G03F0001 | 51 | 45 | 4 | 2.285714286 | 0.25 |
| 360 | 0 | TW | H01L | H01L0021 | 3 | 4 | 4 | 1 | 4.00 |
| 361 | 0 | US | H01L | H01L0021 | 19 | 73 | 0 | 2 | 0.00 |
| 362 | 0 | US | C09K | C09K0011 | 20 | 5 | 1 | 1 | 0.25 |
| 363 | 0 | US | C23C | C23C0016 | 35 | 67 | 18 | 6.75 | 0.33 |
| 364 | 0 | US | C23C | C23C0016 | 7 | 254 | 0 | 2.833333333 | 0.00 |
| 365 | 0 | JP | H01L | H01L0033 | 20 | 11 | 48 | 1.2 | 8.00 |
| 366 | 0 | US | H01L | H01L0029 | 21 | 31 | 18 | 1 | 18.00 |
| 367 | 0 | DE | H01L | H01L0033 | 13 | 24 | 15 | 1 | 2.14 |
| 368 | 0 | US | C30B | C30B0025 | 16 | 15 | 10 | 1 | 10.00 |
| 369 | 0 | TW | H01L | H01L0021 | 17 | 7 | 0 | 1 | 0.00 |
| 370 | 0 | TW | H01L | H01L0033 | 7 | 3 | 92 | 1 | 92.00 |
| 371 | 0 | US | H01S | H01S0005 | 60 | 11 | 0 | 1 | 0.00 |
| 372 | 0 | TW | H01L | H01L0033 | 9 | 2 | 6 | 1 | 6.00 |
| 373 | 0 | CA | F21V | F21V0008 | 22 | 8 | 2 | 1 | 2.00 |
| 374 | 0 | JP | H01L | H01L0021 | 48 | 8 | 10 | 1 | 3.33 |
| 375 | 0 | JP | C30B | C30B0025 | 3 | 27 | 13 | 3 | 2.17 |
| 376 | 0 | US | H01L | H01L0021 | 6 | 2 | 0 | 1 | 0.00 |
| 377 | 0 | TW | H01L | H01L0027 | 19 | 5 | 0 | 1 | 0.00 |
| 378 | 0 | US | F21S | F21S0008 | 3 | 9 | 6 | 1 | 1.50 |
| 379 | 0 | US | G09F | G09F0009 | 13 | 17 | 2 | 1 | 2.00 |
| 380 | 0 | US | G02B | G02B0001 | 14 | 120 | 0 | 1 | 0.00 |
| 381 | 0 | US | H01L | H01L0033 | 38 | 186 | 0 | 1 | 0.00 |
| 382 | 0 | TW | H01L | H01L0033 | 8 | 3 | 3 | 1 | 3.00 |
| 383 | 0 | US | G02F | G02F0002 | 15 | 181 | 0 | 1.285714286 | 0.00 |
| 384 | 0 | TW | H01L | H01L0021 | 10 | 4 | 18 | 1 | 18.00 |
| 385 | 0 | TW | F21V | F21V0029 | 9 | 8 | 3 | 1 | 3.00 |
| 386 | 0 | US | H01L | H01L0051 | 4 | 18 | 7 | 4.083333333 | 0.14 |
| 387 | 0 | TW | H01L | H01L0025 | 12 | 4 | 37 | 1 | 18.50 |
| 388 | 0 | US | H01L | H01L0051 | 7 | 20 | 6 | 4.083333333 | 0.12 |
| 389 | 0 | TW | H01L | H01L0033 | 16 | 3 | 39 | 1 | 39.00 |
| 390 | 0 | US | G03F | G03F0007 | 11 | 62 | 28 | 2.090909091 | 1.22 |
| 391 | 0 | TW | C30B | C30B0011 | 20 | 9 | 11 | 1 | 11.00 |
| 392 | 0 | JP | H01S | H01S0005 | 9 | 16 | 1 | 1 | 0.25 |
| 393 | 0 | US | H01L | H01L0025 | 30 | 5 | 3 | 2.333333333 | 0.43 |
| 394 | 0 | US | H01L | H01L0033 | 24 | 6 | 50 | 3 | 16.67 |
| 395 | 0 | US | H01L | H01L0033 | 21 | 28 | 24 | 1.625 | 1.85 |
| 396 | 0 | US | C30B | C30B0015 | 17 | 32 | 1 | 1.5 | 0.33 |
| 397 | 0 | US | H01L | H01L0033 | 19 | 37 | 0 | 1 | 0.00 |
| 398 | 0 | TW | C09K | C09K0011 | 10 | 21 | 6 | 1 | 2.00 |
| 399 | 0 | TW | H01L | H01L0033 | 4 | 15 | 41 | 1 | 41.00 |
| 400 | 0 | US | H01L | H01L0033 | 53 | 29 | 19 | 1.166666667 | 2.71 |
| 401 | 0 | JP | H01L | H01L0021 | 22 | 18 | 3 | 1 | 1.50 |
| 402 | 0 | US | H01L | H01L0029 | 31 | 10 | 12 | 1.166666667 | 1.71 |
| 403 | 0 | US | H05K | H05K0003 | 10 | 26 | 12 | 1.2 | 2.00 |
| 404 | 0 | US | B60Q | B60Q0001 | 19 | 12 | 56 | 1.333333333 | 14.00 |
| 405 | 0 | CA | C09K | C09K0011 | 35 | 21 | 11 | 1 | 1.83 |
| 406 | 0 | US | H01L | H01L0033 | 14 | 19 | 20 | 1.166666667 | 2.86 |
| 407 | 0 | US | H01L | H01L0033 | 11 | 21 | 3 | 1.166666667 | 0.43 |
| 408 | 0 | US | H01L | H01L0021 | 10 | 10 | 10 | 2 | 5.00 |
| 409 | 0 | US | H01L | H01L0033 | 38 | 33 | 101 | 1 | 16.83 |
| 410 | 0 | CA | H01L | H01L0029 | 54 | 12 | 28 | 1 | 28.00 |
| 411 | 0 | KR | H01L | H01L0025 | 6 | 15 | 78 | 1 | 19.50 |
| 412 | 0 | US | C08J | C08J0003 | 69 | 320 | 4 | 5.357142857 | 0.05 |
| 413 | 0 | US | C12Q | C12Q0001 | 8 | 8 | 8 | 1.333333333 | 1.00 |
| 414 | 0 | JP | H01L | H01L0051 | 9 | 13 | 3 | 1 | 1.50 |
| 415 | 0 | US | H01L | H01L0021 | 29 | 21 | 1 | 1 | 1.00 |
| 416 | 0 | US | H01L | H01L0021 | 8 | 4 | 19 | 1 | 19.00 |
| 417 | 0 | TW | H01L | H01L0033 | 6 | 2 | 3 | 1 | 3.00 |
| 418 | 0 | JP | H01L | H01L0021 | 4 | 17 | 5 | 1.5 | 1.67 |
| 419 | 0 | JP | H01L | H01L0033 | 27 | 7 | 10 | 1.666666667 | 2.00 |
| 420 | 0 | US | H01J | H01J0001 | 9 | 4 | 23 | 1 | 23.00 |
| 421 | 0 | US | G01D | G01D0015 | 5 | 5 | 8 | 1 | 8.00 |
| 422 | 0 | US | C30B | C30B0029 | 9 | 2 | 27 | 1 | 27.00 |
| 423 | 0 | TW | H01L | H01L0021 | 7 | 2 | 2 | 1 | 2.00 |
| 424 | 0 | JP | H01L | H01L0021 | 25 | 7 | 1 | 2.5 | 0.20 |
| 425 | 0 | US | H01L | H01L0033 | 4 | 7 | 40 | 1 | 40.00 |
| 426 | 0 | US | H01L | H01L0033 | 3 | 11 | 47 | 1 | 47.00 |
| 427 | 0 | US | H01L | H01L0021 | 10 | 14 | 5 | 1 | 5.00 |
| 428 | 0 | US | C30B | C30B0015 | 10 | 6 | 4 | 1 | 4.00 |
| 429 | 0 | TW | H01L | H01L0033 | 30 | 4 | 10 | 1 | 10.00 |
| 430 | 0 | TW | G09F | G09F0009 | 9 | 24 | 20 | 1 | 20.00 |
| 431 | 0 | US | C30B | C30B0019 | 1 | 3 | 14 | 1 | 14.00 |
| 432 | 0 | JP | H01L | H01L0033 | 16 | 28 | 9 | 1 | 2.25 |
| 433 | 0 | JP | H01L | H01L0033 | 11 | 22 | 3 | 1 | 3.00 |
| 434 | 0 | HK | F21K | F21K0007 | 22 | 14 | 24 | 1.5 | 8.00 |
| 435 | 0 | KR | H01L | H01L0025 | 10 | 3 | 1 | 1 | 0.25 |
| 436 | 0 | US | C30B | C30B0019 | 1 | 3 | 7 | 1 | 7.00 |
| 437 | 0 | US | H01L | H01L0021 | 8 | 6 | 9 | 1 | 9.00 |
| 438 | 0 | JP | H01L | H01L0021 | 8 | 112 | 7 | 2.5 | 1.40 |
| 439 | 0 | JP | G02F | G02F0001 | 31 | 5 | 11 | 1 | 3.67 |
| 440 | 0 | TW | H01L | H01L0027 | 66 | 6 | 1 | 1 | 0.25 |
| 441 | 0 | TW | H01L | H01L0033 | 6 | 1 | 6 | 1 | 6.00 |
| 442 | 0 | JP | C23C | C23C0016 | 14 | 13 | 2 | 3 | 0.33 |
| 443 | 0 | TW | F21K | F21K0099 | 4 | 6 | 27 | 1 | 27.00 |
| 444 | 0 | US | H01L | H01L0027 | 12 | 7 | 6 | 1 | 6.00 |
| 445 | 0 | US | C09K | C09K0011 | 10 | 3 | 26 | 1 | 26.00 |
| 446 | 0 | JP | G09F | G09F0009 | 7 | 12 | 1 | 1.5 | 0.33 |
| 447 | 0 | US | C08J | C08J0011 | 17 | 8 | 1 | 1 | 1.00 |
| 448 | 0 | US | C30B | C30B0023 | 10 | 2 | 27 | 1 | 27.00 |
| 449 | 0 | US | H01L | H01L0021 | 39 | 32 | 0 | 1 | 0.00 |
| 450 | 0 | US | C30B | C30B0019 | 14 | 5 | 11 | 1 | 11.00 |
| 451 | 0 | US | C30B | C30B0019 | 14 | 9 | 14 | 1 | 14.00 |
| 452 | 0 | TW | H01L | H01L0027 | 10 | 1 | 20 | 1 | 20.00 |
| 453 | 0 | TW | H01L | H01L0021 | 20 | 3 | 7 | 1 | 7.00 |
| 454 | 0 | US | H01L | H01L0031 | 6 | 8 | 5 | 1 | 5.00 |
| 455 | 0 | JP | H01L | H01L0033 | 26 | 10 | 1 | 1.333333333 | 0.25 |
| 456 | 0 | JP | C23C | C23C0016 | 7 | 13 | 0 | 1.666666667 | 0.00 |
| 457 | 0 | US | H01L | H01L0029 | 15 | 6 | 4 | 1 | 4.00 |
| 458 | 0 | JP | H01L | H01L0033 | 29 | 8 | 4 | 1.5 | 1.33 |
| 459 | 0 | US | H01L | H01L0033 | 7 | 8 | 7 | 1 | 7.00 |
| 460 | 0 | US | H01L | H01L0023 | 1 | 12 | 42 | 1 | 42.00 |
| 461 | 0 | TW | H01L | H01L0025 | 8 | 7 | 7 | 1 | 3.50 |
| 462 | 0 | JP | H01L | H01L0023 | 3 | 3 | 7 | 1 | 3.50 |
| 463 | 0 | JP | H01L | H01L0021 | 6 | 103 | 0 | 2.5 | 0.00 |
| 464 | 0 | US | F21K | F21K0007 | 49 | 17 | 65 | 1 | 65.00 |
| 465 | 0 | TW | F21V | F21V0005 | 19 | 3 | 8 | 1 | 1.60 |
| 466 | 0 | JP | H01L | H01L0025 | 2 | 17 | 1 | 1 | 0.50 |
| 467 | 0 | US | H01L | H01L0033 | 3 | 4 | 23 | 1 | 23.00 |
| 468 | 0 | US | H01L | H01L0033 | 11 | 15 | 12 | 1 | 12.00 |
| 469 | 0 | US | H01L | H01L0033 | 7 | 2 | 23 | 1 | 23.00 |
| 470 | 0 | CA | H01L | H01L0029 | 35 | 30 | 7 | 1 | 7.00 |
| 471 | 0 | TW | H01L | H01L0027 | 2 | 3 | 4 | 1 | 4.00 |
| 472 | 0 | TW | G03F | G03F0007 | 7 | 4 | 6 | 1 | 2.00 |
| 473 | 0 | TW | H01L | H01L0021 | 9 | 7 | 2 | 1 | 2.00 |
| 474 | 0 | US | C08F | C08F0010 | 12 | 6 | 17 | 1 | 17.00 |
| 475 | 0 | TW | H01L | H01L0029 | 10 | 8 | 2 | 1 | 2.00 |
| 476 | 0 | US | H01L | H01L0021 | 11 | 4 | 24 | 1 | 24.00 |
| 477 | 0 | JP | F21V | F21V0029 | 9 | 32 | 2 | 1 | 1.00 |
| 478 | 0 | DE | F21V | F21V0021 | 15 | 15 | 6 | 1 | 0.86 |
| 479 | 0 | US | F21S | F21S0008 | 5 | 4 | 11 | 1.25 | 2.20 |
| 480 | 0 | DE | H01L | H01L0025 | 23 | 24 | 2 | 1 | 0.33 |
| 481 | 0 | US | H01L | H01L0033 | 8 | 6 | 5 | 1 | 5.00 |
| 482 | 0 | JP | H01L | H01L0033 | 14 | 22 | 1 | 1 | 0.50 |
| 483 | 0 | US | C23F | C23F0004 | 27 | 9 | 48 | 1 | 48.00 |
| 484 | 0 | TW | H01L | H01L0021 | 8 | 9 | 2 | 1 | 2.00 |
| 485 | 0 | US | H01J | H01J0001 | 4 | 7 | 13 | 1 | 13.00 |
| 486 | 0 | US | H01L | H01L0021 | 8 | 7 | 6 | 1 | 6.00 |
| 487 | 0 | US | H01L | H01L0021 | 9 | 4 | 3 | 1 | 3.00 |
| 488 | 0 | US | H01L | H01L0023 | 76 | 46 | 2 | 1.125 | 0.22 |
| 489 | 0 | US | H01J | H01J0009 | 10 | 7 | 13 | 1 | 13.00 |
| 490 | 0 | US | H01L | H01L0033 | 4 | 6 | 2 | 1 | 2.00 |
| 491 | 0 | DE | H05B | H05B0033 | 21 | 22 | 15 | 1 | 2.50 |
| 492 | 0 | CA | F21V | F21V0007 | 40 | 21 | 19 | 1 | 6.33 |
| 493 | 0 | US | H01L | H01L0021 | 11 | 8 | 13 | 1 | 13.00 |
| 494 | 0 | US | F21S | F21S0008 | 3 | 4 | 8 | 1 | 2.67 |
| 495 | 0 | US | H01L | H01L0033 | 8 | 8 | 3 | 1 | 3.00 |
| 496 | 0 | JP | H01L | H01L0033 | 31 | 6 | 1 | 1 | 0.50 |
| 497 | 0 | US | C30B | C30B0019 | 20 | 9 | 12 | 1 | 12.00 |
| 498 | 0 | TW | H01L | H01L0021 | 20 | 13 | 5 | 1 | 5.00 |
| 499 | 0 | US | H01L | H01L0033 | 11 | 4 | 17 | 1 | 17.00 |
| 500 | 0 | JP | H01L | H01L0033 | 13 | 8 | 1 | 1.333333333 | 0.25 |
| 501 | 0 | US | H01L | H01L0033 | 12 | 27 | 4 | 2.166666667 | 0.31 |
| 502 | 0 | US | F21S | F21S0008 | 4 | 12 | 23 | 1 | 5.75 |
| 503 | 0 | JP | H01L | H01L0027 | 20 | 17 | 0 | 1 | 0.00 |
| 504 | 0 | JP | H01L | H01L0021 | 2 | 3 | 1 | 2 | 0.17 |
| 505 | 0 | JP | B08B | B08B0005 | 13 | 13 | 0 | 1 | 0.00 |
| 506 | 0 | SG | H01L | H01L0021 | 12 | 8 | 0 | 2 | 0.00 |
| 507 | 0 | TW | H01L | H01L0033 | 19 | 4 | 0 | 1 | 0.00 |
| 508 | 0 | SG | H01L | H01L0029 | 17 | 11 | 3 | 1 | 1.50 |
| 509 | 0 | US | H01L | H01L0033 | 10 | 4 | 2 | 1 | 2.00 |
| 510 | 0 | DE | H01L | H01L0023 | 13 | 11 | 0 | 1 | 0.00 |
| 511 | 0 | US | F21S | F21S0008 | 15 | 5 | 11 | 1.25 | 2.20 |
| 512 | 0 | JP | C09K | C09K0011 | 9 | 18 | 1 | 1.5 | 0.33 |
| 513 | 0 | TW | H01L | H01L0021 | 18 | 6 | 2 | 1 | 2.00 |
| 514 | 0 | JP | H01L | H01L0021 | 16 | 27 | 2 | 1.2 | 0.33 |
| 515 | 0 | JP | H01L | H01L0021 | 11 | 13 | 2 | 1.5 | 0.67 |
| 516 | 0 | TW | H01L | H01L0029 | 16 | 2 | 0 | 1 | 0.00 |
| 517 | 0 | SG | H05B | H05B0033 | 13 | 20 | 6 | 1 | 3.00 |
| 518 | 0 | US | C30B | C30B0019 | 13 | 3 | 3 | 1 | 3.00 |
| 519 | 0 | US | H01L | H01L0021 | 1 | 8 | 22 | 1 | 22.00 |
| 520 | 0 | US | F21V | F21V0029 | 20 | 16 | 30 | 1 | 10.00 |
| 521 | 0 | US | F21S | F21S0008 | 21 | 12 | 14 | 1.333333333 | 3.50 |
| 522 | 0 | TW | H01L | H01L0031 | 10 | 4 | 0 | 2.5 | 0.00 |
| 523 | 0 | JP | H01L | H01L0033 | 28 | 16 | 6 | 1 | 2.00 |
| 524 | 0 | US | H01L | H01L0033 | 1 | 8 | 3 | 1 | 3.00 |
| 525 | 0 | JP | H01L | H01L0021 | 47 | 40 | 4 | 1.666666667 | 0.40 |
| 526 | 0 | US | H01L | H01L0033 | 9 | 5 | 11 | 1 | 3.67 |
| 527 | 0 | DE | H01L | H01L0021 | 24 | 24 | 0 | 1.2 | 0.00 |
| 528 | 0 | US | H01L | H01L0023 | 7 | 17 | 3 | 1 | 3.00 |
| 529 | 0 | TW | H01L | H01L0033 | 20 | 5 | 3 | 1 | 3.00 |
| 530 | 0 | JP | H01L | H01L0029 | 9 | 10 | 2 | 1 | 0.50 |
| 531 | 0 | US | H01L | H01L0021 | 9 | 4 | 11 | 1 | 11.00 |
| 532 | 0 | US | H01L | H01L0033 | 7 | 3 | 69 | 1 | 69.00 |
| 533 | 0 | US | H01L | H01L0021 | 6 | 9 | 20 | 1 | 20.00 |
| 534 | 0 | KR | H01L | H01L0027 | 6 | 13 | 0 | 1 | 0.00 |
| 535 | 0 | US | H01L | H01L0029 | 28 | 135 | 11 | 1.142857143 | 1.38 |
| 536 | 0 | SG | H01L | H01L0029 | 18 | 26 | 0 | 1 | 0.00 |
| 537 | 0 | US | B60Q | B60Q0001 | 7 | 11 | 11 | 1 | 1.57 |
| 538 | 0 | JP | H01L | H01L0021 | 7 | 14 | 0 | 1 | 0.00 |
| 539 | 0 | JP | H01L | H01L0027 | 20 | 4 | 1 | 1 | 0.50 |
| 540 | 0 | TW | H01L | H01L0021 | 6 | 2 | 5 | 1 | 5.00 |
| 541 | 0 | US | H01L | H01L0033 | 1 | 22 | 0 | 2.166666667 | 0.00 |
| 542 | 0 | US | H01L | H01L0021 | 6 | 6 | 13 | 1 | 13.00 |
| 543 | 0 | KR | H01L | H01L0021 | 6 | 16 | 1 | 1.2 | 0.17 |
| 544 | 0 | JP | H01L | H01L0029 | 2 | 6 | 1 | 1 | 0.50 |
| 545 | 0 | US | H01L | H01L0033 | 6 | 3 | 10 | 1 | 10.00 |
| 546 | 0 | US | C30B | C30B0019 | 9 | 2 | 12 | 1 | 12.00 |
| 547 | 0 | KR | B23K | B23K0035 | 19 | 13 | 0 | 1 | 0.00 |
| 548 | 0 | TW | H01J | H01J0009 | 2 | 9 | 0 | 1 | 0.00 |
| 549 | 0 | US | G09F | G09F0009 | 18 | 5 | 13 | 1 | 13.00 |
| 550 | 0 | US | H01L | H01L0029 | 4 | 5 | 4 | 1 | 4.00 |
| 551 | 0 | JP | C30B | C30B0025 | 22 | 22 | 0 | 1 | 0.00 |
| 552 | 0 | US | G02B | G02B0005 | 21 | 16 | 2 | 1.25 | 0.40 |
| 553 | 0 | US | F21K | F21K0099 | 14 | 7 | 4 | 1 | 1.00 |
| 554 | 0 | TW | G02B | G02B0006 | 12 | 3 | 10 | 1 | 10.00 |
| 555 | 0 | KR | H01L | H01L0029 | 10 | 4 | 0 | 1.333333333 | 0.00 |
| 556 | 0 | US | F21V | F21V0014 | 21 | 13 | 2 | 1 | 2.00 |
| 557 | 0 | KR | H01L | H01L0029 | 9 | 6 | 2 | 1.333333333 | 0.50 |
| 558 | 1 | DE | H01L | H01L0029 | 18 | 15 | 1 | 1 | 0.14 |
| 559 | 0 | US | H01L | H01L0029 | 18 | 52 | 7 | 1.166666667 | 1.00 |
| 560 | 0 | JP | H01L | H01L0033 | 16 | 9 | 3 | 3 | 0.50 |
| 561 | 0 | US | H01L | H01L0033 | 7 | 3 | 4 | 1 | 4.00 |
| 562 | 0 | JP | F21V | F21V0029 | 2 | 12 | 5 | 1 | 1.67 |
| 563 | 0 | US | G21K | G21K0001 | 15 | 13 | 68 | 1 | 68.00 |
| 564 | 0 | US | C23F | C23F0004 | 5 | 12 | 24 | 1 | 24.00 |
| 565 | 0 | US | H01L | H01L0033 | 6 | 5 | 4 | 1 | 4.00 |
| 566 | 0 | TW | H01L | H01L0033 | 14 | 6 | 2 | 1 | 0.67 |
| 567 | 0 | US | C30B | C30B0019 | 10 | 14 | 1 | 1 | 1.00 |
| 568 | 0 | KR | H01L | H01L0029 | 14 | 17 | 0 | 1 | 0.00 |
| 569 | 0 | JP | H01L | H01L0021 | 24 | 25 | 0 | 1.5 | 0.00 |
| 570 | 0 | US | G02F | G02F0001 | 28 | 169 | 1 | 1 | 0.25 |
| 571 | 0 | TW | H01L | H01L0029 | 10 | 1 | 0 | 1 | 0.00 |
| 572 | 0 | TW | H01L | H01L0029 | 15 | 4 | 0 | 1.5 | 0.00 |
| 573 | 0 | US | F21V | F21V0011 | 29 | 4 | 2 | 1 | 0.67 |
| 574 | 0 | DE | G05F | G05F0001 | 22 | 22 | 5 | 1 | 0.71 |
| 575 | 0 | US | F21V | F21V0009 | 13 | 15 | 8 | 1.75 | 1.14 |
| 576 | 0 | US | H01L | H01L0023 | 21 | 10 | 20 | 1 | 6.67 |
| 577 | 0 | JP | H01L | H01L0023 | 4 | 15 | 0 | 1 | 0.00 |
| 578 | 0 | US | C30B | C30B0019 | 11 | 13 | 5 | 1 | 5.00 |
| 579 | 0 | US | H01L | H01L0021 | 22 | 54 | 13 | 2.333333333 | 0.93 |
| 580 | 0 | US | H01L | H01L0021 | 3 | 7 | 11 | 1 | 11.00 |
| 581 | 0 | JP | H01J | H01J0063 | 26 | 10 | 0 | 1 | 0.00 |
| 582 | 0 | JP | B60Q | B60Q0001 | 23 | 20 | 6 | 1.333333333 | 1.50 |
| 583 | 1 | TW | H01L | H01L0029 | 20 | 11 | 1 | 1 | 1.00 |
| 584 | 0 | KR | H01L | H01L0021 | 8 | 7 | 1 | 1.333333333 | 0.25 |
| 585 | 0 | US | H01L | H01L0021 | 19 | 45 | 8 | 1.833333333 | 0.73 |
| 586 | 0 | JP | H05B | H05B0037 | 14 | 5 | 0 | 1 | 0.00 |
| 587 | 0 | JP | H01L | H01L0021 | 5 | 29 | 0 | 1 | 0.00 |
| 588 | 0 | JP | H01L | H01L0029 | 19 | 12 | 1 | 1 | 0.25 |
| 589 | 0 | AU | H01L | H01L0025 | 51 | 140 | 2 | 2.3 | 0.09 |
| 590 | 0 | US | H01L | H01L0033 | 4 | 6 | 21 | 1 | 21.00 |
| 591 | 0 | DE | F21V | F21V0019 | 13 | 12 | 4 | 1 | 1.33 |
| 592 | 0 | TW | H01L | H01L0029 | 6 | 7 | 1 | 1 | 0.33 |
| 593 | 0 | DE | H01L | H01L0029 | 11 | 18 | 0 | 1 | 0.00 |
| 594 | 0 | US | H05B | H05B0033 | 14 | 34 | 4 | 1.166666667 | 0.57 |
| 595 | 0 | TW | H01L | H01L0033 | 4 | 3 | 0 | 1.5 | 0.00 |
| 596 | 0 | JP | H01L | H01L0021 | 30 | 20 | 0 | 1.5 | 0.00 |
| 597 | 0 | TW | H01J | H01J0001 | 18 | 8 | 0 | 1 | 0.00 |
| 598 | 1 | TW | H01L | H01L0021 | 24 | 14 | 3 | 1 | 1.50 |
| 599 | 0 | US | H01L | H01L0021 | 24 | 2 | 2 | 1 | 2.00 |
| 600 | 0 | KR | H01L | H01L0021 | 8 | 18 | 0 | 1 | 0.00 |
| 601 | 0 | DE | F21V | F21V0007 | 30 | 24 | 2 | 1.25 | 0.40 |
| 602 | 0 | US | H01L | H01L0029 | 10 | 6 | 31 | 1 | 31.00 |
| 603 | 0 | US | G21K | G21K0001 | 11 | 19 | 109 | 1 | 109.00 |
| 604 | 0 | US | H01L | H01L0023 | 13 | 9 | 71 | 1 | 71.00 |
| 605 | 0 | SG | H01L | H01L0033 | 18 | 18 | 4 | 1.2 | 0.67 |
| 606 | 0 | TW | H01L | H01L0029 | 6 | 5 | 1 | 2 | 0.50 |
| 607 | 0 | US | F21V | F21V0005 | 34 | 6 | 1 | 1 | 1.00 |
| 608 | 0 | JP | H01L | H01L0031 | 6 | 14 | 1 | 1 | 0.50 |
| 609 | 0 | JP | F21V | F21V0005 | 17 | 15 | 9 | 1 | 3.00 |
| 610 | 0 | SG | H01L | H01L0033 | 17 | 19 | 2 | 1 | 0.67 |
| 611 | 0 | DE | H05B | H05B0033 | 12 | 24 | 5 | 1.166666667 | 0.71 |
| 612 | 0 | US | H01L | H01L0021 | 13 | 1 | 35 | 1 | 35.00 |
| 613 | 0 | TW | H01L | H01L0027 | 8 | 6 | 0 | 1 | 0.00 |
| 614 | 0 | KR | H05B | H05B0033 | 6 | 14 | 2 | 1 | 0.50 |
| 615 | 0 | DE | H01L | H01L0021 | 22 | 21 | 2 | 1 | 0.29 |
| 616 | 0 | US | H01L | H01L0021 | 4 | 5 | 1 | 1 | 1.00 |
| 617 | 0 | BN | B60Q | B60Q0001 | 24 | 7 | 11 | 1.333333333 | 2.75 |
| 618 | 0 | US | H01L | H01L0033 | 9 | 5 | 14 | 1 | 14.00 |
| 619 | 0 | DE | H01L | H01L0027 | 31 | 34 | 2 | 1 | 2.00 |
| 620 | 0 | JP | H01L | H01L0033 | 8 | 40 | 1 | 1 | 0.25 |
| 621 | 0 | US | H01L | H01L0021 | 8 | 22 | 0 | 1 | 0.00 |
| 622 | 0 | DE | H01L | H01L0023 | 18 | 33 | 1 | 1 | 0.14 |
| 623 | 0 | US | F21S | F21S0009 | 11 | 5 | 3 | 1 | 3.00 |
| 624 | 0 | JP | H01L | H01L0021 | 1 | 11 | 0 | 1 | 0.00 |
| 625 | 0 | JP | H01L | H01L0033 | 35 | 28 | 1 | 1 | 0.25 |
| 626 | 0 | DE | H01L | H01L0023 | 12 | 22 | 0 | 1 | 0.00 |
| 627 | 0 | US | F21V | F21V0005 | 26 | 10 | 2 | 1 | 0.40 |
| 628 | 0 | US | H01J | H01J0009 | 5 | 11 | 0 | 1 | 0.00 |
| 629 | 0 | JP | H01L | H01L0033 | 7 | 15 | 0 | 1.5 | 0.00 |
| 630 | 0 | JP | H01L | H01L0027 | 37 | 19 | 6 | 1.333333333 | 1.50 |
| 631 | 0 | JP | H01L | H01L0029 | 17 | 20 | 0 | 1 | 0.00 |
| 632 | 0 | JP | G05F | G05F0001 | 26 | 9 | 0 | 1 | 0.00 |
| 633 | 0 | DE | F21V | F21V0005 | 19 | 29 | 3 | 1 | 1.00 |
| 634 | 0 | JP | H01L | H01L0033 | 16 | 10 | 0 | 1 | 0.00 |
| 635 | 0 | DE | F21V | F21V0007 | 48 | 32 | 3 | 1.375 | 0.27 |
| 636 | 0 | JP | H01L | H01L0033 | 7 | 5 | 0 | 1 | 0.00 |
| 637 | 0 | KY | H01L | H01L0029 | 11 | 41 | 5 | 3 | 0.56 |
| 638 | 0 | JP | F21V | F21V0021 | 22 | 17 | 4 | 1 | 0.80 |
| 639 | 0 | JP | H01L | H01L0033 | 12 | 6 | 0 | 1 | 0.00 |
| 640 | 0 | DE | H01L | H01L0033 | 16 | 15 | 0 | 1 | 0.00 |
| 641 | 0 | US | H01L | H01L0033 | 4 | 10 | 32 | 1 | 32.00 |
| 642 | 0 | DE | C09K | C09K0011 | 20 | 13 | 0 | 1.125 | 0.00 |
| 643 | 0 | US | H01L | H01L0021 | 24 | 30 | 2 | 2 | 1.00 |
| 644 | 0 | US | C30B | C30B0019 | 9 | 5 | 1 | 1 | 1.00 |
| 645 | 0 | US | C25F | C25F0003 | 32 | 12 | 35 | 1 | 35.00 |
| 646 | 0 | JP | H01L | H01L0021 | 4 | 27 | 0 | 1.75 | 0.00 |
| 647 | 0 | TW | H01L | H01L0033 | 7 | 3 | 0 | 3.5 | 0.00 |
| 648 | 0 | US | G06F | G06F0019 | 45 | 15 | 0 | 1 | 0.00 |
| 649 | 0 | US | C30B | C30B0028 | 8 | 51 | 0 | 3 | 0.00 |
| 650 | 0 | US | C30B | C30B0019 | 12 | 7 | 1 | 1 | 1.00 |
| 651 | 0 | TW | H01L | H01L0029 | 12 | 1 | 1 | 1 | 1.00 |
| 652 | 0 | DE | H01L | H01L0021 | 17 | 43 | 0 | 1 | 0.00 |
| 653 | 0 | TW | H01L | H01L0021 | 12 | 6 | 0 | 1.5 | 0.00 |
| 654 | 0 | US | C08F | C08F0004 | 11 | 29 | 10 | 1 | 10.00 |
| 655 | 0 | JP | H01J | H01J0001 | 51 | 18 | 1 | 1.166666667 | 0.14 |
| 656 | 0 | JP | G09F | G09F0013 | 20 | 6 | 0 | 1 | 0.00 |
| 657 | 0 | DE | H01L | H01L0025 | 22 | 38 | 1 | 1.285714286 | 0.11 |
| 658 | 0 | US | H01L | H01L0021 | 23 | 74 | 5 | 2.333333333 | 0.36 |
| 659 | 0 | US | H01L | H01L0029 | 22 | 38 | 1 | 1.142857143 | 0.13 |
| 660 | 0 | US | H01J | H01J0001 | 22 | 16 | 0 | 1 | 0.00 |
| 661 | 0 | DE | H01L | H01L0021 | 14 | 22 | 0 | 1 | 0.00 |
| 662 | 0 | JP | H01L | H01L0021 | 14 | 24 | 0 | 1 | 0.00 |
| 663 | 0 | US | H01L | H01L0029 | 39 | 39 | 1 | 2.285714286 | 0.06 |
| 664 | 0 | JP | H01L | H01L0023 | 16 | 21 | 0 | 1 | 0.00 |
| 665 | 0 | TW | H01J | H01J0001 | 22 | 4 | 0 | 1 | 0.00 |
| 666 | 0 | US | G02B | G02B0006 | 7 | 7 | 18 | 1 | 2.25 |
| 667 | 0 | JP | H01L | H01L0021 | 9 | 8 | 0 | 1.5 | 0.00 |
| 668 | 0 | JP | H01L | H01L0033 | 49 | 22 | 0 | 1 | 0.00 |
| 669 | 0 | JP | H01L | H01L0021 | 3 | 12 | 0 | 1.333333333 | 0.00 |
| 670 | 0 | KR | H01L | H01L0029 | 16 | 27 | 0 | 1.6 | 0.00 |
| 671 | 0 | JP | F21V | F21V0009 | 16 | 17 | 6 | 1 | 3.00 |
| 672 | 0 | US | C25F | C25F0003 | 16 | 4 | 10 | 1 | 10.00 |
| 673 | 0 | US | H01L | H01L0033 | 20 | 7 | 5 | 1 | 5.00 |
| 674 | 0 | US | H01L | H01L0021 | 5 | 8 | 14 | 1 | 14.00 |
| 675 | 0 | JP | H01L | H01L0033 | 9 | 43 | 1 | 1 | 0.25 |
| 676 | 0 | JP | H01L | H01L0033 | 13 | 15 | 0 | 1 | 0.00 |
| 677 | 0 | KR | C09K | C09K0011 | 18 | 259 | 2 | 1.529411765 | 0.08 |
| 678 | 0 | DE | C09K | C09K0011 | 4 | 11 | 0 | 1 | 0.00 |
| 679 | 0 | US | H01L | H01L0033 | 19 | 44 | 0 | 1.428571429 | 0.00 |
| 680 | 1 | US | H01L | H01L0033 | 11 | 43 | 0 | 1.428571429 | 0.00 |
| 681 | 0 | US | C08K | C08K0003 | 10 | 3 | 3 | 1 | 3.00 |
| 682 | 0 | DE | F21V | F21V0021 | 4 | 14 | 2 | 1 | 1.00 |
| 683 | 0 | KR | F21V | F21V0007 | 21 | 11 | 0 | 1 | 0.00 |
| 684 | 0 | US | H01L | H01L0027 | 25 | 27 | 1 | 1 | 1.00 |
| 685 | 0 | US | H01S | H01S0005 | 19 | 8 | 5 | 1 | 5.00 |
| 686 | 0 | TW | H01L | H01L0021 | 10 | 4 | 1 | 2 | 0.50 |
| 687 | 0 | JP | F21S | F21S0008 | 14 | 60 | 1 | 1.142857143 | 0.13 |
| 688 | 0 | JP | H01L | H01L0027 | 16 | 7 | 5 | 1 | 1.67 |
| 689 | 0 | US | H01L | H01L0029 | 9 | 59 | 0 | 1.428571429 | 0.00 |
| 690 | 0 | JP | H01L | H01L0033 | 4 | 9 | 0 | 1 | 0.00 |
| 691 | 0 | JP | H05B | H05B0033 | 41 | 174 | 1 | 1.666666667 | 0.20 |
| 692 | 0 | KR | H01L | H01L0021 | 18 | 9 | 0 | 1 | 0.00 |
| 693 | 0 | TW | H01L | H01L0033 | 11 | 3 | 1 | 1 | 1.00 |
| 694 | 0 | TW | H01L | H01L0029 | 14 | 6 | 0 | 1 | 0.00 |
| 695 | 0 | DE | H01L | H01L0023 | 21 | 25 | 2 | 1 | 0.29 |
| 696 | 0 | DE | B23K | B23K0026 | 31 | 60 | 0 | 3.5 | 0.00 |
| 697 | 0 | JP | H01L | H01L0033 | 10 | 11 | 1 | 1.5 | 0.33 |
| 698 | 0 | JP | H01L | H01L0033 | 32 | 17 | 0 | 1 | 0.00 |
| 699 | 0 | JP | H01L | H01L0031 | 9 | 35 | 0 | 1.75 | 0.00 |
| 700 | 0 | DE | H01L | H01L0021 | 27 | 27 | 0 | 1.142857143 | 0.00 |
| 701 | 0 | JP | C08G | C08G0077 | 7 | 22 | 0 | 1.2 | 0.00 |
| 702 | 0 | TW | H01J | H01J0001 | 38 | 11 | 0 | 1 | 0.00 |
| 703 | 0 | JP | H01L | H01L0033 | 1 | 28 | 0 | 1 | 0.00 |
| 704 | 0 | US | H01L | H01L0029 | 24 | 58 | 3 | 2.333333333 | 0.21 |
| 705 | 0 | TW | H01L | H01L0021 | 6 | 14 | 1 | 1.25 | 0.20 |
| 706 | 0 | TW | H01L | H01L0027 | 18 | 11 | 0 | 1 | 0.00 |
| 707 | 0 | US | F21V | F21V0029 | 5 | 21 | 2 | 1.333333333 | 0.50 |
| 708 | 0 | US | H01L | H01L0029 | 14 | 8 | 48 | 1 | 48.00 |
| 709 | 0 | US | H01S | H01S0005 | 7 | 14 | 9 | 1 | 9.00 |
| 710 | 0 | JP | H01L | H01L0033 | 8 | 50 | 2 | 1 | 0.50 |
| 711 | 0 | US | H01L | H01L0033 | 7 | 7 | 5 | 1 | 5.00 |
| 712 | 0 | US | F21V | F21V0029 | 29 | 165 | 0 | 1 | 0.00 |
| 713 | 0 | TW | H01L | H01L0021 | 18 | 14 | 0 | 2 | 0.00 |
| 714 | 0 | DE | H01S | H01S0005 | 9 | 21 | 0 | 1.125 | 0.00 |
| 715 | 0 | US | H01L | H01L0021 | 7 | 11 | 3 | 1 | 3.00 |
| 716 | 0 | SG | H01L | H01L0027 | 15 | 8 | 0 | 2 | 0.00 |
| 717 | 0 | JP | H01L | H01L0021 | 19 | 10 | 0 | 1 | 0.00 |
| 718 | 0 | US | H01L | H01L0033 | 67 | 25 | 1 | 1 | 0.33 |
| 719 | 0 | JP | H01L | H01L0029 | 16 | 25 | 1 | 1 | 0.33 |
| 720 | 0 | KR | H01L | H01L0021 | 6 | 31 | 0 | 1.333333333 | 0.00 |
| 721 | 0 | US | H01L | H01L0031 | 21 | 17 | 0 | 1 | 0.00 |
| 722 | 0 | US | F21K | F21K0099 | 13 | 2 | 37 | 1 | 37.00 |
| 723 | 0 | US | F21V | F21V0029 | 20 | 116 | 1 | 1.25 | 0.20 |
| 724 | 0 | KR | H01L | H01L0021 | 9 | 12 | 0 | 1.333333333 | 0.00 |
| 725 | 0 | US | H01L | H01L0033 | 20 | 27 | 1 | 1.6 | 0.13 |
| 726 | 0 | DE | H01L | H01L0023 | 12 | 28 | 0 | 1.125 | 0.00 |
| 727 | 0 | US | H01L | H01L0021 | 12 | 8 | 13 | 1 | 13.00 |
| 728 | 0 | TW | H01L | H01L0029 | 13 | 2 | 0 | 1 | 0.00 |
| 729 | 0 | KR | H01J | H01J0001 | 22 | 52 | 0 | 1.5 | 0.00 |
| 730 | 0 | DE | F21V | F21V0021 | 17 | 35 | 0 | 1 | 0.00 |
| 731 | 0 | US | F21S | F21S0004 | 29 | 119 | 4 | 4 | 1.00 |
| 732 | 0 | DE | H01L | H01L0023 | 8 | 38 | 0 | 1.125 | 0.00 |
| 733 | 0 | JP | H01S | H01S0005 | 20 | 155 | 0 | 1.25 | 0.00 |
| 734 | 0 | DE | H05B | H05B0033 | 18 | 22 | 0 | 1 | 0.00 |
| 735 | 0 | KY | H01L | H01L0021 | 31 | 63 | 0 | 3 | 0.00 |
| 736 | 0 | US | C30B | C30B0019 | 6 | 8 | 8 | 1 | 8.00 |
| 737 | 0 | TW | H01L | H01L0021 | 7 | 13 | 0 | 1 | 0.00 |
| 738 | 0 | KR | H01L | H01L0021 | 10 | 7 | 0 | 1.166666667 | 0.00 |
| 739 | 0 | DE | H01L | H01L0033 | 28 | 41 | 0 | 1 | 0.00 |
| 740 | 0 | KR | H01L | H01L0021 | 18 | 23 | 0 | 1.6 | 0.00 |
| 741 | 0 | US | H01L | H01L0021 | 34 | 18 | 0 | 1 | 0.00 |
| 742 | 0 | JP | H01L | H01L0027 | 4 | 14 | 0 | 1.75 | 0.00 |
| 743 | 0 | TW | H01L | H01L0027 | 68 | 7 | 0 | 1 | 0.00 |
| 744 | 0 | KR | H01L | H01L0027 | 21 | 8 | 0 | 1.333333333 | 0.00 |
| 745 | 0 | JP | H01L | H01L0029 | 24 | 126 | 1 | 1.6 | 0.13 |
| 746 | 0 | KR | H01L | H01L0029 | 4 | 4 | 0 | 1 | 0.00 |
| 747 | 0 | TW | H01L | H01L0023 | 20 | 10 | 0 | 2 | 0.00 |
| 748 | 0 | JP | C30B | C30B0025 | 17 | 32 | 0 | 1.5 | 0.00 |
| 749 | 0 | JP | H01L | H01L0029 | 8 | 30 | 0 | 1.5 | 0.00 |
| 750 | 0 | KR | H01L | H01L0033 | 5 | 11 | 0 | 1.333333333 | 0.00 |
| 751 | 0 | DE | H01S | H01S0005 | 18 | 16 | 0 | 1 | 0.00 |
| 752 | 0 | DE | F21V | F21V0003 | 11 | 6 | 0 | 1 | 0.00 |
| 753 | 0 | TW | H01L | H01L0033 | 29 | 15 | 0 | 1.25 | 0.00 |
| 754 | 0 | JP | H01S | H01S0005 | 13 | 28 | 0 | 2.333333333 | 0.00 |
| 755 | 0 | US | F21V | F21V0005 | 46 | 18 | 0 | 1.166666667 | 0.00 |
| 756 | 0 | KR | H01L | H01L0033 | 18 | 4 | 0 | 1 | 0.00 |
| 757 | 0 | JP | F21V | F21V0003 | 14 | 13 | 0 | 1 | 0.00 |
| 758 | 0 | JP | H01L | H01L0021 | 12 | 41 | 1 | 1 | 0.17 |
| 759 | 0 | US | H01L | H01L0033 | 17 | 47 | 4 | 1.25 | 0.80 |
| 760 | 0 | JP | H01L | H01L0033 | 7 | 5 | 0 | 1 | 0.00 |
| 761 | 0 | JP | H01L | H01L0027 | 11 | 35 | 1 | 1.4 | 0.14 |
| 762 | 0 | TW | H01L | H01L0021 | 10 | 2 | 0 | 1 | 0.00 |
| 763 | 0 | US | G03F | G03F0007 | 8 | 3 | 5 | 1 | 5.00 |
| 764 | 0 | TW | H01L | H01L0033 | 11 | 5 | 1 | 1 | 0.33 |
| 765 | 0 | US | H01L | H01L0031 | 18 | 93 | 3 | 1 | 0.75 |
| 766 | 0 | US | H01L | H01L0033 | 2 | 21 | 60 | 1 | 60.00 |
| 767 | 0 | JP | H01L | H01L0023 | 20 | 26 | 0 | 1.2 | 0.00 |
| 768 | 0 | JP | H01L | H01L0031 | 9 | 8 | 8 | 1.333333333 | 2.00 |
| 769 | 0 | JP | H01L | H01L0021 | 54 | 124 | 1 | 1.5 | 0.17 |
| 770 | 0 | JP | H01L | H01L0033 | 12 | 8 | 0 | 1 | 0.00 |
| 771 | 0 | US | H01L | H01L0021 | 15 | 18 | 27 | 1 | 27.00 |
| 772 | 0 | JP | H01L | H01L0035 | 49 | 143 | 1 | 2.4 | 0.08 |
| 773 | 0 | JP | H01L | H01L0027 | 28 | 23 | 0 | 1 | 0.00 |
| 774 | 0 | TW | H01L | H01L0033 | 11 | 6 | 0 | 1 | 0.00 |
| 775 | 0 | TW | H01L | H01L0021 | 13 | 24 | 1 | 7 | 0.14 |
| 776 | 0 | US | H01L | H01L0021 | 7 | 171 | 3 | 1 | 3.00 |
| 777 | 0 | FR | H01L | H01L0021 | 15 | 43 | 0 | 1 | 0.00 |
| 778 | 0 | JP | H01L | H01L0029 | 2 | 27 | 0 | 1 | 0.00 |
| 779 | 0 | US | F21K | F21K0099 | 57 | 360 | 1 | 5.666666667 | 0.03 |
| 780 | 0 | TW | H01L | H01L0021 | 17 | 17 | 0 | 1 | 0.00 |
| 781 | 0 | TW | H01J | H01J0001 | 20 | 32 | 0 | 1.6 | 0.00 |
| 782 | 0 | TW | H01L | H01L0021 | 11 | 24 | 0 | 2 | 0.00 |
| 783 | 0 | US | H01S | H01S0005 | 10 | 27 | 11 | 1 | 11.00 |
| 784 | 0 | TW | H01L | H01L0029 | 16 | 16 | 0 | 1.2 | 0.00 |
| 785 | 0 | SG | H01L | H01L0033 | 8 | 5 | 0 | 1 | 0.00 |
| 786 | 0 | US | F21S | F21S0008 | 17 | 15 | 0 | 3 | 0.00 |
| 787 | 0 | US | F21S | F21S0008 | 19 | 14 | 0 | 3 | 0.00 |
| 788 | 0 | SG | H01L | H01L0021 | 41 | 128 | 0 | 1 | 0.00 |
| 789 | 0 | US | H01L | H01L0021 | 28 | 41 | 1 | 1.25 | 0.20 |
| 790 | 0 | US | F21S | F21S0004 | 31 | 124 | 0 | 4 | 0.00 |
| 791 | 0 | US | H01J | H01J0001 | 78 | 323 | 1 | 9.666666667 | 0.02 |
| 792 | 0 | JP | H01L | H01L0033 | 1 | 28 | 0 | 1 | 0.00 |
| 793 | 0 | KY | H01L | H01L0033 | 25 | 68 | 0 | 3 | 0.00 |
| 794 | 0 | US | H01L | H01L0021 | 4 | 5 | 37 | 1 | 37.00 |
| 795 | 0 | KR | H01L | H01L0021 | 17 | 12 | 2 | 1 | 0.40 |
| 796 | 0 | JP | H01L | H01L0021 | 29 | 11 | 0 | 1 | 0.00 |
| 797 | 0 | JP | H01L | H01L0021 | 2 | 31 | 0 | 1.5 | 0.00 |
| 798 | 0 | US | C25F | C25F0003 | 11 | 3 | 6 | 1 | 6.00 |
| 799 | 0 | US | G01J | G01J0001 | 9 | 87 | 0 | 1.25 | 0.00 |
| 800 | 0 | TW | H01L | H01L0033 | 13 | 7 | 0 | 3.5 | 0.00 |
| 801 | 0 | JP | H01L | H01L0029 | 15 | 8 | 0 | 1 | 0.00 |
| 802 | 0 | DE | F21K | F21K0099 | 19 | 15 | 0 | 1 | 0.00 |
| 803 | 0 | TW | H01L | H01L0021 | 12 | 2 | 0 | 1 | 0.00 |
| 804 | 0 | KR | H01L | H01L0027 | 7 | 21 | 0 | 1 | 0.00 |
| 805 | 0 | US | H01L | H01L0033 | 27 | 3 | 5 | 1 | 5.00 |
| 806 | 0 | US | G06T | G06T0003 | 7 | 13 | 34 | 1 | 34.00 |
| 807 | 0 | BN | H01L | H01L0027 | 16 | 21 | 0 | 1 | 0.00 |
| 808 | 0 | US | H01L | H01L0033 | 16 | 12 | 0 | 1 | 0.00 |
| 809 | 0 | US | H01L | H01L0029 | 32 | 17 | 0 | 2 | 0.00 |
| 810 | 0 | JP | H01L | H01L 31 | 12 | 15 | 0 | 1 | 0.00 |
| 811 | 0 | US | H05B | H05B0037 | 30 | 19 | 0 | 1 | 0.00 |
| 812 | 0 | US | F21V | F21V0023 | 20 | 30 | 1 | 1 | 1.00 |
| 813 | 0 | US | H01L | H01L0021 | 10 | 19 | 0 | 1 | 0.00 |
| 814 | 0 | JP | H01L | H01L0033 | 5 | 21 | 0 | 1 | 0.00 |
| 815 | 0 | US | H01L | H01L0029 | 32 | 86 | 1 | 2.333333333 | 0.07 |
| 816 | 0 | TW | H01L | H01L0021 | 18 | 5 | 0 | 1 | 0.00 |
| 817 | 0 | DE | H01L | H01L0027 | 30 | 8 | 0 | 1 | 0.00 |
| 818 | 0 | CN | H01L | H01L0033 | 14 | 45 | 0 | 1 | 0.00 |
| 819 | 0 | US | H01L | H01L0021 | 17 | 7 | 7 | 1 | 7.00 |
| 820 | 0 | DE | H01J | H01J0001 | 7 | 25 | 0 | 1.142857143 | 0.00 |
| 821 | 0 | DE | F21V | F21V0029 | 18 | 19 | 1 | 1 | 0.13 |
| 822 | 0 | US | H01L | H01L0033 | 59 | 73 | 1 | 1 | 1.00 |
| 823 | 0 | US | F21V | F21V0009 | 20 | 17 | 0 | 1 | 0.00 |
| 824 | 0 | JP | H01L | H01L0021 | 18 | 17 | 1 | 1 | 0.14 |
| 825 | 0 | TW | H01L | H01L0021 | 16 | 8 | 0 | 1 | 0.00 |
| 826 | 0 | JP | H01L | H01L0021 | 10 | 13 | 0 | 1.5 | 0.00 |
| 827 | 0 | US | H01L | H01L0021 | 2 | 9 | 24 | 1 | 24.00 |
| 828 | 0 | JP | F21V | F21V0003 | 19 | 13 | 0 | 1 | 0.00 |
| 829 | 0 | US | C30B | C30B0019 | 6 | 4 | 3 | 1 | 3.00 |
| 830 | 0 | KR | H01L | H01L0029 | 10 | 10 | 0 | 1.333333333 | 0.00 |
| 831 | 0 | TW | H01L | H01L0033 | 20 | 2 | 0 | 3.5 | 0.00 |
| 832 | 0 | JP | H01L | H01L0029 | 13 | 13 | 0 | 1 | 0.00 |
| 833 | 0 | JP | H01L | H01L0021 | 5 | 35 | 0 | 2.333333333 | 0.00 |
| 834 | 0 | JP | H01L | H01L0029 | 24 | 17 | 0 | 1 | 0.00 |
| 835 | 0 | TW | H01L | H01L0021 | 5 | 15 | 0 | 1.5 | 0.00 |
| 836 | 0 | US | H01J | H01J0001 | 20 | 238 | 0 | 9.666666667 | 0.00 |
| 837 | 0 | JP | H01S | H01S0005 | 13 | 15 | 0 | 1 | 0.00 |
| 838 | 0 | DE | H01L | H01L0021 | 20 | 36 | 0 | 1.285714286 | 0.00 |
| 839 | 0 | US | C09J | C09J0005 | 5 | 16 | 1 | 1 | 1.00 |
| 840 | 0 | US | C30B | C30B0025 | 8 | 11 | 3 | 1 | 1.50 |
| 841 | 0 | US | C23C | C23C0016 | 15 | 20 | 90 | 1 | 90.00 |
| 842 | 0 | DE | H01J | H01J0001 | 27 | 34 | 0 | 1 | 0.00 |
| 843 | 0 | JP | H01L | H01L0033 | 20 | 12 | 0 | 1 | 0.00 |
| 844 | 0 | US | F21V | F21V0009 | 228 | 317 | 0 | 9.666666667 | 0.00 |
| 845 | 0 | JP | F21V | F21V0007 | 18 | 33 | 0 | 1 | 0.00 |
| 846 | 0 | JP | C09K | C09K0011 | 17 | 53 | 0 | 1.25 | 0.00 |
| 847 | 0 | US | B23K | B23K0020 | 17 | 9 | 113 | 1 | 113.00 |
| 848 | 0 | KR | H01L | H01L0029 | 20 | 30 | 1 | 1 | 0.50 |
| 849 | 0 | US | H01L | H01L0029 | 46 | 20 | 0 | 1.2 | 0.00 |
| 850 | 0 | US | H01L | H01L0021 | 8 | 36 | 11 | 1 | 11.00 |
| 851 | 0 | US | C23C | C23C0016 | 15 | 26 | 144 | 1 | 144.00 |
| 852 | 0 | TW | F21V | F21V0009 | 14 | 6 | 0 | 1 | 0.00 |
| 853 | 0 | JP | F21V | F21V0009 | 9 | 11 | 0 | 1 | 0.00 |
| 854 | 0 | TW | H01L | H01L0029 | 22 | 19 | 0 | 1 | 0.00 |
| 855 | 0 | JP | H01L | H01L0029 | 24 | 15 | 0 | 1.166666667 | 0.00 |
| 856 | 0 | KR | H01L | H01L0051 | 26 | 279 | 0 | 1 | 0.00 |
| 857 | 0 | KR | H01J | H01J0009 | 20 | 251 | 0 | 1 | 0.00 |
| 858 | 0 | TW | H01L | H01L0021 | 7 | 27 | 0 | 1.5 | 0.00 |
| 859 | 0 | DE | H01L | H01L0033 | 16 | 35 | 1 | 1 | 1.00 |
| 860 | 0 | US | C30B | C30B0025 | 10 | 4 | 5 | 1 | 5.00 |
| 861 | 0 | US | H01L | H01L0033 | 10 | 9 | 9 | 1 | 9.00 |
| 862 | 0 | US | H01L | H01L0029 | 14 | 39 | 0 | 4 | 0.00 |
| 863 | 0 | US | F21V | F21V0009 | 21 | 17 | 0 | 1 | 0.00 |
| 864 | 0 | US | H01L | H01L0033 | 12 | 23 | 0 | 1 | 0.00 |
| 865 | 0 | US | B23K | B23K0001 | 20 | 3 | 18 | 2 | 9.00 |
| 866 | 0 | DE | H05B | H05B0033 | 16 | 29 | 0 | 1.166666667 | 0.00 |
| 867 | 0 | TW | H05B | H05B0037 | 14 | 18 | 0 | 2 | 0.00 |
| 868 | 0 | DE | H01L | H01L0033 | 20 | 10 | 0 | 1 | 0.00 |
| 869 | 0 | DE | F21V | F21V0033 | 18 | 9 | 0 | 1 | 0.00 |
| 870 | 0 | DE | F21V | F21V0029 | 27 | 41 | 0 | 1 | 0.00 |
| 871 | 0 | US | H01L | H01L0031 | 5 | 12 | 0 | 1.5 | 0.00 |
| 872 | 0 | KR | H01L | H01L0021 | 6 | 17 | 0 | 1 | 0.00 |
| 873 | 0 | US | F21V | F21V0008 | 46 | 33 | 45 | 1 | 45.00 |
| 874 | 0 | US | H01L | H01L0027 | 27 | 29 | 0 | 1.166666667 | 0.00 |
| 875 | 0 | JP | H01L | H01L0033 | 2 | 21 | 0 | 1 | 0.00 |
| 876 | 0 | US | H01L | H01L0021 | 8 | 24 | 25 | 1 | 25.00 |
| 877 | 0 | KR | H01L | H01L0033 | 30 | 21 | 1 | 2 | 0.10 |
| 878 | 0 | DE | H01L | H01L0027 | 20 | 19 | 0 | 1 | 0.00 |
| 879 | 0 | JP | H01L | H01L0033 | 7 | 14 | 0 | 1.75 | 0.00 |
| 880 | 0 | TW | H05B | H05B0033 | 10 | 21 | 0 | 1 | 0.00 |
| 881 | 0 | US | H01S | H01S0005 | 7 | 11 | 13 | 1 | 13.00 |
| 882 | 0 | KR | H01L | H01L0033 | 19 | 16 | 0 | 1.8 | 0.00 |
| 883 | 0 | DE | H01L | H01L0033 | 34 | 30 | 0 | 1.285714286 | 0.00 |
| 884 | 0 | JP | H01R | H01R0013 | 8 | 12 | 0 | 1 | 0.00 |
| 885 | 0 | JP | F21V | F21V0008 | 19 | 17 | 0 | 1.333333333 | 0.00 |
| 886 | 0 | US | H01L | H01L0021 | 21 | 18 | 23 | 1 | 23.00 |
| 887 | 0 | TW | H01L | H01L0021 | 12 | 17 | 0 | 2 | 0.00 |
| 888 | 0 | DE | H01L | H01L0033 | 17 | 20 | 0 | 1 | 0.00 |
| 889 | 0 | KR | H01L | H01L0029 | 55 | 59 | 0 | 1.6 | 0.00 |
| 890 | 0 | TW | H01L | H01L0021 | 20 | 4 | 0 | 1 | 0.00 |
| 891 | 0 | JP | H01L | H01L0033 | 20 | 9 | 0 | 1 | 0.00 |
| 892 | 0 | TW | H01L | H01L0033 | 4 | 8 | 0 | 1 | 0.00 |
| 893 | 0 | JP | H01J | H01J0001 | 10 | 13 | 0 | 1 | 0.00 |
| 894 | 0 | US | C03C | C03C0017 | 8 | 21 | 26 | 1 | 26.00 |
| 895 | 0 | US | F21V | F21V0007 | 17 | 26 | 0 | 1.2 | 0.00 |
| 896 | 0 | JP | H01L | H01L0021 | 10 | 46 | 0 | 1.75 | 0.00 |
| 897 | 0 | DE | H01L | H01L0021 | 10 | 90 | 0 | 1.25 | 0.00 |
| 898 | 0 | KR | H01L | H01L0033 | 6 | 17 | 0 | 1 | 0.00 |
| 899 | 0 | US | H01L | H01L0021 | 10 | 16 | 10 | 1 | 10.00 |
| 900 | 0 | DE | C30B | C30B0025 | 17 | 34 | 0 | 1 | 0.00 |
| 901 | 0 | JP | H01L | H01L0029 | 42 | 186 | 0 | 1 | 0.00 |
| 902 | 0 | US | G01N | G01N0023 | 7 | 4 | 4 | 1 | 4.00 |
| 903 | 0 | US | F21K | F21K0099 | 36 | 290 | 0 | 5.666666667 | 0.00 |
| 904 | 0 | US | F21V | F21V0009 | 33 | 99 | 0 | 1 | 0.00 |
| 905 | 0 | KR | H01L | H01L0033 | 26 | 16 | 0 | 1.166666667 | 0.00 |
| 906 | 0 | US | H01L | H01L0021 | 15 | 10 | 67 | 1 | 67.00 |
| 907 | 0 | DE | H01L | H01L0021 | 26 | 34 | 0 | 1 | 0.00 |
| 908 | 0 | US | H05K | H05K0013 | 44 | 7 | 3 | 1 | 3.00 |
| 909 | 0 | US | H01L | H01L0033 | 14 | 263 | 0 | 1.428571429 | 0.00 |
| 910 | 0 | NL | H01L | H01L0033 | 20 | 82 | 0 | 1 | 0.00 |
| 911 | 0 | KR | H01J | H01J0005 | 6 | 13 | 0 | 1.5 | 0.00 |
| 912 | 0 | TW | H01L | H01L0021 | 24 | 4 | 0 | 1 | 0.00 |
| 913 | 0 | US | H01L | H01L0033 | 32 | 46 | 0 | 1 | 0.00 |
| 914 | 0 | US | H01L | H01L0033 | 29 | 33 | 0 | 2 | 0.00 |
| 915 | 0 | JP | H01L | H01L0033 | 16 | 14 | 0 | 1 | 0.00 |
| 916 | 0 | US | H01L | H01L0021 | 11 | 152 | 0 | 1.75 | 0.00 |
| 917 | 0 | TW | H01L | H01L0033 | 4 | 7 | 0 | 3 | 0.00 |
| 918 | 0 | US | H01L | H01L0033 | 15 | 17 | 2 | 1 | 2.00 |
| 919 | 0 | US | F21V | F21V0009 | 72 | 251 | 0 | 1 | 0.00 |
| 920 | 0 | US | H01L | H01L0021 | 18 | 81 | 0 | 1 | 0.00 |
| 921 | 0 | US | H01L | H01L0023 | 30 | 5 | 49 | 1 | 49.00 |
| 922 | 0 | US | F21V | F21V0007 | 18 | 41 | 0 | 1 | 0.00 |
| 923 | 0 | TW | H01L | H01L0021 | 20 | 4 | 0 | 1 | 0.00 |
| 924 | 0 | US | H01L | H01L0021 | 69 | 23 | 34 | 1 | 34.00 |
| 925 | 0 | TW | H01L | H01L0033 | 8 | 5 | 0 | 1 | 0.00 |
| 926 | 0 | SG | H01L | H01L0023 | 20 | 2 | 0 | 1 | 0.00 |
| 927 | 0 | TW | H01L | H01L0021 | 7 | 8 | 0 | 7 | 0.00 |
| 928 | 0 | TW | H01L | H01L0031 | 14 | 9 | 0 | 1 | 0.00 |
| 929 | 0 | TW | H01L | H01L0033 | 4 | 56 | 0 | 4.6 | 0.00 |
| 930 | 0 | US | H01L | H01L0033 | 19 | 76 | 0 | 1.166666667 | 0.00 |
| 931 | 0 | US | G02B | G02B0003 | 26 | 3 | 37 | 1 | 37.00 |
| 932 | 0 | US | H01L | H01L0021 | 30 | 88 | 0 | 1 | 0.00 |
| 933 | 0 | JP | H01L | H01L0029 | 6 | 5 | 0 | 1 | 0.00 |
| 934 | 0 | DE | H01L | H01L0033 | 21 | 18 | 0 | 1 | 0.00 |
| 935 | 0 | JP | F21V | F21V0029 | 22 | 56 | 0 | 2.25 | 0.00 |
| 936 | 0 | JP | G02F | G02F0001 | 15 | 18 | 0 | 1 | 0.00 |
| 937 | 0 | JP | H01L | H01L0021 | 15 | 22 | 0 | 1 | 0.00 |
| 938 | 0 | US | H01L | H01L0029 | 25 | 66 | 0 | 2.333333333 | 0.00 |
| 939 | 0 | JP | H01L | H01L0033 | 15 | 29 | 0 | 1 | 0.00 |
| 940 | 0 | US | H01L | H01L0021 | 20 | 67 | 0 | 2 | 0.00 |
| 941 | 0 | US | H01L | H01L0029 | 16 | 17 | 0 | 2 | 0.00 |
| 942 | 0 | US | H01L | H01L0033 | 28 | 155 | 0 | 1 | 0.00 |
| 943 | 0 | JP | H01L | H01L0033 | 20 | 97 | 0 | 3.111111111 | 0.00 |
| 944 | 0 | JP | H01L | H01L0033 | 13 | 30 | 0 | 1.5 | 0.00 |
| 945 | 0 | US | H01L | H01L0021 | 25 | 51 | 0 | 1 | 0.00 |
| 946 | 0 | US | H01L | H01L0021 | 18 | 98 | 0 | 1 | 0.00 |
| 947 | 0 | US | H01L | H01L0029 | 7 | 226 | 0 | 8.625 | 0.00 |
| 948 | 0 | US | B23K | B23K0001 | 15 | 12 | 18 | 1 | 18.00 |
| 949 | 0 | US | F21V | F21V0029 | 28 | 82 | 0 | 1 | 0.00 |
| 950 | 0 | US | H01L | H01L0023 | 16 | 39 | 0 | 1 | 0.00 |
| 951 | 0 | TW | H01L | H01L0033 | 18 | 5 | 16 | 2 | 8.00 |
| 952 | 0 | JP | H01L | H01L0021 | 1 | 21 | 7 | 3 | 2.33 |
| 953 | 0 | JP | H01L | H01L0027 | 32 | 30 | 0 | 3 | 0.00 |
| 954 | 0 | JP | H01L | H01L0027 | 31 | 61 | 0 | 1.666666667 | 0.00 |
| 955 | 0 | US | H01L | H01L0033 | 11 | 4 | 4 | 1 | 4.00 |
| 956 | 0 | US | H01L | H01L0027 | 11 | 26 | 71 | 1 | 71.00 |
| 957 | 0 | US | H01L | H01L0027 | 14 | 36 | 69 | 1 | 69.00 |
| 958 | 0 | US | H05B | H05B0033 | 5 | 7 | 6 | 1 | 6.00 |
| 959 | 0 | US | H01L | H01L0021 | 16 | 12 | 14 | 1 | 14.00 |
| 960 | 0 | US | H01L | H01L0021 | 2 | 1 | 19 | 1 | 9.50 |
| 961 | 0 | US | H01L | H01L0021 | 10 | 11 | 11 | 1 | 11.00 |
| 962 | 0 | US | H01L | H01L0029 | 21 | 7 | 31 | 1 | 31.00 |
| 963 | 0 | US | H01L | H01L0023 | 9 | 19 | 29 | 1 | 29.00 |
| 964 | 0 | US | H01L | H01L0033 | 1 | 5 | 8 | 1 | 8.00 |
| 965 | 0 | US | C09K | C09K0011 | 5 | 7 | 3 | 1.333333333 | 0.75 |
| 966 | 0 | US | H05B | H05B0033 | 7 | 3 | 10 | 1 | 5.00 |
| 967 | 0 | US | H01L | H01L0021 | 12 | 22 | 9 | 1 | 9.00 |
| 968 | 0 | US | H01L | H01L0023 | 24 | 6 | 14 | 1 | 14.00 |
| 969 | 0 | US | C30B | C30B0011 | 3 | 4 | 3 | 1 | 3.00 |
| 970 | 0 | US | H01L | H01L0033 | 2 | 3 | 7 | 1 | 7.00 |
| 971 | 0 | US | H01L | H01L0033 | 17 | 7 | 3 | 1 | 3.00 |
| 972 | 0 | JP | H01L | H01L0021 | 3 | 4 | 7 | 1 | 2.33 |
| 973 | 0 | US | C23C | C23C0016 | 13 | 22 | 8 | 1 | 4.00 |
| 974 | 0 | US | C08G | C08G0018 | 9 | 9 | 7 | 1 | 7.00 |
| 975 | 0 | US | H01L | H01L0033 | 5 | 4 | 4 | 1 | 4.00 |
| 976 | 0 | US | H01L | H01L0021 | 23 | 50 | 17 | 1 | 17.00 |
| 977 | 0 | US | C30B | C30B0013 | 7 | 0 | 5 | 1.8 | 0.56 |
| 978 | 0 | US | H01L | H01L0023 | 17 | 3 | 7 | 1 | 7.00 |
| 979 | 0 | US | C23C | C23C0016 | 6 | 9 | 13 | 1 | 13.00 |
| 980 | 0 | US | C23C | C23C0008 | 28 | 0 | 3 | 1 | 3.00 |
| 981 | 0 | US | C30B | C30B0023 | 32 | 24 | 2 | 1 | 2.00 |
| 982 | 0 | US | H01L | H01L0021 | 24 | 6 | 5 | 1 | 5.00 |
| 983 | 0 | US | C08J | C08J0003 | 7 | 7 | 4 | 1 | 4.00 |
| 984 | 0 | US | H01L | H01L0021 | 15 | 16 | 3 | 1 | 3.00 |
| 985 | 0 | US | H01L | H01L0021 | 22 | 8 | 12 | 1 | 12.00 |
| 986 | 0 | US | C30B | C30B0013 | 5 | 0 | 4 | 1.8 | 0.44 |
| 987 | 0 | US | H01L | H01L0021 | 20 | 21 | 100 | 1 | 100.00 |
| 988 | 0 | US | G02F | G02F0001 | 15 | 26 | 15 | 1 | 15.00 |
| 989 | 0 | DE | H01L | H01L0021 | 20 | 0 | 10 | 1 | 5.00 |
| 990 | 0 | JP | C30B | C30B0019 | 6 | 0 | 9 | 1.2 | 1.50 |
| 991 | 0 | US | H01L | H01L0025 | 6 | 11 | 20 | 1.5 | 6.67 |
| 992 | 0 | JP | C30B | C30B0019 | 2 | 0 | 4 | 1 | 2.00 |
| 993 | 0 | JP | H01L | H01L0021 | 7 | 0 | 2 | 1 | 1.00 |
| 994 | 0 | US | C23C | C23C0016 | 20 | 8 | 14 | 1 | 14.00 |
| 995 | 0 | US | H01L | H01L0021 | 20 | 12 | 8 | 1 | 8.00 |
| 996 | 0 | US | H01L | H01L0021 | 15 | 4 | 27 | 1 | 27.00 |
| 997 | 0 | US | H01L | H01L0021 | 9 | 4 | 0 | 1 | 0.00 |
| 998 | 0 | US | H01L | H01L0033 | 8 | 0 | 35 | 1 | 35.00 |
| 999 | 0 | US | H01L | H01L0033 | 19 | 21 | 18 | 1 | 18.00 |
| 1000 | 0 | US | B24D | B24D0003 | 25 | 26 | 25 | 1 | 25.00 |
| 1001 | 0 | SU | H05B | H05B0033 | 17 | 2 | 9 | 1.181818182 | 0.69 |
| 1002 | 0 | US | G02B | G02B0006 | 27 | 6 | 23 | 1 | 23.00 |
| 1003 | 0 | US | H01L | H01L0021 | 22 | 10 | 241 | 1 | 241.00 |
| 1004 | 0 | US | H01L | H01L0021 | 12 | 35 | 12 | 1.5 | 1.00 |
| 1005 | 0 | JP | G02B | G02B0006 | 5 | 6 | 10 | 1.25 | 2.00 |
| 1006 | 1 | US | H05K | H05K0007 | 21 | 19 | 25 | 1 | 3.57 |
| 1007 | 0 | US | H01L | H01L0033 | 14 | 30 | 16 | 1 | 5.33 |
| 1008 | 0 | US | H01L | H01L0021 | 18 | 5 | 4 | 1 | 4.00 |
| 1009 | 0 | JP | C30B | C30B0025 | 6 | 6 | 5 | 1 | 1.67 |
| 1010 | 0 | JP | H01L | H01L0033 | 14 | 44 | 62 | 1.75 | 8.86 |
| 1011 | 0 | JP | H01B | H01B0001 | 1 | 1 | 0 | 2.5 | 0.00 |
| 1012 | 0 | JP | H01L | H01L0021 | 6 | 18 | 89 | 1 | 29.67 |
| 1013 | 0 | US | H01L | H01L0021 | 15 | 7 | 43 | 1 | 43.00 |
| 1014 | 0 | JP | H01B | H01B0001 | 1 | 1 | 0 | 2.5 | 0.00 |
| 1015 | 0 | US | C30B | C30B0023 | 21 | 9 | 5 | 1 | 5.00 |
| 1016 | 0 | US | H01L | H01L0029 | 29 | 29 | 1 | 1 | 1.00 |
| 1017 | 0 | US | H01J | H01J0037 | 11 | 5 | 24 | 1 | 24.00 |
| 1018 | 0 | US | H01L | H01L0029 | 3 | 16 | 1 | 1 | 1.00 |
| 1019 | 0 | JP | H01L | H01L0021 | 12 | 18 | 94 | 7.714285714 | 1.74 |
| 1020 | 0 | US | H01L | H01L0021 | 14 | 101 | 157 | 1 | 157.00 |
| 1021 | 1 | US | H01L | H01L0031 | 4 | 7 | 10 | 2 | 5.00 |
| 1022 | 0 | JP | C23C | C23C0016 | 32 | 4 | 127 | 2 | 12.70 |
| 1023 | 0 | JP | C09K | C09K0011 | 20 | 1 | 17 | 1.5 | 5.67 |
| 1024 | 0 | JP | H01L | H01L0021 | 11 | 6 | 6 | 1 | 1.50 |
| 1025 | 0 | JP | H01L | H01L0033 | 11 | 31 | 29 | 1.8 | 3.22 |
| 1026 | 0 | JP | H01L | H01L0033 | 32 | 19 | 17 | 7 | 0.61 |
| 1027 | 0 | JP | H01L | H01L0021 | 37 | 18 | 94 | 7.714285714 | 1.74 |
| 1028 | 0 | JP | H01L | H01L0033 | 18 | 30 | 15 | 2.8 | 1.07 |
| 1029 | 0 | JP | C30B | C30B0029 | 10 | 11 | 16 | 1.6 | 2.00 |
| 1030 | 0 | JP | H01L | H01L0021 | 6 | 6 | 0 | 1.5 | 0.00 |
| 1031 | 0 | JP | H01L | H01L0021 | 5 | 18 | 67 | 7.714285714 | 1.24 |
| 1032 | 0 | JP | H01L | H01L0033 | 6 | 35 | 14 | 1.8 | 1.56 |
| 1033 | 0 | JP | C30B | C30B0025 | 21 | 1 | 13 | 2 | 3.25 |
| 1034 | 0 | JP | H01L | H01L0033 | 38 | 11 | 98 | 4.666666667 | 3.50 |
| 1035 | 0 | JP | H01L | H01L0033 | 19 | 21 | 28 | 1.6 | 3.50 |
| 1036 | 0 | US | H01L | H01L0033 | 14 | 19 | 5 | 1 | 1.67 |
| 1037 | 0 | JP | H01L | H01L0033 | 11 | 7 | 7 | 1.5 | 2.33 |
| 1038 | 0 | US | H01L | H01L0033 | 4 | 5 | 1 | 1 | 1.00 |
| 1039 | 0 | US | H01L | H01L0033 | 46 | 21 | 227 | 1 | 227.00 |
| 1040 | 1 | JP | H01L | H01L0021 | 26 | 17 | 37 | 7.714285714 | 0.69 |
| 1041 | 0 | JP | H01L | H01L0033 | 8 | 52 | 9 | 1.8 | 1.00 |
| 1042 | 0 | JP | C23C | C23C0016 | 30 | 22 | 207 | 4.25 | 6.09 |
| 1043 | 0 | JP | H01L | H01L0029 | 20 | 3 | 46 | 1 | 11.50 |
| 1044 | 0 | JP | H01L | H01L0021 | 7 | 29 | 163 | 4.777777778 | 3.79 |
| 1045 | 0 | US | H01L | H01L0033 | 20 | 17 | 38 | 1 | 38.00 |
| 1046 | 0 | JP | B41J | B41J0002 | 8 | 9 | 25 | 2 | 3.13 |
| 1047 | 0 | JP | H01L | H01L0029 | 13 | 30 | 45 | 7.714285714 | 0.83 |
| 1048 | 1 | US | H01L | H01L0031 | 14 | 11 | 3 | 2 | 1.50 |
| 1049 | 0 | KR | H05B | H05B0033 | 20 | 3 | 8 | 1 | 1.60 |
| 1050 | 0 | US | G02B | G02B0006 | 31 | 8 | 4 | 1 | 4.00 |
| 1051 | 0 | JP | G03F | G03F0007 | 5 | 15 | 1 | 1.75 | 0.14 |
| 1052 | 0 | JP | H01S | H01S0005 | 6 | 16 | 3 | 1 | 0.75 |
| 1053 | 0 | US | H01S | H01S0005 | 26 | 3 | 7 | 1 | 7.00 |
| 1054 | 0 | US | H01S | H01S0005 | 34 | 27 | 125 | 1 | 125.00 |
| 1055 | 0 | JP | H01L | H01L0033 | 9 | 5 | 37 | 4 | 4.63 |
| 1056 | 0 | FR | C30B | C30B0029 | 30 | 7 | 37 | 2.333333333 | 1.32 |
| 1057 | 0 | JP | H01L | H01L0021 | 33 | 5 | 99 | 1 | 33.00 |
| 1058 | 0 | CA | H01S | H01S0005 | 9 | 10 | 4 | 1.5 | 0.67 |
| 1059 | 0 | JP | H01L | H01L0029 | 16 | 2 | 14 | 1 | 7.00 |
| 1060 | 0 | US | H01L | H01L0021 | 17 | 6 | 1 | 1 | 1.00 |
| 1061 | 0 | JP | H01L | H01L0033 | 12 | 2 | 3 | 1 | 0.60 |
| 1062 | 0 | US | C30B | C30B0025 | 16 | 20 | 9 | 1 | 9.00 |
| 1063 | 0 | JP | H01L | H01L0033 | 11 | 12 | 0 | 2 | 0.00 |
| 1064 | 0 | JP | H01L | H01L0033 | 6 | 21 | 27 | 1 | 6.75 |
| 1065 | 0 | US | H01L | H01L0021 | 27 | 0 | 27 | 1 | 27.00 |
| 1066 | 0 | PL | C30B | C30B0029 | 6 | 5 | 17 | 1 | 2.43 |
| 1067 | 0 | JP | H01L | H01L0033 | 12 | 6 | 3 | 1 | 0.60 |
| 1068 | 0 | US | C30B | C30B0025 | 13 | 19 | 19 | 1 | 19.00 |
| 1069 | 0 | US | H01L | H01L0027 | 24 | 25 | 12 | 1 | 12.00 |
| 1070 | 0 | CA | H01L | H01L0025 | 38 | 22 | 106 | 2.375 | 5.58 |
| 1071 | 0 | JP | H01L | H01L0023 | 6 | 8 | 1 | 1.666666667 | 0.10 |
| 1072 | 0 | CA | C09K | C09K0011 | 34 | 14 | 87 | 1 | 21.75 |
| 1073 | 0 | JP | H01L | H01L0033 | 14 | 18 | 3 | 1.166666667 | 0.43 |
| 1074 | 0 | JP | H01L | H01L0033 | 12 | 15 | 47 | 3.666666667 | 4.27 |
| 1075 | 0 | JP | C30B | C30B0025 | 9 | 6 | 17 | 1.333333333 | 4.25 |
| 1076 | 0 | JP | H01L | H01L0021 | 20 | 12 | 13 | 1.75 | 1.86 |
| 1077 | 0 | JP | C30B | C30B0025 | 9 | 126 | 0 | 1 | 0.00 |
| 1078 | 0 | JP | C30B | C30B0029 | 16 | 13 | 5 | 1 | 1.25 |
| 1079 | 0 | US | F21S | F21S0008 | 11 | 9 | 4 | 1 | 4.00 |
| 1080 | 0 | JP | H01L | H01L0033 | 11 | 5 | 23 | 1.8 | 2.56 |
| 1081 | 0 | JP | H01L | H01L0021 | 9 | 9 | 7 | 1.333333333 | 0.88 |
| 1082 | 0 | JP | H01L | H01L0033 | 6 | 9 | 12 | 2 | 1.50 |
| 1083 | 0 | CA | H01L | H01L0033 | 24 | 12 | 46 | 1.25 | 9.20 |
| 1084 | 0 | US | H01L | H01L0021 | 9 | 35 | 60 | 1.3 | 4.62 |
| 1085 | 0 | US | C23C | C23C0016 | 14 | 23 | 17 | 1 | 17.00 |
| 1086 | 0 | US | H01L | H01L0021 | 18 | 15 | 6 | 1 | 6.00 |
| 1087 | 0 | JP | H01L | H01L0023 | 14 | 11 | 1 | 1.285714286 | 0.11 |
| 1088 | 0 | JP | H01L | H01L0033 | 16 | 27 | 20 | 5 | 1.00 |
| 1089 | 0 | JP | C30B | C30B0025 | 51 | 17 | 40 | 1.625 | 3.08 |
| 1090 | 0 | JP | C30B | C30B0029 | 21 | 16 | 55 | 1.571428571 | 5.00 |
| 1091 | 0 | JP | H01L | H01L0033 | 8 | 2 | 19 | 2 | 1.90 |
| 1092 | 0 | JP | H01L | H01L0033 | 23 | 20 | 0 | 1 | 0.00 |
| 1093 | 0 | CA | H01L | H01L0033 | 18 | 53 | 55 | 5.285714286 | 1.49 |
| 1094 | 0 | US | H01L | H01L0033 | 54 | 11 | 26 | 1 | 26.00 |
| 1095 | 0 | US | G02B | G02B0006 | 17 | 3 | 8 | 1.333333333 | 2.00 |
| 1096 | 0 | JP | H01L | H01L0021 | 5 | 1 | 1 | 1.2 | 0.17 |
| 1097 | 0 | CA | H01L | H01L0023 | 20 | 8 | 19 | 1.2 | 3.17 |
| 1098 | 0 | JP | C30B | C30B0029 | 13 | 9 | 0 | 1.5 | 0.00 |
| 1099 | 0 | US | H01L | H01L0029 | 20 | 14 | 2 | 1 | 2.00 |
| 1100 | 0 | JP | H01L | H01L0033 | 8 | 8 | 6 | 2 | 0.60 |
| 1101 | 0 | JP | H01L | H01L0029 | 6 | 69 | 9 | 7.714285714 | 0.17 |
| 1102 | 0 | CA | H01L | H01L0021 | 14 | 9 | 19 | 1.2 | 3.17 |
| 1103 | 0 | JP | H01L | H01L0021 | 11 | 37 | 5 | 1.333333333 | 0.63 |
| 1104 | 0 | US | H01L | H01L0033 | 5 | 2 | 23 | 1 | 23.00 |
| 1105 | 0 | CA | H01L | H01L0033 | 19 | 60 | 25 | 5.285714286 | 0.68 |
| 1106 | 0 | JP | C30B | C30B0029 | 6 | 13 | 2 | 1.6 | 0.25 |
| 1107 | 0 | JP | C23C | C23C0016 | 14 | 11 | 2 | 2.4 | 0.17 |
| 1108 | 0 | CA | H01L | H01L0033 | 31 | 29 | 22 | 1 | 5.50 |
| 1109 | 0 | JP | C30B | C30B0029 | 21 | 20 | 5 | 1.571428571 | 0.45 |
| 1110 | 0 | JP | C30B | C30B0025 | 4 | 22 | 6 | 1.333333333 | 0.75 |
| 1111 | 0 | US | H01L | H01L0021 | 52 | 21 | 42 | 1 | 42.00 |
| 1112 | 0 | CA | H01L | H01L0021 | 7 | 20 | 13 | 1 | 2.60 |
| 1113 | 0 | JP | H01L | H01L0033 | 17 | 3 | 13 | 2 | 2.17 |
| 1114 | 0 | JP | H01S | H01S0005 | 7 | 3 | 5 | 1 | 1.25 |
| 1115 | 0 | US | H01L | H01L0031 | 46 | 15 | 3 | 1.333333333 | 0.38 |
| 1116 | 0 | DE | H01S | H01S0005 | 11 | 23 | 1 | 1 | 0.14 |
| 1117 | 0 | JP | H01L | H01L0029 | 10 | 3 | 2 | 1 | 0.67 |
| 1118 | 0 | JP | H01L | H01L0033 | 18 | 6 | 16 | 1.666666667 | 3.20 |
| 1119 | 0 | JP | H01L | H01L0021 | 4 | 9 | 3 | 2.6 | 0.23 |
| 1120 | 0 | CA | H01L | H01L0033 | 15 | 24 | 5 | 1.5 | 0.83 |
| 1121 | 0 | JP | C30B | C30B0029 | 10 | 16 | 1 | 1.666666667 | 0.20 |
| 1122 | 0 | JP | C30B | C30B0029 | 22 | 21 | 4 | 1.333333333 | 0.50 |
| 1123 | 0 | US | H01L | H01L0033 | 8 | 10 | 98 | 1 | 98.00 |
| 1124 | 0 | JP | C30B | C30B0029 | 9 | 13 | 2 | 1.25 | 0.40 |
| 1125 | 0 | JP | H01L | H01L0033 | 11 | 3 | 0 | 2.25 | 0.00 |
| 1126 | 0 | CA | C09K | C09K0011 | 28 | 12 | 47 | 1.5 | 7.83 |
| 1127 | 0 | US | H01L | H01L0021 | 15 | 54 | 53 | 1.8 | 2.94 |
| 1128 | 0 | JP | H01L | H01L0033 | 7 | 11 | 7 | 2 | 0.70 |
| 1129 | 0 | JP | H01S | H01S0005 | 6 | 7 | 2 | 2.75 | 0.18 |
| 1130 | 0 | JP | C30B | C30B0025 | 6 | 26 | 15 | 2.166666667 | 1.15 |
| 1131 | 0 | JP | F21V | F21V0007 | 3 | 8 | 23 | 1.4 | 3.29 |
| 1132 | 0 | US | G03F | G03F0001 | 14 | 7 | 29 | 1 | 29.00 |
| 1133 | 0 | CA | C09K | C09K0011 | 14 | 13 | 39 | 1 | 9.75 |
| 1134 | 0 | US | H01L | H01L0021 | 8 | 5 | 3 | 1 | 3.00 |
| 1135 | 0 | US | H01L | H01L0021 | 6 | 42 | 16 | 1.3 | 1.23 |
| 1136 | 0 | JP | H01L | H01L0029 | 18 | 68 | 16 | 7.714285714 | 0.30 |
| 1137 | 0 | CA | H01L | H01L0033 | 10 | 18 | 56 | 1.222222222 | 5.09 |
| 1138 | 0 | JP | C30B | C30B0025 | 5 | 129 | 4 | 2 | 0.67 |
| 1139 | 0 | JP | H01L | H01L0023 | 18 | 29 | 1 | 1.333333333 | 0.25 |
| 1140 | 0 | JP | C30B | C30B0029 | 10 | 4 | 1 | 1.4 | 0.14 |
| 1141 | 0 | JP | H01S | H01S0005 | 9 | 23 | 0 | 1.333333333 | 0.00 |
| 1142 | 0 | JP | H01L | H01L0033 | 4 | 9 | 32 | 2 | 3.20 |
| 1143 | 0 | CA | H01L | H01L0021 | 15 | 14 | 17 | 1.75 | 2.43 |
| 1144 | 0 | CA | C09K | C09K0011 | 30 | 11 | 50 | 1 | 8.33 |
| 1145 | 0 | GB | G02B | G02B0006 | 31 | 7 | 2 | 1 | 0.20 |
| 1146 | 0 | GB | H01S | H01S0005 | 22 | 42 | 3 | 2.545454545 | 0.11 |
| 1147 | 0 | JP | H01S | H01S0003 | 6 | 5 | 2 | 1.166666667 | 0.29 |
| 1148 | 0 | JP | C30B | C30B0023 | 5 | 3 | 6 | 1 | 3.00 |
| 1149 | 0 | JP | H01L | H01L0033 | 5 | 5 | 8 | 1.4 | 1.14 |
| 1150 | 0 | JP | C30B | C30B0029 | 9 | 126 | 13 | 2 | 2.17 |
| 1151 | 0 | CA | H01L | H01L0033 | 30 | 10 | 12 | 5.285714286 | 0.32 |
| 1152 | 0 | US | H01L | H01L0021 | 21 | 6 | 24 | 1 | 24.00 |
| 1153 | 0 | US | H05B | H05B0033 | 17 | 12 | 20 | 1 | 20.00 |
| 1154 | 0 | JP | C23C | C23C0016 | 11 | 5 | 3 | 3 | 0.33 |
| 1155 | 0 | JP | C30B | C30B0025 | 21 | 3 | 5 | 2 | 0.83 |
| 1156 | 0 | CA | C09K | C09K0011 | 14 | 21 | 37 | 1.75 | 5.29 |
| 1157 | 0 | JP | H01L | H01L0021 | 13 | 19 | 1 | 1.75 | 0.14 |
| 1158 | 0 | JP | H01S | H01S0005 | 16 | 3 | 5 | 1.5 | 0.56 |
| 1159 | 0 | JP | C01G | C01G0015 | 12 | 51 | 24 | 8.235294118 | 0.17 |
| 1160 | 1 | CA | H01L | H01L0025 | 36 | 22 | 117 | 2.375 | 6.16 |
| 1161 | 0 | CA | H01S | H01S0005 | 9 | 21 | 11 | 3.25 | 0.85 |
| 1162 | 0 | GB | G02B | G02B0006 | 8 | 18 | 0 | 1 | 0.00 |
| 1163 | 0 | US | H01L | H01L0033 | 72 | 26 | 20 | 2 | 1.67 |
| 1164 | 0 | JP | C30B | C30B0023 | 16 | 7 | 13 | 1.333333333 | 3.25 |
| 1165 | 0 | GB | G02F | G02F0001 | 34 | 35 | 2 | 1.222222222 | 0.18 |
| 1166 | 0 | JP | C30B | C30B0023 | 20 | 6 | 21 | 1.666666667 | 4.20 |
| 1167 | 0 | JP | H01L | H01L0033 | 7 | 14 | 19 | 2.6 | 1.46 |
| 1168 | 0 | JP | H01S | H01S0005 | 28 | 24 | 6 | 1.333333333 | 1.50 |
| 1169 | 0 | JP | H01L | H01L0033 | 8 | 58 | 22 | 4.777777778 | 0.51 |
| 1170 | 0 | JP | H01L | H01L0033 | 9 | 1 | 1 | 1.5 | 0.17 |
| 1171 | 0 | US | B41J | B41J0002 | 4 | 1 | 12 | 1 | 12.00 |
| 1172 | 0 | JP | H01L | H01L0033 | 13 | 14 | 16 | 1.428571429 | 1.60 |
| 1173 | 0 | US | H01L | H01L0021 | 14 | 7 | 29 | 1 | 29.00 |
| 1174 | 0 | JP | H05B | H05B0033 | 12 | 5 | 0 | 1 | 0.00 |
| 1175 | 0 | JP | C30B | C30B0025 | 12 | 33 | 18 | 2.25 | 1.00 |
| 1176 | 0 | JP | H01L | H01L0033 | 7 | 4 | 2 | 1.285714286 | 0.22 |
| 1177 | 0 | CA | H01L | H01L0033 | 26 | 27 | 50 | 1.5 | 5.56 |
| 1178 | 0 | US | C23C | C23C0008 | 6 | 25 | 8 | 1 | 8.00 |
| 1179 | 0 | JP | C30B | C30B0025 | 6 | 3 | 4 | 1.6 | 0.50 |
| 1180 | 0 | JP | C30B | C30B0025 | 17 | 8 | 0 | 2 | 0.00 |
| 1181 | 0 | BE | C01G | C01G0009 | 26 | 25 | 7 | 1.333333333 | 1.75 |
| 1182 | 0 | JP | C30B | C30B0025 | 10 | 12 | 1 | 1.6 | 0.13 |
| 1183 | 0 | JP | H01L | H01L0021 | 7 | 6 | 3 | 3.333333333 | 0.30 |
| 1184 | 0 | JP | C09K | C09K0011 | 20 | 9 | 0 | 1.75 | 0.00 |
| 1185 | 0 | JP | C30B | C30B0029 | 6 | 2 | 2 | 1.333333333 | 0.25 |
| 1186 | 0 | US | H01L | H01L0027 | 8 | 4 | 7 | 1 | 7.00 |
| 1187 | 0 | JP | C30B | C30B0025 | 37 | 17 | 5 | 2 | 0.50 |
| 1188 | 0 | JP | H01L | H01L0033 | 13 | 8 | 5 | 1.8 | 0.56 |
| 1189 | 0 | JP | B41J | B41J0002 | 4 | 15 | 0 | 1.571428571 | 0.00 |
| 1190 | 0 | JP | G02F | G02F0001 | 6 | 21 | 3 | 3.5 | 0.21 |
| 1191 | 0 | GB | H01S | H01S0005 | 21 | 48 | 1 | 1.555555556 | 0.07 |
| 1192 | 0 | JP | H01L | H01L0021 | 4 | 31 | 30 | 2.333333333 | 4.29 |
| 1193 | 0 | US | H01L | H01L0033 | 24 | 30 | 9 | 2 | 0.75 |
| 1194 | 0 | BE | C01G | C01G0009 | 14 | 25 | 8 | 1.333333333 | 2.00 |
| 1195 | 0 | US | H01L | H01L0033 | 57 | 93 | 71 | 8.8 | 0.81 |
| 1196 | 0 | JP | H01L | H01L0023 | 6 | 24 | 2 | 1.2 | 0.33 |
| 1197 | 0 | JP | H01L | H01L0033 | 16 | 13 | 40 | 2.2 | 1.82 |
| 1198 | 0 | CA | H01L | H01L0033 | 18 | 2 | 31 | 2.666666667 | 3.88 |
| 1199 | 0 | US | H01L | H01L0033 | 36 | 38 | 15 | 8.8 | 0.17 |
| 1200 | 0 | JP | C30B | C30B0029 | 16 | 8 | 1 | 2.333333333 | 0.14 |
| 1201 | 0 | US | H01L | H01L0033 | 32 | 23 | 156 | 1 | 156.00 |
| 1202 | 0 | JP | C23C | C23C0016 | 11 | 87 | 5 | 4.25 | 0.15 |
| 1203 | 0 | JP | H01L | H01L0033 | 6 | 12 | 3 | 2 | 0.38 |
| 1204 | 0 | JP | H01L | H01L0021 | 10 | 4 | 2 | 1.6 | 0.25 |
| 1205 | 0 | US | H01L | H01L0033 | 4 | 8 | 76 | 1 | 76.00 |
| 1206 | 0 | US | C30B | C30B0033 | 29 | 31 | 25 | 4.428571429 | 0.81 |
| 1207 | 0 | US | H01L | H01L0033 | 20 | 17 | 32 | 1 | 32.00 |
| 1208 | 0 | JP | C30B | C30B0029 | 11 | 23 | 4 | 1.333333333 | 0.50 |
| 1209 | 0 | JP | H01L | H01L0033 | 14 | 18 | 6 | 3.375 | 0.22 |
| 1210 | 0 | JP | C01B | C01B0031 | 5 | 4 | 1 | 1.166666667 | 0.14 |
| 1211 | 0 | JP | H01L | H01L0021 | 19 | 42 | 4 | 1.166666667 | 0.57 |
| 1212 | 0 | JP | H01L | H01L0021 | 14 | 40 | 0 | 4.5 | 0.00 |
| 1213 | 0 | JP | H01L | H01L0021 | 11 | 3 | 1 | 1.25 | 0.20 |
| 1214 | 0 | US | G01J | G01J0003 | 20 | 7 | 10 | 1 | 10.00 |
| 1215 | 0 | US | C09K | C09K0011 | 10 | 5 | 2 | 1 | 2.00 |
| 1216 | 0 | JP | H01L | H01L0033 | 10 | 5 | 0 | 2 | 0.00 |
| 1217 | 0 | US | H01L | H01L0033 | 104 | 93 | 93 | 8.8 | 1.06 |
| 1218 | 0 | JP | C30B | C30B0025 | 15 | 6 | 3 | 1.6 | 0.38 |
| 1219 | 0 | US | H01L | H01L0021 | 46 | 92 | 42 | 8.8 | 0.48 |
| 1220 | 0 | JP | C23C | C23C0016 | 16 | 21 | 0 | 2.4 | 0.00 |
| 1221 | 0 | US | H01L | H01L0033 | 46 | 20 | 21 | 1 | 21.00 |
| 1222 | 0 | US | H01L | H01L0033 | 19 | 44 | 12 | 5.7 | 0.21 |
| 1223 | 0 | FR | C30B | C30B0025 | 19 | 11 | 14 | 2.333333333 | 0.50 |
| 1224 | 0 | US | H01L | H01L0021 | 22 | 42 | 0 | 1.8 | 0.00 |
| 1225 | 0 | US | H01L | H01L0033 | 20 | 8 | 45 | 1 | 45.00 |
| 1226 | 0 | JP | H01L | H01L0033 | 16 | 6 | 15 | 4 | 1.25 |
| 1227 | 0 | JP | H01L | H01L0021 | 51 | 22 | 5 | 1.833333333 | 0.45 |
| 1228 | 0 | US | H01L | H01L0021 | 29 | 55 | 15 | 1.8 | 0.83 |
| 1229 | 0 | JP | H01L | H01L0033 | 3 | 39 | 9 | 5 | 0.45 |
| 1230 | 0 | JP | H01L | H01L0021 | 22 | 41 | 11 | 2.5 | 0.73 |
| 1231 | 0 | US | H01L | H01L0033 | 20 | 24 | 0 | 1 | 0.00 |
| 1232 | 0 | JP | H01L | H01L0033 | 50 | 17 | 4 | 1.333333333 | 0.50 |
| 1233 | 0 | US | H01L | H01L0025 | 4 | 23 | 35 | 2.375 | 1.84 |
| 1234 | 0 | US | H01L | H01L0023 | 18 | 11 | 11 | 1 | 11.00 |
| 1235 | 0 | US | F21V | F21V0008 | 20 | 2 | 1 | 1 | 1.00 |
| 1236 | 0 | US | H01L | H01L0033 | 21 | 27 | 19 | 3.090909091 | 0.56 |
| 1237 | 0 | JP | H01L | H01L0033 | 4 | 16 | 45 | 4.333333333 | 1.73 |
| 1238 | 0 | JP | H01L | H01L0021 | 12 | 136 | 4 | 1.285714286 | 0.44 |
| 1239 | 0 | CA | H01L | H01L0021 | 19 | 7 | 5 | 1.666666667 | 1.00 |
| 1240 | 0 | JP | H01L | H01L0033 | 17 | 7 | 1 | 1.666666667 | 0.20 |
| 1241 | 0 | JP | H01L | H01L0033 | 48 | 45 | 6 | 3.7 | 0.16 |
| 1242 | 0 | JP | H01L | H01L0021 | 35 | 17 | 2 | 1.25 | 0.20 |
| 1243 | 0 | JP | C30B | C30B0029 | 3 | 10 | 8 | 1.428571429 | 0.80 |
| 1244 | 0 | JP | H01L | H01L0021 | 13 | 150 | 2 | 1.2 | 0.33 |
| 1245 | 0 | CA | H01L | H01L0033 | 36 | 80 | 16 | 5.285714286 | 0.43 |
| 1246 | 0 | JP | H01J | H01J0001 | 13 | 34 | 1 | 2.5 | 0.07 |
| 1247 | 0 | JP | H01L | H01L0033 | 9 | 20 | 20 | 2 | 2.00 |
| 1248 | 0 | CA | H01S | H01S0005 | 16 | 10 | 1 | 1.5 | 0.17 |
| 1249 | 0 | JP | H01L | H01L0033 | 20 | 70 | 12 | 4.777777778 | 0.28 |
| 1250 | 0 | US | H01L | H01L0033 | 53 | 76 | 21 | 8.8 | 0.24 |
| 1251 | 0 | JP | C30B | C30B0029 | 22 | 146 | 3 | 1.2 | 0.50 |
| 1252 | 0 | JP | H01L | H01L0021 | 2 | 1 | 0 | 1.285714286 | 0.00 |
| 1253 | 0 | JP | H01L | H01L0033 | 8 | 5 | 9 | 2.333333333 | 0.64 |
| 1254 | 0 | JP | C23C | C23C0016 | 12 | 7 | 0 | 1 | 0.00 |
| 1255 | 0 | JP | C30B | C30B0029 | 24 | 124 | 1 | 1.666666667 | 0.20 |
| 1256 | 0 | JP | H01L | H01L0033 | 20 | 20 | 3 | 1.285714286 | 0.33 |
| 1257 | 0 | JP | H01L | H01L0021 | 30 | 136 | 5 | 1.875 | 0.33 |
| 1258 | 0 | JP | C23C | C23C0016 | 6 | 20 | 3 | 2.4 | 0.25 |
| 1259 | 0 | JP | H01L | H01L0033 | 26 | 24 | 2 | 1.333333333 | 0.50 |
| 1260 | 0 | JP | C30B | C30B0025 | 14 | 7 | 2 | 1.666666667 | 0.40 |
| 1261 | 0 | JP | C30B | C30B0025 | 10 | 10 | 0 | 1.333333333 | 0.00 |
| 1262 | 0 | US | H01H | H01H0059 | 19 | 16 | 88 | 1 | 88.00 |
| 1263 | 0 | US | H01J | H01J0001 | 41 | 65 | 7 | 1 | 3.50 |
| 1264 | 0 | JP | C30B | C30B0025 | 10 | 10 | 2 | 1.333333333 | 0.50 |
| 1265 | 1 | US | H01L | H01L0025 | 39 | 24 | 10 | 2 | 0.50 |
| 1266 | 1 | JP | H01L | H01L0033 | 27 | 38 | 22 | 3.75 | 0.73 |
| 1267 | 0 | CA | H01L | H01L0033 | 32 | 13 | 18 | 1.6 | 2.25 |
| 1268 | 0 | JP | H01L | H01L0033 | 11 | 19 | 1 | 3.666666667 | 0.09 |
| 1269 | 0 | JP | B24B | B24B0037 | 11 | 14 | 3 | 2.5 | 0.20 |
| 1270 | 0 | JP | C23C | C23C0016 | 4 | 10 | 1 | 1.25 | 0.20 |
| 1271 | 0 | NL | G03F | G03F0001 | 16 | 16 | 9 | 1.5 | 1.00 |
| 1272 | 0 | CA | H01L | H01L0033 | 25 | 10 | 16 | 1.333333333 | 4.00 |
| 1273 | 0 | JP | C30B | C30B0025 | 15 | 156 | 2 | 2 | 0.33 |
| 1274 | 0 | CA | H01L | H01L0033 | 35 | 62 | 27 | 5.285714286 | 0.73 |
| 1275 | 0 | US | H01L | H01L0033 | 21 | 10 | 12 | 1.222222222 | 1.09 |
| 1276 | 0 | US | H01L | H01L0021 | 35 | 44 | 5 | 8.8 | 0.06 |
| 1277 | 0 | CA | H01L | H01L0021 | 14 | 15 | 1 | 1.75 | 0.14 |
| 1278 | 0 | JP | H01L | H01L0033 | 20 | 43 | 1 | 1.25 | 0.10 |
| 1279 | 0 | JP | H01L | H01L0033 | 21 | 16 | 17 | 1.5 | 1.89 |
| 1280 | 0 | CA | H01L | H01L0033 | 26 | 11 | 6 | 2 | 1.00 |
| 1281 | 0 | JP | H01L | H01L0033 | 10 | 15 | 1 | 1.142857143 | 0.13 |
| 1282 | 0 | CA | H01L | H01L0033 | 36 | 58 | 11 | 5.285714286 | 0.30 |
| 1283 | 0 | JP | G03F | G03F0007 | 1 | 9 | 0 | 2.6 | 0.00 |
| 1284 | 0 | US | H01L | H01L0033 | 39 | 47 | 16 | 5.7 | 0.28 |
| 1285 | 0 | US | H01L | H01L0021 | 40 | 7 | 88 | 1 | 88.00 |
| 1286 | 0 | CA | H01L | H01L0033 | 7 | 29 | 12 | 4.25 | 0.71 |
| 1287 | 0 | CA | H01L | H01L0033 | 13 | 25 | 6 | 3.25 | 0.46 |
| 1288 | 0 | JP | H01L | H01L0033 | 19 | 10 | 6 | 2.2 | 0.27 |
| 1289 | 0 | JP | C30B | C30B0025 | 14 | 23 | 0 | 2.166666667 | 0.00 |
| 1290 | 0 | US | H01S | H01S0005 | 18 | 13 | 33 | 1 | 33.00 |
| 1291 | 0 | JP | H01L | H01L0021 | 37 | 10 | 18 | 1.5 | 2.00 |
| 1292 | 0 | JP | H01L | H01L0021 | 4 | 59 | 13 | 4 | 0.46 |
| 1293 | 0 | JP | H01L | H01L0033 | 16 | 14 | 23 | 1.25 | 4.60 |
| 1294 | 0 | JP | H01L | H01L0031 | 4 | 7 | 0 | 1.6 | 0.00 |
| 1295 | 0 | CA | H01L | H01L0033 | 21 | 9 | 5 | 1.333333333 | 1.25 |
| 1296 | 0 | JP | C30B | C30B0025 | 14 | 32 | 7 | 4.2 | 0.33 |
| 1297 | 0 | JP | C23C | C23C0016 | 11 | 90 | 3 | 4.25 | 0.09 |
| 1298 | 0 | US | C30B | C30B0033 | 119 | 33 | 5 | 4.428571429 | 0.16 |
| 1299 | 0 | FR | C23C | C23C0014 | 13 | 16 | 2 | 2.333333333 | 0.29 |
| 1300 | 0 | DE | H01L | H01L0033 | 60 | 32 | 37 | 4.181818182 | 0.80 |
| 1301 | 0 | CA | H01L | H01L0033 | 22 | 8 | 5 | 1.333333333 | 1.25 |
| 1302 | 0 | JP | C30B | C30B0029 | 17 | 14 | 0 | 1.4 | 0.00 |
| 1303 | 0 | US | H01L | H01L0033 | 23 | 21 | 11 | 3.090909091 | 0.32 |
| 1304 | 0 | JP | H01L | H01L0033 | 13 | 13 | 34 | 2.2 | 1.55 |
| 1305 | 0 | US | H01L | H01L0021 | 8 | 3 | 10 | 1 | 10.00 |
| 1306 | 0 | JP | F21K | F21K0099 | 26 | 8 | 38 | 3.666666667 | 1.73 |
| 1307 | 0 | US | C30B | C30B0025 | 91 | 15 | 13 | 2.285714286 | 0.81 |
| 1308 | 0 | CA | H01L | H01L0033 | 16 | 24 | 8 | 1.75 | 1.14 |
| 1309 | 0 | US | H01L | H01L0021 | 40 | 48 | 6 | 1.8 | 0.33 |
| 1310 | 0 | JP | H01L | H01L0033 | 22 | 23 | 10 | 1.333333333 | 2.50 |
| 1311 | 0 | CA | H01S | H01S0005 | 25 | 19 | 8 | 1.333333333 | 2.00 |
| 1312 | 0 | US | H01L | H01L0033 | 52 | 83 | 17 | 5.7 | 0.30 |
| 1313 | 0 | JP | B29C | B29C0067 | 9 | 32 | 13 | 3.444444444 | 0.42 |
| 1314 | 0 | US | H01L | H01L0023 | 16 | 14 | 7 | 1 | 7.00 |
| 1315 | 0 | TW | B05D | B05D0005 | 11 | 1 | 0 | 2 | 0.00 |
| 1316 | 0 | JP | H01L | H01L0021 | 24 | 146 | 2 | 1.714285714 | 0.17 |
| 1317 | 0 | JP | B24B | B24B0037 | 6 | 12 | 1 | 1.2 | 0.17 |
| 1318 | 0 | JP | C30B | C30B0015 | 5 | 14 | 8 | 3 | 0.44 |
| 1319 | 0 | JP | H01S | H01S0005 | 13 | 23 | 4 | 1.5 | 0.44 |
| 1320 | 0 | JP | C30B | C30B0029 | 18 | 150 | 3 | 1.714285714 | 0.25 |
| 1321 | 0 | JP | H01L | H01L0031 | 4 | 27 | 1 | 3.5 | 0.07 |
| 1322 | 0 | JP | H01L | H01L0021 | 5 | 18 | 1 | 2.6 | 0.08 |
| 1323 | 0 | JP | H01L | H01L0029 | 17 | 12 | 2 | 1.666666667 | 0.40 |
| 1324 | 0 | JP | B32B | B32B0009 | 5 | 25 | 2 | 3 | 0.22 |
| 1325 | 0 | CA | H01L | H01L0029 | 15 | 15 | 10 | 1.666666667 | 2.00 |
| 1326 | 0 | JP | H01L | H01L0021 | 6 | 2 | 1 | 1.4 | 0.14 |
| 1327 | 0 | CA | H01L | H01L0029 | 14 | 16 | 6 | 1.25 | 1.20 |
| 1328 | 0 | US | H01L | H01L0031 | 34 | 25 | 0 | 2.285714286 | 0.00 |
| 1329 | 0 | US | H01L | H01L0051 | 9 | 4 | 51 | 1 | 51.00 |
| 1330 | 0 | JP | H01L | H01L0021 | 23 | 5 | 1 | 1.4 | 0.14 |
| 1331 | 0 | JP | H01L | H01L0027 | 5 | 80 | 6 | 7.714285714 | 0.11 |
| 1332 | 0 | JP | H01L | H01L0021 | 5 | 5 | 0 | 2.4 | 0.00 |
| 1333 | 0 | JP | H01L | H01L0029 | 21 | 31 | 8 | 1.333333333 | 1.00 |
| 1334 | 0 | JP | H01L | H01L0021 | 30 | 3 | 10 | 2.333333333 | 0.71 |
| 1335 | 0 | JP | H01L | H01L0033 | 17 | 11 | 0 | 1 | 0.00 |
| 1336 | 0 | JP | C30B | C30B0025 | 40 | 7 | 0 | 1.6 | 0.00 |
| 1337 | 0 | JP | H01L | H01L0029 | 46 | 25 | 8 | 1.6 | 1.00 |
| 1338 | 0 | JP | H01L | H01L0029 | 5 | 22 | 4 | 3.375 | 0.15 |
| 1339 | 0 | CA | H01L | H01L0021 | 13 | 18 | 4 | 5.285714286 | 0.11 |
| 1340 | 0 | US | F28D | F28D0015 | 51 | 19 | 46 | 1 | 46.00 |
| 1341 | 0 | JP | H01L | H01L0027 | 20 | 7 | 0 | 1.5 | 0.00 |
| 1342 | 0 | CA | H01L | H01L0029 | 18 | 14 | 7 | 1.333333333 | 1.75 |
| 1343 | 0 | US | C30B | C30B0025 | 14 | 35 | 85 | 1 | 85.00 |
| 1344 | 0 | JP | H01L | H01L0027 | 20 | 23 | 0 | 1.333333333 | 0.00 |
| 1345 | 0 | JP | H01L | H01L0021 | 8 | 6 | 2 | 1.75 | 0.14 |
| 1346 | 0 | US | H01L | H01L0029 | 6 | 47 | 3 | 1.3 | 0.23 |
| 1347 | 0 | US | H01L | H01L0033 | 21 | 15 | 7 | 1 | 7.00 |
| 1348 | 0 | JP | H01L | H01L0027 | 14 | 7 | 0 | 1.5 | 0.00 |
| 1349 | 0 | JP | F21K | F21K0007 | 12 | 12 | 7 | 3.666666667 | 0.32 |
| 1350 | 0 | CA | C09K | C09K0011 | 9 | 19 | 7 | 1.75 | 1.00 |
| 1351 | 0 | JP | H01L | H01L0027 | 6 | 14 | 7 | 2.2 | 0.32 |
| 1352 | 0 | CA | G02B | G02B0001 | 14 | 84 | 17 | 2.8 | 1.21 |
| 1353 | 0 | JP | H01L | H01L0029 | 16 | 23 | 17 | 2 | 1.42 |
| 1354 | 0 | CA | G02B | G02B0001 | 64 | 74 | 14 | 2.8 | 1.00 |
| 1355 | 0 | US | H01L | H01L0031 | 11 | 49 | 2 | 5.7 | 0.04 |
| 1356 | 0 | CA | G02B | G02B0005 | 22 | 29 | 7 | 1.333333333 | 1.75 |
| 1357 | 0 | JP | C30B | C30B0025 | 47 | 96 | 12 | 8.235294118 | 0.09 |
| 1358 | 0 | US | H01J | H01J0009 | 15 | 15 | 10 | 1.6 | 1.25 |
| 1359 | 0 | JP | C30B | C30B0025 | 23 | 87 | 1 | 4.25 | 0.03 |
| 1360 | 0 | US | H01L | H01L0033 | 31 | 10 | 10 | 1 | 10.00 |
| 1361 | 0 | JP | C30B | C30B0025 | 92 | 26 | 3 | 9.25 | 0.04 |
| 1362 | 0 | US | H01L | H01L0021 | 23 | 23 | 1 | 1.333333333 | 0.25 |
| 1363 | 0 | JP | H01L | H01L0021 | 12 | 24 | 6 | 2.5 | 0.30 |
| 1364 | 0 | US | C30B | C30B0011 | 5 | 8 | 18 | 1 | 18.00 |
| 1365 | 0 | JP | H01J | H01J0001 | 63 | 49 | 9 | 2.2 | 0.82 |
| 1366 | 0 | JP | C30B | C30B0029 | 7 | 8 | 2 | 1.25 | 0.40 |
| 1367 | 0 | JP | H01L | H01L0033 | 2 | 36 | 2 | 2.25 | 0.22 |
| 1368 | 0 | JP | H01L | H01L0031 | 16 | 83 | 15 | 3.166666667 | 0.79 |
| 1369 | 0 | DE | H01L | H01L0029 | 13 | 17 | 1 | 2 | 0.08 |
| 1370 | 0 | JP | H01L | H01L0033 | 29 | 8 | 6 | 9.25 | 0.08 |
| 1371 | 0 | US | H01L | H01L0031 | 17 | 26 | 7 | 2.875 | 0.30 |
| 1372 | 0 | JP | H01L | H01L0033 | 30 | 7 | 9 | 9.25 | 0.12 |
| 1373 | 0 | JP | C30B | C30B0023 | 18 | 9 | 0 | 1.5 | 0.00 |
| 1374 | 0 | US | C30B | C30B0033 | 20 | 10 | 28 | 1 | 28.00 |
| 1375 | 0 | DE | H01L | H01L0033 | 14 | 160 | 0 | 1 | 0.00 |
| 1376 | 0 | JP | H01L | H01L0033 | 36 | 105 | 3 | 3.7 | 0.08 |
| 1377 | 0 | JP | H01L | H01L0029 | 1 | 7 | 1 | 4 | 0.04 |
| 1378 | 0 | JP | H01L | H01L0029 | 1 | 7 | 0 | 4 | 0.00 |
| 1379 | 0 | JP | H01L | H01L0029 | 1 | 7 | 0 | 4 | 0.00 |
| 1380 | 0 | US | H01L | H01L0027 | 20 | 3 | 5 | 1.333333333 | 1.25 |
| 1381 | 0 | KR | C23C | C23C0016 | 6 | 1 | 0 | 1.6 | 0.00 |
| 1382 | 0 | JP | H01L | H01L0031 | 8 | 18 | 0 | 1.5 | 0.00 |
| 1383 | 0 | US | H01L | H01L0021 | 24 | 35 | 2 | 3.090909091 | 0.06 |
| 1384 | 0 | JP | C23F | C23F0001 | 20 | 21 | 5 | 1.333333333 | 0.63 |
| 1385 | 0 | JP | H01L | H01L0029 | 1 | 7 | 1 | 4 | 0.04 |
| 1386 | 0 | JP | H01L | H01L0029 | 1 | 7 | 2 | 4 | 0.07 |
| 1387 | 0 | JP | H01L | H01L0033 | 25 | 3 | 1 | 1.333333333 | 0.13 |
| 1388 | 0 | JP | H01L | H01L0029 | 4 | 6 | 6 | 2.333333333 | 0.43 |
| 1389 | 0 | JP | H01L | H01L0029 | 16 | 29 | 1 | 4.333333333 | 0.04 |
| 1390 | 0 | US | H01L | H01L0021 | 21 | 23 | 0 | 2.285714286 | 0.00 |
| 1391 | 0 | DE | H01L | H01L0021 | 12 | 44 | 2 | 2 | 0.17 |
| 1392 | 0 | DE | H01L | H01L0027 | 13 | 50 | 2 | 4.181818182 | 0.04 |
| 1393 | 0 | JP | H01L | H01L0033 | 20 | 19 | 2 | 4.333333333 | 0.08 |
| 1394 | 0 | JP | H01L | H01L0033 | 20 | 21 | 0 | 4.333333333 | 0.00 |
| 1395 | 0 | JP | H01L | H01L0021 | 10 | 152 | 1 | 1.857142857 | 0.08 |
| 1396 | 0 | JP | H01L | H01L0027 | 20 | 9 | 3 | 1.5 | 0.50 |
| 1397 | 0 | JP | H01L | H01L0021 | 21 | 95 | 0 | 4.25 | 0.00 |
| 1398 | 0 | JP | C30B | C30B0029 | 3 | 7 | 1 | 1.375 | 0.09 |
| 1399 | 0 | DE | C09K | C09K0011 | 27 | 56 | 9 | 4.181818182 | 0.20 |
| 1400 | 0 | JP | H01L | H01L0021 | 11 | 24 | 0 | 2.75 | 0.00 |
| 1401 | 0 | JP | H01L | H01L0033 | 26 | 35 | 0 | 2.25 | 0.00 |
| 1402 | 0 | JP | H01L | H01L0027 | 9 | 106 | 2 | 4.666666667 | 0.07 |
| 1403 | 0 | JP | H01L | H01L0029 | 15 | 66 | 0 | 4.666666667 | 0.00 |
| 1404 | 0 | JP | H01J | H01J0001 | 7 | 21 | 0 | 1 | 0.00 |
| 1405 | 0 | FR | H01L | H01L0021 | 4 | 16 | 1 | 2.333333333 | 0.14 |
| 1406 | 0 | JP | H01L | H01L0029 | 17 | 24 | 2 | 2.5 | 0.10 |
| 1407 | 0 | JP | H01L | H01L0033 | 11 | 51 | 7 | 2.2 | 0.64 |
| 1408 | 0 | JP | H01L | H01L0021 | 9 | 31 | 5 | 2.833333333 | 0.29 |
| 1409 | 0 | JP | H01L | H01L0021 | 1 | 19 | 1 | 4.333333333 | 0.04 |
| 1410 | 0 | KR | H01L | H01L0029 | 21 | 5 | 2 | 2 | 0.20 |
| 1411 | 0 | JP | G03C | G03C0001 | 10 | 33 | 3 | 6.833333333 | 0.07 |
| 1412 | 0 | US | H01L | H01L0027 | 15 | 5 | 2 | 1.5 | 0.22 |
| 1413 | 0 | JP | H01L | H01L0029 | 20 | 7 | 9 | 1.166666667 | 1.29 |
| 1414 | 0 | JP | H01L | H01L0029 | 20 | 125 | 11 | 9.625 | 0.14 |
| 1415 | 0 | JP | C09K | C09K0011 | 9 | 3 | 5 | 4.181818182 | 0.11 |
| 1416 | 0 | US | C30B | C30B0023 | 13 | 38 | 46 | 1 | 15.33 |
| 1417 | 0 | JP | H01L | H01L0033 | 10 | 76 | 15 | 4.333333333 | 0.38 |
| 1418 | 0 | JP | C09K | C09K0011 | 22 | 11 | 4 | 2.5 | 0.40 |
| 1419 | 0 | JP | H01L | H01L0029 | 16 | 26 | 0 | 1.833333333 | 0.00 |
| 1420 | 0 | KR | H01L | H01L0021 | 14 | 1 | 0 | 1.8 | 0.00 |
| 1421 | 0 | US | B60Q | B60Q0001 | 18 | 15 | 4 | 1.333333333 | 1.00 |
| 1422 | 0 | JP | H01L | H01L0021 | 22 | 11 | 3 | 1.375 | 0.27 |
| 1423 | 0 | DE | H01L | H01L0021 | 6 | 9 | 0 | 1.166666667 | 0.00 |
| 1424 | 0 | JP | H01L | H01L0021 | 15 | 90 | 1 | 7.714285714 | 0.02 |
| 1425 | 0 | US | H01L | H01L0021 | 24 | 11 | 20 | 1 | 20.00 |
| 1426 | 0 | DE | H01L | H01L0033 | 13 | 11 | 1 | 1.75 | 0.14 |
| 1427 | 0 | JP | H01L | H01L0031 | 7 | 84 | 4 | 4.777777778 | 0.09 |
| 1428 | 0 | US | H01L | H01L0021 | 14 | 10 | 1 | 1 | 1.00 |
| 1429 | 0 | JP | C30B | C30B0025 | 42 | 12 | 1 | 2.333333333 | 0.14 |
| 1430 | 0 | US | H01L | H01L0021 | 4 | 8 | 4 | 1 | 4.00 |
| 1431 | 0 | US | H01L | H01L0021 | 12 | 28 | 30 | 1 | 30.00 |
| 1432 | 0 | US | H01L | H01L0021 | 21 | 136 | 5 | 9.625 | 0.06 |
| 1433 | 0 | JP | H01L | H01L0033 | 20 | 71 | 3 | 4 | 0.11 |
| 1434 | 0 | JP | H01L | H01L0029 | 8 | 15 | 0 | 4.333333333 | 0.00 |
| 1435 | 0 | FR | C30B | C30B0025 | 28 | 37 | 2 | 1.714285714 | 0.17 |
| 1436 | 0 | JP | C30B | C30B0015 | 8 | 10 | 0 | 1.166666667 | 0.00 |
| 1437 | 0 | JP | H01L | H01L0021 | 6 | 17 | 2 | 2.6 | 0.15 |
| 1438 | 0 | JP | H01L | H01L0029 | 12 | 64 | 1 | 1.285714286 | 0.11 |
| 1439 | 0 | JP | H01L | H01L0027 | 17 | 4 | 1 | 1.666666667 | 0.20 |
| 1440 | 0 | JP | B32B | B32B0009 | 8 | 13 | 0 | 1.333333333 | 0.00 |
| 1441 | 0 | US | H01L | H01L0033 | 23 | 62 | 15 | 3.666666667 | 0.45 |
| 1442 | 0 | JP | H01J | H01J0001 | 7 | 52 | 6 | 2.2 | 0.55 |
| 1443 | 0 | JP | C23C | C23C0016 | 27 | 68 | 0 | 1.428571429 | 0.00 |
| 1444 | 0 | JP | H01L | H01L0033 | 7 | 17 | 3 | 3.666666667 | 0.14 |
| 1445 | 0 | KR | H01C | H01C0007 | 13 | 30 | 16 | 4.333333333 | 0.62 |
| 1446 | 0 | US | C09K | C09K0011 | 33 | 17 | 28 | 1.333333333 | 7.00 |
| 1447 | 0 | JP | C01G | C01G0015 | 20 | 124 | 8 | 8.235294118 | 0.06 |
| 1448 | 0 | JP | C30B | C30B0029 | 12 | 24 | 1 | 1.333333333 | 0.13 |
| 1449 | 0 | US | H01L | H01L0023 | 40 | 15 | 7 | 3 | 0.78 |
| 1450 | 0 | JP | C09K | C09K0011 | 6 | 23 | 9 | 3.6 | 0.25 |
| 1451 | 0 | US | H01L | H01L0021 | 17 | 53 | 4 | 8.8 | 0.05 |
| 1452 | 0 | JP | H01L | H01L0027 | 13 | 64 | 1 | 4.181818182 | 0.02 |
| 1453 | 0 | JP | H01L | H01L0031 | 9 | 20 | 8 | 3.666666667 | 0.36 |
| 1454 | 0 | US | F21V | F21V0029 | 24 | 52 | 22 | 3.666666667 | 0.67 |
| 1455 | 0 | US | H01J | H01J0009 | 13 | 30 | 7 | 4.25 | 0.41 |
| 1456 | 0 | JP | H01L | H01L0021 | 30 | 12 | 2 | 2 | 0.25 |
| 1457 | 0 | US | H01L | H01L0027 | 29 | 11 | 7 | 1.666666667 | 1.40 |
| 1458 | 0 | JP | H01L | H01L0021 | 31 | 15 | 0 | 2 | 0.00 |
| 1459 | 0 | US | H01L | H01L0021 | 37 | 77 | 2 | 2.8 | 0.14 |
| 1460 | 0 | JP | H01L | H01L0033 | 10 | 27 | 3 | 1.5 | 0.33 |
| 1461 | 0 | US | H01L | H01L0027 | 23 | 9 | 2 | 1.333333333 | 0.50 |
| 1462 | 0 | KR | H01L | H01L0029 | 22 | 19 | 4 | 1.4 | 0.57 |
| 1463 | 0 | JP | H01L | H01L0029 | 42 | 16 | 10 | 1.333333333 | 2.50 |
| 1464 | 0 | US | H01L | H01L0023 | 21 | 2 | 1 | 1 | 1.00 |
| 1465 | 0 | JP | H01L | H01L0029 | 22 | 24 | 0 | 1.333333333 | 0.00 |
| 1466 | 0 | SG | H01L | H01L0029 | 36 | 20 | 0 | 1.5 | 0.00 |
| 1467 | 0 | US | H01L | H01L0029 | 5 | 35 | 0 | 2.285714286 | 0.00 |
| 1468 | 0 | US | H01L | H01L0029 | 13 | 93 | 7 | 2 | 1.17 |
| 1469 | 0 | JP | H01L | H01L0029 | 23 | 8 | 3 | 1.6 | 0.38 |
| 1470 | 0 | JP | C09K | C09K0011 | 36 | 22 | 6 | 3.6 | 0.17 |
| 1471 | 0 | JP | H01J | H01J0001 | 14 | 40 | 3 | 2.2 | 0.27 |
| 1472 | 0 | US | H01L | H01L0029 | 21 | 85 | 1 | 5.7 | 0.02 |
| 1473 | 0 | US | B32B | B32B0009 | 50 | 30 | 2 | 1.75 | 0.14 |
| 1474 | 0 | JP | H01L | H01L0021 | 36 | 7 | 4 | 2.333333333 | 0.29 |
| 1475 | 0 | US | H01L | H01L0029 | 18 | 53 | 5 | 1.333333333 | 0.63 |
| 1476 | 0 | US | H01L | H01L0021 | 47 | 48 | 7 | 2.142857143 | 0.47 |
| 1477 | 0 | US | H01L | H01L0027 | 26 | 36 | 4 | 2 | 0.40 |
| 1478 | 0 | US | H01L | H01L0021 | 11 | 46 | 2 | 1.4 | 0.29 |
| 1479 | 0 | JP | H01S | H01S0005 | 19 | 19 | 1 | 1.5 | 0.17 |
| 1480 | 0 | US | B23K | B23K0001 | 11 | 71 | 0 | 8.8 | 0.00 |
| 1481 | 0 | JP | H01J | H01J0001 | 15 | 71 | 8 | 3.444444444 | 0.26 |
| 1482 | 0 | US | H01L | H01L0033 | 11 | 26 | 2 | 3.25 | 0.15 |
| 1483 | 0 | US | H01L | H01L0033 | 15 | 30 | 0 | 1.75 | 0.00 |
| 1484 | 0 | DE | H01L | H01L0021 | 41 | 183 | 0 | 1 | 0.00 |
| 1485 | 0 | KR | H01L | H01L0021 | 10 | 5 | 0 | 1.6 | 0.00 |
| 1486 | 0 | JP | H01L | H01L0033 | 4 | 32 | 1 | 4.333333333 | 0.04 |
| 1487 | 0 | US | H01L | H01L0029 | 30 | 47 | 17 | 2 | 1.21 |
| 1488 | 0 | JP | H01S | H01S0003 | 19 | 3 | 3 | 2.75 | 0.27 |
| 1489 | 0 | JP | C30B | C30B0023 | 8 | 59 | 2 | 2.25 | 0.11 |
| 1490 | 0 | JP | H01L | H01L0033 | 25 | 49 | 3 | 2 | 0.25 |
| 1491 | 0 | US | F21V | F21V0019 | 6 | 3 | 8 | 2 | 0.80 |
| 1492 | 0 | JP | H01L | H01L0021 | 13 | 53 | 0 | 5 | 0.00 |
| 1493 | 0 | JP | H01L | H01L0031 | 15 | 17 | 0 | 1.8 | 0.00 |
| 1494 | 0 | JP | H01L | H01L0029 | 9 | 26 | 1 | 1.166666667 | 0.14 |
| 1495 | 0 | JP | G02B | G02B0006 | 4 | 36 | 0 | 1 | 0.00 |
| 1496 | 0 | JP | H01L | H01L0029 | 15 | 81 | 2 | 7.714285714 | 0.04 |
| 1497 | 0 | US | G09G | G09G0003 | 5 | 142 | 1 | 2.5 | 0.03 |
| 1498 | 0 | JP | C30B | C30B0029 | 34 | 21 | 1 | 1.8 | 0.11 |
| 1499 | 0 | US | B32B | B32B0009 | 35 | 16 | 2 | 2.142857143 | 0.13 |
| 1500 | 0 | JP | H01L | H01L0021 | 16 | 102 | 11 | 3.166666667 | 0.58 |
| 1501 | 0 | US | C30B | C30B0007 | 30 | 18 | 148 | 1 | 148.00 |
| 1502 | 0 | US | C30B | C30B0025 | 12 | 13 | 5 | 1 | 5.00 |
| 1503 | 0 | KR | H01L | H01L0027 | 18 | 259 | 0 | 1 | 0.00 |
| 1504 | 0 | JP | H01L | H01L0027 | 40 | 98 | 0 | 1 | 0.00 |
| 1505 | 0 | JP | H01L | H01L0033 | 19 | 92 | 2 | 4.333333333 | 0.05 |
| 1506 | 0 | US | H01L | H01L0033 | 16 | 15 | 4 | 2.666666667 | 0.50 |
| 1507 | 0 | DE | C01G | C01G0017 | 32 | 29 | 1 | 1.166666667 | 0.14 |
| 1508 | 0 | JP | H01J | H01J0063 | 3 | 16 | 1 | 1.285714286 | 0.11 |
| 1509 | 0 | US | H01L | H01L0021 | 23 | 3 | 4 | 1 | 4.00 |
| 1510 | 0 | US | H01L | H01L0029 | 77 | 20 | 0 | 1.75 | 0.00 |
| 1511 | 0 | US | G02F | G02F0001 | 37 | 45 | 2 | 1.666666667 | 0.40 |
| 1512 | 0 | JP | C30B | C30B0029 | 7 | 19 | 6 | 2.8 | 0.21 |
| 1513 | 0 | JP | H01L | H01L0029 | 6 | 6 | 1 | 2.6 | 0.08 |
| 1514 | 0 | JP | C30B | C30B0025 | 2 | 9 | 2 | 2.5 | 0.13 |
| 1515 | 0 | US | H01L | H01L0021 | 37 | 77 | 8 | 3 | 0.53 |
| 1516 | 0 | JP | C01B | C01B0021 | 26 | 136 | 9 | 8.235294118 | 0.06 |
| 1517 | 0 | DE | H01J | H01J0001 | 19 | 32 | 0 | 1.5 | 0.00 |
| 1518 | 0 | JP | C01G | C01G0015 | 19 | 171 | 8 | 8.235294118 | 0.06 |
| 1519 | 0 | KR | F21V | F21V0001 | 11 | 9 | 2 | 1.4 | 0.29 |
| 1520 | 0 | US | H01L | H01L0021 | 18 | 138 | 1 | 9.625 | 0.01 |
| 1521 | 0 | FR | H01L | H01L0021 | 14 | 5 | 0 | 1.2 | 0.00 |
| 1522 | 0 | JP | H01L | H01L0021 | 7 | 12 | 0 | 1.2 | 0.00 |
| 1523 | 0 | JP | G02F | G02F0002 | 26 | 6 | 1 | 2.25 | 0.11 |
| 1524 | 0 | JP | H01L | H01L0029 | 24 | 26 | 6 | 1.333333333 | 1.50 |
| 1525 | 0 | JP | C30B | C30B0025 | 5 | 26 | 0 | 1.666666667 | 0.00 |
| 1526 | 0 | JP | C30B | C30B0025 | 8 | 7 | 2 | 2 | 0.33 |
| 1527 | 0 | JP | H01L | H01L0021 | 18 | 103 | 5 | 4.25 | 0.15 |
| 1528 | 0 | US | C25D | C25D0005 | 16 | 7 | 3 | 1 | 3.00 |
| 1529 | 0 | US | H01L | H01L0033 | 28 | 89 | 5 | 1.333333333 | 1.25 |
| 1530 | 0 | US | H01L | H01L0033 | 24 | 94 | 1 | 2.333333333 | 0.14 |
| 1531 | 0 | JP | H01L | H01L0021 | 8 | 4 | 1 | 2.6 | 0.08 |
| 1532 | 0 | DE | H01L | H01L0033 | 19 | 24 | 4 | 1.2 | 0.67 |
| 1533 | 0 | JP | C30B | C30B0011 | 5 | 10 | 0 | 1.2 | 0.00 |
| 1534 | 0 | TW | C09K | C09K0011 | 17 | 1 | 0 | 1 | 0.00 |
| 1535 | 0 | DE | H01L | H01L0051 | 35 | 14 | 0 | 2 | 0.00 |
| 1536 | 0 | JP | H01L | H01L0021 | 14 | 24 | 0 | 1.714285714 | 0.00 |
| 1537 | 0 | US | H01L | H01L0021 | 35 | 7 | 1 | 1 | 1.00 |
| 1538 | 0 | JP | F21V | F21V0007 | 11 | 18 | 4 | 1.571428571 | 0.36 |
| 1539 | 0 | US | H01L | H01L0023 | 18 | 54 | 16 | 2.6 | 1.23 |
| 1540 | 0 | US | H01L | H01L0021 | 23 | 17 | 3 | 3 | 0.20 |
| 1541 | 0 | JP | H01L | H01L0029 | 17 | 159 | 0 | 1.714285714 | 0.00 |
| 1542 | 0 | KR | H01L | H01L0033 | 12 | 24 | 0 | 1.4 | 0.00 |
| 1543 | 0 | JP | B32B | B32B0017 | 8 | 15 | 0 | 1.8 | 0.00 |
| 1544 | 0 | US | H05B | H05B0033 | 22 | 22 | 106 | 1 | 106.00 |
| 1545 | 0 | JP | H01L | H01L0021 | 9 | 23 | 1 | 1.75 | 0.07 |
| 1546 | 0 | JP | H01L | H01L0029 | 20 | 20 | 6 | 1.5 | 1.00 |
| 1547 | 0 | US | H01L | H01L0033 | 20 | 47 | 0 | 3.090909091 | 0.00 |
| 1548 | 0 | SG | H01L | H01L0033 | 15 | 22 | 0 | 1.25 | 0.00 |
| 1549 | 0 | JP | H01L | H01L0033 | 6 | 17 | 1 | 1.142857143 | 0.13 |
| 1550 | 0 | US | H01L | H01L0021 | 31 | 9 | 20 | 1 | 20.00 |
| 1551 | 0 | JP | H01J | H01J0001 | 15 | 23 | 0 | 1.25 | 0.00 |
| 1552 | 0 | JP | H01L | H01L0025 | 20 | 20 | 0 | 1.833333333 | 0.00 |
| 1553 | 0 | KR | H01L | H01L0021 | 22 | 17 | 0 | 2 | 0.00 |
| 1554 | 0 | US | H01L | H01L0021 | 32 | 16 | 0 | 3 | 0.00 |
| 1555 | 0 | JP | H01L | H01L0027 | 15 | 11 | 0 | 1.285714286 | 0.00 |
| 1556 | 0 | JP | H01L | H01L0021 | 11 | 147 | 0 | 1.875 | 0.00 |
| 1557 | 0 | JP | F21V | F21V0029 | 22 | 69 | 0 | 3.714285714 | 0.00 |
| 1558 | 0 | US | H01L | H01L0021 | 10 | 10 | 1 | 1.6 | 0.13 |
| 1559 | 0 | US | H01L | H01L0033 | 20 | 11 | 28 | 1 | 28.00 |
| 1560 | 0 | JP | H01L | H01L0027 | 11 | 25 | 1 | 1.2 | 0.17 |
| 1561 | 0 | JP | H01L | H01L0033 | 17 | 4 | 0 | 4 | 0.00 |
| 1562 | 0 | JP | H01L | H01L0033 | 14 | 24 | 1 | 2.25 | 0.11 |
| 1563 | 0 | US | H01L | H01L0021 | 12 | 141 | 2 | 9.625 | 0.03 |
| 1564 | 0 | JP | C30B | C30B0025 | 16 | 61 | 0 | 2.25 | 0.00 |
| 1565 | 0 | TW | C09K | C09K0011 | 7 | 0 | 0 | 1 | 0.00 |
| 1566 | 0 | JP | H01L | H01L0033 | 16 | 9 | 0 | 1.6 | 0.00 |
| 1567 | 0 | DE | H01L | H01L0021 | 20 | 15 | 0 | 1.75 | 0.00 |
| 1568 | 0 | KR | H01L | H01L0033 | 8 | 2 | 0 | 1.25 | 0.00 |
| 1569 | 0 | JP | H01L | H01L0021 | 22 | 44 | 13 | 2.714285714 | 0.68 |
| 1570 | 0 | US | H01L | H01L0021 | 11 | 14 | 20 | 1 | 20.00 |
| 1571 | 0 | US | H01L | H01L0021 | 6 | 145 | 93 | 8.111111111 | 1.27 |
| 1572 | 0 | KR | H01L | H01L0027 | 18 | 2 | 1 | 1.2 | 0.17 |
| 1573 | 0 | JP | H01J | H01J0001 | 12 | 18 | 3 | 2.4 | 0.25 |
| 1574 | 0 | SE | H01L | H01L0021 | 14 | 25 | 1 | 1.666666667 | 0.20 |
| 1575 | 0 | KR | H01L | H01L0029 | 4 | 25 | 0 | 1.2 | 0.00 |
| 1576 | 0 | US | C30B | C30B0025 | 16 | 2 | 1 | 1 | 1.00 |
| 1577 | 0 | JP | C09K | C09K0011 | 7 | 76 | 0 | 3.444444444 | 0.00 |
| 1578 | 0 | JP | H01L | H01L0033 | 5 | 27 | 0 | 1.8 | 0.00 |
| 1579 | 0 | JP | H01L | H01L0027 | 15 | 6 | 0 | 1.4 | 0.00 |
| 1580 | 0 | JP | C09K | C09K0011 | 40 | 49 | 0 | 3.6 | 0.00 |
| 1581 | 0 | JP | H01L | H01L0027 | 10 | 23 | 1 | 1.375 | 0.09 |
| 1582 | 0 | JP | H01L | H01L0033 | 4 | 25 | 0 | 1.333333333 | 0.00 |
| 1583 | 0 | US | H01L | H01L0033 | 32 | 263 | 11 | 4.285714286 | 0.37 |
| 1584 | 0 | JP | H01L | H01L0033 | 7 | 61 | 0 | 2.2 | 0.00 |
| 1585 | 0 | US | H01L | H01L0021 | 12 | 23 | 2 | 1.2 | 0.33 |
| 1586 | 0 | JP | H01L | H01L0021 | 9 | 39 | 0 | 4.2 | 0.00 |
| 1587 | 0 | JP | C30B | C30B0007 | 18 | 259 | 0 | 8.235294118 | 0.00 |
| 1588 | 0 | JP | H01L | H01L0033 | 4 | 11 | 0 | 2.75 | 0.00 |
| 1589 | 0 | KR | H01L | H01L0033 | 3 | 8 | 0 | 1.375 | 0.00 |
| 1590 | 0 | JP | C09K | C09K0011 | 25 | 49 | 0 | 3.6 | 0.00 |
| 1591 | 0 | US | H01L | H01L0033 | 26 | 5 | 0 | 1.166666667 | 0.00 |
| 1592 | 0 | JP | H01L | H01L0021 | 5 | 33 | 1 | 2.6 | 0.08 |
| 1593 | 0 | JP | H01L | H01L0027 | 10 | 25 | 1 | 2 | 0.13 |
| 1594 | 0 | JP | H01L | H01L0021 | 2 | 26 | 1 | 3 | 0.06 |
| 1595 | 0 | US | H01L | H01L0029 | 22 | 61 | 0 | 8.8 | 0.00 |
| 1596 | 0 | US | H01L | H01L0021 | 13 | 11 | 0 | 1.6 | 0.00 |
| 1597 | 0 | JP | H01L | H01L0029 | 20 | 12 | 1 | 1.333333333 | 0.25 |
| 1598 | 0 | JP | H01L | H01L0033 | 20 | 103 | 0 | 4.777777778 | 0.00 |
| 1599 | 0 | SG | H01L | H01L0033 | 7 | 16 | 0 | 1.25 | 0.00 |
| 1600 | 0 | US | H01L | H01L0031 | 25 | 14 | 0 | 1.333333333 | 0.00 |
| 1601 | 0 | JP | H01J | H01J0001 | 6 | 20 | 0 | 1.285714286 | 0.00 |
| 1602 | 0 | JP | H01L | H01L0033 | 13 | 15 | 0 | 1.6 | 0.00 |
| 1603 | 0 | JP | H01L | H01L0029 | 12 | 124 | 0 | 3.7 | 0.00 |
| 1604 | 0 | US | H01L | H01L0033 | 31 | 18 | 28 | 1 | 28.00 |
| 1605 | 0 | US | B01D | B01D0009 | 66 | 70 | 3 | 2.636363636 | 0.10 |
| 1606 | 0 | US | C09K | C09K0011 | 43 | 15 | 35 | 1 | 35.00 |
| 1607 | 0 | CN | H01L | H01L0021 | 20 | 3 | 0 | 1.166666667 | 0.00 |
| 1608 | 0 | KR | H01L | H01L0021 | 9 | 19 | 1 | 3 | 0.07 |
| 1609 | 0 | KR | H01L | H01L0033 | 28 | 57 | 0 | 1 | 0.00 |
| 1610 | 0 | US | F21V | F21V0029 | 17 | 83 | 2 | 3.666666667 | 0.06 |
| 1611 | 0 | JP | H01L | H01L0021 | 20 | 9 | 1 | 1.333333333 | 0.13 |
| 1612 | 0 | US | H01L | H01L0021 | 7 | 22 | 0 | 1.75 | 0.00 |
| 1613 | 0 | US | C09K | C09K0011 | 8 | 19 | 0 | 1.333333333 | 0.00 |
| 1614 | 0 | US | H01L | H01L0031 | 10 | 47 | 0 | 2.285714286 | 0.00 |
| 1615 | 0 | JP | C01G | C01G0015 | 4 | 25 | 0 | 1.8 | 0.00 |
| 1616 | 0 | US | H01L | H01L0033 | 14 | 97 | 0 | 2.333333333 | 0.00 |
| 1617 | 0 | JP | H01L | H01L0033 | 14 | 111 | 0 | 4.181818182 | 0.00 |
| 1618 | 0 | KR | H01L | H01L0021 | 13 | 76 | 1 | 3 | 0.08 |
| 1619 | 0 | JP | H01L | H01L0027 | 11 | 16 | 0 | 1.428571429 | 0.00 |
| 1620 | 0 | US | H01L | H01L0033 | 56 | 100 | 0 | 5.7 | 0.00 |
| 1621 | 0 | US | H01L | H01L0033 | 29 | 69 | 0 | 5.7 | 0.00 |
| 1622 | 0 | US | F21S | F21S0004 | 22 | 363 | 40 | 1 | 40.00 |
| 1623 | 0 | US | B32B | B32B0018 | 20 | 23 | 0 | 2.142857143 | 0.00 |
| 1624 | 0 | JP | H01L | H01L0033 | 12 | 34 | 1 | 1.166666667 | 0.14 |
| 1625 | 0 | KR | H01L | H01L0033 | 14 | 9 | 1 | 1.8 | 0.11 |
| 1626 | 0 | US | H01L | H01L0029 | 30 | 63 | 0 | 9.625 | 0.00 |
| 1627 | 0 | KR | H01L | H01L0021 | 14 | 5 | 0 | 1.5 | 0.00 |
| 1628 | 0 | US | H01L | H01L0033 | 14 | 67 | 0 | 1.5 | 0.00 |
| 1629 | 0 | KR | C30B | C30B0025 | 6 | 19 | 1 | 1.4 | 0.14 |
| 1630 | 0 | KR | H01L | H01L0029 | 14 | 22 | 0 | 2 | 0.00 |
| 1631 | 0 | JP | C30B | C30B0028 | 24 | 50 | 0 | 2.625 | 0.00 |
| 1632 | 0 | JP | H01L | H01L0021 | 5 | 21 | 1 | 1.333333333 | 0.13 |
| 1633 | 0 | JP | H01L | H01L0027 | 9 | 26 | 0 | 1.75 | 0.00 |
| 1634 | 0 | KR | H01L | H01L0033 | 18 | 4 | 1 | 1.4 | 0.14 |
| 1635 | 0 | JP | F21V | F21V0009 | 18 | 48 | 2 | 1.8 | 0.22 |
| 1636 | 0 | JP | H01L | H01L0021 | 9 | 6 | 0 | 1.333333333 | 0.00 |
| 1637 | 0 | US | H01L | H01L0033 | 12 | 14 | 0 | 2 | 0.00 |
| 1638 | 0 | JP | H01L | H01L0021 | 6 | 8 | 0 | 1.333333333 | 0.00 |
| 1639 | 0 | KR | H01L | H01L0033 | 11 | 65 | 1 | 2.5 | 0.07 |
| 1640 | 0 | JP | H01L | H01L0033 | 12 | 17 | 0 | 1.166666667 | 0.00 |
| 1641 | 0 | JP | H01L | H01L0033 | 13 | 36 | 0 | 1.166666667 | 0.00 |
| 1642 | 0 | JP | H01L | H01L0027 | 6 | 14 | 0 | 1.333333333 | 0.00 |
| 1643 | 0 | JP | C23C | C23C0016 | 14 | 12 | 1 | 1.5 | 0.11 |
| 1644 | 0 | US | F21K | F21K0007 | 18 | 17 | 61 | 1 | 61.00 |
| 1645 | 0 | KR | H01L | H01L0027 | 11 | 14 | 0 | 1.2 | 0.00 |
| 1646 | 0 | JP | H01L | H01L0021 | 13 | 16 | 0 | 1.5 | 0.00 |
| 1647 | 0 | JP | H01L | H01L0031 | 23 | 24 | 1 | 2.142857143 | 0.07 |
| 1648 | 0 | JP | C01G | C01G0015 | 14 | 249 | 0 | 8.235294118 | 0.00 |
| 1649 | 0 | JP | H01L | H01L0021 | 5 | 12 | 0 | 1.5 | 0.00 |
| 1650 | 0 | JP | H01L | H01L0029 | 4 | 24 | 0 | 1.333333333 | 0.00 |
| 1651 | 0 | JP | H01L | H01L0029 | 22 | 126 | 0 | 3.7 | 0.00 |
| 1652 | 0 | JP | H01J | H01J0001 | 18 | 17 | 0 | 1.25 | 0.00 |
| 1653 | 0 | JP | C30B | C30B0025 | 27 | 23 | 0 | 1.75 | 0.00 |
| 1654 | 0 | JP | H01L | H01L0021 | 13 | 26 | 0 | 1.6 | 0.00 |
| 1655 | 0 | JP | H01L | H01L0029 | 33 | 8 | 0 | 1.75 | 0.00 |
| 1656 | 0 | US | C09K | C09K0011 | 43 | 24 | 41 | 1 | 41.00 |
| 1657 | 0 | JP | H01L | H01L0029 | 1 | 21 | 0 | 1.285714286 | 0.00 |
| 1658 | 0 | JP | H01L | H01L0033 | 2 | 22 | 0 | 1.333333333 | 0.00 |
| 1659 | 0 | KR | H01L | H01L0021 | 17 | 20 | 0 | 1.8 | 0.00 |
| 1660 | 0 | US | H01L | H01L0029 | 36 | 2 | 43 | 1 | 43.00 |
| 1661 | 0 | JP | B32B | B32B0009 | 6 | 18 | 0 | 1.333333333 | 0.00 |
| 1662 | 0 | JP | H01L | H01L0031 | 5 | 15 | 0 | 1.333333333 | 0.00 |
| 1663 | 0 | KR | H01L | H01L0033 | 2 | 15 | 1 | 2.142857143 | 0.07 |
| 1664 | 0 | KR | H01L | H01L0033 | 6 | 19 | 0 | 2.142857143 | 0.00 |
| 1665 | 0 | US | F21V | F21V0003 | 20 | 161 | 1 | 3.666666667 | 0.03 |
| 1666 | 0 | HK | H01R | H01R0033 | 8 | 0 | 0 | 1 | 0.00 |
| 1667 | 0 | JP | H01L | H01L0033 | 6 | 17 | 0 | 1.333333333 | 0.00 |
| 1668 | 0 | KR | H01L | H01L0021 | 20 | 68 | 1 | 3 | 0.08 |
| 1669 | 0 | JP | H01L | H01L0029 | 10 | 26 | 0 | 1.2 | 0.00 |
| 1670 | 0 | US | H01L | H01L0023 | 20 | 9 | 41 | 1 | 41.00 |
| 1671 | 0 | JP | H01L | H01L0033 | 5 | 12 | 1 | 1.25 | 0.20 |
| 1672 | 0 | US | H01L | H01L0029 | 42 | 83 | 1 | 2.142857143 | 0.07 |
| 1673 | 0 | JP | H01L | H01L0029 | 18 | 5 | 0 | 1.25 | 0.00 |
| 1674 | 0 | JP | C30B | C30B0023 | 18 | 28 | 0 | 2.4 | 0.00 |
| 1675 | 0 | JP | B32B | B32B0009 | 20 | 19 | 0 | 1.666666667 | 0.00 |
| 1676 | 0 | KR | H01L | H01L0029 | 10 | 14 | 0 | 1.2 | 0.00 |
| 1677 | 1 | US | H01L | H01L0027 | 47 | 71 | 0 | 2.25 | 0.00 |
| 1678 | 0 | US | H01L | H01L0023 | 5 | 36 | 0 | 1.2 | 0.00 |
| 1679 | 0 | US | H01L | H01L0021 | 14 | 136 | 143 | 1 | 143.00 |
| 1680 | 0 | JP | H01L | H01L0033 | 4 | 24 | 1 | 1.666666667 | 0.10 |
| 1681 | 0 | US | H01L | H01L0027 | 16 | 53 | 0 | 1.142857143 | 0.00 |
| 1682 | 0 | US | H01L | H01L0031 | 21 | 100 | 0 | 1.333333333 | 0.00 |
| 1683 | 0 | KR | H01L | H01L0029 | 12 | 18 | 0 | 1.8 | 0.00 |
| 1684 | 0 | JP | H01L | H01L0033 | 11 | 20 | 0 | 1.833333333 | 0.00 |
| 1685 | 0 | JP | H01L | H01L0021 | 8 | 14 | 0 | 1.2 | 0.00 |
| 1686 | 0 | US | H01L | H01L0033 | 24 | 30 | 42 | 1 | 42.00 |
| 1687 | 0 | US | B81B | B81B0007 | 63 | 20 | 26 | 1 | 26.00 |
| 1688 | 0 | US | H01L | H01L0033 | 21 | 123 | 3 | 4.285714286 | 0.10 |
| 1689 | 0 | JP | H01L | H01L0021 | 24 | 111 | 0 | 3.714285714 | 0.00 |
| 1690 | 0 | US | H01L | H01L0033 | 39 | 28 | 40 | 1 | 40.00 |
| 1691 | 0 | US | H01L | H01L0021 | 16 | 10 | 0 | 1.142857143 | 0.00 |
| 1692 | 0 | JP | H01L | H01L0023 | 4 | 14 | 0 | 1.2 | 0.00 |
| 1693 | 0 | JP | B32B | B32B0009 | 6 | 23 | 1 | 2 | 0.17 |
| 1694 | 0 | JP | H01L | H01L0021 | 2 | 31 | 1 | 2.428571429 | 0.06 |
| 1695 | 0 | JP | H01L | H01L0029 | 13 | 18 | 0 | 1.333333333 | 0.00 |
| 1696 | 0 | JP | B32B | B32B0027 | 12 | 53 | 0 | 1.5 | 0.00 |
| 1697 | 0 | DE | H01L | H01L0029 | 14 | 59 | 0 | 1.25 | 0.00 |
| 1698 | 0 | US | G02B | G02B0006 | 24 | 19 | 53 | 1 | 53.00 |
| 1699 | 0 | GB | H01L | H01L0021 | 18 | 17 | 0 | 1.2 | 0.00 |
| 1700 | 0 | KR | H01L | H01L0029 | 14 | 35 | 0 | 2 | 0.00 |
| 1701 | 0 | US | H01L | H01L0029 | 9 | 153 | 0 | 9.625 | 0.00 |
| 1702 | 0 | JP | H01L | H01L0021 | 15 | 22 | 0 | 1.2 | 0.00 |
| 1703 | 0 | US | H01L | H01L0023 | 19 | 68 | 0 | 1.375 | 0.00 |
| 1704 | 0 | JP | H01L | H01L0033 | 15 | 23 | 0 | 1.25 | 0.00 |
| 1705 | 0 | JP | H01L | H01L0021 | 14 | 33 | 0 | 1.6 | 0.00 |
| 1706 | 0 | US | H01L | H01L0033 | 18 | 133 | 0 | 2 | 0.00 |
| 1707 | 0 | US | H01L | H01L0033 | 20 | 30 | 0 | 1.5 | 0.00 |
| 1708 | 0 | JP | H01L | H01L0033 | 20 | 18 | 0 | 3.666666667 | 0.00 |
| 1709 | 0 | JP | H01L | H01L0031 | 17 | 40 | 0 | 2.2 | 0.00 |
| 1710 | 0 | JP | H01L | H01L0021 | 8 | 17 | 0 | 1.2 | 0.00 |
| 1711 | 0 | KR | H01L | H01L0033 | 31 | 14 | 0 | 1.8 | 0.00 |
| 1712 | 0 | JP | G02B | G02B0006 | 8 | 12 | 0 | 1.25 | 0.00 |
| 1713 | 0 | KR | H01L | H01L0021 | 3 | 24 | 0 | 2.666666667 | 0.00 |
| 1714 | 0 | JP | H01L | H01L0021 | 12 | 333 | 0 | 8.235294118 | 0.00 |
| 1715 | 0 | JP | H01L | H01L0033 | 2 | 7 | 0 | 1.166666667 | 0.00 |
| 1716 | 0 | JP | H01L | H01L0021 | 20 | 38 | 0 | 1.333333333 | 0.00 |
| 1717 | 0 | JP | H01L | H01L0029 | 22 | 23 | 0 | 1.142857143 | 0.00 |
| 1718 | 0 | JP | H01L | H01L0029 | 9 | 42 | 0 | 1.857142857 | 0.00 |
| 1719 | 0 | JP | H01S | H01S0005 | 21 | 17 | 0 | 1.5 | 0.00 |
| 1720 | 0 | US | H01L | H01L0021 | 19 | 17 | 0 | 3 | 0.00 |
| 1721 | 0 | DE | H01L | H01L0021 | 16 | 28 | 0 | 1.5 | 0.00 |
| 1722 | 0 | JP | H01J | H01J0017 | 7 | 28 | 0 | 2.333333333 | 0.00 |
| 1723 | 0 | US | C30B | C30B0025 | 26 | 62 | 0 | 1.75 | 0.00 |
| 1724 | 0 | KR | H01L | H01L0033 | 11 | 28 | 0 | 2.142857143 | 0.00 |
| 1725 | 0 | JP | H01L | H01L0029 | 16 | 15 | 0 | 1.75 | 0.00 |
| 1726 | 0 | US | C30B | C30B0025 | 8 | 643 | 5 | 1 | 2.50 |
| 1727 | 0 | KR | H01L | H01L0033 | 10 | 29 | 0 | 1.4 | 0.00 |
| 1728 | 0 | JP | H01L | H01L0029 | 11 | 21 | 0 | 1.5 | 0.00 |
| 1729 | 0 | US | H01L | H01L0033 | 22 | 52 | 1 | 1.75 | 0.14 |
| 1730 | 0 | JP | H01L | H01L0033 | 11 | 25 | 0 | 0.5 | 0.00 |
| 1731 | 0 | US | H01L | H01L0033 | 18 | 104 | 0 | 3 | 0.00 |
| 1732 | 0 | KR | H01L | H01L0027 | 1 | 7 | 0 | 1.375 | 0.00 |
| 1733 | 0 | JP | H01L | H01L0033 | 8 | 12 | 0 | 1.166666667 | 0.00 |
| 1734 | 0 | JP | C09K | C09K0011 | 8 | 18 | 0 | 2 | 0.00 |
| 1735 | 0 | JP | H01L | H01L0021 | 15 | 60 | 0 | 1.2 | 0.00 |
| 1736 | 0 | JP | H01L | H01L0027 | 8 | 9 | 0 | 1.666666667 | 0.00 |
| 1737 | 0 | GB | H01L | H01L0029 | 13 | 13 | 0 | 1.142857143 | 0.00 |
| 1738 | 0 | KR | H01L | H01L0029 | 13 | 6 | 0 | 1.166666667 | 0.00 |
| 1739 | 0 | JP | H01L | H01L0033 | 4 | 14 | 0 | 1.5 | 0.00 |
| 1740 | 0 | JP | H01L | H01L0031 | 6 | 33 | 0 | 3.375 | 0.00 |
| 1741 | 0 | JP | H01L | H01L0021 | 6 | 20 | 0 | 1.5 | 0.00 |
| 1742 | 0 | JP | F21V | F21V0009 | 10 | 6 | 0 | 1.333333333 | 0.00 |
| 1743 | 0 | JP | H01L | H01L0029 | 19 | 14 | 0 | 1.333333333 | 0.00 |
| 1744 | 0 | KR | H01L | H01L0029 | 24 | 27 | 0 | 2.333333333 | 0.00 |
| 1745 | 0 | KR | H01L | H01L0029 | 8 | 13 | 0 | 1.25 | 0.00 |
| 1746 | 0 | KR | H01L | H01L0021 | 5 | 47 | 0 | 3 | 0.00 |
| 1747 | 0 | JP | H01L | H01L0033 | 10 | 16 | 0 | 1.166666667 | 0.00 |
| 1748 | 0 | JP | H01L | H01L0033 | 34 | 8 | 0 | 1.333333333 | 0.00 |
| 1749 | 0 | US | H01J | H01J0009 | 5 | 8 | 0 | 1 | 0.00 |
| 1750 | 0 | JP | H01L | H01L0033 | 14 | 53 | 27 | 1.75 | 3.86 |
| 1751 | 0 | JP | H01L | H01L0031 | 19 | 22 | 0 | 2 | 0.00 |
| 1752 | 0 | JP | H01L | H01L0027 | 13 | 23 | 0 | 2.25 | 0.00 |
| 1753 | 0 | JP | C09K | C09K0011 | 48 | 14 | 0 | 1 | 0.00 |
| 1754 | 0 | US | H01L | H01L0029 | 31 | 53 | 0 | 1.3 | 0.00 |
| 1755 | 0 | US | C09K | C09K0011 | 26 | 29 | 84 | 1.166666667 | 12.00 |
| 1756 | 0 | US | H01L | H01L0033 | 17 | 20 | 53 | 1 | 53.00 |
| 1757 | 0 | US | H01L | H01L0033 | 22 | 16 | 28 | 1 | 28.00 |
| 1758 | 0 | US | H01L | H01L0027 | 11 | 737 | 14 | 1 | 14.00 |
| 1759 | 0 | US | H01L | H01L0033 | 17 | 10 | 34 | 1 | 34.00 |
| 1760 | 0 | US | H01L | H01L0033 | 19 | 27 | 4 | 1 | 4.00 |
| 1761 | 0 | US | C30B | C30B0025 | 18 | 3 | 10 | 1 | 10.00 |
| 1762 | 0 | US | G01S | G01S0007 | 30 | 27 | 0 | 1 | 0.00 |
| 1763 | 0 | US | H01L | H01L0021 | 29 | 738 | 0 | 8.111111111 | 0.00 |
| 1764 | 0 | US | C30B | C30B0025 | 22 | 669 | 3 | 1 | 0.43 |
| 1765 | 0 | US | C30B | C30B0023 | 28 | 1 | 0 | 1 | 0.00 |
| 1766 | 0 | JP | H01L | H01L0021 | 7 | 5 | 2 | 1 | 1.00 |
| 1767 | 0 | US | H01S | H01S0003 | 13 | 22 | 1 | 1 | 1.00 |
| 1768 | 0 | US | C30B | C30B0023 | 7 | 3 | 4 | 1 | 4.00 |
| 1769 | 0 | JP | G03B | G03B0027 | 6 | 5 | 22 | 1 | 11.00 |
| 1770 | 0 | US | H01L | H01L0033 | 13 | 2 | 0 | 1 | 0.00 |
| 1771 | 0 | US | H01L | H01L0023 | 20 | 1 | 2 | 1 | 2.00 |
| 1772 | 0 | US | H01L | H01L0033 | 8 | 19 | 0 | 2 | 0.00 |
| 1773 | 0 | US | H01L | H01L0021 | 18 | 1 | 0 | 1 | 0.00 |
| 1774 | 0 | US | C23C | C23C0016 | 28 | 60 | 8 | 3 | 2.67 |
| 1775 | 0 | US | H01L | H01L0021 | 147 | 303 | 25 | 2.285714286 | 1.56 |
| 1776 | 0 | US | C09K | C09K0011 | 12 | 30 | 13 | 1.166666667 | 1.86 |
| 1777 | 0 | US | H01L | H01L0033 | 12 | 41 | 13 | 1 | 13.00 |
| 1778 | 0 | US | H01L | H01L0033 | 14 | 20 | 12 | 1 | 12.00 |
| 1779 | 0 | JP | H01J | H01J0009 | 10 | 1 | 20 | 1.2 | 3.33 |
| 1780 | 0 | CH | H01L | H01L0033 | 20 | 8 | 59 | 1 | 29.50 |
| 1781 | 0 | US | C08G | C08G0059 | 13 | 48 | 2 | 1.333333333 | 0.25 |
| 1782 | 0 | US | H01L | H01L0021 | 14 | 16 | 4 | 1.5 | 1.33 |
| 1783 | 0 | US | H05B | H05B0033 | 31 | 91 | 0 | 1.333333333 | 0.00 |
| 1784 | 0 | DE | H01L | H01L0029 | 23 | 3 | 24 | 1 | 12.00 |
| 1785 | 0 | US | H01L | H01L0021 | 25 | 20 | 6 | 1 | 6.00 |
| 1786 | 0 | US | H01L | H01L0021 | 41 | 17 | 2 | 1 | 2.00 |
| 1787 | 0 | DE | H01L | H01L0031 | 7 | 7 | 11 | 1 | 5.50 |
| 1788 | 0 | US | H01L | H01L0033 | 6 | 7 | 1 | 1.111111111 | 0.10 |
| 1789 | 0 | JP | C30B | C30B0019 | 3 | 7 | 4 | 1.2 | 0.67 |
| 1790 | 0 | US | C08G | C08G0073 | 12 | 50 | 0 | 1 | 0.00 |
| 1791 | 0 | US | H01L | H01L0021 | 37 | 13 | 3 | 3 | 0.11 |
| 1792 | 0 | US | H01L | H01L0023 | 23 | 19 | 0 | 1 | 0.00 |
| 1793 | 0 | US | F21V | F21V0007 | 56 | 153 | 7 | 1.166666667 | 1.00 |
| 1794 | 0 | US | F21V | F21V0009 | 14 | 17 | 12 | 1 | 12.00 |
| 1795 | 0 | DE | H01L | H01L0021 | 2 | 1 | 1 | 1.166666667 | 0.14 |
| 1796 | 0 | US | H01J | H01J0001 | 29 | 8 | 9 | 1 | 9.00 |
| 1797 | 0 | US | H01L | H01L0029 | 13 | 20 | 5 | 1 | 5.00 |
| 1798 | 1 | US | A61B | A61B0006 | 29 | 95 | 186 | 23.28571429 | 0.57 |
| 1799 | 0 | US | H01L | H01L0021 | 32 | 20 | 8 | 1.166666667 | 1.14 |
| 1800 | 0 | JP | H01L | H01L0033 | 2 | 2 | 2 | 1 | 0.29 |
| 1801 | 0 | JP | H01L | H01L0021 | 7 | 7 | 4 | 1 | 0.57 |
| 1802 | 0 | US | F21V | F21V0029 | 42 | 54 | 14 | 1.8 | 1.56 |
| 1803 | 0 | US | H01L | H01L0029 | 18 | 56 | 2 | 3 | 0.07 |
| 1804 | 0 | JP | H01L | H01L0033 | 4 | 9 | 5 | 1.142857143 | 0.63 |
| 1805 | 0 | JP | H01J | H01J0001 | 4 | 2 | 4 | 1 | 0.57 |
| 1806 | 0 | US | C09K | C09K0011 | 22 | 18 | 3 | 1.5 | 1.00 |
| 1807 | 0 | JP | B60C | B60C0009 | 5 | 6 | 18 | 1 | 9.00 |
| 1808 | 0 | DE | H01L | H01L0033 | 5 | 10 | 33 | 1 | 4.71 |
| 1809 | 0 | DE | H01L | H01L0021 | 7 | 8 | 9 | 1 | 0.82 |
| 1810 | 0 | US | C09K | C09K0011 | 32 | 28 | 3 | 1 | 3.00 |
| 1811 | 0 | US | F21V | F21V0007 | 21 | 29 | 9 | 1 | 1.80 |
| 1812 | 0 | US | H01L | H01L0021 | 23 | 12 | 11 | 1 | 11.00 |
| 1813 | 0 | CH | H01L | H01L0033 | 12 | 12 | 9 | 1 | 1.29 |
| 1814 | 0 | US | H01L | H01L0021 | 7 | 1 | 1 | 1 | 0.20 |
| 1815 | 0 | JP | C30B | C30B0029 | 8 | 18 | 15 | 1 | 2.50 |
| 1816 | 0 | US | H01L | H01L0033 | 9 | 50 | 11 | 1 | 1.83 |
| 1817 | 0 | US | H01L | H01L0021 | 16 | 119 | 1 | 1.666666667 | 0.07 |
| 1818 | 0 | JP | H01L | H01L0033 | 10 | 6 | 8 | 1 | 4.00 |
| 1819 | 0 | US | F21V | F21V0029 | 25 | 49 | 7 | 1 | 1.17 |
| 1820 | 0 | US | H01L | H01L0029 | 10 | 14 | 1 | 1 | 0.25 |
| 1821 | 0 | JP | H01L | H01L0033 | 9 | 8 | 5 | 1.142857143 | 0.63 |
| 1822 | 0 | JP | H01L | H01L0021 | 5 | 8 | 6 | 1 | 1.50 |
| 1823 | 0 | US | H01J | H01J0001 | 16 | 80 | 1 | 1 | 0.17 |
| 1824 | 0 | SU | H01L | H01L0021 | 3 | 5 | 9 | 1.181818182 | 0.69 |
| 1825 | 0 | JP | C30B | C30B0019 | 6 | 2 | 10 | 1 | 2.50 |
| 1826 | 0 | JP | H01L | H01L0033 | 18 | 21 | 12 | 1.4 | 1.71 |
| 1827 | 0 | JP | H01L | H01L0021 | 14 | 7 | 18 | 1 | 9.00 |
| 1828 | 0 | JP | H01L | H01L0033 | 6 | 2 | 13 | 1.142857143 | 1.63 |
| 1829 | 0 | JP | H01L | H01L0021 | 8 | 9 | 12 | 1 | 2.40 |
| 1830 | 0 | US | H01L | H01L0029 | 22 | 13 | 2 | 1 | 2.00 |
| 1831 | 0 | FR | H01L | H01L0033 | 5 | 2 | 1 | 1 | 0.20 |
| 1832 | 0 | US | H01L | H01L0029 | 21 | 27 | 0 | 1 | 0.00 |
| 1833 | 0 | US | B24B | B24B0001 | 32 | 43 | 0 | 1 | 0.00 |
| 1834 | 0 | US | C09K | C09K0011 | 12 | 143 | 2 | 1 | 0.40 |
| 1835 | 0 | US | H01L | H01L0023 | 23 | 31 | 1 | 1.75 | 0.14 |
| 1836 | 0 | CH | H01L | H01L0033 | 8 | 7 | 10 | 1 | 2.50 |
| 1837 | 0 | DE | H01L | H01L0021 | 1 | 5 | 0 | 1.166666667 | 0.00 |
| 1838 | 0 | DE | G01D | G01D0007 | 13 | 9 | 17 | 1 | 5.67 |
| 1839 | 0 | DE | H01L | H01L0033 | 13 | 5 | 16 | 1 | 2.67 |
| 1840 | 0 | US | H01L | H01L0027 | 16 | 6 | 0 | 1.333333333 | 0.00 |
| 1841 | 0 | JP | G09F | G09F0009 | 8 | 9 | 10 | 1 | 2.50 |
| 1842 | 0 | JP | C30B | C30B0019 | 4 | 4 | 17 | 1.166666667 | 2.43 |
| 1843 | 0 | JP | H01L | H01L0033 | 5 | 2 | 14 | 1 | 2.33 |
| 1844 | 0 | JP | H01L | H01L0021 | 3 | 3 | 24 | 1 | 12.00 |
| 1845 | 0 | JP | F16B | F16B0005 | 7 | 7 | 11 | 2 | 2.75 |
| 1846 | 0 | JP | H01S | H01S0005 | 14 | 9 | 20 | 1.5 | 2.22 |
| 1847 | 0 | JP | H01L | H01L0033 | 10 | 1 | 7 | 1 | 1.17 |
| 1848 | 0 | DE | H01L | H01L0021 | 2 | 14 | 34 | 1 | 11.33 |
| 1849 | 0 | US | F21V | F21V0009 | 35 | 25 | 0 | 5 | 0.00 |
| 1850 | 0 | JP | H01S | H01S0005 | 11 | 5 | 14 | 1 | 2.33 |
| 1851 | 0 | JP | H01L | H01L0033 | 11 | 4 | 18 | 1.25 | 3.60 |
| 1852 | 0 | SU | H01L | H01L0033 | 4 | 9 | 57 | 1 | 6.33 |
| 1853 | 0 | US | C30B | C30B0029 | 16 | 11 | 7 | 1 | 1.17 |
| 1854 | 0 | US | F21V | F21V0007 | 17 | 25 | 1 | 5 | 0.20 |
| 1855 | 0 | DE | H01L | H01L0033 | 3 | 2 | 14 | 1 | 2.33 |
| 1856 | 0 | JP | C30B | C30B0019 | 5 | 6 | 5 | 1 | 1.67 |
| 1857 | 0 | JP | H01L | H01L0021 | 4 | 16 | 6 | 1.142857143 | 0.75 |
| 1858 | 0 | JP | H01L | H01L0021 | 11 | 5 | 2 | 1 | 0.29 |
| 1859 | 0 | US | H01L | H01L0021 | 6 | 141 | 1 | 1.666666667 | 0.20 |
| 1860 | 0 | US | H01L | H01L0021 | 74 | 231 | 11 | 1 | 11.00 |
| 1861 | 0 | JP | H01L | H01L0021 | 6 | 8 | 18 | 1 | 4.50 |
| 1862 | 0 | US | H01J | H01J0001 | 12 | 71 | 7 | 1 | 7.00 |
| 1863 | 0 | CH | H01L | H01L0029 | 8 | 6 | 13 | 1 | 4.33 |
| 1864 | 0 | US | H01L | H01L0051 | 18 | 210 | 4 | 4.916666667 | 0.07 |
| 1865 | 0 | US | H01L | H01L0031 | 11 | 8 | 15 | 1.142857143 | 1.88 |
| 1866 | 0 | US | F21V | F21V0029 | 28 | 385 | 1 | 1.142857143 | 0.13 |
| 1867 | 0 | US | H01L | H01L0029 | 57 | 20 | 0 | 1.166666667 | 0.00 |
| 1868 | 0 | US | F21V | F21V0009 | 6 | 49 | 2 | 1 | 2.00 |
| 1869 | 0 | JP | G02B | G02B0006 | 10 | 3 | 13 | 1 | 2.17 |
| 1870 | 0 | JP | H01L | H01L0033 | 8 | 9 | 8 | 1.2 | 1.33 |
| 1871 | 0 | US | F21V | F21V0005 | 45 | 10 | 3 | 1 | 3.00 |
| 1872 | 0 | JP | H01L | H01L0033 | 15 | 2 | 8 | 1.666666667 | 1.60 |
| 1873 | 1 | US | F21V | F21V0005 | 38 | 111 | 11 | 1 | 1.22 |
| 1874 | 0 | JP | H01L | H01L0021 | 10 | 6 | 14 | 1 | 7.00 |
| 1875 | 0 | JP | G09F | G09F0009 | 5 | 3 | 12 | 1 | 4.00 |
| 1876 | 0 | US | H01L | H01L0033 | 12 | 3 | 8 | 1.142857143 | 1.00 |
| 1877 | 0 | US | H01L | H01L0031 | 23 | 13 | 4 | 1 | 4.00 |
| 1878 | 1 | JP | C30B | C30B0025 | 8 | 11 | 9 | 1 | 1.80 |
| 1879 | 0 | JP | H01L | H01L0021 | 6 | 11 | 12 | 1 | 2.00 |
| 1880 | 0 | JP | C30B | C30B0019 | 4 | 8 | 10 | 1 | 5.00 |
| 1881 | 0 | US | H01L | H01L0021 | 11 | 5 | 47 | 1.142857143 | 5.88 |
| 1882 | 0 | US | F21V | F21V0023 | 18 | 19 | 1 | 1 | 0.14 |
| 1883 | 0 | US | H01L | H01L0021 | 22 | 4 | 16 | 1.111111111 | 1.60 |
| 1884 | 0 | JP | H01L | H01L0027 | 7 | 1 | 2 | 1 | 0.50 |
| 1885 | 0 | JP | H01L | H01L0027 | 75 | 8 | 90 | 4 | 11.25 |
| 1886 | 0 | US | H01L | H01L0027 | 20 | 21 | 0 | 2.5 | 0.00 |
| 1887 | 0 | JP | C01G | C01G0015 | 2 | 15 | 26 | 1.333333333 | 6.50 |
| 1888 | 0 | JP | H01L | H01L0021 | 14 | 5 | 10 | 1 | 3.33 |
| 1889 | 0 | US | H01L | H01L0033 | 10 | 18 | 0 | 1.4 | 0.00 |
| 1890 | 0 | JP | C30B | C30B0019 | 3 | 7 | 5 | 1 | 1.25 |
| 1891 | 0 | JP | H01L | H01L0021 | 8 | 10 | 3 | 1 | 1.50 |
| 1892 | 0 | JP | H01L | H01L0033 | 3 | 7 | 46 | 1 | 23.00 |
| 1893 | 0 | JP | C30B | C30B0015 | 4 | 10 | 13 | 1 | 3.25 |
| 1894 | 0 | US | H01L | H01L0033 | 7 | 6 | 7 | 1.25 | 0.70 |
| 1895 | 0 | US | H01L | H01L0021 | 4 | 14 | 0 | 1.5 | 0.00 |
| 1896 | 0 | JP | C30B | C30B0019 | 23 | 4 | 17 | 1 | 8.50 |
| 1897 | 0 | JP | H01L | H01L0021 | 16 | 11 | 32 | 1 | 8.00 |
| 1898 | 0 | JP | H01L | H01L0021 | 4 | 5 | 8 | 1 | 4.00 |
| 1899 | 0 | JP | G08B | G08B0005 | 19 | 7 | 189 | 1 | 63.00 |
| 1900 | 0 | JP | G02B | G02B0006 | 14 | 1 | 28 | 1 | 7.00 |
| 1901 | 0 | US | H01L | H01L0033 | 18 | 61 | 1 | 1 | 0.25 |
| 1902 | 0 | US | H01L | H01L0033 | 36 | 58 | 1 | 1.333333333 | 0.13 |
| 1903 | 0 | JP | H01L | H01L0033 | 22 | 16 | 10 | 1.5 | 3.33 |
| 1904 | 0 | JP | G02B | G02B0006 | 21 | 4 | 2 | 1 | 0.67 |
| 1905 | 0 | US | H01L | H01L0021 | 25 | 13 | 0 | 1 | 0.00 |
| 1906 | 0 | US | G09F | G09F0009 | 46 | 11 | 0 | 1 | 0.00 |
| 1907 | 0 | US | H01L | H01L0021 | 25 | 525 | 2 | 4 | 0.50 |
| 1908 | 0 | US | G05F | G05F0001 | 42 | 133 | 17 | 23.28571429 | 0.05 |
| 1909 | 0 | JP | G02B | G02B0006 | 7 | 2 | 16 | 1 | 5.33 |
| 1910 | 0 | US | H01L | H01L0027 | 19 | 28 | 0 | 1.5 | 0.00 |
| 1911 | 0 | US | H01L | H01L0033 | 6 | 9 | 0 | 1 | 0.00 |
| 1912 | 0 | US | F21V | F21V0033 | 16 | 179 | 0 | 1 | 0.00 |
| 1913 | 0 | JP | H01L | H01L0021 | 11 | 12 | 40 | 1 | 10.00 |
| 1914 | 0 | DE | H01L | H01L0033 | 5 | 9 | 4 | 1.25 | 0.80 |
| 1915 | 0 | JP | H01L | H01L0023 | 2 | 8 | 17 | 1 | 8.50 |
| 1916 | 0 | JP | H01L | H01L0033 | 18 | 5 | 49 | 2 | 12.25 |
| 1917 | 0 | JP | H01L | H01L0029 | 11 | 2 | 1 | 1 | 0.25 |
| 1918 | 0 | JP | H01L | H01L0021 | 5 | 1 | 23 | 1.333333333 | 5.75 |
| 1919 | 0 | JP | H01L | H01L0033 | 4 | 7 | 3 | 1.333333333 | 0.75 |
| 1920 | 0 | US | H01S | H01S0005 | 18 | 26 | 0 | 1 | 0.00 |
| 1921 | 0 | JP | H01L | H01L0033 | 15 | 1 | 3 | 1.666666667 | 0.60 |
| 1922 | 0 | JP | C09K | C09K0011 | 11 | 2 | 64 | 1.5 | 21.33 |
| 1923 | 0 | JP | C01B | C01B0025 | 6 | 3 | 5 | 1.2 | 0.83 |
| 1924 | 0 | JP | H01L | H01L0033 | 5 | 8 | 10 | 1.666666667 | 2.00 |
| 1925 | 0 | US | H01L | H01L0021 | 22 | 164 | 3 | 6.714285714 | 0.06 |
| 1926 | 0 | US | H01S | H01S0003 | 12 | 6 | 0 | 1.5 | 0.00 |
| 1927 | 0 | JP | C01G | C01G0009 | 19 | 5 | 17 | 1 | 3.40 |
| 1928 | 0 | JP | C25D | C25D0007 | 2 | 7 | 21 | 2 | 5.25 |
| 1929 | 0 | JP | H01L | H01L0033 | 15 | 1 | 27 | 2 | 6.75 |
| 1930 | 0 | US | H01L | H01L0033 | 20 | 14 | 0 | 1.5 | 0.00 |
| 1931 | 0 | JP | H01L | H01L0021 | 43 | 15 | 59 | 1.666666667 | 11.80 |
| 1932 | 0 | US | H01L | H01L0021 | 18 | 543 | 1 | 1 | 1.00 |
| 1933 | 0 | US | H01L | H01L0033 | 31 | 86 | 0 | 2 | 0.00 |
| 1934 | 0 | US | H01L | H01L0031 | 18 | 257 | 0 | 2.333333333 | 0.00 |
| 1935 | 0 | US | H01L | H01L0021 | 12 | 131 | 0 | 1 | 0.00 |
| 1936 | 0 | US | H01L | H01L0029 | 12 | 14 | 1 | 1 | 1.00 |
| 1937 | 0 | US | H01L | H01L0033 | 29 | 32 | 0 | 1.25 | 0.00 |
| 1938 | 0 | JP | C30B | C30B0031 | 8 | 20 | 16 | 1 | 3.20 |
| 1939 | 0 | JP | C30B | C30B0027 | 7 | 4 | 11 | 1 | 2.75 |
| 1940 | 0 | US | H01L | H01L0033 | 6 | 13 | 0 | 1 | 0.00 |
| 1941 | 0 | DE | H01L | H01L0033 | 7 | 2 | 30 | 1 | 10.00 |
| 1942 | 0 | JP | B32B | B32B0018 | 5 | 2 | 17 | 1 | 5.67 |
| 1943 | 0 | US | H01L | H01L0029 | 25 | 94 | 0 | 1.5 | 0.00 |
| 1944 | 0 | DE | H01L | H01L0021 | 2 | 7 | 10 | 1 | 1.67 |
| 1945 | 0 | JP | C23C | C23C0016 | 21 | 6 | 56 | 1.2 | 9.33 |
| 1946 | 0 | US | H01L | H01L0051 | 13 | 232 | 0 | 4.916666667 | 0.00 |
| 1947 | 0 | JP | H01L | H01L0021 | 12 | 6 | 13 | 1.25 | 2.60 |
| 1948 | 0 | JP | H01L | H01L0029 | 4 | 11 | 7 | 1.333333333 | 1.75 |
| 1949 | 0 | JP | H01L | H01L0033 | 12 | 2 | 11 | 1.333333333 | 2.75 |
| 1950 | 0 | JP | C08L | C08L0007 | 10 | 4 | 5 | 1.4 | 0.71 |
| 1951 | 0 | FR | H01L | H01L0033 | 7 | 11 | 36 | 1 | 9.00 |
| 1952 | 1 | JP | H01L | H01L0033 | 6 | 13 | 11 | 1.333333333 | 2.75 |
| 1953 | 0 | US | H01L | H01L0029 | 15 | 35 | 0 | 1 | 0.00 |
| 1954 | 0 | US | G02B | G02B0001 | 13 | 31 | 0 | 1 | 0.00 |
| 1955 | 0 | JP | H01L | H01L0021 | 1 | 1 | 6 | 1.2 | 1.00 |
| 1956 | 0 | US | H01L | H01L0021 | 18 | 23 | 0 | 1 | 0.00 |
| 1957 | 0 | DE | H01L | H01L0021 | 7 | 5 | 8 | 1 | 1.60 |
| 1958 | 0 | SU | H01L | H01L0021 | 8 | 3 | 27 | 1 | 3.86 |
| 1959 | 0 | US | H01L | H01L0029 | 10 | 10 | 0 | 1 | 0.00 |
| 1960 | 0 | US | H01S | H01S0005 | 26 | 156 | 0 | 2.333333333 | 0.00 |
| 1961 | 0 | JP | C30B | C30B0015 | 8 | 1 | 10 | 1.333333333 | 2.50 |
| 1962 | 0 | JP | C30B | C30B0019 | 7 | 11 | 4 | 1.4 | 0.57 |
| 1963 | 0 | JP | H01L | H01L0033 | 4 | 7 | 2 | 1.333333333 | 0.25 |
| 1964 | 0 | JP | H01L | H01L0033 | 3 | 7 | 5 | 1 | 1.67 |
| 1965 | 0 | US | H01L | H01L0021 | 3 | 7 | 2 | 1 | 0.50 |
| 1966 | 0 | JP | B41J | B41J0002 | 13 | 2 | 56 | 1 | 28.00 |
| 1967 | 0 | JP | G02B | G02B0006 | 3 | 4 | 11 | 1.222222222 | 1.00 |
| 1968 | 0 | JP | H01L | H01L0033 | 15 | 2 | 11 | 3.5 | 1.57 |
| 1969 | 0 | US | H01L | H01L0033 | 7 | 3 | 0 | 2 | 0.00 |
| 1970 | 0 | US | H01L | H01L0029 | 5 | 2 | 17 | 1.25 | 3.40 |
| 1971 | 0 | SE | H01L | H01L0033 | 10 | 7 | 4 | 1.25 | 0.80 |
| 1972 | 0 | US | H01L | H01L0021 | 4 | 1 | 0 | 1.142857143 | 0.00 |
| 1973 | 0 | US | H01L | H01L0033 | 24 | 39 | 0 | 1 | 0.00 |
| 1974 | 0 | US | C07C | C07C0017 | 15 | 1 | 5 | 1 | 1.25 |
| 1975 | 0 | US | H05B | H05B0033 | 7 | 3 | 0 | 1 | 0.00 |
| 1976 | 0 | JP | C30B | C30B0027 | 9 | 8 | 11 | 1 | 2.75 |
| 1977 | 0 | JP | H01L | H01L0021 | 2 | 10 | 4 | 1 | 1.00 |
| 1978 | 0 | US | H05B | H05B0033 | 8 | 1 | 16 | 1 | 16.00 |
| 1979 | 0 | JP | H01S | H01S0005 | 13 | 7 | 11 | 1 | 2.75 |
| 1980 | 0 | JP | H01L | H01L0033 | 14 | 3 | 23 | 1 | 11.50 |
| 1981 | 0 | US | H01L | H01L0033 | 1 | 3 | 1 | 1 | 0.50 |
| 1982 | 0 | JP | C30B | C30B0019 | 10 | 13 | 13 | 1.4 | 1.86 |
| 1983 | 0 | JP | H01L | H01L0021 | 3 | 3 | 3 | 1 | 0.75 |
| 1984 | 0 | US | H01L | H01L0033 | 9 | 2 | 8 | 1 | 8.00 |
| 1985 | 0 | JP | H01L | H01L0033 | 15 | 7 | 7 | 2.4 | 0.58 |
| 1986 | 0 | US | H01L | H01L0021 | 21 | 10 | 24 | 2 | 12.00 |
| 1987 | 0 | JP | H01L | H01L0021 | 6 | 3 | 34 | 3.666666667 | 3.09 |
| 1988 | 0 | US | C23C | C23C0014 | 7 | 4 | 14 | 1 | 3.50 |
| 1989 | 0 | US | H01L | H01L0023 | 7 | 4 | 7 | 1 | 7.00 |
| 1990 | 0 | JP | H01L | H01L0033 | 12 | 14 | 11 | 1 | 3.67 |
| 1991 | 0 | JP | C30B | C30B0019 | 14 | 7 | 1 | 1 | 0.50 |
| 1992 | 0 | JP | H01L | H01L0033 | 10 | 14 | 16 | 1.5 | 2.67 |
| 1993 | 0 | US | H01L | H01L0033 | 4 | 5 | 26 | 1 | 13.00 |
| 1994 | 0 | JP | H01L | H01L0029 | 4 | 17 | 19 | 1 | 9.50 |
| 1995 | 0 | JP | H01L | H01L0021 | 11 | 11 | 9 | 1 | 3.00 |
| 1996 | 0 | US | H01L | H01L0021 | 11 | 4 | 18 | 1 | 4.50 |
| 1997 | 0 | US | H01L | H01L0027 | 14 | 6 | 6 | 1.333333333 | 1.50 |
| 1998 | 0 | DE | H01L | H01L0021 | 11 | 3 | 9 | 1 | 3.00 |
| 1999 | 0 | US | H01L | H01L0025 | 8 | 1 | 18 | 1 | 6.00 |
| 2000 | 0 | JP | F21V | F21V0005 | 11 | 14 | 19 | 1 | 3.80 |
| 2001 | 0 | JP | H01L | H01L0021 | 17 | 9 | 21 | 2 | 5.25 |
| 2002 | 0 | JP | H01L | H01L0021 | 9 | 11 | 99 | 1 | 33.00 |
| 2003 | 0 | US | C30B | C30B0019 | 6 | 3 | 9 | 1 | 1.29 |
| 2004 | 0 | JP | H01L | H01L0021 | 11 | 8 | 7 | 1 | 2.33 |
| 2005 | 0 | JP | H01L | H01L0029 | 1 | 5 | 6 | 1 | 2.00 |
| 2006 | 0 | JP | H01L | H01L0021 | 7 | 17 | 72 | 2.666666667 | 9.00 |
| 2007 | 0 | US | H01L | H01L0033 | 5 | 0 | 5 | 1 | 5.00 |
| 2008 | 0 | JP | H01L | H01L0021 | 15 | 12 | 10 | 1 | 5.00 |
| 2009 | 0 | US | C30B | C30B0015 | 8 | 0 | 12 | 1 | 6.00 |
| 2010 | 0 | JP | G02F | G02F0003 | 12 | 4 | 6 | 1 | 3.00 |
| 2011 | 0 | US | H01L | H01L0021 | 6 | 0 | 6 | 1 | 0.55 |
| 2012 | 0 | US | H01L | H01L0033 | 3 | 0 | 3 | 1 | 3.00 |
| 2013 | 0 | US | H01L | H01L0021 | 13 | 0 | 12 | 1 | 12.00 |
| 2014 | 0 | US | H01L | H01L0021 | 7 | 6 | 80 | 1 | 20.00 |
| 2015 | 0 | US | H01L | H01L0021 | 9 | 0 | 11 | 1 | 11.00 |
| 2016 | 0 | JP | C30B | C30B0019 | 12 | 15 | 6 | 1 | 1.20 |
| 2017 | 0 | US | H01L | H01L0021 | 2 | 0 | 5 | 1 | 5.00 |
| 2018 | 0 | GB | H01L | H01L0021 | 13 | 13 | 12 | 1 | 2.40 |
| 2019 | 0 | JP | C30B | C30B0025 | 4 | 13 | 0 | 1 | 0.00 |
| 2020 | 0 | US | C30B | C30B0019 | 6 | 0 | 19 | 1 | 3.80 |
| 2021 | 0 | US | H01L | H01L0021 | 3 | 0 | 5 | 1 | 5.00 |
| 2022 | 0 | JP | H01L | H01L0033 | 29 | 1 | 14 | 1 | 3.50 |
| 2023 | 0 | JP | C30B | C30B0019 | 2 | 15 | 12 | 1.4 | 1.71 |
| 2024 | 0 | JP | B41J | B41J0002 | 2 | 9 | 42 | 1.5 | 14.00 |
| 2025 | 0 | JP | H01L | H01L0021 | 3 | 21 | 7 | 1.25 | 1.40 |
| 2026 | 0 | JP | H05K | H05K0003 | 6 | 2 | 4 | 1 | 2.00 |
| 2027 | 0 | JP | B41J | B41J0002 | 2 | 2 | 19 | 1 | 6.33 |
| 2028 | 0 | JP | H01L | H01L0021 | 2 | 2 | 22 | 2.5 | 4.40 |
| 2029 | 0 | JP | H01L | H01L0021 | 14 | 7 | 8 | 1.5 | 2.67 |
| 2030 | 0 | JP | C30B | C30B0025 | 5 | 8 | 75 | 1.75 | 10.71 |
| 2031 | 0 | JP | H01L | H01L0021 | 6 | 22 | 10 | 1.25 | 2.00 |
| 2032 | 0 | JP | C30B | C30B0029 | 9 | 14 | 118 | 1.5 | 39.33 |
| 2033 | 0 | JP | H01L | H01L0021 | 12 | 15 | 34 | 1 | 6.80 |
| 2034 | 0 | US | C30B | C30B0025 | 8 | 7 | 13 | 1 | 2.60 |
| 2035 | 0 | JP | H01J | H01J0061 | 9 | 9 | 28 | 1 | 14.00 |
| 2036 | 0 | US | G09F | G09F0009 | 12 | 15 | 6 | 1 | 3.00 |
| 2037 | 0 | JP | H01L | H01L0033 | 5 | 10 | 1 | 1 | 0.25 |
| 2038 | 0 | US | C03C | C03C0003 | 16 | 0 | 11 | 1 | 11.00 |
| 2039 | 0 | US | H01L | H01L0021 | 6 | 0 | 2 | 1 | 2.00 |
| 2040 | 0 | JP | C30B | C30B0029 | 7 | 14 | 16 | 1.2 | 2.67 |
| 2041 | 0 | JP | H01L | H01L0021 | 5 | 15 | 25 | 1 | 12.50 |
| 2042 | 0 | JP | H01L | H01L0021 | 2 | 1 | 3 | 2.5 | 0.60 |
| 2043 | 0 | DE | G02B | G02B0006 | 6 | 20 | 56 | 1 | 18.67 |
| 2044 | 0 | FI | C30B | C30B0029 | 3 | 7 | 111 | 1 | 18.50 |
| 2045 | 0 | JP | H01L | H01L0021 | 8 | 12 | 45 | 1 | 9.00 |
| 2046 | 0 | GB | B41J | B41J0002 | 7 | 3 | 2 | 1 | 0.50 |
| 2047 | 0 | US | H01L | H01L0033 | 13 | 8 | 4 | 1 | 2.00 |
| 2048 | 0 | US | C30B | C30B0023 | 7 | 1 | 3 | 1 | 1.50 |
| 2049 | 0 | US | H01L | H01L0029 | 9 | 3 | 9 | 1.25 | 1.80 |
| 2050 | 0 | JP | H01S | H01S0005 | 8 | 3 | 1 | 1.5 | 0.33 |
| 2051 | 0 | JP | H01L | H01L0021 | 7 | 4 | 15 | 1 | 7.50 |
| 2052 | 0 | JP | H01L | H01L0029 | 9 | 29 | 43 | 1 | 21.50 |
| 2053 | 0 | US | H01L | H01L0021 | 17 | 18 | 14 | 1.666666667 | 2.80 |
| 2054 | 0 | DE | H01L | H01L0021 | 5 | 15 | 4 | 1 | 1.33 |
| 2055 | 0 | GB | C23C | C23C0016 | 8 | 4 | 2 | 1 | 0.50 |
| 2056 | 1 | JP | C30B | C30B0025 | 16 | 11 | 28 | 1.333333333 | 7.00 |
| 2057 | 0 | JP | C30B | C30B0001 | 12 | 30 | 8 | 1 | 4.00 |
| 2058 | 0 | US | A61B | A61B0003 | 41 | 21 | 94 | 13.625 | 0.86 |
| 2059 | 0 | JP | H01L | H01L0021 | 9 | 18 | 24 | 1 | 4.80 |
| 2060 | 0 | JP | C30B | C30B0025 | 12 | 12 | 11 | 1 | 5.50 |
| 2061 | 1 | US | H01L | H01L0021 | 5 | 24 | 19 | 1.166666667 | 2.71 |
| 2062 | 0 | JP | C08G | C08G0059 | 16 | 3 | 7 | 1 | 3.50 |
| 2063 | 0 | JP | H01L | H01L0033 | 1 | 2 | 5 | 1 | 1.25 |
| 2064 | 0 | JP | H01L | H01L0033 | 17 | 10 | 16 | 1 | 4.00 |
| 2065 | 0 | JP | H01L | H01L0021 | 13 | 23 | 21 | 1.5 | 7.00 |
| 2066 | 0 | US | A61B | A61B0003 | 59 | 28 | 62 | 13.625 | 0.57 |
| 2067 | 0 | JP | C23C | C23C0016 | 6 | 17 | 62 | 2.333333333 | 8.86 |
| 2068 | 0 | US | C30B | C30B0029 | 9 | 8 | 25 | 1 | 8.33 |
| 2069 | 0 | TW | G09F | G09F0009 | 1 | 5 | 14 | 1 | 7.00 |
| 2070 | 0 | US | H01L | H01L0021 | 26 | 18 | 228 | 4.75 | 6.00 |
| 2071 | 0 | NL | C30B | C30B0023 | 4 | 21 | 8 | 1 | 2.00 |
| 2072 | 0 | JP | H01L | H01L0033 | 7 | 11 | 23 | 1 | 11.50 |
| 2073 | 0 | US | C30B | C30B0025 | 9 | 24 | 2 | 1 | 2.00 |
| 2074 | 0 | KR | H01L | H01L0021 | 37 | 14 | 15 | 1.5 | 5.00 |
| 2075 | 0 | US | H01L | H01L0033 | 5 | 11 | 30 | 1 | 30.00 |
| 2076 | 0 | JP | H01L | H01L0033 | 7 | 9 | 9 | 1 | 2.25 |
| 2077 | 0 | US | H01L | H01L0033 | 64 | 34 | 188 | 1.222222222 | 17.09 |
| 2078 | 0 | US | C01B | C01B0021 | 13 | 18 | 12 | 1 | 12.00 |
| 2079 | 0 | JP | H01L | H01L0021 | 4 | 48 | 5 | 1 | 0.83 |
| 2080 | 0 | JP | H01L | H01L0021 | 6 | 19 | 9 | 1 | 4.50 |
| 2081 | 0 | US | C30B | C30B0033 | 18 | 7 | 108 | 4.428571429 | 3.48 |
| 2082 | 0 | JP | H01L | H01L0021 | 7 | 18 | 58 | 2.25 | 6.44 |
| 2083 | 0 | DE | H01L | H01L0021 | 4 | 6 | 6 | 1 | 2.00 |
| 2084 | 0 | JP | B41J | B41J0002 | 20 | 3 | 29 | 1 | 9.67 |
| 2085 | 0 | JP | H01C | H01C0001 | 30 | 11 | 46 | 1 | 11.50 |
| 2086 | 0 | US | H01L | H01L0033 | 15 | 19 | 10 | 1.166666667 | 1.43 |
| 2087 | 0 | US | H01J | H01J0037 | 31 | 4 | 14 | 1 | 14.00 |
| 2088 | 0 | US | H01L | H01L0033 | 7 | 24 | 1 | 1.571428571 | 0.09 |
| 2089 | 0 | US | C30B | C30B0029 | 22 | 66 | 121 | 1.454545455 | 7.56 |
| 2090 | 0 | JP | C08G | C08G0059 | 20 | 10 | 13 | 1 | 6.50 |
| 2091 | 0 | DE | H01L | H01L0033 | 15 | 5 | 13 | 1 | 3.25 |
| 2092 | 1 | US | G02B | G02B0003 | 19 | 41 | 42 | 4.6 | 1.83 |
| 2093 | 0 | JP | C30B | C30B0019 | 4 | 5 | 7 | 1.25 | 1.40 |
| 2094 | 0 | JP | H01L | H01L0023 | 10 | 7 | 33 | 1 | 6.60 |
| 2095 | 0 | US | H01L | H01L0021 | 25 | 29 | 155 | 1 | 14.09 |
| 2096 | 0 | JP | F01N | F01N0011 | 9 | 6 | 6 | 1 | 1.20 |
| 2097 | 0 | JP | H01L | H01L0021 | 21 | 20 | 14 | 1 | 7.00 |
| 2098 | 0 | US | H05B | H05B0033 | 43 | 50 | 140 | 2.125 | 8.24 |
| 2099 | 0 | US | C30B | C30B0025 | 39 | 152 | 18 | 1.454545455 | 1.13 |
| 2100 | 0 | JP | H01L | H01L0031 | 10 | 11 | 7 | 1 | 3.50 |
| 2101 | 0 | US | A61B | A61B0003 | 17 | 29 | 19 | 13.625 | 0.17 |
| 2102 | 0 | JP | H01S | H01S0005 | 6 | 12 | 12 | 1.75 | 1.71 |
| 2103 | 0 | US | C30B | C30B0033 | 17 | 30 | 72 | 4.428571429 | 2.32 |
| 2104 | 1 | US | G02B | G02B0003 | 9 | 5 | 25 | 4.6 | 1.09 |
| 2105 | 0 | US | C30B | C30B0029 | 5 | 134 | 22 | 1.454545455 | 1.38 |
| 2106 | 0 | JP | H01L | H01L0021 | 6 | 19 | 73 | 1 | 36.50 |
| 2107 | 0 | JP | H01S | H01S0005 | 9 | 13 | 4 | 1.5 | 1.33 |
| 2108 | 0 | US | C30B | C30B0025 | 79 | 10 | 59 | 2.285714286 | 3.69 |
| 2109 | 0 | JP | G02B | G02B0006 | 17 | 7 | 4 | 1 | 0.80 |
| 2110 | 0 | JP | H01L | H01L0021 | 3 | 23 | 63 | 2.25 | 7.00 |
| 2111 | 0 | US | H01L | H01L0029 | 32 | 128 | 18 | 3 | 0.86 |
| 2112 | 0 | JP | H01L | H01L0021 | 5 | 11 | 43 | 1 | 10.75 |
| 2113 | 0 | JP | H01L | H01L0021 | 21 | 7 | 10 | 2 | 1.67 |
| 2114 | 0 | JP | G02B | G02B0006 | 12 | 13 | 14 | 1 | 7.00 |
| 2115 | 0 | JP | C30B | C30B0029 | 8 | 13 | 4 | 1.2 | 0.67 |
| 2116 | 0 | US | H01L | H01L0033 | 29 | 26 | 110 | 4.25 | 6.47 |
| 2117 | 0 | US | H01L | H01L0029 | 35 | 125 | 41 | 3 | 1.95 |
| 2118 | 0 | US | H01L | H01L0021 | 34 | 19 | 45 | 4.428571429 | 1.45 |
| 2119 | 0 | KR | C08G | C08G0083 | 10 | 4 | 18 | 1 | 9.00 |
| 2120 | 0 | US | H01L | H01L0029 | 17 | 68 | 20 | 3 | 0.95 |
| 2121 | 0 | US | C30B | C30B0029 | 49 | 47 | 68 | 2.636363636 | 2.34 |
| 2122 | 0 | JP | H01L | H01L0021 | 5 | 9 | 63 | 2.5 | 12.60 |
| 2123 | 0 | JP | H01L | H01L0033 | 14 | 15 | 7 | 1 | 1.75 |
| 2124 | 0 | JP | B23K | B23K0020 | 33 | 16 | 18 | 1.8 | 2.00 |
| 2125 | 0 | US | G02B | G02B0006 | 11 | 7 | 7 | 1.5 | 1.17 |
| 2126 | 0 | JP | C23C | C23C0016 | 6 | 15 | 69 | 1.2 | 11.50 |
| 2127 | 0 | US | C07F | C07F0005 | 19 | 7 | 1 | 1.2 | 0.17 |
| 2128 | 0 | JP | B01J | B01J0003 | 3 | 5 | 15 | 2.5 | 3.00 |
| 2129 | 0 | JP | B41J | B41J0002 | 2 | 9 | 21 | 2.333333333 | 3.00 |
| 2130 | 0 | JP | C23C | C23C0016 | 16 | 2 | 8 | 2.166666667 | 0.62 |
| 2131 | 0 | JP | H01L | H01L0021 | 2 | 5 | 11 | 1 | 3.67 |
| 2132 | 0 | JP | H01L | H01L0023 | 60 | 24 | 18 | 2.5 | 3.60 |
| 2133 | 0 | JP | G09F | G09F0013 | 21 | 4 | 17 | 2.75 | 1.55 |
| 2134 | 0 | US | H01L | H01L0021 | 33 | 69 | 19 | 3 | 0.90 |
| 2135 | 0 | JP | H01L | H01L0027 | 5 | 3 | 22 | 1 | 11.00 |
| 2136 | 0 | JP | H01L | H01L0033 | 9 | 11 | 28 | 1.666666667 | 5.60 |
| 2137 | 0 | US | G03C | G03C0001 | 32 | 23 | 5 | 1.666666667 | 1.00 |
| 2138 | 0 | JP | H01L | H01L0021 | 16 | 6 | 24 | 2.5 | 4.80 |
| 2139 | 0 | US | G02B | G02B0003 | 33 | 100 | 8 | 4.6 | 0.35 |
| 2140 | 0 | US | C08G | C08G0059 | 66 | 6 | 13 | 1.428571429 | 1.30 |
| 2141 | 0 | GB | H01L | H01L0023 | 12 | 10 | 10 | 1.142857143 | 1.25 |
| 2142 | 0 | KR | B41J | B41J0002 | 1 | 7 | 13 | 1 | 3.25 |
| 2143 | 0 | US | H01L | H01L0033 | 56 | 3 | 32 | 4.25 | 1.88 |
| 2144 | 0 | US | H01L | H01L0029 | 169 | 29 | 14 | 1.2 | 2.33 |
| 2145 | 0 | US | H01J | H01J0037 | 15 | 32 | 11 | 1.555555556 | 0.79 |
| 2146 | 0 | JP | H01L | H01L0021 | 12 | 18 | 6 | 1 | 1.20 |
| 2147 | 0 | JP | H01L | H01L0027 | 8 | 36 | 4 | 2.333333333 | 0.57 |
| 2148 | 0 | JP | H01L | H01L0033 | 13 | 4 | 2 | 1 | 0.33 |
| 2149 | 0 | JP | H01L | H01L0021 | 7 | 19 | 91 | 1 | 22.75 |
| 2150 | 0 | JP | H01L | H01L0033 | 16 | 6 | 30 | 1 | 15.00 |
| 2151 | 0 | US | C09D | C09D0005 | 45 | 64 | 1 | 1.75 | 0.14 |
| 2152 | 0 | JP | H01L | H01L0021 | 6 | 13 | 7 | 1 | 1.75 |
| 2153 | 0 | JP | H01L | H01L0021 | 2 | 6 | 22 | 1.166666667 | 3.14 |
| 2154 | 0 | GB | G02B | G02B0006 | 9 | 39 | 5 | 1.2 | 0.83 |
| 2155 | 0 | JP | H01L | H01L0021 | 4 | 3 | 15 | 2.5 | 3.00 |
| 2156 | 0 | US | G02F | G02F0001 | 131 | 143 | 27 | 4.75 | 0.71 |
| 2157 | 0 | JP | H01L | H01L0021 | 8 | 24 | 6 | 1 | 1.20 |
| 2158 | 0 | JP | C30B | C30B0017 | 5 | 7 | 12 | 1.75 | 1.71 |
| 2159 | 0 | JP | B23K | B23K0020 | 9 | 7 | 14 | 1.166666667 | 2.00 |
| 2160 | 0 | JP | H01L | H01L0033 | 16 | 7 | 7 | 1 | 1.75 |
| 2161 | 0 | US | H01L | H01L0033 | 65 | 46 | 163 | 1.222222222 | 14.82 |
| 2162 | 0 | JP | H01L | H01L0021 | 3 | 26 | 7 | 1 | 2.33 |
| 2163 | 0 | JP | H01L | H01L0033 | 8 | 24 | 1 | 1 | 0.20 |
| 2164 | 0 | US | H01L | H01L0033 | 73 | 41 | 13 | 3 | 1.08 |
| 2165 | 0 | JP | F21S | F21S0004 | 13 | 9 | 73 | 1 | 24.33 |
| 2166 | 0 | JP | G02B | G02B0006 | 18 | 6 | 14 | 1 | 2.80 |
| 2167 | 0 | US | G03C | G03C0001 | 29 | 32 | 2 | 3 | 0.13 |
| 2168 | 0 | JP | C30B | C30B0029 | 13 | 11 | 11 | 1.666666667 | 2.20 |
| 2169 | 0 | US | H01S | H01S0005 | 17 | 74 | 56 | 2.5 | 2.24 |
| 2170 | 0 | US | H01L | H01L0021 | 2 | 14 | 15 | 1.5 | 2.50 |
| 2171 | 0 | JP | G09F | G09F0013 | 20 | 3 | 18 | 2.75 | 1.64 |
| 2172 | 0 | JP | H01L | H01L0033 | 9 | 8 | 3 | 1.5 | 0.50 |
| 2173 | 0 | US | G03C | G03C0001 | 28 | 14 | 2 | 1.5 | 0.33 |
| 2174 | 0 | US | H01L | H01L0021 | 15 | 13 | 4 | 1.375 | 0.36 |
| 2175 | 0 | DE | B41J | B41J0002 | 18 | 14 | 6 | 1 | 1.20 |
| 2176 | 0 | JP | H01L | H01L0033 | 17 | 25 | 24 | 1.5 | 8.00 |
| 2177 | 0 | JP | B41J | B41J0002 | 7 | 7 | 33 | 1.25 | 6.60 |
| 2178 | 0 | GB | H01L | H01L0033 | 5 | 13 | 9 | 1 | 4.50 |
| 2179 | 0 | JP | H01L | H01L0021 | 10 | 23 | 86 | 1 | 43.00 |
| 2180 | 0 | US | H01L | H01L0031 | 9 | 84 | 10 | 9.625 | 0.13 |
| 2181 | 0 | JP | H01L | H01L0021 | 4 | 8 | 36 | 1.25 | 7.20 |
| 2182 | 0 | JP | H01L | H01L0021 | 10 | 16 | 2 | 1.333333333 | 0.50 |
| 2183 | 0 | US | H01L | H01L0021 | 19 | 131 | 8 | 9.625 | 0.10 |
| 2184 | 0 | JP | H01L | H01L0023 | 19 | 5 | 24 | 1.4 | 3.43 |
| 2185 | 0 | JP | H01L | H01L0033 | 1 | 17 | 5 | 1 | 1.67 |
| 2186 | 0 | JP | H01L | H01L0021 | 1 | 7 | 19 | 1 | 3.80 |
| 2187 | 0 | DE | H01L | H01L0033 | 13 | 6 | 9 | 1 | 2.25 |
| 2188 | 0 | JP | H01L | H01L0023 | 4 | 7 | 66 | 1 | 13.20 |
| 2189 | 0 | GB | G09F | G09F0009 | 7 | 5 | 32 | 1 | 8.00 |
| 2190 | 0 | US | H01S | H01S0005 | 14 | 40 | 3 | 4.6 | 0.13 |
| 2191 | 0 | JP | H01L | H01L0027 | 10 | 16 | 12 | 1 | 6.00 |
| 2192 | 0 | KR | H01L | H01L0027 | 35 | 3 | 0 | 1.333333333 | 0.00 |
| 2193 | 0 | JP | H01L | H01L0033 | 3 | 9 | 22 | 1 | 7.33 |
| 2194 | 0 | US | H01L | H01L0021 | 29 | 74 | 1 | 3 | 0.05 |
| 2195 | 0 | JP | H01L | H01L0033 | 20 | 9 | 20 | 1.6 | 2.50 |
| 2196 | 0 | KR | H01L | H01L0021 | 2 | 14 | 2 | 1.5 | 0.67 |
| 2197 | 0 | JP | C23C | C23C0016 | 7 | 6 | 9 | 1 | 4.50 |
| 2198 | 0 | US | H01L | H01L0029 | 18 | 11 | 9 | 9.625 | 0.12 |
| 2199 | 0 | JP | H01L | H01L0027 | 2 | 12 | 28 | 1 | 7.00 |
| 2200 | 0 | JP | H01L | H01L0021 | 7 | 5 | 10 | 1 | 5.00 |
| 2201 | 0 | US | H01L | H01L0029 | 33 | 25 | 2 | 1.8 | 0.22 |
| 2202 | 0 | JP | H01L | H01L0021 | 8 | 3 | 6 | 1 | 3.00 |
| 2203 | 0 | US | H01L | H01L0021 | 17 | 21 | 0 | 1.5 | 0.00 |
| 2204 | 0 | JP | H01L | H01L0033 | 9 | 4 | 20 | 1.25 | 4.00 |
| 2205 | 0 | US | H01L | H01L0021 | 14 | 0 | 0 | 2 | 0.00 |
| 2206 | 0 | US | C23C | C23C0014 | 15 | 45 | 1 | 3.75 | 0.03 |
| 2207 | 0 | JP | H01L | H01L0023 | 22 | 14 | 86 | 1.4 | 12.29 |
| 2208 | 0 | JP | H01L | H01L0021 | 18 | 17 | 100 | 1.5 | 33.33 |
| 2209 | 0 | JP | H01L | H01L0033 | 17 | 3 | 30 | 1.5 | 5.00 |
| 2210 | 0 | US | H01J | H01J0001 | 22 | 19 | 11 | 4.222222222 | 0.29 |
| 2211 | 0 | US | H01J | H01J0009 | 10 | 20 | 1 | 2.333333333 | 0.07 |
| 2212 | 0 | US | H01L | H01L0029 | 30 | 52 | 5 | 4.222222222 | 0.13 |
| 2213 | 0 | FR | H01J | H01J0001 | 34 | 11 | 75 | 1.166666667 | 10.71 |
| 2214 | 0 | JP | C23C | C23C0016 | 7 | 7 | 3 | 1.5 | 1.00 |
| 2215 | 0 | JP | H01L | H01L0033 | 3 | 10 | 28 | 1 | 14.00 |
| 2216 | 0 | JP | H01L | H01L0033 | 30 | 10 | 7 | 1 | 3.50 |
| 2217 | 0 | US | H01L | H01L0021 | 4 | 20 | 2 | 2.666666667 | 0.25 |
| 2218 | 0 | JP | H01L | H01L0029 | 14 | 8 | 8 | 1 | 1.60 |
| 2219 | 0 | JP | B41J | B41J0002 | 2 | 14 | 6 | 1.25 | 1.20 |
| 2220 | 0 | JP | H05H | H05H0003 | 12 | 10 | 10 | 1.5 | 3.33 |
| 2221 | 0 | US | H01L | H01L0033 | 8 | 40 | 2 | 1.4 | 0.29 |
| 2222 | 0 | US | H01L | H01L0027 | 6 | 25 | 1 | 2.666666667 | 0.13 |
| 2223 | 0 | JP | H01L | H01L0027 | 3 | 40 | 5 | 2.333333333 | 0.71 |
| 2224 | 0 | JP | H01L | H01L0031 | 6 | 8 | 3 | 1.125 | 0.33 |
| 2225 | 0 | JP | H01L | H01L0033 | 5 | 9 | 22 | 1.75 | 3.14 |
| 2226 | 0 | US | C30B | C30B0025 | 16 | 102 | 0 | 2.4 | 0.00 |
| 2227 | 0 | JP | G02B | G02B0006 | 7 | 16 | 6 | 1 | 1.20 |
| 2228 | 0 | US | H01L | H01L0021 | 38 | 103 | 2 | 1.333333333 | 0.25 |
| 2229 | 0 | US | H01J | H01J0001 | 24 | 49 | 0 | 4.25 | 0.00 |
| 2230 | 0 | JP | G02F | G02F0001 | 33 | 5 | 7 | 1 | 2.33 |
| 2231 | 0 | JP | H01L | H01L0033 | 10 | 13 | 4 | 1.25 | 0.80 |
| 2232 | 0 | JP | H01L | H01L0029 | 12 | 11 | 20 | 1.25 | 4.00 |
| 2233 | 0 | BE | H01L | H01L0033 | 19 | 17 | 6 | 1 | 1.50 |
| 2234 | 0 | JP | H01L | H01L0021 | 14 | 11 | 10 | 1 | 2.50 |
| 2235 | 0 | JP | H01L | H01L0021 | 14 | 19 | 73 | 1.6 | 9.13 |
| 2236 | 0 | US | H01L | H01L0021 | 11 | 15 | 0 | 1.5 | 0.00 |
| 2237 | 0 | JP | H01L | H01L0021 | 33 | 9 | 28 | 1.2 | 4.67 |
| 2238 | 0 | JP | H01L | H01L0033 | 34 | 7 | 2 | 1.25 | 0.40 |
| 2239 | 0 | US | H01L | H01L0029 | 17 | 32 | 0 | 1.8 | 0.00 |
| 2240 | 0 | US | H01J | H01J0001 | 22 | 16 | 0 | 4.222222222 | 0.00 |
| 2241 | 0 | US | H01L | H01L0029 | 29 | 135 | 0 | 4.222222222 | 0.00 |
| 2242 | 0 | JP | H01L | H01L0023 | 7 | 3 | 20 | 1 | 6.67 |
| 2243 | 0 | JP | H01L | H01L0023 | 10 | 2 | 15 | 1 | 7.50 |
| 2244 | 0 | JP | G02F | G02F0001 | 16 | 13 | 12 | 1 | 6.00 |
| 2245 | 0 | US | H01L | H01L0029 | 20 | 105 | 0 | 2.4 | 0.00 |
| 2246 | 0 | JP | C30B | C30B0025 | 10 | 20 | 26 | 1 | 6.50 |
| 2247 | 0 | US | H01L | H01L0031 | 18 | 4 | 0 | 2.25 | 0.00 |
| 2248 | 0 | JP | H01L | H01L0023 | 10 | 12 | 4 | 1.5 | 1.33 |
| 2249 | 0 | GB | H01L | H01L0021 | 16 | 16 | 6 | 1 | 1.00 |
| 2250 | 0 | JP | C30B | C30B0025 | 10 | 2 | 118 | 1 | 39.33 |
| 2251 | 0 | US | H01L | H01L0021 | 29 | 19 | 0 | 1.75 | 0.00 |
| 2252 | 0 | US | H01L | H01L0021 | 13 | 8 | 14 | 1 | 2.80 |
| 2253 | 0 | JP | H01L | H01L0033 | 20 | 10 | 15 | 1 | 3.75 |
| 2254 | 0 | US | H01L | H01L0029 | 21 | 5 | 0 | 1.2 | 0.00 |
| 2255 | 0 | JP | H01L | H01L0025 | 21 | 9 | 14 | 1 | 2.80 |
| 2256 | 0 | JP | H01L | H01L0023 | 6 | 16 | 41 | 1 | 5.13 |
| 2257 | 0 | JP | H01L | H01L0021 | 4 | 32 | 76 | 1.666666667 | 15.20 |
| 2258 | 0 | JP | H01L | H01L0029 | 20 | 21 | 12 | 1.333333333 | 3.00 |
| 2259 | 0 | JP | H01L | H01L0021 | 9 | 4 | 2 | 1.5 | 0.67 |
| 2260 | 0 | JP | G01R | G01R0031 | 7 | 1 | 24 | 1.5 | 4.00 |
| 2261 | 0 | JP | H01L | H01L0033 | 19 | 15 | 27 | 1.2 | 4.50 |
| 2262 | 0 | US | C30B | C30B0023 | 29 | 31 | 158 | 2 | 11.29 |
| 2263 | 0 | JP | H01L | H01L0021 | 5 | 4 | 5 | 1.5 | 1.67 |
| 2264 | 0 | JP | H01L | H01L0029 | 11 | 13 | 11 | 1 | 2.75 |
| 2265 | 0 | JP | H01L | H01L0021 | 20 | 6 | 17 | 1 | 5.67 |
| 2266 | 0 | US | G09F | G09F0009 | 7 | 4 | 17 | 1 | 1.70 |
| 2267 | 0 | JP | H01S | H01S0005 | 10 | 1 | 10 | 1 | 2.50 |
| 2268 | 0 | JP | H01S | H01S0005 | 4 | 9 | 8 | 1 | 2.67 |
| 2269 | 0 | US | H01L | H01L0021 | 13 | 3 | 10 | 1 | 1.67 |
| 2270 | 0 | JP | H01L | H01L0033 | 20 | 11 | 20 | 1.6 | 2.50 |
| 2271 | 0 | US | H01L | H01L0021 | 4 | 4 | 15 | 1.428571429 | 1.50 |
| 2272 | 0 | US | H01L | H01L0023 | 10 | 4 | 7 | 1 | 1.17 |
| 2273 | 0 | FR | H01L | H01L0029 | 19 | 15 | 69 | 1 | 17.25 |
| 2274 | 0 | JP | H01S | H01S0005 | 3 | 44 | 20 | 1.25 | 4.00 |
| 2275 | 0 | JP | H01L | H01L0033 | 1 | 18 | 4 | 1 | 2.00 |
| 2276 | 0 | JP | H05H | H05H0003 | 6 | 9 | 1 | 1.5 | 0.33 |
| 2277 | 0 | US | C01B | C01B0025 | 3 | 5 | 2 | 1 | 0.29 |
| 2278 | 0 | JP | H01L | H01L0021 | 11 | 21 | 43 | 3 | 7.17 |
| 2279 | 0 | JP | H01S | H01S0005 | 15 | 9 | 9 | 1 | 4.50 |
| 2280 | 0 | US | H01L | H01L0025 | 7 | 9 | 153 | 1 | 30.60 |
| 2281 | 0 | JP | G03F | G03F0007 | 13 | 8 | 51 | 1.5 | 17.00 |
| 2282 | 0 | US | H01J | H01J0001 | 5 | 7 | 18 | 1 | 3.00 |
| 2283 | 0 | US | G09F | G09F0013 | 11 | 6 | 6 | 1 | 1.00 |
| 2284 | 0 | JP | C23C | C23C0014 | 5 | 16 | 25 | 1 | 5.00 |
| 2285 | 0 | US | H01L | H01L0021 | 14 | 4 | 19 | 2 | 2.38 |
| 2286 | 0 | US | H01L | H01L0021 | 6 | 5 | 18 | 2 | 9.00 |
| 2287 | 0 | US | H01L | H01L0021 | 6 | 5 | 12 | 2 | 6.00 |
| 2288 | 0 | JP | G02F | G02F0001 | 24 | 8 | 8 | 1.25 | 1.60 |
| 2289 | 0 | US | H01L | H01L0021 | 7 | 7 | 1 | 1 | 0.20 |
| 2290 | 0 | US | C30B | C30B0019 | 2 | 6 | 1 | 1.111111111 | 0.10 |
| 2291 | 0 | US | H01L | H01L0021 | 4 | 6 | 14 | 4 | 3.50 |
| 2292 | 0 | DE | G09F | G09F0009 | 15 | 19 | 27 | 1 | 13.50 |
| 2293 | 0 | JP | H01L | H01L0021 | 2 | 6 | 35 | 1.166666667 | 5.00 |
| 2294 | 0 | JP | C30B | C30B0019 | 18 | 5 | 1 | 1.333333333 | 0.25 |
| 2295 | 0 | US | H01L | H01L0021 | 3 | 4 | 6 | 1.285714286 | 0.67 |
| 2296 | 0 | JP | H01L | H01L0033 | 33 | 10 | 7 | 1 | 2.33 |
| 2297 | 0 | JP | G02B | G02B0006 | 49 | 28 | 29 | 2.5 | 2.90 |
| 2298 | 0 | JP | G09F | G09F0009 | 10 | 6 | 31 | 1 | 15.50 |
| 2299 | 0 | US | G09F | G09F0013 | 10 | 11 | 44 | 1 | 11.00 |
| 2300 | 0 | DE | H01L | H01L0033 | 19 | 10 | 5 | 1 | 1.25 |
| 2301 | 0 | JP | H01S | H01S0005 | 9 | 26 | 31 | 1 | 10.33 |
| 2302 | 0 | JP | H01L | H01L0033 | 11 | 6 | 18 | 1.5 | 3.00 |
| 2303 | 0 | US | C30B | C30B0019 | 8 | 13 | 7 | 1.25 | 1.40 |
| 2304 | 0 | US | G09F | G09F0009 | 8 | 8 | 22 | 1.1 | 2.00 |
| 2305 | 0 | US | H01L | H01L0021 | 4 | 6 | 8 | 2 | 4.00 |
| 2306 | 0 | JP | C30B | C30B0023 | 5 | 17 | 3 | 2.25 | 0.33 |
| 2307 | 0 | US | C23C | C23C0016 | 20 | 7 | 61 | 1 | 12.20 |
| 2308 | 0 | JP | H01L | H01L0033 | 34 | 19 | 100 | 1 | 50.00 |
| 2309 | 0 | JP | H01L | H01L0021 | 4 | 25 | 45 | 1 | 9.00 |
| 2310 | 0 | US | H01L | H01L0021 | 12 | 4 | 13 | 1.230769231 | 0.81 |
| 2311 | 0 | US | H01L | H01L0023 | 6 | 2 | 20 | 1 | 5.00 |
| 2312 | 0 | JP | H01L | H01L0021 | 2 | 10 | 9 | 1 | 2.25 |
| 2313 | 0 | JP | H01L | H01L0023 | 4 | 9 | 8 | 1 | 4.00 |
| 2314 | 0 | JP | H01L | H01L0027 | 3 | 43 | 5 | 2.333333333 | 0.71 |
| 2315 | 0 | JP | H01L | H01L0023 | 10 | 12 | 5 | 1 | 1.00 |
| 2316 | 0 | JP | G02B | G02B0006 | 2 | 7 | 62 | 1.333333333 | 15.50 |
| 2317 | 0 | JP | H01L | H01L0029 | 12 | 7 | 35 | 1 | 17.50 |
| 2318 | 0 | JP | H01L | H01L0031 | 8 | 2 | 5 | 1 | 2.50 |
| 2319 | 0 | US | H01L | H01L0029 | 4 | 7 | 5 | 1 | 1.67 |
| 2320 | 0 | JP | H01S | H01S0005 | 11 | 17 | 7 | 1.25 | 1.40 |
| 2321 | 0 | US | H01L | H01L0021 | 5 | 5 | 7 | 2 | 0.88 |
| 2322 | 0 | US | H01L | H01L0033 | 7 | 1 | 3 | 2.166666667 | 0.23 |
| 2323 | 0 | JP | H01L | H01L0021 | 18 | 6 | 47 | 1.5 | 15.67 |
| 2324 | 0 | JP | H01L | H01L0021 | 8 | 34 | 31 | 1 | 7.75 |
| 2325 | 0 | US | C30B | C30B0019 | 3 | 4 | 4 | 2.333333333 | 0.19 |
| 2326 | 0 | JP | C09J | C09J0007 | 2 | 20 | 39 | 1 | 7.80 |
| 2327 | 0 | JP | H01L | H01L0023 | 5 | 14 | 30 | 1 | 15.00 |
| 2328 | 0 | JP | H01L | H01L0033 | 10 | 4 | 17 | 2.5 | 3.40 |
| 2329 | 0 | FR | H01L | H01L0021 | 8 | 8 | 16 | 1 | 3.20 |
| 2330 | 0 | JP | C30B | C30B0015 | 15 | 20 | 7 | 1 | 1.00 |
| 2331 | 0 | JP | C30B | C30B0025 | 26 | 9 | 3 | 1 | 1.50 |
| 2332 | 0 | US | H01L | H01L0033 | 5 | 6 | 5 | 1 | 0.83 |
| 2333 | 0 | US | G09F | G09F0009 | 8 | 2 | 49 | 1 | 4.90 |
| 2334 | 0 | JP | H01L | H01L0033 | 20 | 7 | 18 | 1 | 9.00 |
| 2335 | 0 | JP | H01S | H01S0005 | 12 | 3 | 13 | 1 | 3.25 |
| 2336 | 0 | JP | H01L | H01L0021 | 8 | 13 | 6 | 1 | 3.00 |
| 2337 | 0 | US | H01L | H01L0029 | 17 | 20 | 88 | 1 | 44.00 |
| 2338 | 0 | US | G01N | G01N0033 | 2 | 6 | 14 | 1 | 3.50 |
| 2339 | 0 | JP | H01L | H01L0021 | 7 | 25 | 50 | 1.75 | 7.14 |
| 2340 | 0 | JP | H01L | H01L0025 | 8 | 10 | 9 | 1 | 1.80 |
| 2341 | 0 | US | C08G | C08G0018 | 12 | 2 | 25 | 2 | 6.25 |
| 2342 | 0 | US | H01L | H01L0021 | 6 | 7 | 29 | 1 | 14.50 |
| 2343 | 0 | US | G09F | G09F0009 | 7 | 3 | 17 | 1 | 3.40 |
| 2344 | 0 | JP | C08K | C08K0003 | 5 | 53 | 0 | 1.285714286 | 0.00 |
| 2345 | 0 | US | H01L | H01L0029 | 10 | 4 | 14 | 1 | 4.67 |
| 2346 | 0 | US | H01L | H01L0027 | 5 | 8 | 16 | 2 | 2.67 |
| 2347 | 0 | US | H01L | H01L0031 | 11 | 4 | 6 | 2.333333333 | 0.29 |
| 2348 | 0 | US | C30B | C30B0023 | 16 | 13 | 42 | 1 | 6.00 |
| 2349 | 0 | JP | H01L | H01L0031 | 4 | 5 | 22 | 1 | 7.33 |
| 2350 | 0 | JP | H01L | H01L0021 | 2 | 20 | 7 | 1 | 3.50 |
| 2351 | 0 | JP | H01L | H01L0021 | 23 | 10 | 42 | 1 | 21.00 |
| 2352 | 0 | JP | H01L | H01L0031 | 3 | 9 | 23 | 1 | 4.60 |
| 2353 | 0 | JP | G02F | G02F0001 | 29 | 5 | 10 | 1 | 5.00 |
| 2354 | 0 | US | C30B | C30B0019 | 8 | 3 | 6 | 2.333333333 | 0.29 |
| 2355 | 0 | JP | H01L | H01L0033 | 22 | 13 | 9 | 1.75 | 1.29 |
| 2356 | 0 | JP | H01L | H01L0021 | 5 | 12 | 51 | 2 | 8.50 |
| 2357 | 0 | JP | H01L | H01L0033 | 8 | 1 | 65 | 2.5 | 13.00 |
| 2358 | 0 | JP | H01L | H01L0021 | 2 | 9 | 72 | 1.25 | 14.40 |
| 2359 | 0 | JP | H01L | H01L0033 | 9 | 7 | 2 | 1 | 1.00 |
| 2360 | 0 | JP | G02B | G02B0006 | 11 | 14 | 9 | 1 | 1.80 |
| 2361 | 0 | JP | H01L | H01L0021 | 23 | 9 | 45 | 3.25 | 3.46 |
| 2362 | 0 | KR | H01L | H01L0027 | 26 | 3 | 4 | 1.333333333 | 1.00 |
| 2363 | 0 | JP | H01L | H01L0033 | 12 | 5 | 46 | 1.333333333 | 11.50 |
| 2364 | 0 | US | H01L | H01L0021 | 8 | 10 | 24 | 4 | 6.00 |
| 2365 | 0 | JP | H01L | H01L0021 | 17 | 7 | 37 | 1.2 | 6.17 |
| 2366 | 0 | US | G09F | G09F0009 | 12 | 7 | 17 | 1 | 5.67 |
| 2367 | 0 | JP | C30B | C30B0023 | 8 | 18 | 2 | 1 | 0.50 |
| 2368 | 0 | US | G09F | G09F0009 | 5 | 5 | 33 | 1 | 5.50 |
| 2369 | 0 | JP | C30B | C30B0025 | 5 | 14 | 67 | 1 | 13.40 |
| 2370 | 0 | JP | H01L | H01L0029 | 19 | 10 | 9 | 1 | 4.50 |
| 2371 | 0 | JP | C30B | C30B0029 | 12 | 8 | 14 | 1 | 7.00 |
| 2372 | 0 | JP | H01L | H01L0031 | 4 | 14 | 9 | 1 | 2.25 |
| 2373 | 0 | JP | C30B | C30B0019 | 13 | 16 | 5 | 1 | 1.25 |
| 2374 | 0 | US | H01L | H01L0021 | 16 | 11 | 30 | 1 | 6.00 |
| 2375 | 0 | JP | C23C | C23C0016 | 28 | 8 | 14 | 2.166666667 | 1.08 |
| 2376 | 0 | US | H01L | H01L0021 | 6 | 11 | 22 | 1 | 4.40 |
| 2377 | 0 | US | H01L | H01L0021 | 12 | 6 | 22 | 1 | 3.67 |
| 2378 | 0 | US | H01L | H01L0021 | 3 | 13 | 10 | 4 | 2.50 |
| 2379 | 0 | US | H01L | H01L0021 | 14 | 14 | 19 | 4 | 4.75 |
| 2380 | 0 | US | G02B | G02B0006 | 20 | 4 | 38 | 1 | 6.33 |
| 2381 | 0 | US | H01L | H01L0021 | 6 | 4 | 11 | 2 | 2.75 |
| 2382 | 0 | JP | C30B | C30B0025 | 1 | 13 | 10 | 1 | 2.50 |
| 2383 | 0 | JP | C09K | C09K0011 | 6 | 4 | 1 | 1.25 | 0.20 |
| 2384 | 0 | JP | H01L | H01L0033 | 16 | 3 | 47 | 1 | 15.67 |
| 2385 | 0 | JP | H01L | H01L0033 | 16 | 15 | 4 | 1.4 | 0.57 |
| 2386 | 0 | JP | H01L | H01L0033 | 8 | 10 | 8 | 1 | 4.00 |
| 2387 | 0 | US | H01L | H01L0021 | 4 | 8 | 11 | 1 | 1.57 |
| 2388 | 0 | JP | C09K | C09K0011 | 7 | 9 | 10 | 1 | 3.33 |
| 2389 | 0 | JP | H01L | H01L0027 | 3 | 32 | 6 | 2.333333333 | 0.86 |
| 2390 | 0 | JP | H01L | H01L0023 | 10 | 14 | 15 | 3 | 2.50 |
| 2391 | 0 | JP | H01L | H01L0033 | 19 | 35 | 49 | 3.4 | 2.88 |
| 2392 | 0 | JP | H01L | H01L0033 | 39 | 15 | 6 | 1.5 | 1.00 |
| 2393 | 0 | DE | C23F | C23F0004 | 7 | 3 | 51 | 1 | 10.20 |
| 2394 | 0 | JP | H01L | H01L0033 | 6 | 25 | 75 | 1.2 | 12.50 |
| 2395 | 0 | JP | H01S | H01S0005 | 20 | 7 | 19 | 2 | 4.75 |
| 2396 | 0 | JP | C23C | C23C0016 | 24 | 26 | 22 | 2.166666667 | 1.69 |
| 2397 | 0 | US | C06B | C06B0023 | 9 | 11 | 14 | 1.875 | 0.93 |
| 2398 | 0 | JP | H01L | H01L0031 | 38 | 12 | 50 | 4 | 4.17 |
| 2399 | 0 | US | H01L | H01L0021 | 3 | 7 | 9 | 2 | 2.25 |
| 2400 | 0 | JP | H01L | H01L0021 | 20 | 6 | 2 | 1 | 1.00 |
| 2401 | 0 | JP | C30B | C30B0025 | 16 | 22 | 193 | 1 | 38.60 |
| 2402 | 0 | JP | H01M | H01M0004 | 35 | 10 | 17 | 1.8 | 1.89 |
| 2403 | 0 | US | C30B | C30B0025 | 12 | 11 | 43 | 1 | 8.60 |
| 2404 | 0 | JP | H01L | H01L0023 | 4 | 14 | 36 | 2 | 4.50 |
| 2405 | 0 | JP | C23C | C23C0016 | 34 | 64 | 158 | 1 | 31.60 |
| 2406 | 0 | JP | H01L | H01L0021 | 23 | 4 | 5 | 1 | 1.25 |
| 2407 | 0 | JP | H01L | H01L0023 | 7 | 10 | 18 | 1.5 | 3.00 |
| 2408 | 0 | JP | H01L | H01L0027 | 4 | 5 | 16 | 1.5 | 5.33 |
| 2409 | 0 | JP | H01L | H01L0021 | 6 | 10 | 9 | 1 | 4.50 |
| 2410 | 0 | JP | H01L | H01L0021 | 8 | 13 | 7 | 1 | 1.40 |
| 2411 | 0 | JP | H01L | H01L0033 | 23 | 9 | 50 | 1.333333333 | 12.50 |
| 2412 | 0 | US | H01L | H01L0033 | 8 | 7 | 5 | 1 | 1.25 |
| 2413 | 0 | JP | H01S | H01S0005 | 29 | 7 | 12 | 1 | 6.00 |
| 2414 | 0 | US | H01L | H01L0021 | 2 | 4 | 3 | 2 | 0.75 |
| 2415 | 0 | US | C30B | C30B0019 | 17 | 14 | 15 | 1.142857143 | 1.88 |
| 2416 | 0 | JP | H01L | H01L0021 | 4 | 11 | 1 | 1 | 0.33 |
| 2417 | 0 | US | C30B | C30B0019 | 7 | 11 | 18 | 1.142857143 | 2.25 |
| 2418 | 0 | JP | H01L | H01L0021 | 15 | 12 | 11 | 1.25 | 2.20 |
| 2419 | 0 | JP | H01L | H01L0021 | 22 | 2 | 87 | 3.25 | 6.69 |
| 2420 | 0 | JP | G01N | G01N0003 | 15 | 5 | 30 | 2.333333333 | 4.29 |
| 2421 | 0 | JP | H01L | H01L0021 | 6 | 10 | 6 | 1 | 1.50 |
| 2422 | 0 | JP | C30B | C30B0025 | 8 | 11 | 20 | 1.2 | 3.33 |
| 2423 | 0 | JP | C30B | C30B0023 | 4 | 41 | 12 | 1.5 | 2.00 |
| 2424 | 0 | JP | B23K | B23K0001 | 24 | 7 | 16 | 1 | 5.33 |
| 2425 | 0 | JP | H01L | H01L0033 | 8 | 11 | 136 | 1.75 | 19.43 |
| 2426 | 0 | FR | H01S | H01S0005 | 8 | 15 | 2 | 1 | 0.40 |
| 2427 | 0 | US | C01G | C01G0028 | 28 | 14 | 62 | 1 | 15.50 |
| 2428 | 0 | US | H01L | H01L0027 | 3 | 6 | 2 | 1.2 | 0.33 |
| 2429 | 0 | JP | H01L | H01L0033 | 16 | 16 | 51 | 2 | 6.38 |
| 2430 | 0 | JP | H01L | H01L0021 | 3 | 19 | 5 | 1 | 1.25 |
| 2431 | 0 | JP | H01L | H01L0033 | 22 | 15 | 20 | 1 | 10.00 |
| 2432 | 0 | JP | H01L | H01L0033 | 15 | 14 | 6 | 1.75 | 0.86 |
| 2433 | 0 | US | H01L | H01L0033 | 2 | 3 | 22 | 1 | 11.00 |
| 2434 | 0 | JP | C08G | C08G0059 | 9 | 21 | 15 | 1 | 5.00 |
| 2435 | 0 | US | H01J | H01J0009 | 15 | 31 | 32 | 1.333333333 | 8.00 |
| 2436 | 0 | JP | H01L | H01L0023 | 5 | 6 | 11 | 1.333333333 | 2.75 |
| 2437 | 0 | US | H01L | H01L0029 | 11 | 8 | 39 | 1 | 4.33 |
| 2438 | 0 | JP | H01L | H01L0033 | 66 | 3 | 16 | 1 | 4.00 |
| 2439 | 0 | US | H01L | H01L0021 | 5 | 11 | 70 | 1.6 | 8.75 |
| 2440 | 0 | US | G09F | G09F0009 | 8 | 3 | 13 | 1.25 | 2.60 |
| 2441 | 0 | US | G02F | G02F0001 | 3 | 5 | 23 | 1 | 11.50 |
| 2442 | 0 | US | H01L | H01L0021 | 4 | 14 | 28 | 2 | 14.00 |
| 2443 | 0 | JP | H01L | H01L0033 | 6 | 8 | 6 | 1 | 2.00 |
| 2444 | 0 | JP | H01L | H01L0021 | 14 | 7 | 17 | 1 | 8.50 |
| 2445 | 0 | JP | H01L | H01L0021 | 26 | 22 | 26 | 3.5 | 3.71 |
| 2446 | 0 | JP | H01L | H01L0021 | 17 | 18 | 11 | 1 | 2.20 |
| 2447 | 0 | JP | G02B | G02B0006 | 24 | 23 | 77 | 2 | 9.63 |
| 2448 | 0 | JP | C30B | C30B0025 | 17 | 23 | 53 | 1.5 | 8.83 |
| 2449 | 0 | KR | C30B | C30B0019 | 7 | 9 | 2 | 1.333333333 | 0.50 |
| 2450 | 0 | JP | C30B | C30B0025 | 22 | 14 | 68 | 1 | 17.00 |
| 2451 | 0 | JP | H01L | H01L0023 | 10 | 12 | 24 | 1.5 | 4.00 |
| 2452 | 0 | JP | H01L | H01L0023 | 18 | 19 | 13 | 3 | 0.87 |
| 2453 | 0 | GB | H01L | H01L0033 | 19 | 12 | 5 | 1.166666667 | 0.71 |
| 2454 | 0 | US | G01D | G01D0015 | 7 | 11 | 3 | 1.2 | 0.50 |
| 2455 | 0 | JP | H01L | H01L0033 | 11 | 24 | 13 | 1 | 6.50 |
| 2456 | 0 | JP | H01L | H01L0033 | 1 | 16 | 2 | 1 | 0.50 |
| 2457 | 0 | US | H01L | H01L0033 | 5 | 3 | 4 | 1 | 1.33 |
| 2458 | 0 | US | H01L | H01L0021 | 14 | 9 | 17 | 1 | 3.40 |
| 2459 | 0 | JP | H01L | H01L0021 | 14 | 17 | 5 | 1 | 2.50 |
| 2460 | 0 | JP | B41J | B41J0002 | 16 | 7 | 50 | 1 | 25.00 |
| 2461 | 0 | JP | H01L | H01L0033 | 7 | 12 | 2 | 1 | 1.00 |
| 2462 | 0 | US | H01L | H01L0029 | 6 | 4 | 30 | 1 | 15.00 |
| 2463 | 0 | US | H01L | H01L0033 | 6 | 4 | 10 | 2.166666667 | 0.77 |
| 2464 | 0 | US | H01S | H01S0005 | 28 | 7 | 4 | 2 | 2.00 |
| 2465 | 1 | TW | H01L | H01L0033 | 4 | 3 | 19 | 1 | 6.33 |
| 2466 | 0 | JP | H01L | H01L0021 | 9 | 6 | 17 | 1 | 8.50 |
| 2467 | 0 | JP | C23C | C23C0014 | 11 | 31 | 8 | 1 | 1.33 |
| 2468 | 0 | JP | H01L | H01L0027 | 2 | 11 | 14 | 1.5 | 4.67 |
| 2469 | 0 | US | H01L | H01L0021 | 35 | 4 | 49 | 1 | 24.50 |
| 2470 | 0 | US | H01L | H01L0021 | 9 | 14 | 8 | 1.2 | 1.33 |
| 2471 | 0 | JP | C30B | C30B0025 | 10 | 31 | 3 | 1 | 0.60 |
| 2472 | 0 | US | H01S | H01S0005 | 21 | 7 | 45 | 1 | 15.00 |
| 2473 | 0 | JP | C30B | C30B0019 | 7 | 10 | 2 | 1 | 0.50 |
| 2474 | 0 | JP | H01L | H01L0033 | 18 | 11 | 114 | 1 | 57.00 |
| 2475 | 0 | JP | H01L | H01L0021 | 2 | 9 | 10 | 1 | 2.00 |
| 2476 | 0 | US | C23C | C23C0014 | 10 | 10 | 11 | 1 | 1.83 |
| 2477 | 0 | JP | H01L | H01L0023 | 18 | 21 | 15 | 2 | 1.07 |
| 2478 | 0 | JP | C09K | C09K0011 | 11 | 9 | 12 | 1 | 2.40 |
| 2479 | 0 | JP | H01L | H01L0021 | 7 | 21 | 10 | 1 | 2.50 |
| 2480 | 0 | JP | H01L | H01L0021 | 7 | 8 | 25 | 1 | 12.50 |
| 2481 | 0 | US | H01L | H01L0021 | 17 | 12 | 43 | 1.5 | 4.78 |
| 2482 | 0 | JP | H01L | H01L0021 | 37 | 20 | 22 | 1 | 7.33 |
| 2483 | 0 | JP | H01S | H01S0005 | 10 | 23 | 4 | 1.666666667 | 0.40 |
| 2484 | 0 | US | H01S | H01S0005 | 9 | 12 | 10 | 1.25 | 2.00 |
| 2485 | 0 | JP | C23C | C23C0016 | 9 | 6 | 0 | 1 | 0.00 |
| 2486 | 0 | US | H01L | H01L0033 | 12 | 5 | 28 | 2 | 14.00 |
| 2487 | 0 | US | H01L | H01L0033 | 15 | 12 | 7 | 1.25 | 1.40 |
| 2488 | 0 | US | H01L | H01L0033 | 10 | 7 | 8 | 1 | 1.60 |
| 2489 | 0 | JP | H01L | H01L0021 | 7 | 7 | 7 | 1.5 | 2.33 |
| 2490 | 0 | JP | H01L | H01L0033 | 7 | 5 | 1 | 1 | 0.50 |
| 2491 | 0 | US | H01L | H01L0021 | 6 | 15 | 8 | 2 | 4.00 |
| 2492 | 0 | US | H01L | H01L0021 | 11 | 24 | 52 | 1.75 | 7.43 |
| 2493 | 0 | JP | H01L | H01L0021 | 3 | 5 | 4 | 1 | 1.00 |
| 2494 | 0 | JP | H01L | H01L0033 | 3 | 21 | 59 | 1 | 19.67 |
| 2495 | 0 | BE | H01L | H01L0027 | 6 | 12 | 5 | 1.142857143 | 0.63 |
| 2496 | 0 | JP | H01L | H01L0021 | 2 | 13 | 109 | 1.25 | 21.80 |
| 2497 | 0 | JP | H01L | H01L0033 | 16 | 5 | 6 | 1 | 3.00 |
| 2498 | 0 | US | H01L | H01L0021 | 2 | 9 | 10 | 1.25 | 2.00 |
| 2499 | 0 | JP | C30B | C30B0025 | 15 | 11 | 4 | 1.2 | 0.67 |
| 2500 | 0 | JP | H01L | H01L0021 | 4 | 14 | 1 | 1.2 | 0.17 |
| 2501 | 0 | US | H01L | H01L0021 | 27 | 16 | 244 | 1 | 24.40 |
| 2502 | 0 | JP | G02B | G02B0006 | 25 | 16 | 136 | 1 | 68.00 |
| 2503 | 0 | JP | H01L | H01L0029 | 27 | 31 | 3 | 1.5 | 1.00 |
| 2504 | 0 | JP | H01L | H01L0033 | 1 | 11 | 2 | 1 | 1.00 |
| 2505 | 0 | JP | H01L | H01L0021 | 16 | 6 | 52 | 1 | 17.33 |
| 2506 | 0 | US | H01L | H01L0021 | 23 | 31 | 39 | 1 | 13.00 |
| 2507 | 0 | JP | H01L | H01L0033 | 2 | 12 | 6 | 1 | 2.00 |
| 2508 | 0 | JP | C30B | C30B0025 | 7 | 6 | 7 | 1 | 3.50 |
| 2509 | 0 | JP | H01L | H01L0023 | 5 | 2 | 3 | 3 | 0.20 |
| 2510 | 0 | JP | G02B | G02B0003 | 4 | 7 | 23 | 1 | 3.83 |
| 2511 | 0 | JP | H01L | H01L0033 | 17 | 8 | 57 | 1 | 14.25 |
| 2512 | 0 | JP | H01L | H01L0033 | 3 | 7 | 2 | 1 | 0.50 |
| 2513 | 0 | JP | H01L | H01L0027 | 10 | 9 | 55 | 2.5 | 11.00 |
| 2514 | 0 | US | H01L | H01L0033 | 13 | 6 | 7 | 1.285714286 | 0.78 |
| 2515 | 0 | JP | H01L | H01L0021 | 7 | 6 | 2 | 1 | 0.67 |
| 2516 | 0 | JP | C23C | C23C0014 | 10 | 18 | 15 | 1.5 | 5.00 |
| 2517 | 0 | US | H01L | H01L0021 | 39 | 13 | 15 | 1.5 | 2.50 |
| 2518 | 0 | JP | H01S | H01S0005 | 9 | 9 | 2 | 5.5 | 0.18 |
| 2519 | 0 | JP | G02B | G02B0006 | 6 | 15 | 83 | 2 | 20.75 |
| 2520 | 0 | US | C30B | C30B0023 | 22 | 6 | 34 | 1 | 4.25 |
| 2521 | 0 | JP | H01L | H01L0033 | 10 | 45 | 29 | 1 | 9.67 |
| 2522 | 0 | US | H01L | H01L0025 | 6 | 9 | 33 | 1.090909091 | 2.75 |
| 2523 | 0 | US | H01L | H01L0033 | 53 | 11 | 121 | 1 | 40.33 |
| 2524 | 0 | JP | F21V | F21V0008 | 20 | 19 | 53 | 1 | 13.25 |
| 2525 | 0 | JP | H01L | H01L0027 | 6 | 7 | 5 | 1.5 | 1.67 |
| 2526 | 0 | US | C30B | C30B0019 | 10 | 8 | 10 | 2 | 5.00 |
| 2527 | 0 | US | H01L | H01L0029 | 10 | 6 | 26 | 1 | 13.00 |
| 2528 | 0 | DE | H01L | H01L0029 | 16 | 11 | 1 | 1 | 0.25 |
| 2529 | 0 | JP | C30B | C30B0025 | 12 | 6 | 3 | 1 | 1.50 |
| 2530 | 0 | JP | H01L | H01L0021 | 6 | 17 | 7 | 1.666666667 | 1.40 |
| 2531 | 0 | JP | H01L | H01L0021 | 19 | 16 | 13 | 1 | 4.33 |
| 2532 | 0 | JP | C30B | C30B0025 | 15 | 2 | 4 | 1 | 2.00 |
| 2533 | 0 | JP | H01L | H01L0021 | 24 | 17 | 22 | 3.5 | 3.14 |
| 2534 | 0 | JP | H01L | H01L0033 | 37 | 11 | 11 | 1 | 5.50 |
| 2535 | 0 | DE | H01L | H01L0021 | 16 | 12 | 44 | 1 | 14.67 |
| 2536 | 0 | JP | H01L | H01L0029 | 18 | 12 | 3 | 1.666666667 | 0.60 |
| 2537 | 0 | US | H01L | H01L0033 | 3 | 2 | 40 | 1 | 6.67 |
| 2538 | 0 | JP | H01L | H01L0033 | 6 | 11 | 3 | 1 | 1.00 |
| 2539 | 0 | JP | H01L | H01L0033 | 20 | 9 | 94 | 1.5 | 31.33 |
| 2540 | 0 | JP | C30B | C30B0025 | 13 | 20 | 51 | 1.5 | 8.50 |
| 2541 | 0 | JP | C23C | C23C0016 | 20 | 2 | 5 | 1.25 | 1.00 |
| 2542 | 0 | US | H01L | H01L0029 | 5 | 7 | 17 | 1 | 8.50 |
| 2543 | 0 | US | H01L | H01L0033 | 3 | 14 | 4 | 1.285714286 | 0.44 |
| 2544 | 0 | JP | H01L | H01L0021 | 17 | 22 | 16 | 1.5 | 5.33 |
| 2545 | 0 | JP | H01L | H01L0029 | 15 | 7 | 6 | 1.5 | 2.00 |
| 2546 | 0 | FR | H01L | H01L0021 | 3 | 10 | 18 | 1 | 4.50 |
| 2547 | 0 | JP | H01L | H01L0021 | 9 | 6 | 49 | 1.5 | 16.33 |
| 2548 | 0 | JP | H01L | H01L0021 | 17 | 4 | 62 | 1 | 31.00 |
| 2549 | 0 | US | C08G | C08G0059 | 44 | 17 | 61 | 1.071428571 | 4.07 |
| 2550 | 0 | JP | H01L | H01L0021 | 7 | 21 | 0 | 1 | 0.00 |
| 2551 | 0 | JP | H01L | H01L0033 | 10 | 5 | 1 | 1.5 | 0.33 |
| 2552 | 0 | JP | H01L | H01L0033 | 7 | 9 | 21 | 1 | 5.25 |
| 2553 | 0 | US | H01L | H01L0029 | 16 | 11 | 9 | 1 | 1.80 |
| 2554 | 0 | JP | H01L | H01L0021 | 8 | 24 | 12 | 1 | 6.00 |
| 2555 | 0 | JP | H01L | H01L0021 | 18 | 42 | 53 | 3.25 | 4.08 |
| 2556 | 0 | JP | H01L | H01L0021 | 1 | 5 | 6 | 1 | 1.50 |
| 2557 | 0 | US | H01L | H01L0021 | 11 | 9 | 9 | 1 | 4.50 |
| 2558 | 0 | JP | H01L | H01L0023 | 8 | 25 | 2 | 3 | 0.13 |
| 2559 | 0 | US | F21K | F21K0099 | 8 | 12 | 16 | 1 | 8.00 |
| 2560 | 0 | US | H01L | H01L0029 | 7 | 19 | 15 | 1 | 5.00 |
| 2561 | 0 | US | H01L | H01L0027 | 10 | 5 | 21 | 1.5 | 3.50 |
| 2562 | 0 | US | G02B | G02B0006 | 9 | 6 | 37 | 1 | 7.40 |
| 2563 | 0 | JP | H01L | H01L0033 | 8 | 15 | 13 | 1.333333333 | 3.25 |
| 2564 | 0 | KR | H01L | H01L0021 | 6 | 7 | 2 | 1 | 1.00 |
| 2565 | 0 | JP | H01L | H01L0021 | 5 | 14 | 1 | 1 | 0.33 |
| 2566 | 0 | JP | H01L | H01L0021 | 8 | 8 | 7 | 1.5 | 2.33 |
| 2567 | 0 | JP | C23C | C23C0016 | 13 | 11 | 22 | 1.5 | 7.33 |
| 2568 | 0 | US | H01L | H01L0033 | 17 | 5 | 23 | 1 | 11.50 |
| 2569 | 0 | JP | H01L | H01L0033 | 3 | 9 | 2 | 1 | 0.50 |
| 2570 | 0 | JP | H01L | H01L0027 | 10 | 7 | 13 | 2 | 3.25 |
| 2571 | 0 | JP | H01L | H01L0021 | 3 | 19 | 52 | 1.25 | 10.40 |
| 2572 | 0 | US | H01L | H01L0021 | 19 | 3 | 38 | 1 | 19.00 |
| 2573 | 0 | US | H01L | H01L0027 | 9 | 24 | 15 | 1.5 | 2.50 |
| 2574 | 0 | JP | C30B | C30B0025 | 6 | 13 | 10 | 1.25 | 2.00 |
| 2575 | 0 | US | H01L | H01L0033 | 7 | 10 | 1 | 2 | 0.50 |
| 2576 | 0 | JP | H01L | H01L0021 | 52 | 55 | 74 | 1 | 18.50 |
| 2577 | 0 | JP | H01L | H01L0033 | 18 | 9 | 32 | 1 | 10.67 |
| 2578 | 1 | US | H01L | H01L0021 | 12 | 3 | 46 | 1.625 | 3.54 |
| 2579 | 0 | US | H01L | H01L0021 | 11 | 11 | 39 | 1 | 9.75 |
| 2580 | 0 | JP | H01L | H01L0033 | 8 | 11 | 46 | 1.75 | 6.57 |
| 2581 | 0 | US | C23C | C23C0014 | 10 | 14 | 43 | 1 | 8.60 |
| 2582 | 0 | US | H01L | H01L0021 | 12 | 4 | 6 | 1.166666667 | 0.86 |
| 2583 | 0 | DE | H01L | H01L0031 | 5 | 39 | 7 | 1.25 | 1.40 |
| 2584 | 0 | JP | H01L | H01L0029 | 5 | 6 | 9 | 1 | 4.50 |
| 2585 | 0 | US | H01L | H01L0033 | 3 | 12 | 6 | 2 | 3.00 |
| 2586 | 0 | JP | H01L | H01L0023 | 10 | 5 | 22 | 1.2 | 3.67 |
| 2587 | 0 | JP | H01L | H01L0021 | 16 | 5 | 7 | 1 | 2.33 |
| 2588 | 0 | JP | G02B | G02B0006 | 7 | 13 | 38 | 1.5 | 12.67 |
| 2589 | 0 | FR | C23C | C23C0014 | 26 | 5 | 8 | 1.333333333 | 2.00 |
| 2590 | 0 | US | H01L | H01L0021 | 5 | 22 | 30 | 1.5 | 10.00 |
| 2591 | 0 | JP | H01L | H01L0021 | 4 | 35 | 154 | 1.666666667 | 30.80 |
| 2592 | 0 | US | C30B | C30B0025 | 12 | 16 | 21 | 1.8 | 2.33 |
| 2593 | 0 | JP | H01L | H01L0033 | 24 | 8 | 37 | 2 | 9.25 |
| 2594 | 0 | JP | H01L | H01L0033 | 10 | 11 | 3 | 1 | 1.50 |
| 2595 | 0 | JP | H01L | H01L0033 | 10 | 14 | 9 | 1.2 | 1.50 |
| 2596 | 0 | US | H01L | H01L0029 | 9 | 25 | 21 | 1.25 | 4.20 |
| 2597 | 0 | US | H01L | H01L0033 | 12 | 4 | 16 | 1.5 | 5.33 |
| 2598 | 0 | US | C23C | C23C0016 | 16 | 17 | 22 | 1 | 2.44 |
| 2599 | 0 | JP | H01L | H01L0025 | 12 | 24 | 31 | 1.333333333 | 7.75 |
| 2600 | 0 | JP | H01L | H01L0033 | 12 | 3 | 88 | 1 | 29.33 |
| 2601 | 0 | JP | C30B | C30B0023 | 19 | 24 | 38 | 1 | 9.50 |
| 2602 | 0 | JP | H01L | H01L0021 | 45 | 13 | 49 | 1 | 12.25 |
| 2603 | 0 | JP | C09K | C09K0011 | 17 | 10 | 16 | 1 | 8.00 |
| 2604 | 0 | US | C07C | C07C0391 | 43 | 18 | 30 | 1.25 | 3.00 |
| 2605 | 0 | JP | H01L | H01L0033 | 4 | 9 | 20 | 1 | 6.67 |
| 2606 | 0 | US | H01L | H01L0033 | 9 | 6 | 4 | 1.5 | 1.33 |
| 2607 | 0 | JP | H01L | H01L0021 | 5 | 10 | 3 | 1 | 0.75 |
| 2608 | 0 | US | H01L | H01L0023 | 18 | 9 | 165 | 1 | 27.50 |
| 2609 | 0 | JP | G01N | G01N0021 | 2 | 9 | 0 | 1 | 0.00 |
| 2610 | 0 | US | H01L | H01L0033 | 21 | 17 | 21 | 1.125 | 2.33 |
| 2611 | 0 | US | H01L | H01L0021 | 6 | 13 | 21 | 1 | 5.25 |
| 2612 | 0 | US | H01S | H01S0005 | 58 | 37 | 30 | 1 | 7.50 |
| 2613 | 0 | US | H01L | H01L0029 | 6 | 15 | 12 | 1 | 3.00 |
| 2614 | 0 | US | C30B | C30B0025 | 10 | 5 | 10 | 2 | 5.00 |
| 2615 | 0 | JP | H01L | H01L0021 | 30 | 14 | 13 | 1 | 3.25 |
| 2616 | 0 | JP | H01L | H01L0021 | 17 | 4 | 10 | 1 | 5.00 |
| 2617 | 0 | JP | H01L | H01L0021 | 25 | 3 | 23 | 1 | 11.50 |
| 2618 | 0 | US | G02B | G02B0001 | 16 | 1 | 33 | 3 | 11.00 |
| 2619 | 0 | JP | C08G | C08G0059 | 18 | 4 | 10 | 5.666666667 | 0.59 |
| 2620 | 0 | US | C23C | C23C0016 | 25 | 44 | 74 | 1.25 | 14.80 |
| 2621 | 0 | DE | C30B | C30B0019 | 3 | 12 | 1 | 1 | 0.20 |
| 2622 | 0 | JP | C04B | C04B0035 | 9 | 10 | 74 | 1.5 | 24.67 |
| 2623 | 0 | JP | H01L | H01L0021 | 5 | 8 | 14 | 1 | 7.00 |
| 2624 | 0 | US | C30B | C30B0025 | 59 | 10 | 15 | 1 | 2.50 |
| 2625 | 0 | JP | H01L | H01L0033 | 4 | 1 | 0 | 1.25 | 0.00 |
| 2626 | 0 | JP | H01L | H01L0021 | 19 | 4 | 4 | 1.333333333 | 1.00 |
| 2627 | 0 | US | C08J | C08J0003 | 21 | 8 | 2 | 2 | 1.00 |
| 2628 | 0 | JP | C30B | C30B0029 | 14 | 6 | 4 | 1 | 1.00 |
| 2629 | 0 | FR | G02F | G02F0001 | 13 | 9 | 6 | 1 | 2.00 |
| 2630 | 0 | JP | H01L | H01L0029 | 14 | 14 | 12 | 1 | 4.00 |
| 2631 | 0 | JP | H01B | H01B0001 | 1 | 9 | 1 | 2.5 | 0.20 |
| 2632 | 0 | US | H01L | H01L0033 | 36 | 20 | 270 | 1 | 27.00 |
| 2633 | 0 | JP | C30B | C30B0025 | 10 | 9 | 26 | 1.25 | 5.20 |
| 2634 | 0 | DE | C30B | C30B0025 | 14 | 10 | 17 | 2.25 | 1.89 |
| 2635 | 0 | US | H01L | H01L0021 | 12 | 17 | 21 | 1.5 | 7.00 |
| 2636 | 0 | JP | H01L | H01L0033 | 4 | 6 | 5 | 1 | 1.25 |
| 2637 | 0 | JP | C23C | C23C0016 | 11 | 30 | 5 | 1 | 1.67 |
| 2638 | 0 | US | H01L | H01L0033 | 7 | 5 | 19 | 1.5 | 3.17 |
| 2639 | 0 | US | H01L | H01L0021 | 8 | 4 | 2 | 1.333333333 | 0.50 |
| 2640 | 0 | GB | C08J | C08J0003 | 20 | 20 | 23 | 1.222222222 | 2.09 |
| 2641 | 0 | JP | H01L | H01L0033 | 6 | 4 | 5 | 1.333333333 | 1.25 |
| 2642 | 0 | JP | H01L | H01L0021 | 11 | 10 | 81 | 1 | 20.25 |
| 2643 | 0 | JP | H01L | H01L0033 | 1 | 22 | 46 | 1.5 | 7.67 |
| 2644 | 0 | JP | C30B | C30B0023 | 3 | 8 | 1 | 1 | 0.50 |
| 2645 | 0 | JP | G11B | G11B0007 | 5 | 10 | 9 | 1 | 2.25 |
| 2646 | 0 | US | H01L | H01L0021 | 10 | 7 | 4 | 1 | 0.80 |
| 2647 | 0 | JP | H01L | H01L0021 | 12 | 15 | 17 | 1.333333333 | 4.25 |
| 2648 | 0 | JP | C30B | C30B0025 | 5 | 12 | 1 | 1 | 0.20 |
| 2649 | 0 | JP | G02F | G02F0001 | 4 | 6 | 16 | 1.25 | 3.20 |
| 2650 | 0 | US | C23C | C23C0016 | 44 | 20 | 19 | 1 | 3.80 |
| 2651 | 0 | US | H01L | H01L0033 | 12 | 6 | 5 | 1.142857143 | 0.63 |
| 2652 | 0 | US | C30B | C30B0029 | 3 | 19 | 0 | 1.6 | 0.00 |
| 2653 | 0 | JP | H01L | H01L0033 | 6 | 10 | 8 | 2.5 | 0.80 |
| 2654 | 0 | JP | H01L | H01L0021 | 8 | 16 | 2 | 1.5 | 0.67 |
| 2655 | 0 | US | C09J | C09J0005 | 3 | 14 | 2 | 1 | 0.33 |
| 2656 | 0 | JP | H01L | H01L0021 | 12 | 12 | 3 | 1.5 | 1.00 |
| 2657 | 0 | JP | H01L | H01L0033 | 12 | 23 | 7 | 1 | 3.50 |
| 2658 | 0 | US | C30B | C30B0025 | 12 | 10 | 32 | 1.625 | 2.46 |
| 2659 | 0 | US | C23C | C23C0016 | 12 | 8 | 191 | 3.75 | 6.37 |
| 2660 | 0 | US | H01L | H01L0021 | 36 | 24 | 84 | 1.333333333 | 21.00 |
| 2661 | 0 | JP | C30B | C30B0011 | 10 | 10 | 5 | 1 | 1.67 |
| 2662 | 0 | US | H01L | H01L0021 | 15 | 9 | 41 | 1 | 8.20 |
| 2663 | 0 | US | H01L | H01L0021 | 11 | 6 | 12 | 1.5 | 2.00 |
| 2664 | 0 | JP | H01L | H01L0027 | 3 | 16 | 11 | 1 | 5.50 |
| 2665 | 0 | JP | H01L | H01L0033 | 6 | 3 | 65 | 1.5 | 21.67 |
| 2666 | 0 | FR | G02F | G02F0001 | 7 | 15 | 31 | 1 | 10.33 |
| 2667 | 0 | JP | G02B | G02B0006 | 16 | 3 | 27 | 2 | 6.75 |
| 2668 | 0 | JP | H01L | H01L0021 | 49 | 13 | 163 | 7.714285714 | 3.02 |
| 2669 | 0 | JP | G09F | G09F0009 | 6 | 7 | 38 | 1 | 19.00 |
| 2670 | 0 | GB | H01L | H01L0021 | 39 | 17 | 17 | 1 | 2.83 |
| 2671 | 0 | JP | H01L | H01L0033 | 14 | 15 | 3 | 1 | 0.75 |
| 2672 | 0 | JP | H01L | H01L0023 | 23 | 1 | 26 | 1 | 13.00 |
| 2673 | 0 | KR | H01L | H01L0027 | 11 | 4 | 39 | 1 | 13.00 |
| 2674 | 0 | JP | H01L | H01L0021 | 1 | 9 | 3 | 1 | 1.00 |
| 2675 | 0 | JP | H01L | H01L0021 | 7 | 24 | 2 | 1.25 | 0.40 |
| 2676 | 0 | JP | H01L | H01L0033 | 2 | 11 | 8 | 3 | 1.33 |
| 2677 | 0 | JP | G02B | G02B0006 | 11 | 13 | 16 | 1 | 2.67 |
| 2678 | 0 | JP | C30B | C30B0025 | 12 | 18 | 8 | 1.5 | 2.67 |
| 2679 | 0 | JP | H01L | H01L0021 | 39 | 13 | 175 | 4.6 | 7.61 |
| 2680 | 0 | JP | C30B | C30B0025 | 15 | 16 | 16 | 1.6 | 2.00 |
| 2681 | 0 | JP | G03C | G03C0001 | 20 | 11 | 1 | 1.25 | 0.20 |
| 2682 | 0 | GB | C09D | C09D0005 | 10 | 17 | 17 | 2.2 | 1.55 |
| 2683 | 0 | JP | H01L | H01L0021 | 12 | 11 | 2 | 1.25 | 0.40 |
| 2684 | 0 | JP | H01L | H01L0021 | 12 | 22 | 11 | 1 | 3.67 |
| 2685 | 0 | JP | H01L | H01L0021 | 13 | 3 | 19 | 4 | 1.58 |
| 2686 | 0 | US | H01L | H01L0021 | 71 | 14 | 12 | 3 | 4.00 |
| 2687 | 0 | JP | H01L | H01L0021 | 7 | 7 | 2 | 1 | 0.67 |
| 2688 | 0 | US | H01L | H01L0033 | 38 | 26 | 102 | 1.5 | 34.00 |
| 2689 | 0 | JP | H01L | H01L0029 | 19 | 5 | 32 | 1 | 16.00 |
| 2690 | 0 | JP | G02B | G02B0006 | 6 | 54 | 18 | 1.25 | 3.60 |
| 2691 | 0 | JP | C30B | C30B0025 | 10 | 14 | 56 | 1.5 | 18.67 |
| 2692 | 0 | JP | H01L | H01L0033 | 1 | 11 | 35 | 1 | 17.50 |
| 2693 | 0 | JP | H01L | H01L0029 | 6 | 9 | 9 | 1.25 | 1.80 |
| 2694 | 1 | US | H01L | H01L0021 | 51 | 16 | 21 | 5 | 2.10 |
| 2695 | 0 | JP | C23C | C23C0016 | 2 | 31 | 32 | 1.5 | 5.33 |
| 2696 | 0 | US | H01L | H01L0029 | 7 | 3 | 5 | 2 | 2.50 |
| 2697 | 0 | US | H01L | H01L0023 | 30 | 8 | 27 | 1 | 6.75 |
| 2698 | 0 | JP | H01L | H01L0021 | 8 | 9 | 79 | 1 | 19.75 |
| 2699 | 0 | JP | H01L | H01L0027 | 4 | 13 | 24 | 2 | 6.00 |
| 2700 | 0 | JP | H01L | H01L0021 | 3 | 5 | 25 | 1 | 6.25 |
| 2701 | 0 | JP | H01L | H01L0021 | 6 | 21 | 18 | 1 | 4.50 |
| 2702 | 0 | JP | H01L | H01L0021 | 8 | 7 | 8 | 1 | 2.00 |
| 2703 | 0 | JP | H01L | H01L0021 | 15 | 17 | 4 | 1.333333333 | 1.00 |
| 2704 | 0 | JP | H01L | H01L0021 | 24 | 8 | 111 | 1 | 22.20 |
| 2705 | 0 | JP | C30B | C30B0019 | 8 | 8 | 0 | 1.2 | 0.00 |
| 2706 | 0 | JP | C30B | C30B0025 | 8 | 8 | 2 | 1.25 | 0.40 |
| 2707 | 0 | US | B41J | B41J0002 | 5 | 31 | 6 | 1 | 2.00 |
| 2708 | 0 | US | H01L | H01L0021 | 12 | 23 | 15 | 2 | 7.50 |
| 2709 | 0 | JP | C30B | C30B0015 | 20 | 6 | 3 | 2 | 0.75 |
| 2710 | 0 | US | G09F | G09F0009 | 8 | 4 | 47 | 1.2 | 7.83 |
| 2711 | 0 | US | G02B | G02B0001 | 11 | 7 | 25 | 3 | 8.33 |
| 2712 | 0 | JP | H01L | H01L0023 | 4 | 15 | 20 | 1.2 | 3.33 |
| 2713 | 0 | JP | H01L | H01L0021 | 10 | 8 | 2 | 1 | 1.00 |
| 2714 | 0 | JP | C30B | C30B0025 | 8 | 9 | 60 | 1 | 30.00 |
| 2715 | 0 | JP | H01L | H01L0033 | 5 | 20 | 7 | 1.25 | 1.40 |
| 2716 | 0 | JP | H01L | H01L0033 | 11 | 9 | 13 | 1.25 | 2.60 |
| 2717 | 0 | JP | C30B | C30B0025 | 12 | 12 | 63 | 1 | 21.00 |
| 2718 | 0 | JP | G02F | G02F0001 | 11 | 14 | 5 | 1.666666667 | 1.00 |
| 2719 | 0 | JP | C30B | C30B0025 | 11 | 10 | 4 | 1 | 1.00 |
| 2720 | 0 | JP | H01L | H01L0021 | 8 | 17 | 4 | 1 | 1.33 |
| 2721 | 0 | US | H01L | H01L0021 | 6 | 7 | 6 | 1 | 2.00 |
| 2722 | 0 | US | C23F | C23F0001 | 14 | 8 | 7 | 1 | 2.33 |
| 2723 | 0 | JP | H01L | H01L0021 | 17 | 12 | 20 | 1 | 10.00 |
| 2724 | 0 | JP | H01S | H01S0005 | 5 | 11 | 46 | 1 | 23.00 |
| 2725 | 0 | JP | H01L | H01L0029 | 20 | 17 | 20 | 1.5 | 6.67 |
| 2726 | 0 | US | H01L | H01L0021 | 30 | 14 | 166 | 1.111111111 | 16.60 |
| 2727 | 0 | JP | H01L | H01L0031 | 7 | 7 | 47 | 1 | 23.50 |
| 2728 | 0 | JP | H01L | H01L0029 | 9 | 16 | 6 | 1 | 2.00 |
| 2729 | 0 | JP | C30B | C30B0025 | 7 | 5 | 10 | 1 | 5.00 |
| 2730 | 0 | US | H01L | H01L0029 | 3 | 9 | 7 | 2 | 3.50 |
| 2731 | 0 | JP | H01L | H01L0033 | 16 | 5 | 5 | 1 | 1.25 |
| 2732 | 0 | JP | H01S | H01S0005 | 24 | 11 | 18 | 1.25 | 3.60 |
| 2733 | 0 | US | H01L | H01L0021 | 8 | 14 | 68 | 1.333333333 | 17.00 |
| 2734 | 0 | PL | C30B | C30B0029 | 29 | 17 | 44 | 1 | 4.00 |
| 2735 | 0 | JP | H01L | H01L0021 | 19 | 8 | 4 | 1.333333333 | 1.00 |
| 2736 | 0 | JP | H01L | H01L0033 | 2 | 22 | 7 | 1.2 | 1.17 |
| 2737 | 0 | JP | H05B | H05B0033 | 20 | 18 | 14 | 1.25 | 2.80 |
| 2738 | 0 | US | G02F | G02F0001 | 7 | 16 | 31 | 1 | 10.33 |
| 2739 | 0 | JP | C08G | C08G0059 | 8 | 13 | 1 | 1 | 0.33 |
| 2740 | 0 | JP | C30B | C30B0019 | 6 | 8 | 1 | 1.25 | 0.20 |
| 2741 | 0 | JP | H01L | H01L0033 | 2 | 10 | 0 | 1 | 0.00 |
| 2742 | 0 | JP | H05B | H05B0033 | 9 | 11 | 32 | 1 | 16.00 |
| 2743 | 0 | JP | H01L | H01L0021 | 4 | 8 | 4 | 1 | 1.00 |
| 2744 | 0 | US | H01L | H01L0027 | 14 | 10 | 23 | 2 | 11.50 |
| 2745 | 0 | US | H05K | H05K0001 | 10 | 2 | 61 | 1 | 15.25 |
| 2746 | 0 | JP | H01L | H01L0033 | 28 | 3 | 35 | 2.5 | 7.00 |
| 2747 | 0 | US | H01L | H01L0021 | 23 | 8 | 8 | 1 | 2.00 |
| 2748 | 0 | JP | H01L | H01L0033 | 20 | 25 | 1 | 2 | 0.25 |
| 2749 | 0 | JP | H01L | H01L0033 | 26 | 9 | 43 | 1 | 21.50 |
| 2750 | 0 | JP | C30B | C30B0025 | 6 | 7 | 3 | 1.2 | 0.50 |
| 2751 | 0 | JP | H01S | H01S0005 | 18 | 8 | 10 | 1 | 2.50 |
| 2752 | 0 | JP | H01S | H01S0005 | 69 | 11 | 9 | 1 | 3.00 |
| 2753 | 0 | JP | H01L | H01L0021 | 12 | 18 | 59 | 3 | 9.83 |
| 2754 | 0 | DE | C30B | C30B0025 | 13 | 10 | 16 | 2.25 | 1.78 |
| 2755 | 0 | JP | H01L | H01L0033 | 25 | 9 | 47 | 1 | 11.75 |
| 2756 | 0 | JP | H01L | H01L0029 | 12 | 8 | 86 | 3 | 14.33 |
| 2757 | 0 | JP | G02F | G02F0001 | 2 | 20 | 9 | 1 | 1.80 |
| 2758 | 0 | US | H01L | H01L0023 | 15 | 19 | 49 | 1 | 16.33 |
| 2759 | 0 | US | H01L | H01L0033 | 2 | 5 | 5 | 1.333333333 | 1.25 |
| 2760 | 0 | US | C23C | C23C0016 | 20 | 3 | 15 | 1.666666667 | 3.00 |
| 2761 | 0 | TW | H01L | H01L0033 | 16 | 4 | 62 | 1 | 31.00 |
| 2762 | 0 | US | H01L | H01L0021 | 20 | 13 | 22 | 1 | 5.50 |
| 2763 | 0 | US | H01L | H01L0033 | 5 | 27 | 24 | 2 | 12.00 |
| 2764 | 0 | US | H01L | H01L0021 | 12 | 13 | 10 | 1.333333333 | 2.50 |
| 2765 | 0 | JP | H01L | H01L0021 | 6 | 23 | 5 | 1.125 | 0.56 |
| 2766 | 0 | JP | H01L | H01L0033 | 7 | 7 | 7 | 1 | 3.50 |
| 2767 | 0 | JP | H01L | H01L0033 | 20 | 11 | 19 | 1.4 | 2.71 |
| 2768 | 0 | JP | C09K | C09K0011 | 13 | 16 | 8 | 1 | 4.00 |
| 2769 | 0 | US | H01L | H01L0029 | 18 | 7 | 5 | 1.166666667 | 0.71 |
| 2770 | 0 | JP | H01L | H01L0029 | 19 | 15 | 30 | 1.25 | 6.00 |
| 2771 | 0 | JP | H01L | H01L0029 | 10 | 8 | 2 | 1 | 1.00 |
| 2772 | 0 | JP | H01L | H01L0033 | 3 | 9 | 28 | 2.666666667 | 3.50 |
| 2773 | 0 | JP | H01L | H01L0029 | 27 | 8 | 31 | 1 | 15.50 |
| 2774 | 0 | JP | H01L | H01L0021 | 3 | 18 | 53 | 1 | 13.25 |
| 2775 | 0 | JP | H01L | H01L0029 | 5 | 9 | 3 | 1 | 0.60 |
| 2776 | 0 | JP | H01L | H01L0033 | 9 | 11 | 8 | 1 | 4.00 |
| 2777 | 0 | US | C30B | C30B0019 | 32 | 28 | 55 | 1.1 | 5.00 |
| 2778 | 0 | JP | H01L | H01L0029 | 22 | 17 | 9 | 1 | 2.25 |
| 2779 | 0 | US | H01L | H01L0021 | 12 | 4 | 19 | 1 | 6.33 |
| 2780 | 0 | JP | H01L | H01L0029 | 22 | 10 | 17 | 1 | 5.67 |
| 2781 | 0 | US | H01L | H01L0021 | 4 | 13 | 2 | 2 | 1.00 |
| 2782 | 0 | US | H01L | H01L0033 | 3 | 10 | 52 | 2 | 26.00 |
| 2783 | 0 | US | G01R | G01R0001 | 13 | 6 | 159 | 12.71428571 | 1.79 |
| 2784 | 0 | US | C30B | C30B0025 | 10 | 9 | 4 | 1 | 1.00 |
| 2785 | 0 | US | H01L | H01L0021 | 5 | 25 | 18 | 2 | 9.00 |
| 2786 | 0 | US | C30B | C30B0025 | 12 | 19 | 23 | 1.625 | 1.77 |
| 2787 | 0 | JP | G02B | G02B0006 | 18 | 56 | 23 | 1.25 | 4.60 |
| 2788 | 0 | JP | H01L | H01L0021 | 11 | 16 | 8 | 1.25 | 1.60 |
| 2789 | 0 | JP | H01L | H01L0033 | 20 | 10 | 42 | 2.5 | 8.40 |
| 2790 | 0 | US | H01L | H01L0021 | 2 | 13 | 15 | 1 | 3.00 |
| 2791 | 0 | JP | H01L | H01L0027 | 21 | 8 | 90 | 1 | 45.00 |
| 2792 | 0 | US | C23C | C23C0016 | 19 | 14 | 497 | 3.75 | 16.57 |
| 2793 | 0 | JP | H01L | H01L0033 | 10 | 8 | 34 | 1 | 17.00 |
| 2794 | 0 | FR | G02F | G02F0001 | 2 | 25 | 0 | 1 | 0.00 |
| 2795 | 0 | US | H01L | H01L0021 | 16 | 17 | 16 | 1 | 2.67 |
| 2796 | 0 | US | H01L | H01L0021 | 9 | 8 | 2 | 2.8 | 0.14 |
| 2797 | 0 | JP | C30B | C30B0025 | 7 | 4 | 1 | 1 | 0.25 |
| 2798 | 0 | JP | H01L | H01L0033 | 10 | 7 | 9 | 1.5 | 3.00 |
| 2799 | 0 | JP | H01L | H01L0033 | 37 | 25 | 39 | 2 | 9.75 |
| 2800 | 0 | JP | C30B | C30B0023 | 4 | 6 | 10 | 1 | 5.00 |
| 2801 | 0 | US | B32B | B32B0018 | 31 | 25 | 11 | 1.833333333 | 1.00 |
| 2802 | 0 | JP | H01L | H01L0033 | 3 | 13 | 5 | 2 | 1.25 |
| 2803 | 0 | US | H01L | H01L0021 | 17 | 19 | 26 | 2 | 13.00 |
| 2804 | 0 | US | H01L | H01L0021 | 20 | 11 | 19 | 1 | 9.50 |
| 2805 | 0 | US | C30B | C30B0025 | 35 | 18 | 20 | 1 | 4.00 |
| 2806 | 0 | US | H01L | H01L0021 | 5 | 25 | 9 | 2 | 1.13 |
| 2807 | 0 | TW | H01L | H01L0031 | 3 | 5 | 7 | 2 | 3.50 |
| 2808 | 0 | VG | G03F | G03F0007 | 16 | 10 | 2 | 1.125 | 0.22 |
| 2809 | 0 | JP | H01L | H01L0027 | 9 | 5 | 3 | 1 | 1.50 |
| 2810 | 0 | JP | H01L | H01L0029 | 7 | 15 | 23 | 1 | 11.50 |
| 2811 | 0 | JP | C30B | C30B0029 | 16 | 7 | 0 | 1 | 0.00 |
| 2812 | 0 | US | H01L | H01L0021 | 16 | 16 | 3 | 1 | 0.60 |
| 2813 | 0 | JP | H01L | H01L0033 | 9 | 21 | 15 | 1 | 3.00 |
| 2814 | 0 | US | H01L | H01L0027 | 15 | 9 | 4 | 1 | 2.00 |
| 2815 | 0 | US | G02F | G02F0001 | 9 | 14 | 31 | 1 | 7.75 |
| 2816 | 0 | JP | C23C | C23C0016 | 1 | 18 | 106 | 1.333333333 | 13.25 |
| 2817 | 0 | US | H01L | H01L0027 | 17 | 10 | 24 | 1 | 12.00 |
| 2818 | 0 | JP | H01L | H01L0033 | 43 | 11 | 29 | 1.5 | 9.67 |
| 2819 | 0 | JP | C30B | C30B0023 | 22 | 43 | 12 | 1.5 | 1.00 |
| 2820 | 0 | JP | H01L | H01L0033 | 2 | 71 | 31 | 3.4 | 1.82 |
| 2821 | 0 | US | H01L | H01L0021 | 15 | 37 | 15 | 1.25 | 3.00 |
| 2822 | 0 | JP | H01L | H01L0033 | 9 | 22 | 42 | 4.6 | 1.83 |
| 2823 | 0 | US | H01L | H01L0021 | 11 | 11 | 13 | 1.333333333 | 3.25 |
| 2824 | 0 | JP | H01L | H01L0033 | 16 | 6 | 8 | 1.333333333 | 2.00 |
| 2825 | 0 | US | H01L | H01L0033 | 42 | 74 | 230 | 1.125 | 25.56 |
| 2826 | 0 | JP | H01L | H01L0033 | 9 | 8 | 1 | 1.5 | 0.33 |
| 2827 | 0 | JP | C30B | C30B0025 | 14 | 6 | 3 | 1.5 | 1.00 |
| 2828 | 0 | US | H01L | H01L0021 | 17 | 30 | 50 | 1 | 7.14 |
| 2829 | 0 | US | C30B | C30B0023 | 14 | 9 | 10 | 1 | 5.00 |
| 2830 | 0 | US | H01L | H01L0027 | 11 | 7 | 20 | 2 | 10.00 |
| 2831 | 0 | JP | H01L | H01L0033 | 15 | 6 | 32 | 1 | 16.00 |
| 2832 | 0 | JP | H01L | H01L0033 | 12 | 12 | 33 | 1 | 16.50 |
| 2833 | 0 | JP | H01L | H01L0033 | 9 | 16 | 68 | 4.6 | 2.96 |
| 2834 | 0 | JP | H01L | H01L0021 | 19 | 11 | 2 | 1.25 | 0.40 |
| 2835 | 0 | KR | H01L | H01L0021 | 4 | 9 | 0 | 1 | 0.00 |
| 2836 | 0 | JP | H01L | H01L0021 | 69 | 16 | 41 | 2.666666667 | 1.71 |
| 2837 | 0 | JP | H01L | H01L0033 | 14 | 5 | 13 | 1.25 | 2.60 |
| 2838 | 0 | JP | H01L | H01L0033 | 5 | 7 | 43 | 1 | 21.50 |
| 2839 | 0 | JP | H01L | H01L0033 | 2 | 10 | 43 | 5.5 | 3.91 |
| 2840 | 0 | JP | H01S | H01S0005 | 14 | 3 | 23 | 1 | 11.50 |
| 2841 | 0 | JP | H01L | H01L0033 | 27 | 4 | 36 | 1 | 18.00 |
| 2842 | 0 | JP | C30B | C30B0023 | 18 | 9 | 10 | 1 | 5.00 |
| 2843 | 0 | JP | H01L | H01L0033 | 9 | 11 | 5 | 1.4 | 0.71 |
| 2844 | 0 | US | H01L | H01L0021 | 7 | 8 | 4 | 1.5 | 1.33 |
| 2845 | 0 | JP | H01L | H01L0021 | 3 | 5 | 3 | 1 | 0.75 |
| 2846 | 0 | JP | C30B | C30B0019 | 20 | 11 | 1 | 1.2 | 0.17 |
| 2847 | 0 | JP | G02B | G02B0005 | 24 | 30 | 56 | 1.166666667 | 8.00 |
| 2848 | 0 | US | H01L | H01L0021 | 12 | 22 | 4 | 1 | 2.00 |
| 2849 | 0 | JP | H01L | H01L0033 | 11 | 13 | 6 | 1 | 1.00 |
| 2850 | 0 | JP | H01L | H01L0033 | 24 | 7 | 33 | 1 | 16.50 |
| 2851 | 0 | JP | H01L | H01L0021 | 5 | 5 | 80 | 1 | 26.67 |
| 2852 | 0 | JP | C30B | C30B0025 | 9 | 10 | 11 | 2.5 | 2.20 |
| 2853 | 0 | JP | H01L | H01L0033 | 3 | 29 | 14 | 1 | 3.50 |
| 2854 | 0 | JP | H01L | H01L0021 | 55 | 10 | 2 | 1.666666667 | 0.40 |
| 2855 | 0 | JP | G02F | G02F0001 | 9 | 8 | 15 | 1 | 7.50 |
| 2856 | 0 | JP | H01L | H01L0021 | 11 | 53 | 2 | 1.5 | 0.33 |
| 2857 | 0 | JP | H01L | H01L0023 | 9 | 7 | 18 | 1.5 | 6.00 |
| 2858 | 0 | US | F21K | F21K0007 | 7 | 9 | 20 | 1 | 6.67 |
| 2859 | 0 | JP | C23C | C23C0016 | 6 | 18 | 15 | 1.5 | 5.00 |
| 2860 | 0 | US | H01L | H01L0021 | 5 | 13 | 9 | 1 | 4.50 |
| 2861 | 0 | JP | H01L | H01L0033 | 3 | 4 | 2 | 1 | 0.67 |
| 2862 | 0 | JP | H01L | H01L0033 | 21 | 8 | 169 | 5 | 8.45 |
| 2863 | 0 | JP | H01L | H01L0021 | 5 | 7 | 4 | 1 | 1.33 |
| 2864 | 0 | US | H01L | H01L0027 | 1 | 24 | 5 | 2 | 2.50 |
| 2865 | 0 | JP | H01L | H01L0021 | 18 | 5 | 6 | 1.333333333 | 1.50 |
| 2866 | 0 | JP | H01L | H01L0027 | 14 | 5 | 37 | 1 | 18.50 |
| 2867 | 0 | JP | H01L | H01L0033 | 5 | 7 | 36 | 1.5 | 12.00 |
| 2868 | 0 | US | H01L | H01L0021 | 12 | 4 | 55 | 1 | 13.75 |
| 2869 | 0 | JP | H01L | H01L0021 | 9 | 14 | 16 | 1.25 | 3.20 |
| 2870 | 0 | GB | F21S | F21S0004 | 8 | 3 | 4 | 1 | 2.00 |
| 2871 | 0 | US | C30B | C30B0025 | 10 | 21 | 8 | 1 | 2.67 |
| 2872 | 0 | JP | F21K | F21K0099 | 24 | 8 | 61 | 1.5 | 10.17 |
| 2873 | 0 | KR | G03F | G03F0007 | 3 | 3 | 1 | 1 | 0.33 |
| 2874 | 0 | JP | H01L | H01L0033 | 28 | 10 | 21 | 1 | 10.50 |
| 2875 | 0 | JP | H01L | H01L0033 | 10 | 8 | 8 | 1 | 4.00 |
| 2876 | 0 | US | H01J | H01J0037 | 21 | 2 | 27 | 1.25 | 5.40 |
| 2877 | 0 | JP | H01L | H01L0021 | 3 | 2 | 3 | 1 | 1.50 |
| 2878 | 0 | JP | H01L | H01L0033 | 9 | 7 | 3 | 1 | 1.00 |
| 2879 | 0 | JP | H01L | H01L0023 | 30 | 17 | 16 | 1.25 | 3.20 |
| 2880 | 0 | JP | H01L | H01L0021 | 4 | 35 | 9 | 1.333333333 | 2.25 |
| 2881 | 0 | JP | H01L | H01L0033 | 7 | 5 | 63 | 2 | 10.50 |
| 2882 | 0 | JP | H01L | H01L0033 | 13 | 9 | 83 | 1 | 41.50 |
| 2883 | 0 | JP | H01L | H01L0023 | 4 | 16 | 19 | 2 | 3.17 |
| 2884 | 0 | JP | H01L | H01L0029 | 12 | 9 | 41 | 2 | 10.25 |
| 2885 | 0 | JP | H01L | H01L0033 | 12 | 7 | 5 | 1 | 1.25 |
| 2886 | 0 | US | H01L | H01L0027 | 7 | 18 | 9 | 1 | 4.50 |
| 2887 | 0 | JP | H01L | H01L0021 | 14 | 10 | 11 | 1 | 5.50 |
| 2888 | 0 | JP | H01S | H01S0005 | 12 | 12 | 6 | 1 | 3.00 |
| 2889 | 0 | US | H01L | H01L0021 | 15 | 3 | 87 | 2 | 43.50 |
| 2890 | 0 | US | C30B | C30B0025 | 14 | 23 | 21 | 1.5 | 7.00 |
| 2891 | 0 | JP | H01J | H01J0001 | 7 | 10 | 13 | 1.5 | 2.17 |
| 2892 | 0 | JP | H01L | H01L0033 | 20 | 7 | 23 | 2.666666667 | 2.88 |
| 2893 | 0 | JP | C30B | C30B0025 | 9 | 12 | 65 | 1 | 21.67 |
| 2894 | 0 | JP | G03C | G03C0008 | 3 | 6 | 5 | 1 | 2.50 |
| 2895 | 0 | JP | H01L | H01L0021 | 17 | 5 | 7 | 2.5 | 1.40 |
| 2896 | 0 | JP | G03F | G03F0007 | 5 | 6 | 1 | 1 | 0.17 |
| 2897 | 0 | JP | H01L | H01L0033 | 4 | 4 | 15 | 1.5 | 5.00 |
| 2898 | 0 | JP | H01L | H01L0021 | 8 | 6 | 14 | 1.5 | 4.67 |
| 2899 | 0 | KR | H01L | H01L0031 | 11 | 10 | 7 | 1 | 3.50 |
| 2900 | 0 | JP | H01L | H01L0033 | 5 | 3 | 2 | 1.333333333 | 0.50 |
| 2901 | 0 | US | H01L | H01L0023 | 29 | 12 | 18 | 1.2 | 3.00 |
| 2902 | 0 | US | H01L | H01L0033 | 13 | 15 | 15 | 1.5 | 2.50 |
| 2903 | 0 | JP | H01L | H01L0033 | 13 | 15 | 21 | 1 | 10.50 |
| 2904 | 0 | JP | H01L | H01L0021 | 28 | 10 | 29 | 2 | 7.25 |
| 2905 | 0 | JP | H01L | H01L0033 | 6 | 8 | 57 | 5.5 | 5.18 |
| 2906 | 0 | JP | C30B | C30B0029 | 9 | 10 | 15 | 1 | 7.50 |
| 2907 | 0 | JP | H01L | H01L0029 | 7 | 9 | 11 | 1 | 3.67 |
| 2908 | 0 | JP | H01S | H01S0005 | 12 | 11 | 10 | 1.5 | 3.33 |
| 2909 | 0 | JP | H01L | H01L0033 | 5 | 8 | 0 | 1.25 | 0.00 |
| 2910 | 0 | US | H01L | H01L0021 | 7 | 12 | 2 | 2 | 1.00 |
| 2911 | 0 | US | B29C | B29C0045 | 5 | 21 | 12 | 1.25 | 2.40 |
| 2912 | 0 | JP | H01L | H01L0021 | 12 | 13 | 3 | 1 | 0.75 |
| 2913 | 0 | JP | H01L | H01L0033 | 17 | 12 | 7 | 1.333333333 | 1.75 |
| 2914 | 0 | JP | G03F | G03F0007 | 3 | 17 | 26 | 1 | 4.33 |
| 2915 | 0 | JP | H01L | H01L0033 | 2 | 6 | 32 | 1 | 16.00 |
| 2916 | 0 | JP | H01L | H01L0033 | 5 | 8 | 69 | 1 | 34.50 |
| 2917 | 0 | CA | G01F | G01F0013 | 14 | 4 | 3 | 1 | 1.50 |
| 2918 | 0 | JP | H01L | H01L0021 | 18 | 7 | 56 | 2 | 14.00 |
| 2919 | 0 | JP | H01L | H01L0021 | 20 | 5 | 7 | 1 | 3.50 |
| 2920 | 0 | JP | B41J | B41J0002 | 14 | 6 | 2 | 1 | 0.67 |
| 2921 | 0 | JP | H01L | H01L0033 | 3 | 14 | 12 | 1.5 | 4.00 |
| 2922 | 0 | JP | H01L | H01L0033 | 2 | 8 | 0 | 1 | 0.00 |
| 2923 | 0 | JP | H01L | H01L0033 | 20 | 6 | 5 | 1 | 1.67 |
| 2924 | 0 | JP | H01L | H01L0033 | 2 | 11 | 2 | 1.5 | 0.67 |
| 2925 | 0 | JP | H01J | H01J0001 | 19 | 22 | 8 | 1 | 2.00 |
| 2926 | 0 | JP | H01L | H01L0033 | 8 | 15 | 41 | 4.6 | 1.78 |
| 2927 | 0 | JP | H01S | H01S0005 | 46 | 6 | 6 | 2 | 1.50 |
| 2928 | 0 | US | C09J | C09J0163 | 3 | 3 | 34 | 1 | 8.50 |
| 2929 | 0 | JP | H01L | H01L0033 | 16 | 9 | 22 | 2.666666667 | 2.75 |
| 2930 | 0 | KR | H01L | H01L0021 | 8 | 4 | 0 | 1 | 0.00 |
| 2931 | 0 | JP | H01L | H01L0021 | 8 | 19 | 20 | 1.25 | 4.00 |
| 2932 | 0 | JP | H01L | H01L0021 | 16 | 2 | 1 | 1 | 0.50 |
| 2933 | 0 | KR | H01L | H01L0021 | 16 | 11 | 7 | 1 | 2.33 |
| 2934 | 0 | US | H05B | H05B0033 | 27 | 1 | 12 | 1 | 2.40 |
| 2935 | 0 | JP | H01L | H01L0033 | 5 | 12 | 2 | 1.5 | 0.67 |
| 2936 | 0 | JP | H01L | H01L0033 | 10 | 7 | 4 | 1.25 | 0.80 |
| 2937 | 0 | US | H05K | H05K0001 | 16 | 44 | 24 | 2 | 12.00 |
| 2938 | 0 | JP | G02B | G02B0006 | 6 | 7 | 40 | 1.5 | 13.33 |
| 2939 | 0 | JP | H01H | H01H0001 | 20 | 9 | 2 | 1 | 0.50 |
| 2940 | 0 | US | H01L | H01L0033 | 8 | 6 | 75 | 1 | 8.33 |
| 2941 | 0 | JP | G06F | G06F0003 | 9 | 6 | 37 | 1 | 18.50 |
| 2942 | 0 | JP | H01L | H01L0033 | 6 | 14 | 1 | 1.333333333 | 0.25 |
| 2943 | 0 | US | G01C | G01C0019 | 24 | 87 | 115 | 2.428571429 | 6.76 |
| 2944 | 0 | US | H01L | H01L0027 | 12 | 4 | 51 | 1 | 25.50 |
| 2945 | 0 | JP | H01L | H01L0021 | 37 | 3 | 5 | 1 | 2.50 |
| 2946 | 0 | DE | H01L | H01L0033 | 11 | 17 | 8 | 1 | 2.67 |
| 2947 | 0 | KR | H01L | H01L0021 | 6 | 14 | 1 | 1 | 0.50 |
| 2948 | 0 | JP | H01L | H01L0033 | 9 | 7 | 12 | 1.25 | 2.40 |
| 2949 | 0 | JP | H01L | H01L0033 | 26 | 9 | 39 | 1.5 | 13.00 |
| 2950 | 0 | JP | H01L | H01L0021 | 20 | 14 | 38 | 1 | 19.00 |
| 2951 | 0 | JP | C30B | C30B0025 | 13 | 14 | 19 | 1 | 9.50 |
| 2952 | 0 | JP | H01L | H01L0033 | 11 | 4 | 34 | 1.5 | 11.33 |
| 2953 | 0 | JP | H01L | H01L0021 | 5 | 10 | 1 | 1.666666667 | 0.20 |
| 2954 | 0 | JP | H01L | H01L0033 | 12 | 5 | 44 | 1 | 22.00 |
| 2955 | 0 | JP | H01L | H01L0033 | 11 | 27 | 21 | 1 | 7.00 |
| 2956 | 0 | US | H01L | H01L0027 | 8 | 11 | 14 | 2 | 7.00 |
| 2957 | 0 | JP | H01L | H01L0029 | 15 | 10 | 27 | 3 | 4.50 |
| 2958 | 0 | US | H01L | H01L0021 | 14 | 19 | 8 | 2 | 4.00 |
| 2959 | 0 | JP | H01L | H01L0033 | 1 | 12 | 9 | 1.25 | 1.80 |
| 2960 | 0 | JP | H01L | H01L0021 | 13 | 10 | 3 | 1 | 1.00 |
| 2961 | 0 | US | H01L | H01L0021 | 17 | 28 | 159 | 1.111111111 | 15.90 |
| 2962 | 0 | JP | H01L | H01L0021 | 7 | 6 | 11 | 1 | 2.75 |
| 2963 | 0 | US | H01L | H01L0021 | 4 | 13 | 2 | 2 | 1.00 |
| 2964 | 0 | US | H01L | H01L0021 | 13 | 8 | 13 | 2 | 6.50 |
| 2965 | 0 | US | H01L | H01L0021 | 17 | 7 | 61 | 2 | 30.50 |
| 2966 | 0 | US | H01H | H01H0001 | 14 | 36 | 33 | 2 | 16.50 |
| 2967 | 0 | JP | C30B | C30B0019 | 24 | 16 | 9 | 1 | 2.25 |
| 2968 | 0 | DE | H01L | H01L0029 | 6 | 7 | 27 | 1.2 | 4.50 |
| 2969 | 0 | JP | H01L | H01L0033 | 8 | 9 | 4 | 1 | 1.33 |
| 2970 | 0 | US | H01L | H01L0021 | 22 | 20 | 18 | 1 | 1.64 |
| 2971 | 0 | JP | H01L | H01L0021 | 10 | 5 | 12 | 1.5 | 4.00 |
| 2972 | 0 | JP | H01L | H01L0029 | 5 | 11 | 0 | 1.5 | 0.00 |
| 2973 | 0 | US | H01L | H01L0025 | 15 | 9 | 46 | 1 | 9.20 |
| 2974 | 0 | US | H01L | H01L0021 | 7 | 16 | 3 | 1 | 1.00 |
| 2975 | 0 | JP | H01L | H01L0029 | 10 | 10 | 69 | 3 | 11.50 |
| 2976 | 0 | JP | C30B | C30B0025 | 7 | 10 | 9 | 1 | 4.50 |
| 2977 | 0 | JP | H01L | H01L0029 | 16 | 3 | 43 | 1.333333333 | 10.75 |
| 2978 | 0 | US | H01S | H01S0005 | 9 | 16 | 13 | 1 | 2.60 |
| 2979 | 0 | JP | H01L | H01L0033 | 5 | 1 | 9 | 1 | 4.50 |
| 2980 | 0 | US | H01L | H01L0021 | 7 | 5 | 45 | 2 | 22.50 |
| 2981 | 0 | US | B41J | B41J0002 | 30 | 5 | 25 | 1 | 12.50 |
| 2982 | 0 | JP | H01L | H01L0033 | 8 | 6 | 29 | 1 | 14.50 |
| 2983 | 1 | US | H01L | H01L0021 | 10 | 28 | 12 | 1.25 | 2.40 |
| 2984 | 0 | JP | H01L | H01L0021 | 1 | 5 | 16 | 1 | 8.00 |
| 2985 | 0 | JP | B41J | B41J0002 | 13 | 13 | 3 | 1.25 | 0.60 |
| 2986 | 0 | JP | H01L | H01L0033 | 39 | 11 | 51 | 2.2 | 4.64 |
| 2987 | 0 | JP | C30B | C30B0025 | 6 | 23 | 11 | 2.5 | 2.20 |
| 2988 | 0 | US | H01L | H01L0033 | 23 | 3 | 64 | 1 | 32.00 |
| 2989 | 0 | JP | H01L | H01L0029 | 19 | 23 | 40 | 1 | 10.00 |
| 2990 | 0 | JP | H01L | H01L0033 | 7 | 6 | 3 | 1 | 1.50 |
| 2991 | 0 | JP | H01L | H01L0029 | 14 | 9 | 11 | 1 | 5.50 |
| 2992 | 0 | US | C04B | C04B0035 | 15 | 79 | 27 | 2.047619048 | 0.63 |
| 2993 | 0 | JP | C09K | C09K0011 | 9 | 8 | 1 | 1.25 | 0.20 |
| 2994 | 0 | JP | H01L | H01L0027 | 19 | 7 | 9 | 1.75 | 1.29 |
| 2995 | 0 | JP | B41J | B41J0002 | 10 | 28 | 16 | 1 | 8.00 |
| 2996 | 0 | US | H01L | H01L0033 | 16 | 1 | 8 | 1.5 | 2.67 |
| 2997 | 0 | US | H01L | H01L0029 | 16 | 23 | 10 | 1.2 | 1.67 |
| 2998 | 0 | US | C08F | C08F0220 | 22 | 5 | 96 | 1.8 | 10.67 |
| 2999 | 0 | JP | H01L | H01L0033 | 13 | 6 | 33 | 1 | 16.50 |
| 3000 | 0 | US | H01L | H01L0021 | 6 | 15 | 11 | 1 | 5.50 |
| 3001 | 0 | JP | H01L | H01L0021 | 8 | 2 | 2 | 1.333333333 | 0.50 |
| 3002 | 0 | JP | H01L | H01L0021 | 52 | 25 | 148 | 1.272727273 | 10.57 |
| 3003 | 0 | US | H01L | H01L0033 | 4 | 45 | 10 | 1.5 | 1.67 |
| 3004 | 0 | US | C23C | C23C0014 | 8 | 2 | 4 | 1 | 1.00 |
| 3005 | 0 | GB | G02F | G02F0001 | 23 | 12 | 9 | 1 | 4.50 |
| 3006 | 0 | JP | B41J | B41J0002 | 11 | 15 | 4 | 1 | 1.33 |
| 3007 | 0 | JP | H01S | H01S0005 | 12 | 8 | 18 | 1 | 9.00 |
| 3008 | 0 | JP | H01L | H01L0033 | 14 | 5 | 28 | 2 | 7.00 |
| 3009 | 0 | US | H01L | H01L0033 | 18 | 14 | 11 | 1 | 1.57 |
| 3010 | 0 | JP | H01L | H01L0033 | 10 | 9 | 7 | 1 | 1.17 |
| 3011 | 0 | US | C23C | C23C0016 | 5 | 10 | 18 | 1.5 | 6.00 |
| 3012 | 0 | JP | H01L | H01L0033 | 16 | 13 | 27 | 1 | 5.40 |
| 3013 | 0 | JP | H01L | H01L0033 | 18 | 7 | 45 | 1 | 22.50 |
| 3014 | 0 | JP | H01L | H01L0029 | 6 | 1 | 36 | 1 | 18.00 |
| 3015 | 0 | JP | C30B | C30B0019 | 3 | 16 | 0 | 1 | 0.00 |
| 3016 | 0 | JP | H01L | H01L0033 | 14 | 4 | 2 | 1 | 0.67 |
| 3017 | 0 | US | H01L | H01L0023 | 12 | 15 | 13 | 1.2 | 2.17 |
| 3018 | 0 | JP | C30B | C30B0023 | 1 | 14 | 4 | 1.25 | 0.80 |
| 3019 | 0 | JP | H01L | H01L0033 | 12 | 6 | 68 | 1 | 34.00 |
| 3020 | 0 | JP | H01L | H01L0033 | 12 | 2 | 17 | 1 | 5.67 |
| 3021 | 0 | US | H01L | H01L0029 | 2 | 4 | 40 | 1.2 | 6.67 |
| 3022 | 0 | JP | H01S | H01S0005 | 8 | 4 | 17 | 2.5 | 3.40 |
| 3023 | 1 | JP | C09K | C09K0011 | 23 | 18 | 417 | 5 | 4.63 |
| 3024 | 0 | JP | C30B | C30B0029 | 16 | 21 | 5 | 1 | 1.00 |
| 3025 | 0 | US | H01L | H01L0033 | 10 | 6 | 13 | 1 | 6.50 |
| 3026 | 0 | US | C30B | C30B0023 | 14 | 14 | 65 | 1.333333333 | 16.25 |
| 3027 | 0 | US | C08J | C08J0003 | 11 | 17 | 0 | 2 | 0.00 |
| 3028 | 0 | KR | H01L | H01L0023 | 13 | 7 | 8 | 1.333333333 | 2.00 |
| 3029 | 0 | JP | H01L | H01L0033 | 13 | 16 | 10 | 3.75 | 0.67 |
| 3030 | 0 | JP | H01L | H01L0033 | 5 | 6 | 4 | 1.5 | 1.33 |
| 3031 | 0 | JP | H01L | H01L0033 | 3 | 4 | 1 | 1.333333333 | 0.25 |
| 3032 | 0 | JP | H01L | H01L0021 | 3 | 7 | 44 | 1 | 22.00 |
| 3033 | 0 | JP | H01L | H01L0021 | 5 | 23 | 62 | 1.5 | 20.67 |
| 3034 | 0 | JP | H01L | H01L0023 | 5 | 16 | 23 | 1 | 11.50 |
| 3035 | 0 | JP | H01L | H01L0033 | 13 | 5 | 4 | 1.25 | 0.80 |
| 3036 | 0 | US | H01L | H01L0033 | 9 | 35 | 14 | 1.25 | 2.80 |
| 3037 | 0 | US | H01L | H01L0023 | 8 | 18 | 9 | 1 | 4.50 |
| 3038 | 0 | JP | H01L | H01L0033 | 18 | 11 | 87 | 1 | 43.50 |
| 3039 | 0 | JP | H01L | H01L0021 | 15 | 12 | 33 | 1 | 11.00 |
| 3040 | 0 | JP | C30B | C30B0025 | 15 | 10 | 23 | 1.333333333 | 5.75 |
| 3041 | 0 | JP | H01L | H01L0023 | 17 | 18 | 16 | 1 | 4.00 |
| 3042 | 0 | JP | H01L | H01L0033 | 3 | 1 | 1 | 1 | 0.50 |
| 3043 | 0 | JP | H01L | H01L0033 | 11 | 6 | 28 | 1.5 | 9.33 |
| 3044 | 0 | KR | H01L | H01L0023 | 44 | 13 | 118 | 1.571428571 | 10.73 |
| 3045 | 0 | US | H01L | H01L0021 | 7 | 4 | 5 | 1 | 1.67 |
| 3046 | 0 | JP | H01L | H01L0033 | 7 | 5 | 13 | 1 | 6.50 |
| 3047 | 0 | JP | H01L | H01L0033 | 12 | 16 | 14 | 2.8 | 1.00 |
| 3048 | 0 | JP | B41J | B41J0002 | 22 | 13 | 5 | 1.25 | 1.00 |
| 3049 | 0 | JP | H01L | H01L0033 | 6 | 7 | 17 | 1 | 8.50 |
| 3050 | 0 | JP | H01L | H01L0021 | 23 | 9 | 24 | 1.333333333 | 6.00 |
| 3051 | 0 | JP | C30B | C30B0029 | 20 | 8 | 3 | 1 | 1.00 |
| 3052 | 0 | JP | H01L | H01L0021 | 4 | 6 | 11 | 1 | 5.50 |
| 3053 | 0 | KR | H01L | H01L0021 | 10 | 9 | 12 | 1 | 6.00 |
| 3054 | 0 | JP | H01L | H01L0031 | 20 | 11 | 20 | 1 | 4.00 |
| 3055 | 0 | JP | H01L | H01L0033 | 9 | 1 | 5 | 1.25 | 1.00 |
| 3056 | 0 | JP | H01L | H01L0029 | 10 | 3 | 26 | 2.666666667 | 3.25 |
| 3057 | 0 | US | H01L | H01L0033 | 16 | 22 | 21 | 2.375 | 0.55 |
| 3058 | 0 | VG | C08G | C08G0008 | 12 | 8 | 1 | 1.333333333 | 0.13 |
| 3059 | 0 | JP | H01L | H01L0021 | 6 | 9 | 177 | 1 | 88.50 |
| 3060 | 0 | JP | G03F | G03F0007 | 2 | 28 | 5 | 1.8 | 0.56 |
| 3061 | 0 | US | G21F | G21F0009 | 10 | 24 | 7 | 1 | 1.75 |
| 3062 | 0 | US | C30B | C30B0025 | 61 | 33 | 55 | 1 | 6.88 |
| 3063 | 0 | JP | H01L | H01L0033 | 7 | 4 | 12 | 1.5 | 4.00 |
| 3064 | 0 | JP | H01L | H01L0021 | 51 | 5 | 37 | 1 | 18.50 |
| 3065 | 0 | JP | H01L | H01L0025 | 27 | 3 | 62 | 4.5 | 6.89 |
| 3066 | 0 | TW | H01L | H01L0033 | 23 | 4 | 28 | 1 | 7.00 |
| 3067 | 0 | TW | H01L | H01L0033 | 4 | 1 | 3 | 1 | 0.75 |
| 3068 | 0 | JP | H01L | H01L0029 | 8 | 10 | 16 | 1 | 8.00 |
| 3069 | 0 | JP | H01L | H01L0033 | 17 | 7 | 35 | 1 | 17.50 |
| 3070 | 0 | JP | B32B | B32B0015 | 23 | 14 | 53 | 3 | 4.42 |
| 3071 | 0 | JP | H01L | H01L0021 | 19 | 8 | 31 | 1 | 15.50 |
| 3072 | 0 | JP | H01L | H01L0023 | 7 | 15 | 49 | 1 | 16.33 |
| 3073 | 0 | JP | H01L | H01L0021 | 6 | 5 | 24 | 1 | 6.00 |
| 3074 | 0 | JP | H01L | H01L0033 | 3 | 6 | 9 | 2 | 2.25 |
| 3075 | 0 | JP | H01L | H01L0033 | 7 | 4 | 2 | 1 | 1.00 |
| 3076 | 0 | US | H01L | H01L0021 | 10 | 9 | 93 | 1.333333333 | 23.25 |
| 3077 | 0 | TW | H01L | H01L0033 | 12 | 5 | 4 | 1 | 2.00 |
| 3078 | 0 | JP | H01S | H01S0005 | 28 | 6 | 43 | 1 | 10.75 |
| 3079 | 0 | JP | H01L | H01L0033 | 9 | 5 | 1 | 1 | 0.33 |
| 3080 | 0 | JP | H01L | H01L0021 | 10 | 7 | 58 | 3.5 | 8.29 |
| 3081 | 0 | US | H01L | H01L0025 | 17 | 13 | 372 | 1 | 62.00 |
| 3082 | 0 | JP | H01L | H01L0029 | 3 | 4 | 5 | 1 | 2.50 |
| 3083 | 0 | JP | C30B | C30B0025 | 9 | 6 | 13 | 1.333333333 | 3.25 |
| 3084 | 0 | JP | F21V | F21V0008 | 8 | 22 | 187 | 5 | 2.08 |
| 3085 | 0 | US | C08F | C08F0220 | 10 | 20 | 72 | 1.8 | 8.00 |
| 3086 | 0 | US | C08F | C08F0002 | 7 | 12 | 6 | 1.181818182 | 0.46 |
| 3087 | 0 | JP | H01L | H01L0033 | 17 | 17 | 29 | 1.2 | 4.83 |
| 3088 | 0 | JP | H01L | H01L0033 | 21 | 20 | 33 | 7 | 2.36 |
| 3089 | 0 | JP | H01L | H01L0027 | 41 | 3 | 51 | 1 | 25.50 |
| 3090 | 0 | JP | H01L | H01L0029 | 18 | 13 | 2 | 1.5 | 0.67 |
| 3091 | 0 | JP | H01S | H01S0005 | 4 | 10 | 22 | 1.5 | 7.33 |
| 3092 | 0 | JP | H01L | H01L0021 | 4 | 6 | 25 | 1.5 | 8.33 |
| 3093 | 0 | JP | H01L | H01L0033 | 10 | 14 | 1 | 1.333333333 | 0.25 |
| 3094 | 0 | US | H01L | H01L0033 | 22 | 20 | 99 | 1 | 14.14 |
| 3095 | 0 | US | C23C | C23C0016 | 4 | 13 | 12 | 1 | 4.00 |
| 3096 | 0 | JP | H01L | H01L0033 | 17 | 25 | 5 | 1 | 2.50 |
| 3097 | 0 | JP | H01L | H01L0033 | 4 | 26 | 20 | 4.6 | 0.87 |
| 3098 | 0 | US | H01L | H01L0033 | 18 | 5 | 102 | 1 | 25.50 |
| 3099 | 0 | JP | H01L | H01L0033 | 21 | 11 | 10 | 2 | 2.50 |
| 3100 | 0 | JP | H01L | H01L0033 | 8 | 4 | 8 | 1.5 | 2.67 |
| 3101 | 0 | JP | H01L | H01L0033 | 5 | 4 | 7 | 1 | 1.40 |
| 3102 | 0 | JP | H01L | H01L0021 | 10 | 5 | 16 | 1 | 4.00 |
| 3103 | 0 | US | C30B | C30B0025 | 20 | 10 | 42 | 1 | 14.00 |
| 3104 | 0 | JP | H01L | H01L0021 | 5 | 8 | 5 | 1.5 | 1.67 |
| 3105 | 0 | US | C09K | C09K0011 | 7 | 4 | 168 | 2 | 16.80 |
| 3106 | 0 | JP | H01L | H01L0023 | 9 | 10 | 23 | 1 | 5.75 |
| 3107 | 0 | US | H01L | H01L0023 | 12 | 9 | 171 | 1.333333333 | 42.75 |
| 3108 | 1 | JP | H01L | H01L0033 | 45 | 29 | 42 | 5.5 | 3.82 |
| 3109 | 0 | JP | H01L | H01L0031 | 14 | 24 | 7 | 1.2 | 1.17 |
| 3110 | 0 | DE | H01L | H01L0025 | 33 | 14 | 6 | 1.166666667 | 0.86 |
| 3111 | 0 | JP | H01L | H01L0033 | 5 | 13 | 42 | 5.5 | 3.82 |
| 3112 | 0 | JP | H01L | H01L0023 | 18 | 6 | 5 | 1 | 1.00 |
| 3113 | 0 | JP | H01L | H01L0033 | 15 | 22 | 5 | 1 | 2.50 |
| 3114 | 0 | JP | H01L | H01L0021 | 11 | 45 | 6 | 2.8 | 0.43 |
| 3115 | 0 | JP | H01L | H01L0033 | 6 | 8 | 6 | 1 | 1.50 |
| 3116 | 0 | US | C30B | C30B0023 | 46 | 15 | 28 | 1.5 | 3.11 |
| 3117 | 0 | JP | H01L | H01L0023 | 10 | 5 | 4 | 1 | 1.33 |
| 3118 | 0 | US | H05B | H05B0033 | 18 | 15 | 7 | 1 | 1.75 |
| 3119 | 0 | US | H01L | H01L0027 | 9 | 8 | 24 | 1 | 12.00 |
| 3120 | 0 | US | H01L | H01L0027 | 9 | 12 | 23 | 1 | 7.67 |
| 3121 | 0 | JP | C30B | C30B0029 | 17 | 11 | 9 | 1 | 4.50 |
| 3122 | 0 | JP | H01L | H01L0021 | 36 | 6 | 20 | 1 | 6.67 |
| 3123 | 0 | JP | H01L | H01L0023 | 16 | 2 | 22 | 1 | 11.00 |
| 3124 | 0 | JP | C30B | C30B0023 | 43 | 12 | 1 | 1 | 0.33 |
| 3125 | 0 | US | H01L | H01L0021 | 20 | 4 | 20 | 2 | 2.00 |
| 3126 | 0 | US | H01L | H01L0021 | 12 | 5 | 23 | 1.125 | 2.56 |
| 3127 | 0 | KR | H01L | H01L0033 | 9 | 13 | 13 | 1 | 6.50 |
| 3128 | 0 | JP | H01L | H01L0033 | 12 | 11 | 14 | 1 | 7.00 |
| 3129 | 0 | SG | H01L | H01L0021 | 18 | 9 | 78 | 1.5 | 26.00 |
| 3130 | 0 | JP | H01L | H01L0033 | 8 | 4 | 54 | 4.5 | 6.00 |
| 3131 | 0 | US | F28D | F28D0015 | 5 | 20 | 58 | 1.4 | 8.29 |
| 3132 | 0 | JP | H01L | H01L0039 | 5 | 7 | 44 | 2 | 11.00 |
| 3133 | 0 | US | H01L | H01L0021 | 6 | 34 | 14 | 1 | 4.67 |
| 3134 | 0 | US | H01L | H01L0021 | 9 | 30 | 75 | 1.25 | 15.00 |
| 3135 | 0 | US | H01L | H01L0021 | 17 | 15 | 8 | 1 | 4.00 |
| 3136 | 0 | JP | H01L | H01L0021 | 9 | 9 | 2 | 1 | 0.33 |
| 3137 | 0 | JP | H01L | H01L0021 | 11 | 31 | 3 | 2.666666667 | 0.38 |
| 3138 | 0 | JP | H01S | H01S0005 | 11 | 16 | 17 | 1.5 | 5.67 |
| 3139 | 0 | JP | H01L | H01L0021 | 20 | 13 | 63 | 3.333333333 | 6.30 |
| 3140 | 0 | JP | H01L | H01L0033 | 10 | 1 | 33 | 3.5 | 4.71 |
| 3141 | 0 | JP | H01L | H01L0033 | 8 | 6 | 10 | 2 | 2.50 |
| 3142 | 0 | JP | H01L | H01L0033 | 9 | 3 | 18 | 1.666666667 | 3.60 |
| 3143 | 0 | DE | H01L | H01L0021 | 13 | 3 | 33 | 1 | 11.00 |
| 3144 | 0 | JP | H01L | H01L0021 | 1 | 21 | 9 | 1.25 | 1.80 |
| 3145 | 0 | DE | H01L | H01L0033 | 4 | 9 | 33 | 1.5 | 11.00 |
| 3146 | 0 | JP | H01L | H01L0021 | 17 | 3 | 46 | 1.5 | 15.33 |
| 3147 | 0 | US | C09K | C09K0011 | 3 | 2 | 0 | 1 | 0.00 |
| 3148 | 0 | FI | C09K | C09K0011 | 20 | 12 | 74 | 1 | 18.50 |
| 3149 | 0 | JP | H01L | H01L0021 | 15 | 10 | 22 | 1.5 | 7.33 |
| 3150 | 0 | US | G03F | G03F0007 | 17 | 75 | 34 | 7.6 | 0.89 |
| 3151 | 0 | US | H01L | H01L0033 | 7 | 81 | 145 | 1.125 | 16.11 |
| 3152 | 0 | JP | H01L | H01L0021 | 3 | 11 | 122 | 4.5 | 13.56 |
| 3153 | 0 | JP | H01L | H01L0033 | 17 | 6 | 34 | 1 | 17.00 |
| 3154 | 0 | JP | H01L | H01L0033 | 15 | 5 | 23 | 1 | 7.67 |
| 3155 | 0 | JP | H01L | H01L0033 | 18 | 3 | 31 | 1 | 15.50 |
| 3156 | 0 | JP | H01L | H01L0033 | 10 | 3 | 29 | 1.666666667 | 5.80 |
| 3157 | 0 | JP | H01L | H01L0033 | 3 | 4 | 17 | 1.5 | 5.67 |
| 3158 | 0 | US | C23C | C23C0016 | 23 | 11 | 8 | 1 | 1.33 |
| 3159 | 0 | JP | H05K | H05K0001 | 23 | 19 | 9 | 1.333333333 | 2.25 |
| 3160 | 0 | JP | H01L | H01L0021 | 18 | 5 | 21 | 1.25 | 4.20 |
| 3161 | 0 | JP | H01L | H01L0033 | 11 | 3 | 1 | 1 | 0.50 |
| 3162 | 0 | CA | H01L | H01L0033 | 14 | 6 | 77 | 1.2 | 12.83 |
| 3163 | 0 | JP | H05B | H05B0033 | 6 | 4 | 10 | 1 | 3.33 |
| 3164 | 0 | JP | H01L | H01L0027 | 22 | 25 | 7 | 1.5 | 1.17 |
| 3165 | 0 | US | H01L | H01L0029 | 6 | 23 | 6 | 1.25 | 1.20 |
| 3166 | 0 | US | H01L | H01L0021 | 6 | 22 | 17 | 1 | 5.67 |
| 3167 | 0 | JP | C30B | C30B0029 | 13 | 6 | 42 | 1 | 14.00 |
| 3168 | 0 | US | C30B | C30B0025 | 30 | 4 | 22 | 1 | 11.00 |
| 3169 | 0 | US | H01S | H01S0005 | 28 | 22 | 11 | 1 | 5.50 |
| 3170 | 0 | DE | H01L | H01L0029 | 4 | 18 | 4 | 1.2 | 0.67 |
| 3171 | 0 | US | H01L | H01L0021 | 22 | 27 | 14 | 2 | 7.00 |
| 3172 | 0 | JP | H01L | H01L0027 | 11 | 4 | 3 | 1 | 1.50 |
| 3173 | 0 | JP | H01L | H01L0033 | 8 | 8 | 2 | 1.2 | 0.33 |
| 3174 | 0 | US | H01L | H01L0023 | 34 | 16 | 25 | 1.125 | 2.78 |
| 3175 | 0 | JP | C30B | C30B0023 | 15 | 27 | 10 | 1 | 2.00 |
| 3176 | 0 | JP | H01L | H01L0021 | 12 | 10 | 19 | 1.25 | 3.80 |
| 3177 | 0 | JP | H01L | H01L0021 | 5 | 8 | 72 | 1.5 | 24.00 |
| 3178 | 0 | JP | H01L | H01L0033 | 8 | 13 | 85 | 1 | 21.25 |
| 3179 | 0 | JP | H01L | H01L0021 | 7 | 7 | 47 | 1 | 15.67 |
| 3180 | 0 | JP | H01L | H01L0033 | 3 | 7 | 3 | 1 | 1.50 |
| 3181 | 0 | TW | H01L | H01L0033 | 43 | 11 | 50 | 1 | 25.00 |
| 3182 | 0 | JP | H01L | H01L0033 | 18 | 9 | 3 | 1 | 0.60 |
| 3183 | 0 | JP | C30B | C30B0025 | 25 | 23 | 19 | 1.5 | 3.17 |
| 3184 | 0 | JP | H01L | H01L0021 | 6 | 6 | 4 | 1 | 2.00 |
| 3185 | 0 | JP | H01S | H01S0005 | 4 | 7 | 2 | 1.5 | 0.67 |
| 3186 | 0 | JP | H01L | H01L0033 | 12 | 10 | 9 | 1 | 1.80 |
| 3187 | 0 | JP | C30B | C30B0029 | 17 | 16 | 1 | 1 | 0.33 |
| 3188 | 0 | JP | H01L | H01L0051 | 14 | 16 | 6 | 1 | 1.50 |
| 3189 | 0 | TW | H01S | H01S0005 | 20 | 2 | 8 | 1 | 4.00 |
| 3190 | 0 | KR | C30B | C30B0029 | 30 | 1 | 84 | 2 | 14.00 |
| 3191 | 0 | JP | H01L | H01L0033 | 16 | 7 | 6 | 1 | 3.00 |
| 3192 | 0 | JP | H01L | H01L0033 | 32 | 7 | 3 | 1 | 1.50 |
| 3193 | 0 | JP | H01L | H01L0033 | 5 | 11 | 12 | 1 | 3.00 |
| 3194 | 0 | JP | H01L | H01L0033 | 6 | 8 | 13 | 2.333333333 | 1.86 |
| 3195 | 0 | JP | B22F | B22F0003 | 14 | 15 | 13 | 1.8 | 1.44 |
| 3196 | 0 | JP | C30B | C30B0025 | 5 | 1 | 3 | 1 | 0.75 |
| 3197 | 0 | JP | H01L | H01L0021 | 30 | 48 | 39 | 2 | 9.75 |
| 3198 | 0 | JP | C30B | C30B0029 | 4 | 4 | 8 | 1 | 2.00 |
| 3199 | 0 | US | H01L | H01L0021 | 10 | 12 | 107 | 1.857142857 | 8.23 |
| 3200 | 0 | JP | H01L | H01L0021 | 28 | 11 | 0 | 1.5 | 0.00 |
| 3201 | 0 | JP | H01L | H01L0021 | 12 | 16 | 1 | 2 | 0.25 |
| 3202 | 0 | JP | H01L | H01L0021 | 6 | 23 | 3 | 1 | 1.50 |
| 3203 | 0 | JP | H01L | H01L0027 | 2 | 16 | 6 | 1.75 | 0.86 |
| 3204 | 0 | JP | H01L | H01L0021 | 35 | 19 | 21 | 2 | 5.25 |
| 3205 | 0 | JP | H01L | H01L0033 | 9 | 4 | 10 | 2 | 2.50 |
| 3206 | 0 | JP | H01L | H01L0033 | 22 | 6 | 6 | 1 | 1.50 |
| 3207 | 0 | JP | H01L | H01L0021 | 3 | 8 | 10 | 3.333333333 | 1.00 |
| 3208 | 0 | JP | H01L | H01L0021 | 6 | 2 | 6 | 1 | 3.00 |
| 3209 | 0 | JP | H01L | H01L0021 | 11 | 8 | 41 | 3.333333333 | 4.10 |
| 3210 | 0 | CA | H01L | H01L0021 | 7 | 13 | 33 | 1.25 | 6.60 |
| 3211 | 0 | US | H01L | H01L0033 | 13 | 1 | 5 | 1 | 2.50 |
| 3212 | 0 | JP | H01L | H01L0021 | 6 | 6 | 15 | 3.5 | 2.14 |
| 3213 | 0 | JP | H01L | H01L0021 | 5 | 2 | 15 | 1 | 5.00 |
| 3214 | 0 | JP | H01L | H01L0029 | 9 | 16 | 13 | 1 | 6.50 |
| 3215 | 0 | JP | H01L | H01L0033 | 4 | 3 | 2 | 1 | 0.50 |
| 3216 | 0 | US | H01L | H01L0021 | 16 | 14 | 126 | 1.857142857 | 9.69 |
| 3217 | 0 | CA | H01L | H01L0033 | 23 | 9 | 20 | 1.166666667 | 2.86 |
| 3218 | 0 | JP | C03C | C03C0017 | 7 | 8 | 3 | 1.333333333 | 0.75 |
| 3219 | 0 | JP | H01S | H01S0005 | 15 | 15 | 4 | 1 | 2.00 |
| 3220 | 0 | JP | H01S | H01S0005 | 12 | 8 | 20 | 1.333333333 | 5.00 |
| 3221 | 0 | CA | H01L | H01L0033 | 7 | 15 | 55 | 1.2 | 9.17 |
| 3222 | 0 | JP | H01L | H01L0021 | 9 | 1 | 4 | 1 | 2.00 |
| 3223 | 0 | JP | C30B | C30B0023 | 11 | 11 | 4 | 1 | 1.00 |
| 3224 | 0 | JP | H01L | H01L0021 | 9 | 6 | 2 | 1 | 1.00 |
| 3225 | 0 | JP | H01L | H01L0033 | 41 | 21 | 70 | 3.5 | 10.00 |
| 3226 | 0 | JP | H01L | H01L0021 | 22 | 19 | 29 | 2 | 7.25 |
| 3227 | 0 | US | C09D | C09D0127 | 18 | 5 | 9 | 1.166666667 | 1.29 |
| 3228 | 0 | US | H01S | H01S0005 | 11 | 9 | 40 | 1 | 10.00 |
| 3229 | 0 | KR | H01L | H01L0021 | 16 | 5 | 14 | 1 | 7.00 |
| 3230 | 0 | US | H01L | H01L0033 | 6 | 3 | 10 | 1.5 | 3.33 |
| 3231 | 0 | US | H05B | H05B0033 | 5 | 3 | 0 | 1 | 0.00 |
| 3232 | 0 | JP | H01L | H01L0033 | 13 | 26 | 16 | 4.6 | 0.70 |
| 3233 | 0 | JP | H01L | H01L0021 | 13 | 13 | 7 | 1.5 | 2.33 |
| 3234 | 0 | US | H01L | H01L0021 | 26 | 33 | 24 | 1.571428571 | 2.18 |
| 3235 | 0 | US | C09K | C09K0011 | 3 | 11 | 10 | 1.142857143 | 1.25 |
| 3236 | 0 | JP | H01L | H01L0033 | 25 | 4 | 0 | 1 | 0.00 |
| 3237 | 0 | DE | H01L | H01L0033 | 9 | 6 | 21 | 1.333333333 | 2.63 |
| 3238 | 0 | JP | H01L | H01L0033 | 12 | 14 | 15 | 1.5 | 5.00 |
| 3239 | 0 | DE | H01L | H01L0033 | 33 | 14 | 3 | 1 | 1.00 |
| 3240 | 0 | CA | H01L | H01L0033 | 23 | 11 | 83 | 1.2 | 13.83 |
| 3241 | 0 | JP | G09F | G09F0009 | 14 | 13 | 5 | 1 | 2.50 |
| 3242 | 0 | JP | H01L | H01L0021 | 10 | 13 | 6 | 1.4 | 0.86 |
| 3243 | 0 | JP | H01L | H01L0031 | 15 | 5 | 8 | 3 | 0.53 |
| 3244 | 0 | CA | H01L | H01L0033 | 18 | 17 | 72 | 2.2 | 6.55 |
| 3245 | 0 | JP | H01L | H01L0033 | 23 | 22 | 9 | 1.5 | 3.00 |
| 3246 | 0 | JP | H01L | H01L0021 | 15 | 6 | 5 | 2 | 1.25 |
| 3247 | 0 | US | H01L | H01L0033 | 21 | 4 | 60 | 1 | 30.00 |
| 3248 | 0 | JP | H01L | H01L0021 | 11 | 2 | 38 | 2 | 9.50 |
| 3249 | 0 | JP | H01L | H01L0021 | 24 | 28 | 11 | 2.5 | 2.20 |
| 3250 | 0 | US | H01L | H01L0021 | 4 | 11 | 10 | 1.333333333 | 2.50 |
| 3251 | 0 | JP | H01L | H01L0033 | 5 | 3 | 5 | 1 | 2.50 |
| 3252 | 0 | JP | H01L | H01L0033 | 8 | 7 | 3 | 1 | 1.50 |
| 3253 | 0 | JP | G09F | G09F0009 | 2 | 8 | 23 | 1.333333333 | 5.75 |
| 3254 | 0 | US | H01L | H01L0033 | 16 | 6 | 41 | 1 | 20.50 |
| 3255 | 0 | US | F21V | F21V0007 | 36 | 5 | 13 | 1.5 | 4.33 |
| 3256 | 0 | JP | H01L | H01L0021 | 3 | 9 | 10 | 1.5 | 3.33 |
| 3257 | 0 | JP | H01L | H01L0021 | 16 | 6 | 2 | 1 | 1.00 |
| 3258 | 0 | US | H01L | H01L0021 | 36 | 20 | 36 | 1 | 12.00 |
| 3259 | 0 | JP | H01L | H01L0025 | 21 | 16 | 92 | 1.666666667 | 18.40 |
| 3260 | 0 | US | C08L | C08L0061 | 6 | 10 | 5 | 1.142857143 | 0.63 |
| 3261 | 0 | KR | H01L | H01L0021 | 8 | 13 | 11 | 1 | 5.50 |
| 3262 | 0 | JP | H01L | H01L0033 | 4 | 20 | 1 | 1 | 0.50 |
| 3263 | 0 | FI | C03C | C03C0017 | 18 | 17 | 68 | 1 | 17.00 |
| 3264 | 0 | JP | H01L | H01L0021 | 6 | 5 | 14 | 1 | 4.67 |
| 3265 | 0 | JP | H01L | H01L0033 | 9 | 79 | 7 | 3.4 | 0.41 |
| 3266 | 0 | JP | G02B | G02B0006 | 17 | 6 | 11 | 1 | 2.75 |
| 3267 | 0 | US | C30B | C30B0023 | 17 | 16 | 37 | 1.5 | 12.33 |
| 3268 | 1 | US | H01L | H01L0021 | 29 | 21 | 178 | 3 | 11.87 |
| 3269 | 0 | US | F21V | F21V0019 | 4 | 3 | 11 | 2 | 5.50 |
| 3270 | 0 | JP | H01L | H01L0021 | 12 | 3 | 28 | 2 | 4.67 |
| 3271 | 0 | JP | H01L | H01L0025 | 7 | 20 | 45 | 2.833333333 | 2.65 |
| 3272 | 0 | DE | C30B | C30B0029 | 15 | 6 | 8 | 1 | 2.67 |
| 3273 | 0 | US | C03C | C03C0004 | 13 | 14 | 11 | 1 | 2.20 |
| 3274 | 0 | US | C23C | C23C0016 | 12 | 9 | 16 | 1 | 5.33 |
| 3275 | 0 | US | H01L | H01L0027 | 16 | 8 | 7 | 3 | 0.58 |
| 3276 | 0 | US | C30B | C30B0023 | 27 | 11 | 53 | 3.333333333 | 2.65 |
| 3277 | 0 | US | H01L | H01L0033 | 18 | 5 | 54 | 1 | 10.80 |
| 3278 | 0 | US | H01L | H01L0021 | 7 | 23 | 43 | 1.4 | 6.14 |
| 3279 | 0 | CA | H01L | H01L0033 | 13 | 14 | 20 | 1 | 5.00 |
| 3280 | 0 | CA | H01L | H01L0033 | 5 | 4 | 31 | 1.142857143 | 3.88 |
| 3281 | 0 | JP | H01L | H01L0021 | 3 | 7 | 3 | 1.5 | 1.00 |
| 3282 | 0 | RU | C07C | C07C0043 | 27 | 27 | 1 | 1.666666667 | 0.20 |
| 3283 | 0 | JP | G09F | G09F0009 | 24 | 23 | 12 | 1 | 4.00 |
| 3284 | 0 | US | C23F | C23F0004 | 17 | 8 | 5 | 1 | 1.25 |
| 3285 | 0 | FR | H01L | H01L0029 | 25 | 24 | 17 | 1 | 3.40 |
| 3286 | 0 | US | C23C | C23C0016 | 19 | 12 | 17 | 1 | 5.67 |
| 3287 | 0 | US | B22F | B22F0001 | 15 | 10 | 13 | 2.333333333 | 1.86 |
| 3288 | 0 | US | G02B | G02B0006 | 9 | 19 | 14 | 1.166666667 | 2.00 |
| 3289 | 0 | DE | H01L | H01L0033 | 13 | 31 | 1 | 1 | 0.33 |
| 3290 | 0 | US | H01L | H01L0021 | 21 | 9 | 11 | 1 | 5.50 |
| 3291 | 0 | US | H01L | H01L0021 | 16 | 13 | 7 | 1.333333333 | 1.75 |
| 3292 | 0 | JP | H01L | H01L0033 | 9 | 32 | 5 | 3.75 | 0.33 |
| 3293 | 0 | JP | H01S | H01S0005 | 16 | 8 | 5 | 2 | 0.63 |
| 3294 | 0 | DE | H01S | H01S0005 | 3 | 19 | 8 | 1.166666667 | 1.14 |
| 3295 | 0 | DE | B24B | B24B0037 | 38 | 70 | 40 | 30 | 1.33 |
| 3296 | 0 | US | H01L | H01L0021 | 6 | 26 | 14 | 2 | 7.00 |
| 3297 | 0 | JP | H01L | H01L0033 | 39 | 15 | 30 | 4.333333333 | 2.31 |
| 3298 | 0 | JP | H01L | H01L0021 | 1 | 56 | 2 | 3.333333333 | 0.20 |
| 3299 | 0 | US | H01L | H01L0021 | 29 | 6 | 3 | 1.2 | 0.50 |
| 3300 | 0 | JP | H01L | H01L0025 | 14 | 12 | 10 | 1.5 | 3.33 |
| 3301 | 0 | US | H01L | H01L0023 | 22 | 16 | 5 | 1 | 1.00 |
| 3302 | 0 | US | G02B | G02B0027 | 9 | 6 | 14 | 1.333333333 | 3.50 |
| 3303 | 0 | CA | H01L | H01L0021 | 11 | 25 | 20 | 1.25 | 4.00 |
| 3304 | 0 | US | G09F | G09F0009 | 22 | 8 | 13 | 1.333333333 | 3.25 |
| 3305 | 0 | JP | H01L | H01L0033 | 25 | 25 | 77 | 1.2 | 12.83 |
| 3306 | 1 | CA | H01L | H01L0033 | 28 | 2 | 172 | 1 | 28.67 |
| 3307 | 0 | US | C23C | C23C0016 | 15 | 60 | 116 | 7.153846154 | 1.25 |
| 3308 | 0 | US | C09K | C09K0011 | 8 | 9 | 26 | 1.142857143 | 3.25 |
| 3309 | 0 | US | C23C | C23C0016 | 6 | 15 | 1 | 1 | 0.25 |
| 3310 | 0 | JP | H01L | H01L0033 | 7 | 11 | 24 | 1 | 12.00 |
| 3311 | 0 | JP | H01L | H01L0033 | 4 | 13 | 0 | 1 | 0.00 |
| 3312 | 0 | JP | H01L | H01L0033 | 19 | 4 | 16 | 1.4 | 2.29 |
| 3313 | 0 | DE | H01L | H01L0021 | 7 | 8 | 1 | 1 | 0.25 |
| 3314 | 0 | JP | H01L | H01L0023 | 5 | 3 | 9 | 1.857142857 | 0.69 |
| 3315 | 0 | JP | H01L | H01L0021 | 9 | 40 | 34 | 1.4 | 4.86 |
| 3316 | 0 | JP | H01L | H01L0021 | 20 | 8 | 65 | 1 | 32.50 |
| 3317 | 0 | JP | H01L | H01L0021 | 5 | 15 | 3 | 1.5 | 1.00 |
| 3318 | 0 | JP | H01L | H01L0033 | 12 | 13 | 28 | 1.5 | 9.33 |
| 3319 | 0 | JP | H01L | H01L0021 | 10 | 12 | 6 | 1 | 3.00 |
| 3320 | 0 | US | G01J | G01J0005 | 9 | 7 | 3 | 1.333333333 | 0.75 |
| 3321 | 0 | JP | H01L | H01L0025 | 16 | 8 | 8 | 1 | 4.00 |
| 3322 | 0 | DE | H01L | H01L0021 | 13 | 15 | 7 | 1 | 2.33 |
| 3323 | 0 | CA | H01L | H01L0033 | 23 | 18 | 66 | 1 | 11.00 |
| 3324 | 1 | US | F21K | F21K0099 | 8 | 11 | 233 | 1 | 116.50 |
| 3325 | 0 | JP | H01L | H01L0033 | 16 | 12 | 22 | 2.2 | 2.00 |
| 3326 | 0 | JP | H05B | H05B0033 | 4 | 2 | 7 | 1 | 2.33 |
| 3327 | 0 | US | C30B | C30B0025 | 59 | 32 | 32 | 1.2 | 5.33 |
| 3328 | 0 | US | H01J | H01J0009 | 22 | 32 | 53 | 1.333333333 | 13.25 |
| 3329 | 0 | JP | H01S | H01S0005 | 12 | 6 | 5 | 1.5 | 1.67 |
| 3330 | 0 | JP | H01L | H01L0021 | 8 | 26 | 25 | 1.333333333 | 3.13 |
| 3331 | 0 | JP | H01L | H01L0021 | 8 | 6 | 8 | 1.5 | 2.67 |
| 3332 | 0 | TW | H01L | H01L0021 | 11 | 4 | 5 | 1 | 2.50 |
| 3333 | 0 | CA | H01L | H01L0033 | 13 | 6 | 46 | 1.142857143 | 5.75 |
| 3334 | 0 | JP | H01L | H01L0033 | 10 | 23 | 14 | 2.166666667 | 1.08 |
| 3335 | 0 | JP | H01L | H01L0025 | 11 | 18 | 37 | 1 | 9.25 |
| 3336 | 0 | DE | B24B | B24B0037 | 51 | 54 | 26 | 30 | 0.87 |
| 3337 | 0 | JP | H01L | H01L0021 | 20 | 8 | 13 | 1 | 4.33 |
| 3338 | 0 | JP | H01L | H01L0021 | 26 | 42 | 40 | 2 | 10.00 |
| 3339 | 0 | US | C23F | C23F0004 | 12 | 10 | 12 | 1.75 | 1.71 |
| 3340 | 0 | US | F21S | F21S0008 | 17 | 40 | 61 | 1 | 20.33 |
| 3341 | 0 | SE | H01L | H01L0021 | 15 | 26 | 16 | 1.166666667 | 2.29 |
| 3342 | 0 | JP | H01L | H01L0033 | 3 | 14 | 4 | 1 | 2.00 |
| 3343 | 0 | JP | H01L | H01L0021 | 26 | 4 | 129 | 1 | 64.50 |
| 3344 | 0 | KR | H01L | H01L0021 | 9 | 7 | 131 | 1 | 26.20 |
| 3345 | 0 | CA | H01L | H01L0033 | 36 | 27 | 95 | 1 | 23.75 |
| 3346 | 0 | JP | H01L | H01L0033 | 8 | 3 | 9 | 1 | 4.50 |
| 3347 | 0 | US | C23F | C23F0004 | 18 | 8 | 18 | 1.142857143 | 2.25 |
| 3348 | 0 | US | C09K | C09K0011 | 10 | 2 | 9 | 1 | 3.00 |
| 3349 | 0 | DE | H01L | H01L0029 | 4 | 19 | 6 | 1.2 | 1.00 |
| 3350 | 0 | US | H01L | H01L0021 | 4 | 7 | 88 | 2 | 8.80 |
| 3351 | 0 | JP | C08G | C08G0059 | 16 | 10 | 0 | 1.666666667 | 0.00 |
| 3352 | 0 | JP | H01L | H01L0033 | 13 | 24 | 37 | 1.5 | 12.33 |
| 3353 | 0 | JP | H01L | H01L0021 | 2 | 59 | 3 | 3.333333333 | 0.30 |
| 3354 | 0 | JP | B32B | B32B0027 | 6 | 15 | 10 | 1.333333333 | 2.50 |
| 3355 | 0 | US | H01L | H01L0029 | 25 | 28 | 5 | 1.166666667 | 0.71 |
| 3356 | 0 | JP | H01L | H01L0033 | 13 | 9 | 12 | 1.5 | 4.00 |
| 3357 | 0 | JP | H01L | H01L0027 | 19 | 12 | 9 | 1 | 4.50 |
| 3358 | 0 | TW | H01L | H01L0023 | 14 | 2 | 6 | 2 | 3.00 |
| 3359 | 0 | JP | H01L | H01L0021 | 19 | 23 | 7 | 1 | 3.50 |
| 3360 | 0 | JP | G02B | G02B0006 | 7 | 7 | 4 | 1 | 1.33 |
| 3361 | 0 | DE | C08F | C08F0002 | 2 | 40 | 11 | 1.555555556 | 0.79 |
| 3362 | 0 | JP | H01L | H01L0021 | 14 | 6 | 90 | 1 | 45.00 |
| 3363 | 0 | US | G01R | G01R0001 | 37 | 27 | 158 | 12.71428571 | 1.78 |
| 3364 | 0 | JP | H01L | H01L0033 | 18 | 6 | 12 | 2 | 3.00 |
| 3365 | 0 | JP | H01L | H01L0021 | 17 | 6 | 2 | 1 | 1.00 |
| 3366 | 0 | JP | H01L | H01L0021 | 7 | 2 | 2 | 1 | 0.50 |
| 3367 | 0 | JP | B05D | B05D0001 | 2 | 15 | 5 | 2.25 | 0.56 |
| 3368 | 0 | US | H01L | H01L0021 | 22 | 14 | 2 | 1.333333333 | 0.50 |
| 3369 | 0 | TW | H01L | H01L0033 | 12 | 4 | 12 | 1.5 | 4.00 |
| 3370 | 0 | JP | H01L | H01L0021 | 14 | 2 | 1 | 1.5 | 0.33 |
| 3371 | 0 | JP | H01S | H01S0005 | 9 | 6 | 42 | 1 | 21.00 |
| 3372 | 0 | JP | H01L | H01L0023 | 10 | 8 | 11 | 1.25 | 2.20 |
| 3373 | 0 | JP | H01L | H01L0021 | 4 | 22 | 3 | 2.666666667 | 0.38 |
| 3374 | 0 | JP | H01L | H01L0021 | 16 | 14 | 13 | 1 | 4.33 |
| 3375 | 0 | CA | H01L | H01L0033 | 32 | 20 | 29 | 2.2 | 2.64 |
| 3376 | 0 | US | C30B | C30B0023 | 7 | 7 | 4 | 3 | 1.33 |
| 3377 | 0 | JP | H01L | H01L0033 | 16 | 4 | 1 | 1 | 0.25 |
| 3378 | 0 | JP | C30B | C30B0011 | 23 | 12 | 3 | 1 | 0.75 |
| 3379 | 0 | JP | H01L | H01L0033 | 8 | 17 | 7 | 4.333333333 | 0.54 |
| 3380 | 0 | JP | H01L | H01L0029 | 2 | 6 | 10 | 2.666666667 | 1.25 |
| 3381 | 0 | JP | H01S | H01S0005 | 4 | 13 | 17 | 3.5 | 2.43 |
| 3382 | 0 | JP | H01L | H01L0033 | 2 | 6 | 25 | 2 | 6.25 |
| 3383 | 0 | US | C23C | C23C0016 | 2 | 14 | 10 | 1 | 2.00 |
| 3384 | 0 | AN | H01L | H01L0021 | 13 | 94 | 29 | 1.941176471 | 0.88 |
| 3385 | 0 | JP | H01L | H01L0033 | 7 | 18 | 38 | 1.5 | 12.67 |
| 3386 | 0 | JP | F21V | F21V0005 | 13 | 11 | 35 | 1.75 | 5.00 |
| 3387 | 0 | US | B41M | B41M0005 | 23 | 19 | 77 | 1.375 | 7.00 |
| 3388 | 0 | JP | H01L | H01L0021 | 3 | 5 | 3 | 1 | 1.50 |
| 3389 | 0 | JP | H01L | H01L0021 | 23 | 6 | 25 | 1.5 | 8.33 |
| 3390 | 0 | CA | H01L | H01L0023 | 13 | 12 | 8 | 2 | 4.00 |
| 3391 | 0 | JP | H01L | H01L0021 | 12 | 8 | 4 | 1.5 | 1.33 |
| 3392 | 0 | US | H01L | H01L0033 | 25 | 20 | 61 | 1 | 15.25 |
| 3393 | 0 | US | C08L | C08L0023 | 3 | 1 | 11 | 1.142857143 | 1.38 |
| 3394 | 0 | JP | H01L | H01L0021 | 6 | 41 | 50 | 2.2 | 4.55 |
| 3395 | 0 | JP | H01L | H01L0033 | 10 | 5 | 23 | 4.5 | 2.56 |
| 3396 | 0 | JP | H01L | H01L0021 | 7 | 29 | 11 | 1 | 3.67 |
| 3397 | 0 | JP | H01L | H01L0027 | 33 | 6 | 2 | 1 | 0.50 |
| 3398 | 0 | JP | H01L | H01L0021 | 15 | 4 | 101 | 4 | 12.63 |
| 3399 | 0 | JP | H01L | H01L0033 | 7 | 6 | 2 | 1.5 | 0.67 |
| 3400 | 0 | US | H01L | H01L0025 | 22 | 9 | 124 | 1 | 41.33 |
| 3401 | 0 | JP | H01L | H01L0021 | 8 | 14 | 2 | 1 | 0.67 |
| 3402 | 0 | JP | C09K | C09K0011 | 11 | 3 | 9 | 1 | 3.00 |
| 3403 | 0 | KR | H01S | H01S0005 | 6 | 7 | 8 | 1.5 | 2.67 |
| 3404 | 0 | JP | H01L | H01L0021 | 16 | 47 | 22 | 1 | 5.50 |
| 3405 | 0 | JP | H01L | H01L0033 | 18 | 13 | 37 | 1 | 18.50 |
| 3406 | 0 | US | G02B | G02B0027 | 15 | 12 | 21 | 1.333333333 | 5.25 |
| 3407 | 0 | US | C30B | C30B0023 | 3 | 1 | 8 | 1 | 1.33 |
| 3408 | 0 | JP | H01L | H01L0021 | 19 | 12 | 7 | 2 | 3.50 |
| 3409 | 1 | US | H01L | H01L0033 | 16 | 9 | 86 | 3 | 5.73 |
| 3410 | 0 | JP | H01L | H01L0021 | 5 | 7 | 22 | 1.5 | 7.33 |
| 3411 | 0 | JP | H01L | H01L0021 | 21 | 9 | 5 | 1 | 2.50 |
| 3412 | 0 | US | H01J | H01J0001 | 15 | 30 | 12 | 1.25 | 2.40 |
| 3413 | 0 | JP | B24B | B24B0037 | 27 | 13 | 12 | 3.5 | 0.34 |
| 3414 | 0 | JP | H01L | H01L0021 | 16 | 13 | 11 | 1.333333333 | 2.75 |
| 3415 | 0 | JP | H01L | H01L0021 | 48 | 13 | 5 | 1 | 1.67 |
| 3416 | 0 | JP | H01L | H01L0033 | 9 | 3 | 0 | 2.2 | 0.00 |
| 3417 | 0 | KR | H01L | H01L0021 | 4 | 6 | 8 | 1.25 | 1.60 |
| 3418 | 0 | US | H01L | H01L0021 | 16 | 20 | 8 | 1 | 2.00 |
| 3419 | 0 | US | H01L | H01L0023 | 7 | 18 | 44 | 2 | 22.00 |
| 3420 | 0 | JP | G09G | G09G0003 | 21 | 5 | 12 | 1 | 6.00 |
| 3421 | 0 | JP | H01L | H01L0029 | 19 | 9 | 22 | 1 | 7.33 |
| 3422 | 0 | JP | H01L | H01L0029 | 3 | 5 | 6 | 1 | 1.50 |
| 3423 | 0 | JP | H01L | H01L0023 | 2 | 8 | 38 | 1.25 | 7.60 |
| 3424 | 0 | CA | H01L | H01L0033 | 18 | 16 | 71 | 1.5 | 7.89 |
| 3425 | 0 | JP | H01L | H01L0033 | 3 | 8 | 3 | 2.333333333 | 0.43 |
| 3426 | 0 | US | B41J | B41J0002 | 8 | 11 | 10 | 1.25 | 2.00 |
| 3427 | 0 | JP | H01L | H01L0021 | 18 | 31 | 52 | 1 | 17.33 |
| 3428 | 0 | US | H01L | H01L0023 | 7 | 10 | 56 | 1 | 18.67 |
| 3429 | 0 | JP | B32B | B32B0015 | 26 | 18 | 4 | 3 | 0.33 |
| 3430 | 0 | US | G02B | G02B0006 | 16 | 8 | 56 | 1.166666667 | 8.00 |
| 3431 | 0 | JP | H01S | H01S0005 | 23 | 9 | 15 | 1 | 7.50 |
| 3432 | 0 | JP | B32B | B32B0015 | 11 | 31 | 1 | 3 | 0.08 |
| 3433 | 0 | US | C30B | C30B0001 | 8 | 3 | 2 | 2 | 1.00 |
| 3434 | 0 | JP | H01L | H01L0021 | 9 | 6 | 7 | 1 | 3.50 |
| 3435 | 0 | JP | C30B | C30B0023 | 26 | 26 | 5 | 1 | 1.25 |
| 3436 | 0 | JP | H05K | H05K0001 | 10 | 20 | 58 | 1 | 29.00 |
| 3437 | 0 | JP | H01L | H01L0033 | 13 | 7 | 16 | 1.5 | 5.33 |
| 3438 | 0 | CA | H05K | H05K0003 | 23 | 23 | 8 | 1.25 | 1.60 |
| 3439 | 0 | US | G03F | G03F0001 | 16 | 9 | 33 | 1 | 11.00 |
| 3440 | 0 | US | H01L | H01L0021 | 15 | 20 | 7 | 1 | 1.75 |
| 3441 | 0 | US | C09K | C09K0011 | 2 | 11 | 1 | 1.142857143 | 0.13 |
| 3442 | 0 | US | C30B | C30B0019 | 6 | 9 | 10 | 1.5 | 1.67 |
| 3443 | 0 | JP | H01L | H01L0033 | 1 | 28 | 3 | 1.5 | 1.00 |
| 3444 | 0 | JP | H01L | H01L0033 | 24 | 88 | 35 | 3.4 | 2.06 |
| 3445 | 0 | JP | C23C | C23C0014 | 30 | 62 | 3 | 1 | 0.60 |
| 3446 | 0 | JP | H01L | H01L0021 | 5 | 10 | 8 | 1.25 | 1.60 |
| 3447 | 0 | JP | C23C | C23C0018 | 8 | 1 | 1 | 1.166666667 | 0.14 |
| 3448 | 0 | JP | H01L | H01L0033 | 29 | 9 | 13 | 1 | 6.50 |
| 3449 | 0 | US | H05B | H05B0033 | 1 | 2 | 1 | 1.111111111 | 0.10 |
| 3450 | 0 | US | H01L | H01L0021 | 22 | 65 | 19 | 1.5 | 6.33 |
| 3451 | 0 | VG | G03F | G03F0007 | 19 | 34 | 13 | 1 | 1.86 |
| 3452 | 0 | US | C23C | C23C0014 | 20 | 38 | 32 | 1 | 10.67 |
| 3453 | 0 | JP | H01L | H01L0021 | 6 | 5 | 4 | 1.5 | 1.33 |
| 3454 | 0 | US | B41J | B41J0002 | 7 | 16 | 8 | 1.25 | 1.60 |
| 3455 | 0 | JP | G02F | G02F0001 | 27 | 6 | 35 | 1.5 | 11.67 |
| 3456 | 0 | US | H01S | H01S0003 | 20 | 14 | 7 | 3 | 0.58 |
| 3457 | 0 | JP | C09J | C09J0009 | 5 | 13 | 6 | 1.2 | 1.00 |
| 3458 | 0 | US | H01L | H01L0021 | 18 | 15 | 14 | 1.857142857 | 1.08 |
| 3459 | 0 | US | C30B | C30B0025 | 34 | 9 | 107 | 1 | 17.83 |
| 3460 | 0 | US | H01L | H01L0021 | 11 | 9 | 47 | 2.8 | 3.36 |
| 3461 | 0 | US | C07F | C07F0003 | 4 | 16 | 19 | 1.333333333 | 4.75 |
| 3462 | 0 | US | H01L | H01L0033 | 14 | 14 | 8 | 1 | 4.00 |
| 3463 | 0 | KR | H01L | H01L0033 | 23 | 7 | 2 | 1.5 | 0.33 |
| 3464 | 0 | JP | H01L | H01L0033 | 6 | 16 | 15 | 5.5 | 1.36 |
| 3465 | 0 | DE | H01L | H01L0033 | 24 | 15 | 46 | 3.333333333 | 2.30 |
| 3466 | 0 | JP | H01L | H01L0033 | 5 | 9 | 7 | 2.333333333 | 1.00 |
| 3467 | 0 | US | C30B | C30B0023 | 21 | 5 | 66 | 2 | 33.00 |
| 3468 | 0 | US | B32B | B32B0005 | 19 | 24 | 19 | 1.8 | 2.11 |
| 3469 | 0 | JP | H01L | H01L0021 | 11 | 33 | 10 | 1.8 | 1.11 |
| 3470 | 0 | US | H01L | H01L0033 | 15 | 12 | 34 | 1 | 8.50 |
| 3471 | 0 | JP | H01L | H01L0021 | 9 | 18 | 10 | 1.5 | 3.33 |
| 3472 | 0 | JP | H01L | H01L0033 | 21 | 16 | 19 | 2 | 4.75 |
| 3473 | 0 | JP | H01L | H01L0033 | 32 | 8 | 4 | 1 | 2.00 |
| 3474 | 0 | JP | H01S | H01S0005 | 8 | 6 | 15 | 1.5 | 2.50 |
| 3475 | 0 | JP | H01L | H01L0021 | 1 | 20 | 6 | 1.8 | 0.67 |
| 3476 | 0 | JP | H01L | H01L0033 | 2 | 18 | 4 | 2 | 1.00 |
| 3477 | 0 | US | H01L | H01L0021 | 65 | 70 | 13 | 1.5 | 4.33 |
| 3478 | 0 | US | G09F | G09F0009 | 35 | 4 | 28 | 1 | 7.00 |
| 3479 | 0 | CA | G09F | G09F0013 | 11 | 16 | 36 | 1.2 | 6.00 |
| 3480 | 0 | TW | B41J | B41J0002 | 16 | 1 | 35 | 1 | 7.00 |
| 3481 | 0 | JP | H01L | H01L0033 | 18 | 2 | 14 | 1.5 | 4.67 |
| 3482 | 0 | KR | H01L | H01L0023 | 24 | 18 | 34 | 1.571428571 | 3.09 |
| 3483 | 0 | DE | H01L | H01L0033 | 13 | 13 | 8 | 1 | 1.60 |
| 3484 | 0 | TW | H01L | H01L0023 | 11 | 3 | 13 | 1.5 | 4.33 |
| 3485 | 0 | TW | H01L | H01L0033 | 11 | 7 | 1 | 1 | 0.50 |
| 3486 | 0 | TW | H01L | H01L0023 | 9 | 6 | 6 | 2 | 3.00 |
| 3487 | 0 | JP | H01L | H01L0021 | 7 | 10 | 3 | 2 | 0.75 |
| 3488 | 0 | JP | G11B | G11B0007 | 10 | 3 | 32 | 1.5 | 10.67 |
| 3489 | 0 | US | H01L | H01L0021 | 26 | 22 | 54 | 1 | 27.00 |
| 3490 | 0 | US | H01L | H01L0027 | 16 | 18 | 43 | 1 | 14.33 |
| 3491 | 0 | JP | B60Q | B60Q0003 | 18 | 3 | 16 | 1.5 | 5.33 |
| 3492 | 0 | FR | C30B | C30B0011 | 15 | 25 | 3 | 1.875 | 0.20 |
| 3493 | 0 | JP | H01L | H01L0033 | 12 | 9 | 2 | 1.25 | 0.40 |
| 3494 | 0 | US | C30B | C30B0023 | 3 | 13 | 9 | 3 | 3.00 |
| 3495 | 0 | JP | H01L | H01L0021 | 19 | 30 | 5 | 1 | 1.67 |
| 3496 | 0 | US | C30B | C30B0023 | 10 | 128 | 46 | 3.333333333 | 2.30 |
| 3497 | 0 | JP | H01L | H01L0033 | 3 | 14 | 13 | 4.333333333 | 1.00 |
| 3498 | 0 | JP | G09F | G09F0009 | 27 | 4 | 57 | 1 | 28.50 |
| 3499 | 0 | MD | H01S | H01S0005 | 20 | 15 | 15 | 1 | 5.00 |
| 3500 | 0 | JP | H01L | H01L0033 | 8 | 13 | 16 | 1.5 | 5.33 |
| 3501 | 0 | US | G09F | G09F0009 | 18 | 14 | 46 | 1 | 9.20 |
| 3502 | 0 | US | H01L | H01L0021 | 5 | 23 | 18 | 1.4 | 1.29 |
| 3503 | 0 | JP | H01L | H01L0033 | 8 | 3 | 0 | 1 | 0.00 |
| 3504 | 0 | JP | H01L | H01L0023 | 4 | 15 | 4 | 1.833333333 | 0.36 |
| 3505 | 0 | JP | H01L | H01L0021 | 39 | 82 | 101 | 1.6 | 12.63 |
| 3506 | 0 | JP | H01L | H01L0021 | 10 | 17 | 5 | 1 | 2.50 |
| 3507 | 0 | US | H05B | H05B0033 | 21 | 1 | 15 | 1 | 3.00 |
| 3508 | 0 | KR | H01L | H01L0021 | 5 | 3 | 3 | 1 | 1.00 |
| 3509 | 0 | JP | C30B | C30B0025 | 14 | 11 | 60 | 1.142857143 | 7.50 |
| 3510 | 0 | US | H01L | H01L0021 | 21 | 29 | 42 | 1.5 | 7.00 |
| 3511 | 0 | JP | H01L | H01L0033 | 2 | 11 | 0 | 1.333333333 | 0.00 |
| 3512 | 0 | JP | H01L | H01L0033 | 7 | 2 | 8 | 1.5 | 2.67 |
| 3513 | 0 | JP | H01L | H01L0021 | 12 | 8 | 5 | 1.333333333 | 1.25 |
| 3514 | 0 | JP | F21V | F21V0008 | 9 | 63 | 19 | 1.5 | 3.17 |
| 3515 | 0 | US | H01L | H01L0025 | 37 | 27 | 238 | 1 | 39.67 |
| 3516 | 0 | JP | H01L | H01L0033 | 6 | 7 | 4 | 1.5 | 1.33 |
| 3517 | 0 | KR | H01L | H01L0021 | 11 | 5 | 26 | 1 | 13.00 |
| 3518 | 0 | JP | H01L | H01L0021 | 13 | 26 | 21 | 1 | 4.20 |
| 3519 | 0 | CA | H01L | H01L0033 | 10 | 20 | 40 | 1.142857143 | 5.00 |
| 3520 | 0 | DE | C08K | C08K0003 | 22 | 57 | 22 | 1 | 3.67 |
| 3521 | 0 | CA | H01L | H01L0033 | 57 | 33 | 30 | 1 | 15.00 |
| 3522 | 0 | JP | H01L | H01L0029 | 7 | 11 | 10 | 2.666666667 | 1.25 |
| 3523 | 0 | JP | H01L | H01L0033 | 6 | 4 | 9 | 1.5 | 3.00 |
| 3524 | 0 | KR | H01L | H01L0033 | 6 | 5 | 7 | 1 | 3.50 |
| 3525 | 0 | JP | H01L | H01L0021 | 5 | 27 | 12 | 1.333333333 | 3.00 |
| 3526 | 0 | US | H01L | H01L0033 | 15 | 37 | 87 | 1.5 | 29.00 |
| 3527 | 0 | JP | H01L | H01L0033 | 5 | 19 | 32 | 3 | 5.33 |
| 3528 | 0 | US | C09K | C09K0011 | 8 | 3 | 1 | 1.142857143 | 0.13 |
| 3529 | 0 | JP | C30B | C30B0029 | 17 | 3 | 6 | 1.2 | 1.00 |
| 3530 | 0 | JP | H01L | H01L0021 | 18 | 16 | 18 | 3.333333333 | 1.80 |
| 3531 | 0 | JP | H01L | H01L0021 | 10 | 15 | 4 | 1.333333333 | 1.00 |
| 3532 | 0 | JP | H01L | H01L0021 | 21 | 8 | 9 | 1.4 | 1.29 |
| 3533 | 0 | JP | H01L | H01L0021 | 4 | 25 | 24 | 1.2 | 4.00 |
| 3534 | 0 | TW | H01L | H01L0021 | 16 | 12 | 4 | 1.5 | 0.67 |
| 3535 | 0 | US | H01L | H01L0021 | 5 | 24 | 54 | 1.25 | 10.80 |
| 3536 | 0 | TW | H01L | H01L0033 | 11 | 13 | 16 | 1 | 5.33 |
| 3537 | 0 | JP | C30B | C30B0025 | 7 | 7 | 2 | 1 | 1.00 |
| 3538 | 0 | US | B23K | B23K0003 | 10 | 21 | 19 | 3 | 6.33 |
| 3539 | 0 | DE | H01L | H01L0031 | 8 | 24 | 4 | 3 | 0.67 |
| 3540 | 0 | US | G09F | G09F0009 | 15 | 3 | 5 | 1.333333333 | 1.25 |
| 3541 | 0 | KR | H01L | H01L0023 | 18 | 8 | 39 | 1 | 39.00 |
| 3542 | 0 | JP | H01L | H01L0033 | 20 | 9 | 7 | 1 | 3.50 |
| 3543 | 0 | JP | H01L | H01L0033 | 16 | 5 | 0 | 1 | 0.00 |
| 3544 | 0 | JP | H01L | H01L0021 | 11 | 5 | 4 | 1 | 2.00 |
| 3545 | 0 | JP | G02F | G02F0001 | 7 | 6 | 9 | 1.166666667 | 1.29 |
| 3546 | 0 | JP | H01L | H01L0023 | 10 | 10 | 2 | 1.25 | 0.40 |
| 3547 | 0 | JP | H01L | H01L0021 | 31 | 42 | 9 | 3 | 1.50 |
| 3548 | 0 | JP | C08G | C08G0059 | 16 | 34 | 5 | 1.5 | 1.67 |
| 3549 | 0 | JP | G02F | G02F0001 | 10 | 18 | 8 | 1 | 2.00 |
| 3550 | 0 | JP | H01L | H01L0033 | 12 | 20 | 3 | 1 | 1.00 |
| 3551 | 0 | US | C08G | C08G0008 | 8 | 66 | 8 | 1 | 1.60 |
| 3552 | 0 | JP | H01L | H01L0031 | 13 | 10 | 1 | 1 | 0.50 |
| 3553 | 0 | US | H01L | H01L0029 | 23 | 51 | 260 | 1.333333333 | 32.50 |
| 3554 | 0 | DE | H01L | H01L0021 | 11 | 12 | 1 | 1 | 0.20 |
| 3555 | 0 | CA | C23C | C23C0016 | 13 | 17 | 29 | 1 | 5.80 |
| 3556 | 0 | TW | H01L | H01L0033 | 24 | 11 | 31 | 1 | 15.50 |
| 3557 | 0 | JP | H01L | H01L0021 | 6 | 4 | 5 | 1 | 2.50 |
| 3558 | 0 | US | F21K | F21K0002 | 58 | 22 | 62 | 1.166666667 | 8.86 |
| 3559 | 0 | JP | H01S | H01S0005 | 7 | 10 | 4 | 1.5 | 1.33 |
| 3560 | 0 | TW | H01L | H01L0033 | 47 | 3 | 20 | 1 | 20.00 |
| 3561 | 0 | JP | H01L | H01L0033 | 10 | 7 | 7 | 1.5 | 2.33 |
| 3562 | 0 | JP | B60Q | B60Q0003 | 11 | 8 | 22 | 2 | 3.67 |
| 3563 | 0 | US | C07F | C07F0005 | 5 | 4 | 77 | 1 | 19.25 |
| 3564 | 0 | IE | F21V | F21V0005 | 14 | 10 | 14 | 1 | 3.50 |
| 3565 | 0 | US | C30B | C30B0025 | 26 | 19 | 170 | 2 | 85.00 |
| 3566 | 0 | US | H01L | H01L0021 | 33 | 19 | 5 | 2 | 2.50 |
| 3567 | 1 | US | C30B | C30B0023 | 21 | 137 | 31 | 3.333333333 | 1.55 |
| 3568 | 0 | JP | H01L | H01L0021 | 11 | 5 | 5 | 1 | 2.50 |
| 3569 | 0 | US | H01L | H01L0033 | 60 | 24 | 111 | 4 | 27.75 |
| 3570 | 0 | JP | H01L | H01L0021 | 27 | 5 | 7 | 1.4 | 1.00 |
| 3571 | 0 | JP | H01L | H01L0029 | 7 | 1 | 0 | 1.5 | 0.00 |
| 3572 | 0 | US | H01L | H01L0021 | 1 | 37 | 56 | 1.5 | 6.22 |
| 3573 | 0 | TW | H01L | H01L0033 | 7 | 14 | 17 | 1 | 4.25 |
| 3574 | 0 | JP | H01L | H01L0033 | 25 | 8 | 23 | 1.5 | 7.67 |
| 3575 | 0 | US | H05B | H05B0033 | 8 | 20 | 7 | 1.333333333 | 0.88 |
| 3576 | 0 | JP | B23K | B23K0035 | 7 | 9 | 1 | 1.5 | 0.33 |
| 3577 | 0 | TW | H01L | H01L0033 | 20 | 1 | 7 | 1 | 2.33 |
| 3578 | 0 | JP | H01L | H01L0021 | 13 | 24 | 2 | 1 | 0.40 |
| 3579 | 1 | DE | H01L | H01L0031 | 23 | 28 | 44 | 2 | 5.50 |
| 3580 | 0 | US | G09F | G09F0009 | 41 | 7 | 34 | 1 | 8.50 |
| 3581 | 0 | JP | H01L | H01L0021 | 11 | 50 | 10 | 2.75 | 0.91 |
| 3582 | 0 | JP | H01L | H01L0033 | 31 | 19 | 5 | 1 | 2.50 |
| 3583 | 0 | TW | C30B | C30B0029 | 21 | 1 | 8 | 1 | 2.67 |
| 3584 | 0 | TW | H01L | H01L0021 | 17 | 8 | 25 | 1.2 | 4.17 |
| 3585 | 0 | US | H05B | H05B0033 | 29 | 46 | 378 | 2.761904762 | 6.52 |
| 3586 | 0 | JP | H01S | H01S0005 | 15 | 2 | 1 | 1 | 0.33 |
| 3587 | 0 | JP | H01L | H01L0033 | 17 | 7 | 5 | 1 | 1.67 |
| 3588 | 0 | JP | H01L | H01L0025 | 4 | 9 | 17 | 1.4 | 2.43 |
| 3589 | 0 | JP | H01L | H01L0023 | 23 | 6 | 3 | 1.25 | 0.60 |
| 3590 | 0 | TW | H01L | H01L0021 | 5 | 3 | 3 | 1 | 1.50 |
| 3591 | 0 | JP | H01L | H01L0021 | 10 | 4 | 12 | 2 | 3.00 |
| 3592 | 0 | DE | H01L | H01L0033 | 8 | 15 | 15 | 3.333333333 | 0.75 |
| 3593 | 0 | US | H01L | H01L0033 | 17 | 4 | 31 | 1.666666667 | 6.20 |
| 3594 | 0 | JP | H01L | H01L0033 | 8 | 36 | 5 | 4.6 | 0.22 |
| 3595 | 0 | US | H01L | H01L0033 | 3 | 38 | 54 | 1 | 27.00 |
| 3596 | 0 | JP | H01S | H01S0005 | 7 | 9 | 1 | 1.5 | 0.33 |
| 3597 | 0 | JP | C23C | C23C0016 | 10 | 7 | 6 | 1.5 | 2.00 |
| 3598 | 0 | TW | H01L | H01L0033 | 15 | 14 | 1 | 1 | 0.50 |
| 3599 | 0 | JP | H01L | H01L0033 | 37 | 99 | 0 | 3.4 | 0.00 |
| 3600 | 0 | JP | H01L | H01L0033 | 37 | 99 | 1 | 3.4 | 0.06 |
| 3601 | 0 | US | C30B | C30B0025 | 52 | 82 | 35 | 1.2 | 5.83 |
| 3602 | 0 | JP | H01L | H01L0021 | 7 | 15 | 0 | 1 | 0.00 |
| 3603 | 0 | DE | H01L | H01L0021 | 22 | 9 | 4 | 1 | 1.00 |
| 3604 | 0 | JP | C30B | C30B0029 | 8 | 11 | 0 | 1 | 0.00 |
| 3605 | 0 | TW | H01L | H01L0033 | 7 | 6 | 13 | 1.5 | 2.17 |
| 3606 | 0 | TW | F21S | F21S0004 | 2 | 3 | 4 | 1 | 4.00 |
| 3607 | 0 | US | H01L | H01L0021 | 17 | 19 | 152 | 1 | 76.00 |
| 3608 | 0 | JP | C08L | C08L0063 | 16 | 6 | 7 | 1 | 3.50 |
| 3609 | 0 | US | H01L | H01L0033 | 19 | 9 | 7 | 1 | 2.33 |
| 3610 | 0 | JP | H01L | H01L0021 | 1 | 19 | 105 | 1.833333333 | 9.55 |
| 3611 | 0 | JP | H01L | H01L0023 | 12 | 25 | 14 | 2 | 1.40 |
| 3612 | 0 | US | C23F | C23F0004 | 19 | 8 | 5 | 1.142857143 | 0.63 |
| 3613 | 0 | JP | G03H | G03H0001 | 54 | 2 | 7 | 3 | 1.17 |
| 3614 | 0 | KR | H01L | H01L0033 | 6 | 3 | 19 | 1 | 4.75 |
| 3615 | 0 | US | C07F | C07F0003 | 20 | 3 | 18 | 1.333333333 | 4.50 |
| 3616 | 0 | US | H01L | H01L0033 | 21 | 4 | 27 | 1.2 | 4.50 |
| 3617 | 0 | US | H01S | H01S0005 | 49 | 14 | 33 | 1.333333333 | 8.25 |
| 3618 | 0 | JP | H01L | H01L0033 | 23 | 6 | 13 | 1 | 4.33 |
| 3619 | 0 | US | G02B | G02B0025 | 20 | 13 | 11 | 1 | 3.67 |
| 3620 | 0 | TW | H01L | H01L0021 | 32 | 2 | 59 | 1 | 19.67 |
| 3621 | 0 | JP | H01J | H01J0037 | 8 | 35 | 6 | 8.5 | 0.35 |
| 3622 | 0 | JP | H01L | H01L0033 | 7 | 24 | 0 | 1 | 0.00 |
| 3623 | 0 | TW | H01L | H01L0021 | 20 | 8 | 6 | 1 | 2.00 |
| 3624 | 0 | TW | H01L | H01L0023 | 7 | 8 | 12 | 1 | 12.00 |
| 3625 | 0 | JP | H01S | H01S0005 | 34 | 8 | 5 | 1 | 1.67 |
| 3626 | 0 | CA | H01L | H01L0033 | 24 | 15 | 18 | 1 | 4.50 |
| 3627 | 0 | JP | H01L | H01L0051 | 2 | 26 | 25 | 1 | 12.50 |
| 3628 | 0 | US | H01L | H01L0021 | 12 | 17 | 2 | 1.857142857 | 0.15 |
| 3629 | 0 | SG | H01L | H01L0023 | 28 | 8 | 52 | 1 | 26.00 |
| 3630 | 0 | US | C08F | C08F0014 | 3 | 83 | 52 | 2.666666667 | 1.63 |
| 3631 | 0 | JP | B23K | B23K0020 | 4 | 19 | 9 | 1.25 | 1.80 |
| 3632 | 0 | JP | G09F | G09F0009 | 3 | 5 | 36 | 1 | 9.00 |
| 3633 | 0 | JP | H01L | H01L0033 | 13 | 10 | 94 | 1.5 | 31.33 |
| 3634 | 0 | US | H01L | H01L0021 | 12 | 22 | 14 | 1 | 3.50 |
| 3635 | 0 | JP | H01L | H01L0021 | 16 | 9 | 11 | 2.5 | 2.20 |
| 3636 | 0 | CA | H01L | H01L0021 | 34 | 11 | 22 | 1.125 | 2.44 |
| 3637 | 0 | CA | H01L | H01L0025 | 25 | 2 | 128 | 1 | 32.00 |
| 3638 | 0 | JP | H01L | H01L0033 | 7 | 46 | 15 | 2.166666667 | 1.15 |
| 3639 | 0 | JP | G09F | G09F0013 | 11 | 9 | 30 | 1 | 15.00 |
| 3640 | 0 | US | C30B | C30B0029 | 22 | 3 | 101 | 1 | 50.50 |
| 3641 | 0 | CA | C09K | C09K0011 | 27 | 33 | 43 | 1 | 8.60 |
| 3642 | 0 | JP | H01L | H01L0023 | 23 | 29 | 10 | 1 | 1.43 |
| 3643 | 0 | US | F21K | F21K0007 | 41 | 7 | 10 | 1 | 2.50 |
| 3644 | 0 | JP | H01L | H01L0023 | 2 | 5 | 11 | 1.333333333 | 1.38 |
| 3645 | 0 | CA | H01L | H01L0033 | 43 | 28 | 18 | 1 | 3.60 |
| 3646 | 0 | TW | C30B | C30B0029 | 10 | 10 | 2 | 1.5 | 0.67 |
| 3647 | 0 | CA | F21S | F21S0002 | 8 | 21 | 79 | 1 | 26.33 |
| 3648 | 0 | TW | F21S | F21S0004 | 8 | 6 | 14 | 1 | 14.00 |
| 3649 | 0 | US | H01L | H01L0021 | 8 | 24 | 8 | 1.4 | 1.14 |
| 3650 | 0 | JP | H01L | H01L0021 | 16 | 4 | 1 | 1 | 0.50 |
| 3651 | 0 | US | H05B | H05B0033 | 18 | 2 | 212 | 1.2 | 35.33 |
| 3652 | 0 | US | H01L | H01L0021 | 16 | 3 | 180 | 1.333333333 | 45.00 |
| 3653 | 0 | JP | H01L | H01L0029 | 30 | 6 | 16 | 2.5 | 3.20 |
| 3654 | 0 | JP | H01S | H01S0005 | 7 | 15 | 15 | 1.25 | 3.00 |
| 3655 | 0 | KR | C30B | C30B0025 | 5 | 13 | 14 | 1 | 7.00 |
| 3656 | 0 | JP | C30B | C30B0025 | 7 | 5 | 20 | 1 | 10.00 |
| 3657 | 0 | US | C09K | C09K0011 | 4 | 13 | 2 | 1.333333333 | 0.25 |
| 3658 | 0 | JP | H01L | H01L0021 | 20 | 5 | 13 | 1.25 | 2.60 |
| 3659 | 0 | JP | H01L | H01L0033 | 4 | 11 | 12 | 2 | 2.00 |
| 3660 | 0 | CA | H01L | H01L0025 | 16 | 52 | 39 | 1.428571429 | 3.90 |
| 3661 | 0 | TW | H01L | H01L0033 | 26 | 8 | 14 | 1.5 | 4.67 |
| 3662 | 0 | JP | H01L | H01L0021 | 10 | 5 | 8 | 1 | 4.00 |
| 3663 | 0 | CA | H01L | H01L0033 | 43 | 24 | 43 | 1.125 | 4.78 |
| 3664 | 0 | KR | H01L | H01L0023 | 17 | 11 | 12 | 1.333333333 | 3.00 |
| 3665 | 0 | JP | H01L | H01L0021 | 13 | 21 | 0 | 1.166666667 | 0.00 |
| 3666 | 0 | JP | H01L | H01L0033 | 49 | 7 | 1 | 1 | 0.50 |
| 3667 | 0 | JP | H01L | H01L0021 | 12 | 2 | 13 | 1 | 6.50 |
| 3668 | 0 | US | H05B | H05B0033 | 15 | 10 | 141 | 1 | 23.50 |
| 3669 | 0 | JP | G03C | G03C0005 | 17 | 9 | 0 | 1 | 0.00 |
| 3670 | 0 | TW | H01L | H01L0023 | 20 | 2 | 0 | 1 | 0.00 |
| 3671 | 0 | JP | H01L | H01L0021 | 14 | 10 | 7 | 1 | 3.50 |
| 3672 | 0 | JP | H01L | H01L0021 | 11 | 4 | 7 | 1.5 | 2.33 |
| 3673 | 0 | TW | H01L | H01L0033 | 23 | 10 | 45 | 1 | 45.00 |
| 3674 | 0 | US | C25F | C25F0003 | 11 | 26 | 19 | 1 | 6.33 |
| 3675 | 0 | SG | H01L | H01L0021 | 9 | 28 | 19 | 1 | 6.33 |
| 3676 | 0 | JP | H01S | H01S0005 | 6 | 6 | 1 | 1 | 0.25 |
| 3677 | 0 | JP | H01L | H01L0033 | 6 | 14 | 0 | 1.5 | 0.00 |
| 3678 | 0 | CA | H01S | H01S0005 | 9 | 14 | 22 | 2 | 11.00 |
| 3679 | 0 | DE | C30B | C30B0029 | 14 | 12 | 0 | 1.166666667 | 0.00 |
| 3680 | 0 | JP | H01S | H01S0005 | 13 | 17 | 0 | 1 | 0.00 |
| 3681 | 0 | KR | C30B | C30B0025 | 17 | 23 | 16 | 1 | 5.33 |
| 3682 | 0 | JP | H01L | H01L0033 | 11 | 18 | 12 | 1 | 3.00 |
| 3683 | 0 | JP | H01L | H01L0051 | 63 | 26 | 40 | 1 | 20.00 |
| 3684 | 0 | JP | H01L | H01L0023 | 11 | 23 | 11 | 1.5 | 3.67 |
| 3685 | 1 | US | H01L | H01L0033 | 22 | 14 | 221 | 1.4 | 31.57 |
| 3686 | 0 | US | H01L | H01L0021 | 15 | 5 | 35 | 1.25 | 7.00 |
| 3687 | 0 | US | H01L | H01L0023 | 12 | 28 | 21 | 2 | 10.50 |
| 3688 | 0 | TW | H01L | H01L0021 | 5 | 7 | 5 | 1.25 | 1.00 |
| 3689 | 0 | JP | C30B | C30B0023 | 8 | 20 | 6 | 1 | 3.00 |
| 3690 | 0 | JP | H01L | H01L0021 | 8 | 6 | 8 | 1 | 2.67 |
| 3691 | 0 | JP | H01L | H01L0029 | 8 | 17 | 6 | 1 | 3.00 |
| 3692 | 0 | JP | H01L | H01L0031 | 74 | 11 | 9 | 2 | 2.25 |
| 3693 | 0 | JP | H01L | H01L0025 | 22 | 10 | 4 | 2.2 | 0.36 |
| 3694 | 0 | JP | H01L | H01L0023 | 13 | 1 | 26 | 1 | 13.00 |
| 3695 | 0 | US | H01L | H01L0033 | 13 | 20 | 155 | 1.25 | 31.00 |
| 3696 | 0 | JP | G01B | G01B0011 | 4 | 13 | 2 | 1.5 | 0.22 |
| 3697 | 0 | US | H01L | H01L0033 | 29 | 44 | 11 | 1.25 | 1.10 |
| 3698 | 0 | JP | H01L | H01L0033 | 9 | 7 | 10 | 1.333333333 | 2.50 |
| 3699 | 0 | JP | B81B | B81B0003 | 13 | 7 | 2 | 2 | 1.00 |
| 3700 | 0 | US | H05B | H05B0033 | 28 | 10 | 159 | 1 | 31.80 |
| 3701 | 0 | CA | H01L | H01L0021 | 9 | 10 | 9 | 1 | 1.80 |
| 3702 | 0 | CA | H01L | H01L0029 | 15 | 14 | 8 | 1 | 2.00 |
| 3703 | 0 | JP | H01L | H01L0033 | 6 | 20 | 7 | 1.5 | 2.33 |
| 3704 | 0 | US | C23F | C23F0004 | 17 | 14 | 14 | 1 | 3.50 |
| 3705 | 0 | JP | H01L | H01L0033 | 4 | 7 | 0 | 1.5 | 0.00 |
| 3706 | 0 | JP | H01L | H01L0025 | 6 | 9 | 3 | 1.333333333 | 0.75 |
| 3707 | 0 | JP | G02F | G02F0001 | 30 | 14 | 23 | 1.2 | 3.83 |
| 3708 | 0 | JP | H01L | H01L0029 | 4 | 12 | 7 | 2.666666667 | 0.88 |
| 3709 | 0 | JP | H01L | H01L0031 | 26 | 13 | 9 | 1 | 4.50 |
| 3710 | 0 | US | C08K | C08K0009 | 9 | 27 | 10 | 1.2 | 0.83 |
| 3711 | 0 | US | H01L | H01L0021 | 20 | 1 | 61 | 1 | 30.50 |
| 3712 | 0 | US | B29C | B29C0045 | 25 | 13 | 17 | 1.5 | 1.89 |
| 3713 | 0 | JP | H01L | H01L0021 | 3 | 22 | 0 | 1.5 | 0.00 |
| 3714 | 0 | JP | H01L | H01L0033 | 5 | 34 | 12 | 1.5 | 4.00 |
| 3715 | 0 | JP | H01L | H01L0033 | 4 | 12 | 6 | 1.5 | 2.00 |
| 3716 | 0 | JP | H01L | H01L0033 | 10 | 17 | 7 | 1 | 2.33 |
| 3717 | 0 | US | C30B | C30B0025 | 13 | 27 | 66 | 2 | 33.00 |
| 3718 | 0 | JP | H01S | H01S0005 | 63 | 14 | 33 | 2.5 | 6.60 |
| 3719 | 0 | US | H01S | H01S0005 | 5 | 14 | 28 | 1.333333333 | 7.00 |
| 3720 | 0 | JP | H01L | H01L0021 | 4 | 6 | 4 | 4 | 0.50 |
| 3721 | 0 | DE | H01L | H01L0021 | 30 | 20 | 11 | 1 | 3.67 |
| 3722 | 0 | CA | C30B | C30B0023 | 12 | 22 | 6 | 1 | 2.00 |
| 3723 | 0 | TW | H01L | H01L0021 | 54 | 12 | 0 | 1.5 | 0.00 |
| 3724 | 0 | JP | C23C | C23C0016 | 8 | 7 | 0 | 1.333333333 | 0.00 |
| 3725 | 0 | KR | H01L | H01L0031 | 7 | 11 | 10 | 1 | 10.00 |
| 3726 | 0 | JP | H01L | H01L0021 | 2 | 23 | 5 | 1 | 2.50 |
| 3727 | 0 | JP | H01L | H01L0033 | 8 | 6 | 6 | 1 | 3.00 |
| 3728 | 1 | CA | H01L | H01L0021 | 24 | 10 | 65 | 1 | 16.25 |
| 3729 | 0 | NL | F21S | F21S0010 | 24 | 19 | 38 | 1.2 | 6.33 |
| 3730 | 0 | NL | F21V | F21V0005 | 31 | 15 | 122 | 1 | 20.33 |
| 3731 | 0 | US | G02B | G02B0003 | 32 | 3 | 13 | 2 | 6.50 |
| 3732 | 0 | JP | B32B | B32B0015 | 20 | 25 | 6 | 3 | 0.50 |
| 3733 | 0 | JP | G01R | G01R0001 | 7 | 13 | 3 | 1.666666667 | 0.60 |
| 3734 | 0 | JP | H01L | H01L0033 | 23 | 10 | 7 | 1 | 1.75 |
| 3735 | 0 | US | C09K | C09K0011 | 10 | 7 | 15 | 1 | 5.00 |
| 3736 | 0 | JP | B60Q | B60Q0003 | 19 | 8 | 24 | 1.666666667 | 4.80 |
| 3737 | 0 | US | H01S | H01S0005 | 12 | 14 | 26 | 1.75 | 3.71 |
| 3738 | 0 | US | C30B | C30B0023 | 13 | 5 | 3 | 1.2 | 0.50 |
| 3739 | 0 | GB | H01L | H01L0021 | 25 | 7 | 0 | 1 | 0.00 |
| 3740 | 0 | TW | H01L | H01L0033 | 17 | 12 | 4 | 1 | 1.00 |
| 3741 | 0 | JP | H01L | H01L0021 | 15 | 6 | 7 | 1.5 | 2.33 |
| 3742 | 0 | US | H01L | H01L0033 | 22 | 1 | 60 | 1.25 | 12.00 |
| 3743 | 0 | TW | H01L | H01L0021 | 15 | 2 | 23 | 2 | 11.50 |
| 3744 | 0 | US | B32B | B32B0005 | 12 | 32 | 11 | 1.8 | 1.22 |
| 3745 | 0 | US | H01L | H01L0021 | 11 | 8 | 16 | 1.25 | 3.20 |
| 3746 | 0 | JP | H01L | H01L0025 | 20 | 7 | 3 | 2.2 | 0.27 |
| 3747 | 0 | US | C30B | C30B0025 | 24 | 9 | 69 | 2 | 34.50 |
| 3748 | 0 | US | H01L | H01L0033 | 6 | 8 | 60 | 1 | 12.00 |
| 3749 | 0 | JP | H05B | H05B0033 | 26 | 10 | 51 | 4.5 | 1.89 |
| 3750 | 0 | JP | B41J | B41J0002 | 8 | 12 | 6 | 1 | 3.00 |
| 3751 | 0 | US | C23F | C23F0001 | 6 | 10 | 2 | 1 | 0.67 |
| 3752 | 0 | TW | H01L | H01L0033 | 4 | 1 | 15 | 1 | 7.50 |
| 3753 | 0 | JP | C30B | C30B0029 | 37 | 6 | 1 | 1.5 | 0.33 |
| 3754 | 0 | US | H01L | H01L0033 | 16 | 16 | 8 | 1 | 4.00 |
| 3755 | 0 | JP | H01S | H01S0005 | 9 | 16 | 1 | 1.5 | 0.33 |
| 3756 | 0 | JP | C30B | C30B0025 | 17 | 17 | 22 | 1.333333333 | 5.50 |
| 3757 | 0 | JP | H01L | H01L0029 | 6 | 6 | 12 | 1 | 3.00 |
| 3758 | 0 | JP | H01L | H01L0027 | 3 | 21 | 3 | 1.75 | 0.43 |
| 3759 | 0 | TW | H01L | H01L0025 | 23 | 7 | 23 | 1 | 11.50 |
| 3760 | 0 | GB | H01L | H01L0027 | 53 | 10 | 10 | 1.2 | 1.67 |
| 3761 | 0 | US | C30B | C30B0025 | 29 | 19 | 37 | 1 | 18.50 |
| 3762 | 0 | US | H01L | H01L0033 | 26 | 20 | 13 | 2 | 6.50 |
| 3763 | 0 | JP | H01L | H01L0033 | 24 | 50 | 9 | 1.333333333 | 2.25 |
| 3764 | 0 | JP | H01L | H01L0021 | 19 | 11 | 15 | 1 | 7.50 |
| 3765 | 0 | DE | B24B | B24B0037 | 13 | 24 | 4 | 1 | 1.33 |
| 3766 | 0 | SG | B22F | B22F0003 | 43 | 11 | 1 | 1.25 | 0.20 |
| 3767 | 0 | JP | H01L | H01L0033 | 5 | 23 | 5 | 2 | 2.50 |
| 3768 | 0 | CA | H01L | H01L0033 | 47 | 32 | 31 | 2.2 | 2.82 |
| 3769 | 0 | JP | H05B | H05B0041 | 9 | 3 | 1 | 1 | 0.50 |
| 3770 | 0 | US | H01L | H01L0029 | 27 | 22 | 56 | 1 | 11.20 |
| 3771 | 0 | CA | H01L | H01L0025 | 19 | 57 | 75 | 1.428571429 | 7.50 |
| 3772 | 0 | DE | H01L | H01L0033 | 27 | 8 | 21 | 3.333333333 | 1.05 |
| 3773 | 0 | JP | G03F | G03F0001 | 4 | 7 | 2 | 1.25 | 0.40 |
| 3774 | 0 | CA | H01L | H01L0021 | 33 | 76 | 9 | 1.25 | 1.80 |
| 3775 | 0 | US | H01L | H01L0033 | 10 | 4 | 23 | 2 | 11.50 |
| 3776 | 0 | US | H01L | H01L0021 | 23 | 6 | 44 | 1 | 11.00 |
| 3777 | 0 | JP | H01L | H01L0033 | 4 | 47 | 11 | 5 | 0.55 |
| 3778 | 0 | JP | H01S | H01S0005 | 17 | 10 | 14 | 1 | 7.00 |
| 3779 | 0 | TW | H01L | H01L0033 | 22 | 3 | 8 | 1 | 4.00 |
| 3780 | 0 | JP | B41J | B41J0002 | 27 | 5 | 15 | 1 | 7.50 |
| 3781 | 0 | TW | H01L | H01L0033 | 15 | 10 | 3 | 1.2 | 0.50 |
| 3782 | 0 | US | H01L | H01L0023 | 15 | 14 | 9 | 1 | 3.00 |
| 3783 | 0 | JP | F21K | F21K0099 | 24 | 1 | 43 | 1.5 | 14.33 |
| 3784 | 0 | JP | G03F | G03F0007 | 26 | 17 | 0 | 1 | 0.00 |
| 3785 | 0 | US | H01L | H01L0027 | 26 | 20 | 105 | 1.333333333 | 13.13 |
| 3786 | 0 | JP | H01L | H01L0021 | 14 | 7 | 9 | 1.5 | 3.00 |
| 3787 | 0 | US | G02B | G02B0006 | 28 | 21 | 144 | 1 | 72.00 |
| 3788 | 0 | JP | G11B | G11B0007 | 3 | 3 | 8 | 1.5 | 2.67 |
| 3789 | 0 | TW | H01L | H01L0033 | 15 | 6 | 3 | 1.5 | 1.00 |
| 3790 | 0 | US | H01L | H01L0021 | 35 | 25 | 61 | 3 | 20.33 |
| 3791 | 0 | JP | H01S | H01S0005 | 7 | 11 | 0 | 2 | 0.00 |
| 3792 | 0 | JP | H01L | H01L0033 | 49 | 8 | 4 | 4.5 | 0.44 |
| 3793 | 0 | JP | H01L | H01L0031 | 10 | 6 | 1 | 1 | 0.33 |
| 3794 | 0 | JP | H01L | H01L0033 | 18 | 11 | 3 | 1 | 1.50 |
| 3795 | 0 | KR | H01L | H01L0033 | 4 | 3 | 3 | 1 | 1.50 |
| 3796 | 1 | CA | H01L | H01L0033 | 17 | 20 | 35 | 1.2 | 5.83 |
| 3797 | 0 | US | H01L | H01L0033 | 20 | 6 | 283 | 1.25 | 56.60 |
| 3798 | 0 | TW | C09K | C09K0011 | 10 | 7 | 4 | 1 | 2.00 |
| 3799 | 0 | JP | C30B | C30B0025 | 11 | 15 | 9 | 1.333333333 | 2.25 |
| 3800 | 0 | CA | H01L | H01L0033 | 11 | 16 | 36 | 1.2 | 6.00 |
| 3801 | 0 | JP | H01L | H01L0033 | 5 | 20 | 2 | 1 | 0.67 |
| 3802 | 0 | JP | H01L | H01L0033 | 41 | 88 | 0 | 3.4 | 0.00 |
| 3803 | 0 | US | C09K | C09K0011 | 14 | 28 | 9 | 2.25 | 1.00 |
| 3804 | 0 | JP | H01L | H01L0033 | 13 | 8 | 0 | 1.5 | 0.00 |
| 3805 | 0 | JP | H01L | H01L0021 | 5 | 50 | 14 | 2.2 | 1.27 |
| 3806 | 0 | JP | H01L | H01L0023 | 21 | 8 | 17 | 1.5 | 1.89 |
| 3807 | 0 | US | C30B | C30B0023 | 6 | 3 | 15 | 2 | 7.50 |
| 3808 | 0 | JP | H01J | H01J0009 | 5 | 13 | 3 | 1 | 0.60 |
| 3809 | 0 | JP | H01S | H01S0005 | 36 | 22 | 32 | 1.25 | 6.40 |
| 3810 | 0 | CA | F21V | F21V0005 | 63 | 27 | 101 | 1 | 25.25 |
| 3811 | 0 | US | G02B | G02B0006 | 23 | 12 | 73 | 1.333333333 | 18.25 |
| 3812 | 0 | CA | C30B | C30B0023 | 16 | 9 | 1 | 1 | 1.00 |
| 3813 | 0 | US | G02B | G02B0006 | 11 | 32 | 9 | 1 | 2.25 |
| 3814 | 0 | DE | C03C | C03C0017 | 6 | 6 | 0 | 1 | 0.00 |
| 3815 | 0 | DE | H01L | H01L0021 | 17 | 23 | 0 | 1.25 | 0.00 |
| 3816 | 0 | US | H01L | H01L0029 | 32 | 120 | 19 | 2.714285714 | 1.00 |
| 3817 | 0 | US | H01L | H01L0023 | 10 | 7 | 14 | 1 | 7.00 |
| 3818 | 0 | JP | G02B | G02B0006 | 29 | 14 | 37 | 1.5 | 4.11 |
| 3819 | 0 | KR | H01L | H01L0033 | 12 | 4 | 11 | 1 | 2.75 |
| 3820 | 0 | CA | G02B | G02B0027 | 24 | 16 | 5 | 1 | 5.00 |
| 3821 | 0 | JP | G01N | G01N0021 | 3 | 5 | 4 | 1 | 2.00 |
| 3822 | 0 | CA | H01L | H01L0021 | 8 | 10 | 3 | 1 | 1.50 |
| 3823 | 0 | JP | H01L | H01L0021 | 15 | 15 | 0 | 1 | 0.00 |
| 3824 | 0 | CA | F21V | F21V0005 | 54 | 10 | 70 | 1 | 14.00 |
| 3825 | 0 | JP | H01L | H01L0033 | 100 | 105 | 3 | 3.4 | 0.18 |
| 3826 | 0 | DE | H01L | H01L0021 | 6 | 6 | 20 | 1 | 5.00 |
| 3827 | 0 | US | F21V | F21V0008 | 17 | 4 | 4 | 2 | 2.00 |
| 3828 | 0 | US | H01L | H01L0021 | 44 | 35 | 330 | 3.333333333 | 33.00 |
| 3829 | 0 | TW | H01L | H01L0033 | 28 | 15 | 9 | 1 | 9.00 |
| 3830 | 0 | JP | F21V | F21V0008 | 18 | 48 | 62 | 5 | 0.69 |
| 3831 | 0 | JP | B29C | B29C0045 | 6 | 9 | 17 | 1 | 3.40 |
| 3832 | 0 | US | B23K | B23K0003 | 18 | 9 | 26 | 2 | 13.00 |
| 3833 | 0 | US | H01L | H01L0033 | 6 | 3 | 44 | 1.25 | 8.80 |
| 3834 | 0 | JP | H01L | H01L0021 | 9 | 10 | 13 | 2 | 2.17 |
| 3835 | 0 | JP | H01L | H01L0033 | 4 | 5 | 1 | 1.5 | 0.33 |
| 3836 | 0 | JP | H01L | H01L0021 | 8 | 82 | 1 | 1 | 0.17 |
| 3837 | 0 | US | H01S | H01S0005 | 4 | 4 | 8 | 1.666666667 | 1.60 |
| 3838 | 0 | US | H01S | H01S0005 | 26 | 9 | 19 | 2 | 9.50 |
| 3839 | 0 | JP | H01L | H01L0033 | 6 | 14 | 11 | 1.5 | 3.67 |
| 3840 | 0 | JP | H01L | H01L0033 | 6 | 7 | 2 | 1 | 0.67 |
| 3841 | 0 | US | H01L | H01L0033 | 8 | 4 | 38 | 1 | 9.50 |
| 3842 | 0 | TW | H01L | H01L0033 | 23 | 8 | 17 | 1 | 5.67 |
| 3843 | 0 | JP | H01L | H01L0033 | 5 | 17 | 19 | 3 | 3.17 |
| 3844 | 0 | TW | H01L | H01L0033 | 8 | 18 | 31 | 1.25 | 6.20 |
| 3845 | 0 | US | H01L | H01L0033 | 5 | 10 | 13 | 1.166666667 | 1.86 |
| 3846 | 0 | US | H01L | H01L0023 | 11 | 23 | 10 | 4 | 2.50 |
| 3847 | 0 | TW | H01L | H01L0033 | 23 | 3 | 31 | 1 | 10.33 |
| 3848 | 0 | JP | F21V | F21V0008 | 19 | 75 | 55 | 5 | 0.61 |
| 3849 | 0 | JP | H01L | H01L0033 | 3 | 21 | 3 | 1.5 | 1.00 |
| 3850 | 0 | JP | C30B | C30B0023 | 14 | 21 | 4 | 1 | 2.00 |
| 3851 | 0 | JP | H01L | H01L0033 | 20 | 6 | 4 | 1 | 1.33 |
| 3852 | 0 | JP | H01L | H01L0033 | 6 | 15 | 6 | 2 | 1.50 |
| 3853 | 0 | US | H01L | H01L0023 | 46 | 39 | 50 | 1.4 | 7.14 |
| 3854 | 0 | JP | H01L | H01L0021 | 12 | 15 | 9 | 4 | 1.13 |
| 3855 | 0 | US | C23C | C23C0016 | 50 | 11 | 12 | 1 | 6.00 |
| 3856 | 0 | US | C09K | C09K0011 | 7 | 10 | 9 | 2.6 | 0.69 |
| 3857 | 0 | JP | H01L | H01L0033 | 19 | 9 | 21 | 1 | 10.50 |
| 3858 | 0 | US | H01L | H01L0021 | 13 | 16 | 3 | 1.125 | 0.33 |
| 3859 | 0 | JP | B41J | B41J0002 | 15 | 2 | 6 | 1 | 3.00 |
| 3860 | 0 | JP | H01L | H01L0033 | 5 | 16 | 4 | 1.666666667 | 0.80 |
| 3861 | 0 | US | H01L | H01L0023 | 43 | 28 | 79 | 2 | 39.50 |
| 3862 | 0 | TW | H01J | H01J0001 | 10 | 7 | 3 | 1 | 1.50 |
| 3863 | 0 | JP | C30B | C30B0029 | 12 | 11 | 12 | 1 | 6.00 |
| 3864 | 0 | JP | H01L | H01L0031 | 74 | 12 | 8 | 2 | 2.00 |
| 3865 | 0 | DE | H01L | H01L0031 | 7 | 30 | 61 | 2 | 5.08 |
| 3866 | 1 | JP | G02B | G02B0006 | 14 | 13 | 5 | 1 | 2.50 |
| 3867 | 0 | JP | C30B | C30B0025 | 8 | 28 | 4 | 1.625 | 0.31 |
| 3868 | 0 | JP | C30B | C30B0029 | 24 | 26 | 35 | 1 | 17.50 |
| 3869 | 0 | JP | C30B | C30B0025 | 8 | 27 | 4 | 1.625 | 0.31 |
| 3870 | 0 | JP | H01L | H01L0027 | 8 | 19 | 4 | 1.5 | 1.33 |
| 3871 | 0 | US | H01L | H01L0021 | 14 | 25 | 4 | 1.857142857 | 0.31 |
| 3872 | 0 | CA | C30B | C30B0025 | 28 | 26 | 18 | 1 | 4.50 |
| 3873 | 0 | US | C23C | C23C0014 | 16 | 50 | 41 | 4.333333333 | 3.15 |
| 3874 | 0 | FR | C30B | C30B0025 | 22 | 9 | 3 | 1 | 3.00 |
| 3875 | 0 | US | G03F | G03F0007 | 15 | 18 | 19 | 1 | 1.73 |
| 3876 | 0 | CH | H01L | H01L0025 | 54 | 14 | 2 | 4.333333333 | 0.15 |
| 3877 | 0 | JP | G02B | G02B0019 | 14 | 4 | 4 | 1 | 2.00 |
| 3878 | 0 | US | F21K | F21K0099 | 17 | 14 | 28 | 1 | 28.00 |
| 3879 | 0 | US | F21K | F21K0099 | 8 | 14 | 10 | 1 | 10.00 |
| 3880 | 0 | US | C23C | C23C0014 | 20 | 47 | 26 | 4.333333333 | 2.00 |
| 3881 | 0 | US | H01L | H01L0027 | 20 | 47 | 16 | 1 | 1.78 |
| 3882 | 0 | TW | H01L | H01L0025 | 10 | 3 | 17 | 1 | 8.50 |
| 3883 | 0 | JP | H01L | H01L0033 | 5 | 5 | 10 | 1 | 5.00 |
| 3884 | 0 | CA | H01L | H01L0033 | 26 | 21 | 15 | 1 | 3.75 |
| 3885 | 0 | US | H05K | H05K0001 | 20 | 19 | 3 | 1 | 1.50 |
| 3886 | 0 | US | H01L | H01L0023 | 9 | 12 | 4 | 1.5 | 1.33 |
| 3887 | 0 | JP | H01L | H01L0021 | 28 | 8 | 2 | 1 | 1.00 |
| 3888 | 0 | JP | H01L | H01L0031 | 7 | 6 | 0 | 1.5 | 0.00 |
| 3889 | 0 | JP | H01L | H01L0033 | 16 | 6 | 10 | 1.4 | 1.43 |
| 3890 | 0 | US | H01L | H01L0021 | 3 | 24 | 76 | 3.333333333 | 7.60 |
| 3891 | 0 | US | G01R | G01R0001 | 14 | 37 | 102 | 12.71428571 | 1.15 |
| 3892 | 0 | JP | H01L | H01L0033 | 7 | 6 | 28 | 1 | 9.33 |
| 3893 | 0 | JP | H01L | H01L0021 | 12 | 69 | 8 | 2.2 | 0.73 |
| 3894 | 0 | TW | H01L | H01L0021 | 36 | 8 | 20 | 4 | 1.67 |
| 3895 | 0 | JP | C30B | C30B0029 | 64 | 5 | 8 | 1.25 | 1.60 |
| 3896 | 0 | US | B29C | B29C0035 | 28 | 30 | 27 | 2 | 13.50 |
| 3897 | 0 | US | H01B | H01B0005 | 13 | 4 | 2 | 1.666666667 | 0.40 |
| 3898 | 0 | US | H01L | H01L0033 | 38 | 24 | 25 | 4 | 6.25 |
| 3899 | 0 | CA | C09K | C09K0011 | 28 | 14 | 67 | 1 | 16.75 |
| 3900 | 0 | JP | C23C | C23C0016 | 4 | 6 | 5 | 1 | 1.67 |
| 3901 | 0 | JP | H01L | H01L0033 | 20 | 15 | 5 | 2.2 | 0.45 |
| 3902 | 0 | SG | H01L | H01L0021 | 10 | 17 | 8 | 2 | 4.00 |
| 3903 | 0 | JP | G02B | G02B0006 | 17 | 6 | 3 | 1 | 1.50 |
| 3904 | 0 | US | G03F | G03F0007 | 19 | 30 | 7 | 2.5 | 0.70 |
| 3905 | 0 | JP | C30B | C30B0029 | 10 | 16 | 0 | 1 | 0.00 |
| 3906 | 0 | JP | C30B | C30B0029 | 5 | 10 | 1 | 1 | 0.50 |
| 3907 | 0 | CA | H01L | H01L0033 | 9 | 27 | 30 | 1.2 | 5.00 |
| 3908 | 0 | DE | H01L | H01L0033 | 20 | 13 | 13 | 1.142857143 | 1.63 |
| 3909 | 0 | JP | H01L | H01L0021 | 11 | 11 | 11 | 1.5 | 3.67 |
| 3910 | 0 | JP | H01L | H01L0033 | 26 | 21 | 14 | 1 | 7.00 |
| 3911 | 0 | US | B41M | B41M0005 | 23 | 41 | 6 | 1 | 2.00 |
| 3912 | 0 | JP | H01L | H01L0023 | 12 | 57 | 5 | 1.5 | 1.67 |
| 3913 | 0 | US | H01S | H01S0005 | 9 | 6 | 3 | 1 | 0.75 |
| 3914 | 0 | TW | G09F | G09F0009 | 15 | 2 | 11 | 1 | 11.00 |
| 3915 | 0 | KR | B23K | B23K0026 | 17 | 5 | 4 | 1 | 1.33 |
| 3916 | 0 | DE | H01L | H01L0021 | 10 | 33 | 1 | 1.571428571 | 0.09 |
| 3917 | 0 | US | G01B | G01B0011 | 35 | 40 | 6 | 4.833333333 | 0.21 |
| 3918 | 0 | JP | H01L | H01L0023 | 4 | 6 | 19 | 1.5 | 6.33 |
| 3919 | 0 | JP | G03F | G03F0007 | 37 | 21 | 3 | 1 | 1.50 |
| 3920 | 0 | US | C30B | C30B0023 | 12 | 126 | 5 | 3.333333333 | 0.25 |
| 3921 | 0 | US | B29C | B29C0045 | 5 | 30 | 11 | 1.5 | 1.22 |
| 3922 | 0 | US | B29C | B29C0045 | 16 | 34 | 14 | 1.5 | 1.56 |
| 3923 | 0 | JP | H01L | H01L0033 | 47 | 3 | 15 | 3.5 | 2.14 |
| 3924 | 0 | KR | H01L | H01L0033 | 29 | 18 | 10 | 1.666666667 | 1.00 |
| 3925 | 0 | JP | H01L | H01L0033 | 6 | 12 | 2 | 1 | 1.00 |
| 3926 | 0 | US | C30B | C30B0023 | 24 | 6 | 14 | 2 | 7.00 |
| 3927 | 0 | DE | H05B | H05B0033 | 65 | 5 | 7 | 1 | 2.33 |
| 3928 | 0 | US | C25B | C25B0011 | 54 | 15 | 12 | 1.833333333 | 0.55 |
| 3929 | 0 | MI | B32B | B32B0003 | 11 | 3 | 15 | 1 | 15.00 |
| 3930 | 0 | US | C09K | C09K0011 | 21 | 19 | 10 | 1.333333333 | 1.25 |
| 3931 | 0 | US | C30B | C30B0025 | 30 | 8 | 95 | 2 | 11.88 |
| 3932 | 0 | JP | G02B | G02B0006 | 14 | 10 | 3 | 1.5 | 1.00 |
| 3933 | 0 | TW | H01L | H01L0025 | 7 | 3 | 4 | 1 | 2.00 |
| 3934 | 0 | US | H01S | H01S0005 | 8 | 7 | 3 | 1 | 0.50 |
| 3935 | 0 | JP | G02B | G02B0006 | 12 | 7 | 17 | 1 | 8.50 |
| 3936 | 0 | US | H01L | H01L0033 | 12 | 8 | 5 | 2 | 2.50 |
| 3937 | 0 | JP | H01L | H01L0033 | 4 | 2 | 0 | 1 | 0.00 |
| 3938 | 0 | US | C30B | C30B0025 | 29 | 13 | 71 | 2 | 17.75 |
| 3939 | 0 | JP | C30B | C30B0033 | 5 | 3 | 7 | 1.25 | 1.40 |
| 3940 | 0 | TW | H01L | H01L0025 | 24 | 1 | 1 | 1 | 0.50 |
| 3941 | 0 | DE | C09K | C09K0011 | 33 | 15 | 58 | 3 | 1.14 |
| 3942 | 0 | JP | H01L | H01L0021 | 2 | 6 | 2 | 2 | 0.50 |
| 3943 | 0 | US | H01L | H01L0021 | 26 | 22 | 62 | 1.2 | 10.33 |
| 3944 | 0 | US | H01L | H01L0033 | 24 | 19 | 16 | 1 | 8.00 |
| 3945 | 0 | JP | G11B | G11B0007 | 15 | 17 | 12 | 1.5 | 4.00 |
| 3946 | 0 | KR | H01L | H01L0033 | 20 | 5 | 26 | 1.333333333 | 6.50 |
| 3947 | 0 | JP | C30B | C30B0029 | 22 | 10 | 9 | 1 | 2.25 |
| 3948 | 0 | US | H01L | H01L0021 | 9 | 46 | 10 | 3.333333333 | 1.00 |
| 3949 | 0 | JP | C30B | C30B0029 | 3 | 6 | 13 | 1.166666667 | 1.86 |
| 3950 | 0 | US | H01J | H01J0037 | 15 | 29 | 9 | 2 | 4.50 |
| 3951 | 0 | JP | H01L | H01L0033 | 18 | 8 | 2 | 2 | 0.33 |
| 3952 | 0 | JP | H01L | H01L0033 | 9 | 4 | 1 | 1 | 0.50 |
| 3953 | 0 | JP | G09G | G09G0003 | 41 | 26 | 17 | 1.4 | 2.43 |
| 3954 | 0 | JP | H01L | H01L0033 | 6 | 8 | 7 | 1.5 | 2.33 |
| 3955 | 0 | JP | C08K | C08K0003 | 20 | 5 | 14 | 1 | 2.80 |
| 3956 | 0 | JP | H01L | H01L0033 | 1 | 17 | 0 | 5.5 | 0.00 |
| 3957 | 0 | CA | H01L | H01L0033 | 25 | 22 | 34 | 1.5 | 3.78 |
| 3958 | 0 | US | H01L | H01L0023 | 5 | 39 | 25 | 1.4 | 3.57 |
| 3959 | 0 | JP | H01L | H01L0021 | 16 | 15 | 5 | 1.5 | 1.67 |
| 3960 | 0 | TW | H01L | H01L0033 | 34 | 22 | 7 | 1 | 1.75 |
| 3961 | 0 | JP | H01L | H01L0021 | 31 | 21 | 2 | 1.375 | 0.18 |
| 3962 | 0 | KR | H01L | H01L0029 | 1 | 7 | 1 | 1 | 0.50 |
| 3963 | 0 | CA | H01L | H01L0033 | 26 | 30 | 6 | 1 | 1.50 |
| 3964 | 0 | JP | H01L | H01L0021 | 10 | 7 | 1 | 2 | 0.17 |
| 3965 | 0 | US | C09K | C09K0011 | 13 | 64 | 200 | 1.333333333 | 25.00 |
| 3966 | 0 | TW | H01L | H01L0021 | 27 | 4 | 1 | 1 | 1.00 |
| 3967 | 0 | JP | C25D | C25D0007 | 3 | 11 | 0 | 1 | 0.00 |
| 3968 | 0 | TW | H01L | H01L0033 | 18 | 3 | 5 | 1 | 2.50 |
| 3969 | 0 | JP | H01L | H01L0021 | 4 | 10 | 1 | 1.4 | 0.14 |
| 3970 | 0 | CA | C09K | C09K0011 | 32 | 52 | 53 | 1 | 10.60 |
| 3971 | 0 | US | C09K | C09K0011 | 12 | 4 | 100 | 2 | 50.00 |
| 3972 | 0 | US | H01L | H01L0023 | 39 | 20 | 6 | 6 | 1.00 |
| 3973 | 0 | JP | H01L | H01L0029 | 4 | 22 | 6 | 1.111111111 | 0.60 |
| 3974 | 0 | US | H01L | H01L0021 | 36 | 128 | 123 | 1.333333333 | 30.75 |
| 3975 | 0 | DE | H05B | H05B0033 | 17 | 18 | 8 | 1 | 1.60 |
| 3976 | 0 | TW | H01L | H01L0033 | 18 | 1 | 7 | 1 | 7.00 |
| 3977 | 0 | JP | H01L | H01L0033 | 43 | 22 | 4 | 1.2 | 0.67 |
| 3978 | 0 | JP | H01L | H01L0021 | 19 | 14 | 1 | 1 | 0.50 |
| 3979 | 0 | JP | H01L | H01L0021 | 50 | 1 | 7 | 3 | 1.17 |
| 3980 | 0 | JP | H01L | H01L0033 | 12 | 8 | 0 | 1.8 | 0.00 |
| 3981 | 0 | KR | H01L | H01L0021 | 7 | 4 | 0 | 1 | 0.00 |
| 3982 | 0 | US | G01R | G01R0001 | 24 | 26 | 65 | 2 | 5.42 |
| 3983 | 0 | US | B81C | B81C0001 | 8 | 8 | 18 | 1 | 6.00 |
| 3984 | 0 | SI | C30B | C30B0023 | 28 | 27 | 2 | 1 | 1.00 |
| 3985 | 0 | JP | H01L | H01L0025 | 20 | 28 | 17 | 1.4 | 2.43 |
| 3986 | 0 | JP | H01L | H01L0023 | 37 | 7 | 13 | 1 | 3.25 |
| 3987 | 0 | JP | H01L | H01L0023 | 7 | 6 | 10 | 2 | 1.67 |
| 3988 | 0 | US | C09K | C09K0011 | 9 | 138 | 26 | 2.761904762 | 0.45 |
| 3989 | 0 | US | C30B | C30B0029 | 80 | 90 | 70 | 1.428571429 | 7.00 |
| 3990 | 0 | US | C23F | C23F0004 | 140 | 56 | 12 | 3.4 | 0.71 |
| 3991 | 0 | JP | H01L | H01L0033 | 6 | 7 | 2 | 1 | 0.50 |
| 3992 | 0 | JP | H01L | H01L0021 | 19 | 6 | 0 | 1 | 0.00 |
| 3993 | 0 | JP | C30B | C30B0029 | 2 | 22 | 5 | 1.25 | 1.00 |
| 3994 | 0 | US | F21K | F21K0099 | 3 | 22 | 4 | 1.666666667 | 0.80 |
| 3995 | 0 | JP | H01S | H01S0005 | 13 | 9 | 6 | 1.5 | 1.00 |
| 3996 | 0 | FR | C30B | C30B0011 | 21 | 18 | 1 | 1.875 | 0.07 |
| 3997 | 0 | TW | H01L | H01L0033 | 14 | 19 | 9 | 1 | 2.25 |
| 3998 | 0 | JP | H01L | H01L0033 | 4 | 14 | 0 | 1.5 | 0.00 |
| 3999 | 0 | US | H05B | H05B0037 | 25 | 303 | 20 | 23.28571429 | 0.06 |
| 4000 | 0 | JP | C30B | C30B0015 | 10 | 4 | 3 | 1 | 1.50 |
| 4001 | 0 | JP | H01L | H01L0033 | 9 | 4 | 5 | 1 | 1.00 |
| 4002 | 0 | DE | F21V | F21V0005 | 13 | 7 | 5 | 1 | 0.83 |
| 4003 | 0 | JP | G02B | G02B0006 | 7 | 9 | 0 | 1 | 0.00 |
| 4004 | 0 | TW | H01L | H01L0033 | 13 | 2 | 4 | 1.5 | 1.33 |
| 4005 | 0 | US | C23C | C23C0016 | 34 | 14 | 10 | 1 | 3.33 |
| 4006 | 0 | US | H01S | H01S0005 | 7 | 11 | 4 | 5 | 0.80 |
| 4007 | 0 | JP | H01L | H01L0021 | 7 | 11 | 0 | 2 | 0.00 |
| 4008 | 0 | US | H01L | H01L0021 | 16 | 14 | 4 | 3 | 1.33 |
| 4009 | 0 | TW | H01L | H01L0033 | 9 | 3 | 10 | 2 | 5.00 |
| 4010 | 0 | JP | C30B | C30B0023 | 8 | 7 | 0 | 1 | 0.00 |
| 4011 | 0 | DE | H01L | H01L0033 | 11 | 26 | 1 | 1 | 0.25 |
| 4012 | 0 | DE | H01L | H01L0031 | 5 | 25 | 4 | 3 | 0.67 |
| 4013 | 0 | JP | C30B | C30B0023 | 3 | 7 | 3 | 1 | 0.75 |
| 4014 | 0 | US | H01L | H01L0033 | 25 | 45 | 5 | 1.25 | 0.50 |
| 4015 | 0 | JP | H01L | H01L0021 | 5 | 7 | 6 | 3.5 | 0.86 |
| 4016 | 1 | CA | C09K | C09K0011 | 23 | 1 | 63 | 1 | 21.00 |
| 4017 | 0 | JP | C09K | C09K0011 | 37 | 6 | 26 | 1 | 8.67 |
| 4018 | 0 | TW | H01L | H01L0031 | 29 | 7 | 1 | 1 | 0.50 |
| 4019 | 0 | US | F21K | F21K0099 | 7 | 21 | 11 | 1 | 11.00 |
| 4020 | 0 | JP | C01C | C01C0001 | 3 | 11 | 1 | 1.333333333 | 0.25 |
| 4021 | 0 | TW | G03F | G03F0007 | 23 | 11 | 9 | 1 | 9.00 |
| 4022 | 0 | JP | H01L | H01L0021 | 26 | 13 | 7 | 1 | 3.50 |
| 4023 | 0 | TW | H01L | H01L0033 | 14 | 11 | 6 | 1.333333333 | 1.50 |
| 4024 | 0 | TW | H01S | H01S0005 | 6 | 10 | 0 | 1 | 0.00 |
| 4025 | 0 | JP | G02B | G02B0005 | 7 | 7 | 7 | 2 | 1.17 |
| 4026 | 0 | US | C09K | C09K0011 | 14 | 21 | 13 | 1.333333333 | 1.63 |
| 4027 | 0 | JP | H01L | H01L0021 | 22 | 6 | 11 | 1 | 5.50 |
| 4028 | 0 | US | H01L | H01L0021 | 83 | 48 | 15 | 1.4 | 2.14 |
| 4029 | 0 | JP | G03F | G03F0001 | 15 | 19 | 0 | 1 | 0.00 |
| 4030 | 0 | US | H01L | H01L0051 | 27 | 24 | 16 | 2 | 8.00 |
| 4031 | 0 | US | H01L | H01L0023 | 71 | 18 | 10 | 6 | 1.67 |
| 4032 | 0 | JP | H01L | H01L0033 | 11 | 31 | 14 | 2 | 7.00 |
| 4033 | 0 | US | C30B | C30B0029 | 36 | 31 | 3 | 2 | 0.75 |
| 4034 | 0 | US | C30B | C30B0029 | 4 | 31 | 5 | 2 | 1.25 |
| 4035 | 0 | JP | H01L | H01L0021 | 13 | 2 | 1 | 1 | 0.25 |
| 4036 | 0 | JP | B41J | B41J0002 | 8 | 6 | 1 | 1 | 0.25 |
| 4037 | 0 | JP | H01L | H01L0033 | 7 | 1 | 0 | 1 | 0.00 |
| 4038 | 0 | DE | H01L | H01L0033 | 26 | 57 | 31 | 3.125 | 1.24 |
| 4039 | 0 | CA | H01L | H01L0033 | 21 | 6 | 30 | 1.333333333 | 7.50 |
| 4040 | 0 | FR | H01L | H01L0033 | 17 | 6 | 6 | 1 | 0.75 |
| 4041 | 0 | US | H01L | H01L0021 | 20 | 7 | 9 | 1 | 3.00 |
| 4042 | 0 | JP | C23C | C23C0016 | 17 | 53 | 4 | 1 | 2.00 |
| 4043 | 0 | JP | H01L | H01L0033 | 37 | 6 | 0 | 1.5 | 0.00 |
| 4044 | 0 | US | C09K | C09K0011 | 33 | 21 | 128 | 2 | 64.00 |
| 4045 | 0 | US | H01S | H01S0005 | 12 | 11 | 8 | 5 | 1.60 |
| 4046 | 0 | DE | H01L | H01L0033 | 13 | 13 | 26 | 1 | 5.20 |
| 4047 | 0 | JP | H01L | H01L0021 | 10 | 18 | 3 | 1.5 | 1.00 |
| 4048 | 0 | TW | H01L | H01L0021 | 23 | 13 | 29 | 53 | 0.14 |
| 4049 | 0 | VG | C08F | C08F0220 | 22 | 17 | 14 | 1 | 2.33 |
| 4050 | 0 | JP | H01S | H01S0005 | 25 | 4 | 7 | 1.5 | 2.33 |
| 4051 | 0 | US | B81C | B81C0001 | 23 | 31 | 62 | 1.166666667 | 8.86 |
| 4052 | 0 | JP | B23K | B23K0035 | 1 | 16 | 3 | 1.5 | 1.00 |
| 4053 | 0 | JP | H01S | H01S0005 | 25 | 11 | 0 | 2 | 0.00 |
| 4054 | 0 | JP | H01L | H01L0033 | 11 | 8 | 6 | 2.333333333 | 0.86 |
| 4055 | 0 | JP | H01L | H01L0033 | 21 | 11 | 1 | 1 | 0.50 |
| 4056 | 0 | JP | C09K | C09K0011 | 6 | 12 | 13 | 1 | 1.86 |
| 4057 | 0 | US | H01L | H01L0021 | 19 | 24 | 8 | 1 | 1.60 |
| 4058 | 0 | US | H01L | H01L0025 | 24 | 18 | 41 | 2 | 20.50 |
| 4059 | 0 | DE | H01L | H01L0033 | 17 | 15 | 28 | 2 | 2.33 |
| 4060 | 0 | US | F21K | F21K0099 | 22 | 23 | 11 | 2 | 0.79 |
| 4061 | 0 | US | G02B | G02B0026 | 7 | 23 | 68 | 4 | 17.00 |
| 4062 | 0 | TW | H01L | H01L0021 | 60 | 20 | 19 | 2 | 4.75 |
| 4063 | 0 | JP | H01S | H01S0005 | 2 | 7 | 8 | 1.5 | 2.67 |
| 4064 | 0 | JP | H01L | H01L0029 | 2 | 11 | 2 | 1.333333333 | 0.50 |
| 4065 | 0 | CA | H01L | H01L0021 | 9 | 7 | 3 | 1.5 | 1.00 |
| 4066 | 0 | US | B29C | B29C0045 | 38 | 23 | 27 | 1.4 | 3.86 |
| 4067 | 0 | US | C04B | C04B0035 | 10 | 16 | 2 | 1.25 | 0.40 |
| 4068 | 0 | TW | H05K | H05K0001 | 8 | 3 | 2 | 1 | 2.00 |
| 4069 | 0 | US | F21K | F21K0099 | 10 | 39 | 55 | 1.75 | 7.86 |
| 4070 | 0 | CA | H01L | H01L0033 | 20 | 1 | 5 | 1 | 1.25 |
| 4071 | 0 | TW | H01L | H01L0021 | 26 | 9 | 9 | 2 | 4.50 |
| 4072 | 0 | TW | H01L | H01L0023 | 9 | 3 | 15 | 1 | 15.00 |
| 4073 | 0 | JP | H01L | H01L0021 | 8 | 16 | 1 | 1.333333333 | 0.13 |
| 4074 | 0 | US | C01B | C01B0031 | 12 | 9 | 8 | 2.5 | 1.60 |
| 4075 | 0 | US | H01L | H01L0021 | 18 | 14 | 39 | 1.25 | 7.80 |
| 4076 | 0 | TW | H01L | H01L0033 | 15 | 4 | 4 | 1.5 | 1.33 |
| 4077 | 0 | HK | H01L | H01L0021 | 35 | 1 | 0 | 2 | 0.00 |
| 4078 | 0 | JP | H01L | H01L0033 | 13 | 8 | 4 | 1 | 4.00 |
| 4079 | 0 | AU | H01L | H01L0021 | 8 | 20 | 17 | 2 | 1.70 |
| 4080 | 0 | DE | H01L | H01L0033 | 17 | 19 | 11 | 3.333333333 | 0.55 |
| 4081 | 0 | TW | H01L | H01L0023 | 10 | 7 | 5 | 1 | 2.50 |
| 4082 | 0 | DE | F21V | F21V0005 | 14 | 14 | 22 | 2 | 1.83 |
| 4083 | 0 | US | H01L | H01L0021 | 23 | 49 | 13 | 4 | 3.25 |
| 4084 | 0 | TW | H01L | H01L0033 | 36 | 1 | 46 | 1 | 46.00 |
| 4085 | 0 | JP | H01L | H01L0021 | 5 | 13 | 4 | 2 | 0.67 |
| 4086 | 0 | JP | B41J | B41J0002 | 14 | 6 | 0 | 1 | 0.00 |
| 4087 | 0 | US | H01L | H01L0021 | 20 | 9 | 16 | 1.25 | 3.20 |
| 4088 | 0 | JP | B24B | B24B0027 | 4 | 7 | 0 | 1 | 0.00 |
| 4089 | 0 | JP | C09K | C09K0011 | 25 | 15 | 2 | 2 | 0.50 |
| 4090 | 0 | US | H01L | H01L0021 | 8 | 35 | 1 | 3.5 | 0.07 |
| 4091 | 0 | JP | H01L | H01L0033 | 12 | 7 | 2 | 1 | 1.00 |
| 4092 | 0 | US | H01L | H01L0051 | 23 | 18 | 9 | 1 | 1.50 |
| 4093 | 0 | US | H01L | H01L0021 | 51 | 22 | 8 | 2 | 4.00 |
| 4094 | 0 | JP | C09J | C09J0007 | 10 | 1 | 0 | 1 | 0.00 |
| 4095 | 0 | TW | H01L | H01L0033 | 5 | 3 | 18 | 1.5 | 6.00 |
| 4096 | 0 | JP | H01L | H01L0021 | 28 | 36 | 5 | 4.75 | 0.26 |
| 4097 | 0 | JP | H01L | H01L0033 | 11 | 8 | 1 | 1 | 0.33 |
| 4098 | 0 | JP | G09F | G09F0009 | 8 | 3 | 7 | 2.5 | 1.40 |
| 4099 | 0 | US | H01S | H01S0005 | 11 | 18 | 5 | 1.333333333 | 1.25 |
| 4100 | 0 | CA | H05B | H05B0033 | 137 | 163 | 10 | 1.555555556 | 0.71 |
| 4101 | 0 | US | F21K | F21K0007 | 18 | 4 | 26 | 2 | 2.60 |
| 4102 | 0 | KR | H01L | H01L0021 | 4 | 3 | 3 | 1 | 1.50 |
| 4103 | 0 | JP | H01L | H01L0021 | 21 | 9 | 1 | 1 | 0.50 |
| 4104 | 0 | JP | H01L | H01L0033 | 13 | 5 | 1 | 1 | 0.50 |
| 4105 | 0 | TW | H01L | H01L0029 | 7 | 5 | 0 | 1.5 | 0.00 |
| 4106 | 0 | JP | H01L | H01L0023 | 4 | 10 | 21 | 1 | 4.20 |
| 4107 | 0 | JP | H01L | H01L0025 | 21 | 10 | 12 | 1 | 6.00 |
| 4108 | 0 | DE | H01L | H01L0023 | 51 | 20 | 1 | 1 | 0.50 |
| 4109 | 0 | US | H01L | H01L0021 | 20 | 41 | 17 | 2 | 8.50 |
| 4110 | 0 | US | H01L | H01L0023 | 15 | 18 | 14 | 2 | 7.00 |
| 4111 | 0 | JP | H01L | H01L0033 | 55 | 11 | 4 | 2.5 | 0.80 |
| 4112 | 0 | JP | H01L | H01L0033 | 5 | 5 | 9 | 1 | 2.25 |
| 4113 | 0 | JP | H01L | H01L0033 | 9 | 11 | 0 | 1 | 0.00 |
| 4114 | 0 | JP | H01L | H01L0023 | 31 | 25 | 1 | 2.5 | 0.20 |
| 4115 | 0 | JP | H01L | H01L0023 | 6 | 10 | 1 | 2 | 0.17 |
| 4116 | 0 | US | G02B | G02B0006 | 33 | 16 | 23 | 1.333333333 | 5.75 |
| 4117 | 0 | JP | H01L | H01L0021 | 20 | 8 | 28 | 1 | 7.00 |
| 4118 | 0 | TW | H01L | H01L0033 | 10 | 17 | 2 | 1 | 0.67 |
| 4119 | 0 | JP | B41J | B41J0002 | 16 | 5 | 0 | 1 | 0.00 |
| 4120 | 0 | JP | H01L | H01L0033 | 18 | 3 | 0 | 1 | 0.00 |
| 4121 | 0 | TW | B81B | B81B0007 | 63 | 1 | 19 | 1 | 9.50 |
| 4122 | 0 | DE | H01L | H01L0023 | 28 | 4 | 43 | 1.333333333 | 10.75 |
| 4123 | 0 | DE | H01L | H01L0021 | 82 | 41 | 14 | 1.571428571 | 1.27 |
| 4124 | 0 | US | H01L | H01L0021 | 18 | 26 | 15 | 1 | 5.00 |
| 4125 | 0 | US | H01L | H01L0033 | 20 | 44 | 0 | 1.25 | 0.00 |
| 4126 | 0 | US | C30B | C30B0023 | 11 | 7 | 5 | 2 | 2.50 |
| 4127 | 0 | TW | H01L | H01L0033 | 19 | 18 | 9 | 1 | 9.00 |
| 4128 | 0 | JP | C30B | C30B0029 | 9 | 22 | 7 | 1 | 3.50 |
| 4129 | 0 | JP | H01L | H01L0033 | 35 | 10 | 12 | 1 | 4.00 |
| 4130 | 0 | US | H01L | H01L0033 | 85 | 144 | 56 | 1.2 | 9.33 |
| 4131 | 0 | US | H01L | H01L0021 | 23 | 8 | 17 | 5 | 3.40 |
| 4132 | 0 | US | H01L | H01L0023 | 24 | 25 | 12 | 1 | 6.00 |
| 4133 | 0 | JP | H01L | H01L0021 | 16 | 13 | 0 | 1 | 0.00 |
| 4134 | 0 | JP | B41J | B41J0002 | 19 | 20 | 8 | 1 | 4.00 |
| 4135 | 0 | US | H01L | H01L0033 | 36 | 8 | 2 | 1 | 0.67 |
| 4136 | 0 | JP | H01L | H01L0033 | 9 | 8 | 5 | 4.6 | 0.22 |
| 4137 | 0 | JP | H01L | H01L0033 | 15 | 9 | 21 | 1.2 | 3.50 |
| 4138 | 0 | TW | H01L | H01L0033 | 25 | 5 | 7 | 1 | 3.50 |
| 4139 | 0 | DE | H01L | H01L0023 | 13 | 12 | 2 | 1 | 1.00 |
| 4140 | 0 | JP | C23C | C23C0016 | 3 | 30 | 0 | 1.5 | 0.00 |
| 4141 | 0 | KR | H01L | H01L0033 | 10 | 10 | 5 | 1.5 | 0.83 |
| 4142 | 0 | FR | H01L | H01L0033 | 25 | 9 | 36 | 3.272727273 | 1.00 |
| 4143 | 0 | JP | H01L | H01L0021 | 7 | 10 | 15 | 1 | 7.50 |
| 4144 | 0 | CA | H01L | H01L0029 | 14 | 21 | 0 | 1.25 | 0.00 |
| 4145 | 0 | US | H01L | H01L0023 | 63 | 16 | 19 | 4 | 4.75 |
| 4146 | 0 | TW | H01L | H01L0021 | 10 | 3 | 2 | 2 | 1.00 |
| 4147 | 0 | JP | H01L | H01L0033 | 12 | 5 | 2 | 1 | 1.00 |
| 4148 | 0 | JP | C30B | C30B0025 | 24 | 9 | 0 | 1 | 0.00 |
| 4149 | 0 | JP | H01L | H01L0021 | 23 | 1 | 1 | 1.5 | 0.33 |
| 4150 | 0 | US | H01L | H01L0029 | 42 | 24 | 8 | 1.333333333 | 2.00 |
| 4151 | 0 | TW | H01L | H01L0033 | 11 | 3 | 14 | 1 | 7.00 |
| 4152 | 0 | US | H01L | H01L0021 | 20 | 20 | 23 | 1 | 11.50 |
| 4153 | 0 | JP | H01L | H01L0021 | 12 | 28 | 33 | 1 | 6.60 |
| 4154 | 0 | DE | H05B | H05B0033 | 21 | 6 | 6 | 1 | 1.20 |
| 4155 | 0 | US | C09K | C09K0011 | 26 | 25 | 66 | 1.166666667 | 9.43 |
| 4156 | 0 | US | H01L | H01L0021 | 10 | 3 | 30 | 1 | 15.00 |
| 4157 | 0 | US | H01L | H01L0021 | 42 | 1 | 10 | 2 | 2.50 |
| 4158 | 0 | VG | C08F | C08F0032 | 9 | 19 | 2 | 1 | 0.40 |
| 4159 | 0 | CA | H01L | H01L0033 | 14 | 39 | 85 | 1.285714286 | 9.44 |
| 4160 | 0 | JP | H01L | H01L0033 | 4 | 28 | 2 | 4.333333333 | 0.15 |
| 4161 | 0 | US | H01L | H01L0029 | 20 | 8 | 11 | 2 | 2.75 |
| 4162 | 0 | JP | H01L | H01L0025 | 9 | 10 | 23 | 1.8 | 2.56 |
| 4163 | 0 | US | H01L | H01L0021 | 15 | 24 | 10 | 1.8 | 0.56 |
| 4164 | 0 | TW | C09K | C09K0011 | 7 | 5 | 14 | 1 | 4.67 |
| 4165 | 0 | US | H01L | H01L0033 | 20 | 8 | 7 | 1.25 | 1.40 |
| 4166 | 0 | JP | H01L | H01L0021 | 22 | 33 | 1 | 1 | 0.20 |
| 4167 | 0 | JP | H01L | H01L0033 | 11 | 11 | 2 | 1 | 0.50 |
| 4168 | 0 | US | H01L | H01L0025 | 18 | 39 | 86 | 5 | 17.20 |
| 4169 | 0 | JP | H01L | H01L0021 | 8 | 12 | 3 | 1 | 0.50 |
| 4170 | 0 | JP | H01L | H01L0033 | 9 | 13 | 2 | 1.4 | 0.29 |
| 4171 | 0 | JP | H01L | H01L0021 | 13 | 11 | 4 | 1.5 | 1.33 |
| 4172 | 0 | JP | C08L | C08L0083 | 5 | 15 | 17 | 1.5 | 5.67 |
| 4173 | 0 | JP | C30B | C30B0029 | 2 | 9 | 4 | 1.25 | 0.80 |
| 4174 | 0 | US | H01L | H01L0029 | 15 | 2 | 0 | 2 | 0.00 |
| 4175 | 0 | JP | F21S | F21S0008 | 12 | 11 | 7 | 1 | 1.40 |
| 4176 | 0 | US | H01L | H01L0035 | 21 | 57 | 11 | 1 | 5.50 |
| 4177 | 0 | TW | H01L | H01L0033 | 10 | 8 | 10 | 2 | 5.00 |
| 4178 | 0 | JP | H01L | H01L0033 | 18 | 9 | 0 | 1 | 0.00 |
| 4179 | 0 | JP | H01L | H01L0021 | 14 | 24 | 19 | 1.2 | 3.17 |
| 4180 | 0 | JP | H05B | H05B0033 | 20 | 12 | 7 | 2 | 1.75 |
| 4181 | 0 | US | H05B | H05B0033 | 84 | 40 | 13 | 1 | 2.60 |
| 4182 | 0 | JP | H01L | H01L0023 | 6 | 11 | 6 | 1.25 | 1.20 |
| 4183 | 0 | TW | H01L | H01L0021 | 35 | 3 | 2 | 1 | 1.00 |
| 4184 | 0 | US | G02B | G02B0006 | 27 | 38 | 48 | 1 | 24.00 |
| 4185 | 0 | JP | H01L | H01L0033 | 19 | 11 | 7 | 2.333333333 | 1.00 |
| 4186 | 0 | JP | H01L | H01L0029 | 1 | 1 | 1 | 2 | 0.25 |
| 4187 | 0 | US | H01L | H01L0023 | 38 | 12 | 38 | 4 | 9.50 |
| 4188 | 0 | JP | H01L | H01L0021 | 12 | 9 | 2 | 2.5 | 0.40 |
| 4189 | 0 | JP | H01L | H01L0021 | 5 | 6 | 0 | 1 | 0.00 |
| 4190 | 0 | JP | H01L | H01L0021 | 7 | 6 | 3 | 1 | 1.50 |
| 4191 | 0 | JP | H01L | H01L0033 | 3 | 14 | 2 | 2 | 0.50 |
| 4192 | 0 | JP | G03F | G03F0001 | 3 | 18 | 4 | 1.5 | 1.33 |
| 4193 | 0 | JP | F21S | F21S0008 | 15 | 9 | 29 | 2 | 7.25 |
| 4194 | 0 | US | C09K | C09K0011 | 19 | 10 | 53 | 1.333333333 | 6.63 |
| 4195 | 0 | JP | C30B | C30B0025 | 19 | 7 | 4 | 1.5 | 1.33 |
| 4196 | 0 | US | C30B | C30B0025 | 20 | 4 | 7 | 1 | 2.33 |
| 4197 | 0 | JP | H01L | H01L0021 | 15 | 147 | 1 | 4.5 | 0.11 |
| 4198 | 0 | JP | H01L | H01L0023 | 6 | 3 | 1 | 1 | 0.50 |
| 4199 | 0 | US | H01L | H01L0021 | 24 | 30 | 3 | 1 | 3.00 |
| 4200 | 0 | US | H01L | H01L0023 | 19 | 18 | 17 | 2 | 8.50 |
| 4201 | 0 | JP | H01L | H01L0021 | 15 | 4 | 1 | 1 | 0.33 |
| 4202 | 0 | US | H01L | H01L0033 | 12 | 19 | 5 | 1 | 5.00 |
| 4203 | 0 | JP | F21V | F21V0007 | 20 | 9 | 20 | 1 | 4.00 |
| 4204 | 0 | JP | C01B | C01B0031 | 14 | 31 | 2 | 1.666666667 | 0.40 |
| 4205 | 0 | US | H01L | H01L0023 | 14 | 5 | 11 | 2 | 5.50 |
| 4206 | 0 | JP | H01L | H01L0029 | 4 | 12 | 6 | 2.666666667 | 0.75 |
| 4207 | 0 | JP | H01L | H01L0033 | 12 | 18 | 0 | 1 | 0.00 |
| 4208 | 0 | JP | H01L | H01L0033 | 16 | 24 | 2 | 1 | 1.00 |
| 4209 | 0 | JP | C01G | C01G0055 | 9 | 13 | 3 | 1.333333333 | 0.75 |
| 4210 | 0 | JP | H01L | H01L0021 | 9 | 6 | 1 | 1 | 0.50 |
| 4211 | 0 | JP | H01L | H01L0023 | 7 | 13 | 2 | 1 | 0.67 |
| 4212 | 0 | US | H01L | H01L0021 | 18 | 26 | 2 | 1.2 | 0.33 |
| 4213 | 0 | JP | C30B | C30B0029 | 14 | 8 | 7 | 1 | 1.17 |
| 4214 | 0 | US | F21V | F21V0009 | 14 | 21 | 48 | 1.333333333 | 4.00 |
| 4215 | 0 | TW | H01L | H01L0021 | 19 | 7 | 0 | 1 | 0.00 |
| 4216 | 0 | TW | H01L | H01L0033 | 10 | 2 | 7 | 1 | 3.50 |
| 4217 | 1 | US | F21L | F21L0004 | 31 | 10 | 23 | 4.444444444 | 0.58 |
| 4218 | 0 | US | H01L | H01L0025 | 32 | 41 | 101 | 5 | 20.20 |
| 4219 | 0 | JP | G03C | G03C0001 | 17 | 5 | 0 | 1 | 0.00 |
| 4220 | 0 | TW | H01L | H01L0021 | 20 | 4 | 2 | 7 | 0.14 |
| 4221 | 0 | US | B81C | B81C0001 | 6 | 31 | 16 | 1.25 | 3.20 |
| 4222 | 0 | CA | H01L | H01L0023 | 18 | 23 | 6 | 1 | 1.20 |
| 4223 | 0 | TW | H01L | H01L0033 | 6 | 2 | 7 | 1 | 7.00 |
| 4224 | 0 | JP | H01L | H01L0021 | 7 | 56 | 5 | 1.5 | 1.67 |
| 4225 | 0 | JP | H01L | H01L0021 | 16 | 16 | 2 | 2 | 0.50 |
| 4226 | 0 | CA | H01L | H01L0021 | 28 | 18 | 13 | 1.666666667 | 2.60 |
| 4227 | 0 | JP | G02B | G02B0006 | 16 | 7 | 8 | 1 | 8.00 |
| 4228 | 0 | US | H01L | H01L0021 | 84 | 17 | 67 | 4 | 16.75 |
| 4229 | 0 | JP | H01L | H01L0021 | 7 | 9 | 1 | 1.8 | 0.11 |
| 4230 | 0 | JP | C30B | C30B0029 | 9 | 3 | 5 | 1.333333333 | 1.25 |
| 4231 | 0 | TW | H01J | H01J0009 | 12 | 2 | 1 | 1 | 1.00 |
| 4232 | 0 | JP | H01L | H01L0033 | 32 | 95 | 0 | 3.4 | 0.00 |
| 4233 | 0 | JP | H01L | H01L0023 | 11 | 8 | 6 | 1 | 2.00 |
| 4234 | 0 | JP | H01L | H01L0021 | 16 | 6 | 2 | 1.5 | 0.67 |
| 4235 | 0 | JP | H01L | H01L0033 | 14 | 70 | 2 | 1 | 0.33 |
| 4236 | 0 | US | C30B | C30B0023 | 6 | 9 | 1 | 1.333333333 | 0.25 |
| 4237 | 0 | JP | H01J | H01J0001 | 4 | 11 | 4 | 1.25 | 0.80 |
| 4238 | 0 | US | G01T | G01T0001 | 24 | 20 | 41 | 1.2 | 6.83 |
| 4239 | 0 | CA | H01L | H01L0033 | 27 | 6 | 10 | 1 | 2.50 |
| 4240 | 0 | JP | H01L | H01L0033 | 4 | 11 | 7 | 2.333333333 | 1.00 |
| 4241 | 0 | US | H01L | H01L0021 | 28 | 26 | 11 | 3 | 3.67 |
| 4242 | 0 | US | H01L | H01L0027 | 33 | 47 | 0 | 1 | 0.00 |
| 4243 | 0 | US | H01L | H01L0021 | 40 | 50 | 3 | 3 | 1.00 |
| 4244 | 0 | US | H01L | H01L0023 | 17 | 25 | 34 | 1.166666667 | 4.86 |
| 4245 | 0 | JP | H01L | H01L0021 | 8 | 402 | 7 | 1.285714286 | 0.78 |
| 4246 | 0 | JP | H01L | H01L0033 | 17 | 26 | 5 | 1 | 2.50 |
| 4247 | 0 | TW | H01L | H01L0033 | 14 | 4 | 11 | 2 | 5.50 |
| 4248 | 0 | JP | H01L | H01L0033 | 13 | 15 | 3 | 1.666666667 | 0.60 |
| 4249 | 0 | JP | H01L | H01L0033 | 20 | 18 | 7 | 1.5 | 2.33 |
| 4250 | 0 | JP | H01L | H01L0021 | 23 | 145 | 2 | 4.5 | 0.22 |
| 4251 | 0 | US | H01L | H01L0023 | 5 | 40 | 0 | 9 | 0.00 |
| 4252 | 0 | DE | H01L | H01L0033 | 18 | 8 | 18 | 1 | 6.00 |
| 4253 | 0 | US | H01L | H01L0023 | 16 | 9 | 4 | 6 | 0.67 |
| 4254 | 0 | TW | H01L | H01L0033 | 18 | 4 | 5 | 1.5 | 1.67 |
| 4255 | 0 | JP | H01L | H01L0033 | 6 | 28 | 12 | 1.375 | 1.09 |
| 4256 | 0 | TW | B81B | B81B0001 | 12 | 7 | 4 | 1 | 0.80 |
| 4257 | 0 | JP | H01L | H01L0021 | 10 | 3 | 14 | 1.5 | 4.67 |
| 4258 | 0 | US | G03D | G03D0005 | 20 | 3 | 5 | 1.5 | 1.67 |
| 4259 | 0 | JP | H01L | H01L0021 | 18 | 15 | 13 | 1.5 | 4.33 |
| 4260 | 0 | JP | H01L | H01L0025 | 31 | 13 | 14 | 1.333333333 | 3.50 |
| 4261 | 0 | JP | C09K | C09K0011 | 23 | 23 | 7 | 1 | 3.50 |
| 4262 | 0 | JP | G03F | G03F0007 | 41 | 14 | 32 | 1 | 8.00 |
| 4263 | 0 | JP | C25D | C25D0011 | 11 | 25 | 1 | 1 | 0.25 |
| 4264 | 0 | US | G02F | G02F0002 | 11 | 35 | 21 | 1 | 21.00 |
| 4265 | 0 | DE | H01L | H01L0033 | 31 | 21 | 4 | 1.166666667 | 0.57 |
| 4266 | 0 | KR | H01L | H01L0021 | 21 | 4 | 9 | 1 | 3.00 |
| 4267 | 0 | JP | C30B | C30B0025 | 8 | 15 | 5 | 1 | 2.50 |
| 4268 | 0 | JP | H01L | H01L0021 | 10 | 19 | 1 | 1 | 0.25 |
| 4269 | 0 | US | H01L | H01L0021 | 34 | 14 | 4 | 1.2 | 0.67 |
| 4270 | 0 | US | F21K | F21K0007 | 3 | 17 | 10 | 1.25 | 2.00 |
| 4271 | 0 | US | C09K | C09K0011 | 49 | 147 | 53 | 2.761904762 | 0.91 |
| 4272 | 0 | TW | H01L | H01L0021 | 18 | 4 | 4 | 1 | 1.33 |
| 4273 | 0 | US | H01L | H01L0051 | 24 | 13 | 27 | 1 | 5.40 |
| 4274 | 0 | JP | H01L | H01L0033 | 15 | 11 | 3 | 1 | 0.75 |
| 4275 | 0 | CA | H01L | H01L0027 | 50 | 19 | 65 | 1 | 65.00 |
| 4276 | 0 | JP | H01L | H01L0023 | 23 | 34 | 20 | 1.125 | 2.22 |
| 4277 | 0 | JP | H01L | H01L0023 | 8 | 6 | 3 | 1 | 0.75 |
| 4278 | 0 | US | H01L | H01L0021 | 41 | 35 | 2 | 3.5 | 0.14 |
| 4279 | 0 | GB | B41J | B41J0002 | 77 | 30 | 25 | 1.111111111 | 2.50 |
| 4280 | 0 | US | H01S | H01S0005 | 10 | 20 | 70 | 1.5 | 23.33 |
| 4281 | 0 | US | C30B | C30B0023 | 8 | 21 | 0 | 1 | 0.00 |
| 4282 | 0 | US | H01L | H01L0021 | 9 | 43 | 2 | 9 | 0.22 |
| 4283 | 0 | US | H01L | H01L0033 | 59 | 156 | 25 | 1.2 | 4.17 |
| 4284 | 0 | DE | H01L | H01L0033 | 7 | 21 | 20 | 1.285714286 | 2.22 |
| 4285 | 1 | DE | H01L | H01L0033 | 25 | 21 | 24 | 1.5 | 2.00 |
| 4286 | 0 | JP | H01L | H01L0021 | 23 | 26 | 6 | 2.666666667 | 0.75 |
| 4287 | 0 | US | H01L | H01L0023 | 14 | 28 | 0 | 4 | 0.00 |
| 4288 | 0 | DE | C09K | C09K0011 | 29 | 8 | 18 | 1 | 4.50 |
| 4289 | 0 | JP | C30B | C30B0025 | 84 | 16 | 5 | 1.125 | 0.56 |
| 4290 | 0 | JP | C23C | C23C0016 | 24 | 9 | 2 | 1 | 0.67 |
| 4291 | 0 | CH | G03F | G03F0007 | 20 | 15 | 2 | 1.166666667 | 0.29 |
| 4292 | 0 | JP | H01L | H01L0033 | 4 | 59 | 5 | 3 | 0.83 |
| 4293 | 0 | TW | H01L | H01L0033 | 7 | 2 | 7 | 1 | 3.50 |
| 4294 | 0 | TW | H01L | H01L0033 | 15 | 3 | 0 | 1 | 0.00 |
| 4295 | 0 | US | C09K | C09K0011 | 89 | 158 | 14 | 2.761904762 | 0.24 |
| 4296 | 0 | US | H01S | H01S0005 | 13 | 40 | 1 | 2 | 0.50 |
| 4297 | 0 | US | H01L | H01L0033 | 26 | 24 | 116 | 5 | 23.20 |
| 4298 | 0 | JP | H01L | H01L0033 | 14 | 7 | 1 | 2 | 0.25 |
| 4299 | 0 | JP | C30B | C30B0025 | 25 | 17 | 1 | 1 | 0.50 |
| 4300 | 0 | JP | H01L | H01L0033 | 15 | 6 | 2 | 1 | 0.33 |
| 4301 | 0 | US | H01L | H01L0033 | 10 | 13 | 2 | 1.25 | 0.40 |
| 4302 | 0 | JP | H01L | H01L0023 | 17 | 7 | 1 | 1 | 0.50 |
| 4303 | 0 | JP | H01L | H01L0021 | 13 | 6 | 9 | 1 | 1.80 |
| 4304 | 0 | DE | H01L | H01L0033 | 13 | 18 | 14 | 2 | 1.17 |
| 4305 | 0 | DE | H01L | H01L0033 | 12 | 15 | 1 | 1.166666667 | 0.14 |
| 4306 | 0 | JP | H01L | H01L0033 | 27 | 17 | 18 | 1.333333333 | 4.50 |
| 4307 | 0 | US | H01L | H01L0021 | 16 | 124 | 58 | 1.571428571 | 5.27 |
| 4308 | 0 | JP | C30B | C30B0029 | 13 | 124 | 2 | 1 | 1.00 |
| 4309 | 0 | SG | H01L | H01L0021 | 10 | 22 | 1 | 2 | 0.50 |
| 4310 | 0 | JP | H01L | H01L0033 | 16 | 13 | 1 | 1 | 0.25 |
| 4311 | 0 | US | C30B | C30B0025 | 32 | 18 | 31 | 1 | 31.00 |
| 4312 | 0 | JP | H01L | H01L0021 | 12 | 8 | 4 | 2.5 | 0.80 |
| 4313 | 0 | TW | H01L | H01L0025 | 18 | 7 | 8 | 1 | 2.67 |
| 4314 | 0 | KR | H01L | H01L0027 | 14 | 2 | 3 | 1.5 | 1.00 |
| 4315 | 0 | JP | H05B | H05B0033 | 8 | 27 | 16 | 2 | 4.00 |
| 4316 | 0 | JP | C30B | C30B0025 | 22 | 33 | 4 | 1.625 | 0.31 |
| 4317 | 0 | US | H01L | H01L0023 | 11 | 18 | 1 | 6 | 0.17 |
| 4318 | 0 | TW | H01L | H01L0021 | 17 | 5 | 2 | 1 | 2.00 |
| 4319 | 0 | JP | H01L | H01L0021 | 19 | 10 | 6 | 1 | 1.50 |
| 4320 | 0 | US | H01S | H01S0005 | 35 | 27 | 1 | 1 | 0.50 |
| 4321 | 0 | DE | H01L | H01L0021 | 19 | 39 | 0 | 1.166666667 | 0.00 |
| 4322 | 0 | JP | H01L | H01L0033 | 8 | 5 | 3 | 1 | 1.50 |
| 4323 | 0 | TW | H01L | H01L0033 | 51 | 3 | 4 | 1 | 2.00 |
| 4324 | 0 | US | H01L | H01L0021 | 9 | 13 | 58 | 1.142857143 | 7.25 |
| 4325 | 0 | JP | H01L | H01L0033 | 6 | 6 | 0 | 1.5 | 0.00 |
| 4326 | 0 | JP | H01L | H01L0021 | 3 | 33 | 0 | 1.5 | 0.00 |
| 4327 | 0 | US | H01L | H01L0023 | 26 | 28 | 70 | 3 | 23.33 |
| 4328 | 0 | US | B29C | B29C0045 | 6 | 43 | 17 | 1.5 | 1.89 |
| 4329 | 0 | US | C23C | C23C0018 | 4 | 8 | 2 | 1 | 2.00 |
| 4330 | 0 | JP | H01L | H01L0033 | 6 | 22 | 6 | 1.5 | 2.00 |
| 4331 | 0 | HK | H01L | H01L0021 | 40 | 3 | 0 | 2 | 0.00 |
| 4332 | 0 | TW | H01L | H01L0033 | 6 | 8 | 30 | 1 | 15.00 |
| 4333 | 0 | US | C08L | C08L0035 | 18 | 1 | 6 | 3 | 2.00 |
| 4334 | 0 | JP | G09F | G09F0009 | 24 | 57 | 5 | 2 | 1.25 |
| 4335 | 0 | JP | H05B | H05B0033 | 20 | 10 | 12 | 1.333333333 | 3.00 |
| 4336 | 0 | JP | H05K | H05K0001 | 22 | 69 | 5 | 2.166666667 | 0.38 |
| 4337 | 0 | US | H01L | H01L0021 | 43 | 154 | 14 | 4 | 3.50 |
| 4338 | 0 | DE | G03F | G03F0001 | 43 | 4 | 3 | 1 | 1.50 |
| 4339 | 0 | JP | H01L | H01L0025 | 6 | 23 | 6 | 2.333333333 | 0.86 |
| 4340 | 0 | JP | H01L | H01L0021 | 5 | 12 | 1 | 1.5 | 0.33 |
| 4341 | 0 | JP | H01L | H01L0051 | 32 | 48 | 20 | 2.166666667 | 1.54 |
| 4342 | 0 | JP | H01L | H01L0033 | 2 | 7 | 1 | 4 | 0.13 |
| 4343 | 0 | US | H01L | H01L0021 | 8 | 6 | 1 | 1.666666667 | 0.20 |
| 4344 | 0 | JP | H01L | H01L0025 | 22 | 32 | 48 | 1 | 6.86 |
| 4345 | 0 | JP | H01L | H01L0021 | 26 | 10 | 1 | 1.571428571 | 0.09 |
| 4346 | 0 | TW | H01L | H01L0021 | 6 | 13 | 1 | 1 | 1.00 |
| 4347 | 0 | JP | H01L | H01L0021 | 34 | 3 | 7 | 1 | 3.50 |
| 4348 | 0 | JP | H01L | H01L0023 | 13 | 9 | 2 | 1 | 1.00 |
| 4349 | 0 | JP | G01B | G01B0011 | 14 | 15 | 2 | 1 | 1.00 |
| 4350 | 0 | DE | H01L | H01L0033 | 8 | 59 | 24 | 5.857142857 | 0.59 |
| 4351 | 0 | JP | H01L | H01L0033 | 20 | 45 | 0 | 1.5 | 0.00 |
| 4352 | 0 | CA | H01L | H01L0033 | 36 | 5 | 11 | 1 | 2.75 |
| 4353 | 0 | US | F21K | F21K0007 | 3 | 10 | 7 | 1.25 | 1.40 |
| 4354 | 0 | JP | H01L | H01L0021 | 5 | 14 | 1 | 2.5 | 0.20 |
| 4355 | 0 | JP | H01L | H01L0033 | 8 | 13 | 4 | 1.5 | 1.33 |
| 4356 | 0 | TW | H01L | H01L0033 | 16 | 3 | 1 | 1.333333333 | 0.25 |
| 4357 | 0 | JP | H01L | H01L0033 | 15 | 7 | 0 | 1 | 0.00 |
| 4358 | 0 | JP | G02B | G02B0006 | 7 | 13 | 3 | 1.5 | 1.00 |
| 4359 | 0 | JP | H01L | H01L0033 | 16 | 22 | 0 | 1.666666667 | 0.00 |
| 4360 | 0 | US | H01L | H01L0021 | 28 | 4 | 0 | 2 | 0.00 |
| 4361 | 0 | US | H01L | H01L0023 | 17 | 18 | 2 | 6 | 0.33 |
| 4362 | 0 | JP | H01L | H01L0021 | 3 | 129 | 4 | 1 | 1.00 |
| 4363 | 0 | JP | H01L | H01L0021 | 2 | 5 | 0 | 1.5 | 0.00 |
| 4364 | 0 | US | G02B | G02B0006 | 22 | 31 | 5 | 11.33333333 | 0.15 |
| 4365 | 0 | US | H01L | H01L0021 | 20 | 2 | 4 | 1 | 4.00 |
| 4366 | 0 | US | H01L | H01L0033 | 51 | 42 | 10 | 3 | 3.33 |
| 4367 | 0 | JP | H01L | H01L0021 | 3 | 6 | 3 | 1 | 0.75 |
| 4368 | 0 | US | G02B | G02B0006 | 21 | 3 | 4 | 1.25 | 0.80 |
| 4369 | 0 | JP | H01L | H01L0033 | 19 | 6 | 1 | 1 | 0.50 |
| 4370 | 0 | JP | H01L | H01L0021 | 10 | 4 | 0 | 1.5 | 0.00 |
| 4371 | 0 | US | G09F | G09F0013 | 18 | 37 | 37 | 1.666666667 | 3.70 |
| 4372 | 0 | DE | H01L | H01L0023 | 9 | 11 | 2 | 1 | 0.67 |
| 4373 | 0 | US | C09K | C09K0011 | 59 | 9 | 17 | 1 | 2.83 |
| 4374 | 0 | JP | H01L | H01L0021 | 17 | 7 | 4 | 1.4 | 0.57 |
| 4375 | 0 | JP | H01L | H01L0023 | 9 | 12 | 3 | 1.5 | 1.00 |
| 4376 | 0 | DE | F21K | F21K0099 | 8 | 9 | 17 | 1.5 | 5.67 |
| 4377 | 0 | US | H01L | H01L0033 | 23 | 1 | 1 | 1 | 1.00 |
| 4378 | 0 | JP | H01L | H01L0021 | 10 | 7 | 15 | 1 | 5.00 |
| 4379 | 0 | US | G02B | G02B0005 | 19 | 10 | 4 | 1.5 | 0.67 |
| 4380 | 0 | TW | C30B | C30B0019 | 18 | 1 | 0 | 2 | 0.00 |
| 4381 | 0 | JP | H01L | H01L0021 | 7 | 10 | 1 | 2.75 | 0.09 |
| 4382 | 0 | JP | H01L | H01L0021 | 3 | 4 | 0 | 1 | 0.00 |
| 4383 | 0 | US | H01L | H01L0023 | 5 | 44 | 0 | 9 | 0.00 |
| 4384 | 0 | US | F21K | F21K0007 | 21 | 13 | 70 | 1 | 35.00 |
| 4385 | 0 | DE | H01L | H01L0033 | 20 | 41 | 5 | 1.285714286 | 0.56 |
| 4386 | 0 | US | C23C | C23C0016 | 114 | 17 | 2 | 1.666666667 | 0.20 |
| 4387 | 0 | JP | H01L | H01L0023 | 10 | 5 | 3 | 1.25 | 0.60 |
| 4388 | 0 | TW | H01L | H01L0021 | 25 | 12 | 2 | 1 | 1.00 |
| 4389 | 0 | JP | H01L | H01L0027 | 77 | 32 | 5 | 2 | 0.83 |
| 4390 | 0 | JP | H01L | H01L0021 | 33 | 141 | 1 | 4.5 | 0.11 |
| 4391 | 0 | JP | H01L | H01L0021 | 1 | 3 | 2 | 1 | 1.00 |
| 4392 | 0 | JP | H01L | H01L0033 | 1 | 3 | 1 | 1 | 0.20 |
| 4393 | 0 | JP | G03F | G03F0001 | 14 | 6 | 1 | 1 | 0.50 |
| 4394 | 0 | US | H01L | H01L0021 | 111 | 20 | 23 | 2 | 1.92 |
| 4395 | 0 | US | G02B | G02B0001 | 7 | 9 | 12 | 1.25 | 2.40 |
| 4396 | 1 | DE | H01L | H01L0033 | 20 | 16 | 13 | 1.428571429 | 1.30 |
| 4397 | 0 | JP | H01L | H01L0021 | 6 | 28 | 1 | 1 | 0.50 |
| 4398 | 0 | TW | H01L | H01L0033 | 12 | 4 | 3 | 1.5 | 1.00 |
| 4399 | 0 | JP | H01L | H01L0033 | 3 | 53 | 6 | 5 | 0.30 |
| 4400 | 0 | JP | H01L | H01L0033 | 12 | 4 | 0 | 2 | 0.00 |
| 4401 | 0 | DE | H01L | H01L0023 | 30 | 7 | 1 | 1.333333333 | 0.25 |
| 4402 | 0 | DE | H01L | H01L0023 | 13 | 10 | 14 | 2.333333333 | 2.00 |
| 4403 | 0 | JP | H01L | H01L0023 | 13 | 27 | 4 | 1.25 | 0.80 |
| 4404 | 0 | JP | H01L | H01L0033 | 6 | 6 | 34 | 1 | 17.00 |
| 4405 | 0 | US | H01L | H01L0023 | 23 | 11 | 7 | 2 | 3.50 |
| 4406 | 0 | KR | C30B | C30B0029 | 14 | 10 | 5 | 1 | 1.67 |
| 4407 | 0 | TW | H01L | H01L0021 | 23 | 17 | 1 | 1 | 1.00 |
| 4408 | 1 | US | G01N | G01N0033 | 27 | 40 | 25 | 3.666666667 | 1.14 |
| 4409 | 0 | US | H01L | H01L0025 | 23 | 4 | 13 | 1 | 13.00 |
| 4410 | 0 | TW | H01L | H01L0025 | 7 | 8 | 1 | 1 | 1.00 |
| 4411 | 0 | US | H01L | H01L0021 | 22 | 45 | 27 | 3 | 9.00 |
| 4412 | 0 | US | H01L | H01L0021 | 10 | 18 | 0 | 1.125 | 0.00 |
| 4413 | 0 | KR | H01L | H01L0021 | 32 | 47 | 6 | 1.4 | 0.86 |
| 4414 | 0 | US | H01L | H01L0033 | 90 | 37 | 88 | 18.83333333 | 0.78 |
| 4415 | 0 | JP | H01L | H01L0051 | 21 | 40 | 2 | 1 | 1.00 |
| 4416 | 0 | MI | B60Q | B60Q0003 | 8 | 11 | 2 | 2 | 1.00 |
| 4417 | 0 | JP | H01L | H01L0023 | 19 | 23 | 3 | 2.5 | 0.60 |
| 4418 | 0 | US | C23C | C23C0016 | 18 | 18 | 1 | 1.666666667 | 0.10 |
| 4419 | 0 | US | H05K | H05K0003 | 9 | 2 | 29 | 1 | 29.00 |
| 4420 | 0 | CA | H01L | H01L0025 | 22 | 29 | 58 | 5.166666667 | 1.87 |
| 4421 | 0 | JP | H01L | H01L0033 | 24 | 34 | 5 | 2 | 0.83 |
| 4422 | 0 | TW | H01L | H01L0031 | 10 | 4 | 0 | 1 | 0.00 |
| 4423 | 0 | JP | H01L | H01L0051 | 11 | 11 | 3 | 1.75 | 0.43 |
| 4424 | 0 | TW | H01L | H01L0021 | 13 | 3 | 2 | 1 | 1.00 |
| 4425 | 0 | JP | H01L | H01L0021 | 8 | 58 | 3 | 1.5 | 1.00 |
| 4426 | 0 | TW | H01L | H01L0029 | 6 | 3 | 0 | 1 | 0.00 |
| 4427 | 0 | US | H01L | H01L0021 | 13 | 28 | 3 | 1.8 | 0.17 |
| 4428 | 0 | JP | H01L | H01L0021 | 8 | 17 | 2 | 1.5 | 0.67 |
| 4429 | 0 | US | H01L | H01L0023 | 24 | 40 | 6 | 3 | 2.00 |
| 4430 | 0 | VG | G03F | G03F0007 | 26 | 13 | 1 | 1 | 0.14 |
| 4431 | 0 | JP | H01L | H01L0033 | 7 | 12 | 0 | 1 | 0.00 |
| 4432 | 0 | US | H01L | H01L0021 | 23 | 150 | 45 | 3 | 3.75 |
| 4433 | 0 | US | H01L | H01L0023 | 20 | 33 | 23 | 3 | 7.67 |
| 4434 | 0 | TW | H05B | H05B0041 | 20 | 2 | 20 | 1 | 20.00 |
| 4435 | 0 | KR | H01L | H01L0027 | 24 | 2 | 1 | 1.333333333 | 0.25 |
| 4436 | 0 | TW | H01L | H01L0021 | 24 | 4 | 11 | 1 | 11.00 |
| 4437 | 0 | JP | H01L | H01L0023 | 9 | 3 | 2 | 1 | 1.00 |
| 4438 | 0 | US | G01J | G01J0001 | 38 | 23 | 1 | 1.5 | 0.11 |
| 4439 | 0 | US | H01L | H01L0033 | 39 | 1 | 31 | 3 | 10.33 |
| 4440 | 0 | US | H01L | H01L0021 | 20 | 27 | 4 | 4 | 1.00 |
| 4441 | 0 | JP | H01L | H01L0033 | 4 | 3 | 7 | 1.5 | 2.33 |
| 4442 | 0 | US | C23C | C23C0016 | 12 | 4 | 3 | 1.333333333 | 0.75 |
| 4443 | 0 | JP | H01L | H01L0023 | 19 | 28 | 12 | 1 | 3.00 |
| 4444 | 0 | TW | H01L | H01L0021 | 10 | 7 | 5 | 4 | 0.42 |
| 4445 | 0 | JP | F21V | F21V0029 | 19 | 8 | 6 | 1 | 3.00 |
| 4446 | 0 | JP | G02B | G02B0006 | 10 | 18 | 0 | 1.166666667 | 0.00 |
| 4447 | 0 | US | H01L | H01L0021 | 3 | 43 | 7 | 2.333333333 | 1.00 |
| 4448 | 0 | JP | H01L | H01L0021 | 34 | 26 | 3 | 1.4 | 0.43 |
| 4449 | 0 | US | H01L | H01L0023 | 14 | 51 | 1 | 9 | 0.11 |
| 4450 | 0 | TW | H01L | H01L0027 | 9 | 2 | 0 | 1 | 0.00 |
| 4451 | 0 | JP | H01L | H01L0021 | 20 | 14 | 0 | 1 | 0.00 |
| 4452 | 0 | JP | C09K | C09K0011 | 16 | 10 | 0 | 2.5 | 0.00 |
| 4453 | 0 | JP | H01L | H01L0021 | 5 | 15 | 5 | 1 | 1.00 |
| 4454 | 0 | TW | H01L | H01L0033 | 6 | 5 | 5 | 2 | 2.50 |
| 4455 | 0 | HK | H01L | H01L0033 | 8 | 2 | 13 | 1.5 | 4.33 |
| 4456 | 0 | JP | H01L | H01L0021 | 10 | 15 | 10 | 1 | 5.00 |
| 4457 | 0 | US | B29C | B29C0065 | 7 | 1 | 1 | 2 | 0.50 |
| 4458 | 0 | JP | H01L | H01L0033 | 11 | 14 | 23 | 1.5 | 7.67 |
| 4459 | 0 | US | B81B | B81B0007 | 29 | 5 | 21 | 1 | 21.00 |
| 4460 | 0 | JP | H01L | H01L0021 | 10 | 5 | 3 | 1 | 0.75 |
| 4461 | 0 | JP | H01L | H01L0029 | 28 | 10 | 35 | 1 | 17.50 |
| 4462 | 0 | JP | C09K | C09K0011 | 5 | 16 | 9 | 1.5 | 3.00 |
| 4463 | 0 | JP | G02B | G02B0006 | 5 | 8 | 6 | 1 | 3.00 |
| 4464 | 0 | DE | H01L | H01L0021 | 13 | 8 | 3 | 1 | 0.60 |
| 4465 | 0 | JP | H01L | H01L0033 | 7 | 58 | 8 | 5 | 0.80 |
| 4466 | 0 | US | C09K | C09K0011 | 30 | 21 | 25 | 1 | 12.50 |
| 4467 | 0 | DE | H01L | H01L0033 | 12 | 15 | 3 | 1.666666667 | 0.60 |
| 4468 | 0 | JP | F21S | F21S0008 | 14 | 13 | 4 | 1.666666667 | 0.80 |
| 4469 | 0 | US | C23C | C23C0026 | 22 | 43 | 1 | 1.4 | 0.14 |
| 4470 | 0 | DE | H01L | H01L0033 | 33 | 9 | 0 | 1 | 0.00 |
| 4471 | 0 | JP | H01L | H01L0021 | 18 | 123 | 2 | 4.5 | 0.22 |
| 4472 | 0 | JP | H01L | H01L0023 | 5 | 13 | 3 | 1.2 | 0.50 |
| 4473 | 0 | TW | H01L | H01L0033 | 19 | 13 | 4 | 1.333333333 | 1.00 |
| 4474 | 0 | JP | H01L | H01L0033 | 8 | 4 | 1 | 2 | 0.25 |
| 4475 | 0 | US | H01L | H01L0023 | 33 | 16 | 2 | 3 | 0.67 |
| 4476 | 0 | JP | H01L | H01L0021 | 7 | 82 | 18 | 2 | 4.50 |
| 4477 | 0 | US | C30B | C30B0025 | 23 | 22 | 3 | 1 | 1.50 |
| 4478 | 0 | DE | H01L | H01L0023 | 27 | 11 | 1 | 1 | 0.50 |
| 4479 | 0 | JP | H01L | H01L0023 | 13 | 7 | 1 | 1 | 0.25 |
| 4480 | 0 | US | H01L | H01L0033 | 19 | 1 | 12 | 2 | 6.00 |
| 4481 | 0 | US | C08F | C08F0214 | 66 | 27 | 29 | 1.125 | 3.22 |
| 4482 | 0 | US | H01L | H01L0021 | 6 | 4 | 7 | 2 | 3.50 |
| 4483 | 0 | US | H01L | H01L0021 | 14 | 4 | 2 | 2 | 1.00 |
| 4484 | 0 | JP | C30B | C30B0025 | 24 | 10 | 0 | 1.6 | 0.00 |
| 4485 | 0 | US | F21K | F21K0007 | 19 | 23 | 42 | 1.2 | 7.00 |
| 4486 | 0 | US | H01L | H01L0021 | 14 | 23 | 6 | 1 | 3.00 |
| 4487 | 0 | JP | H01L | H01L0033 | 17 | 46 | 18 | 2 | 2.25 |
| 4488 | 0 | JP | H01L | H01L0033 | 22 | 18 | 2 | 1 | 0.50 |
| 4489 | 0 | JP | G02B | G02B0006 | 11 | 4 | 4 | 1 | 2.00 |
| 4490 | 0 | JP | C30B | C30B0025 | 27 | 20 | 1 | 2.75 | 0.09 |
| 4491 | 0 | JP | H01L | H01L0023 | 3 | 38 | 12 | 1.5 | 2.00 |
| 4492 | 0 | JP | H01L | H01L0033 | 8 | 8 | 2 | 1 | 0.50 |
| 4493 | 0 | JP | H01L | H01L0033 | 9 | 19 | 3 | 3 | 0.50 |
| 4494 | 0 | JP | H01L | H01L0021 | 24 | 37 | 4 | 1.4 | 0.57 |
| 4495 | 0 | US | F21L | F21L0004 | 16 | 7 | 17 | 4.444444444 | 0.43 |
| 4496 | 0 | US | G03D | G03D0005 | 12 | 6 | 0 | 1.5 | 0.00 |
| 4497 | 0 | JP | H01L | H01L0033 | 24 | 8 | 32 | 1.5 | 10.67 |
| 4498 | 0 | JP | G02B | G02B0006 | 16 | 6 | 0 | 1 | 0.00 |
| 4499 | 0 | US | C30B | C30B0023 | 56 | 10 | 2 | 1.5 | 0.67 |
| 4500 | 0 | JP | F21V | F21V0029 | 32 | 11 | 3 | 1 | 0.50 |
| 4501 | 0 | JP | B60Q | B60Q0003 | 26 | 6 | 2 | 2.5 | 0.40 |
| 4502 | 0 | JP | G02B | G02B0006 | 6 | 14 | 0 | 1.333333333 | 0.00 |
| 4503 | 0 | JP | C30B | C30B0029 | 7 | 6 | 2 | 1 | 0.33 |
| 4504 | 0 | CA | H01L | H01L0033 | 5 | 74 | 6 | 2.2 | 0.55 |
| 4505 | 0 | JP | H01L | H01L0033 | 10 | 12 | 6 | 1.5 | 2.00 |
| 4506 | 0 | JP | H01L | H01L0033 | 29 | 32 | 6 | 4 | 0.50 |
| 4507 | 0 | KR | H01L | H01L0023 | 21 | 9 | 4 | 1 | 1.33 |
| 4508 | 0 | US | H01L | H01L0021 | 3 | 116 | 0 | 1 | 0.00 |
| 4509 | 0 | US | H01L | H01L0033 | 17 | 27 | 5 | 1 | 2.50 |
| 4510 | 0 | JP | H01L | H01L0033 | 16 | 19 | 1 | 1 | 0.20 |
| 4511 | 0 | DE | H05B | H05B0033 | 34 | 41 | 6 | 1 | 0.86 |
| 4512 | 0 | US | C08G | C08G0059 | 35 | 24 | 3 | 1.25 | 0.60 |
| 4513 | 0 | JP | H01L | H01L0033 | 42 | 26 | 25 | 4 | 2.08 |
| 4514 | 0 | US | C23C | C23C0016 | 19 | 136 | 11 | 2 | 5.50 |
| 4515 | 0 | US | H01L | H01L0021 | 19 | 49 | 1 | 3 | 0.33 |
| 4516 | 0 | US | H01L | H01L0021 | 47 | 8 | 8 | 1.166666667 | 1.14 |
| 4517 | 0 | US | H01L | H01L0033 | 21 | 4 | 16 | 1 | 3.20 |
| 4518 | 0 | US | H01L | H01L0023 | 27 | 4 | 13 | 1.2 | 2.17 |
| 4519 | 0 | US | H01L | H01L0023 | 12 | 14 | 9 | 4 | 2.25 |
| 4520 | 0 | JP | H01L | H01L0051 | 36 | 38 | 4 | 1.5 | 1.33 |
| 4521 | 0 | JP | H01L | H01L0021 | 8 | 35 | 16 | 2 | 2.00 |
| 4522 | 0 | JP | H01L | H01L0033 | 8 | 2 | 0 | 1 | 0.00 |
| 4523 | 0 | US | C07F | C07F0005 | 25 | 14 | 3 | 1 | 0.75 |
| 4524 | 0 | US | F21K | F21K0007 | 6 | 18 | 2 | 1.25 | 0.40 |
| 4525 | 0 | US | H01L | H01L0051 | 19 | 14 | 2 | 1 | 1.00 |
| 4526 | 0 | JP | H05B | H05B0033 | 33 | 11 | 14 | 1.5 | 4.67 |
| 4527 | 0 | JP | H01L | H01L0027 | 2 | 21 | 3 | 1.25 | 0.60 |
| 4528 | 0 | US | H01L | H01L0021 | 23 | 6 | 2 | 1 | 2.00 |
| 4529 | 0 | US | H01L | H01L0021 | 118 | 141 | 8 | 18.83333333 | 0.07 |
| 4530 | 0 | JP | H01L | H01L0029 | 16 | 11 | 1 | 1.4 | 0.14 |
| 4531 | 0 | JP | H01L | H01L0033 | 2 | 22 | 1 | 2 | 0.25 |
| 4532 | 0 | JP | H01L | H01L0021 | 20 | 19 | 1 | 1.5 | 0.33 |
| 4533 | 0 | US | B81B | B81B0007 | 33 | 34 | 10 | 2.7 | 0.37 |
| 4534 | 0 | JP | G02F | G02F0001 | 36 | 26 | 10 | 1.5 | 3.33 |
| 4535 | 0 | TW | H01J | H01J0001 | 12 | 1 | 0 | 1 | 0.00 |
| 4536 | 0 | JP | C30B | C30B0029 | 10 | 9 | 1 | 1 | 0.20 |
| 4537 | 0 | US | H01L | H01L0021 | 14 | 10 | 5 | 4 | 1.25 |
| 4538 | 0 | FR | C30B | C30B0029 | 23 | 26 | 7 | 2.75 | 0.32 |
| 4539 | 0 | JP | H01L | H01L0033 | 10 | 8 | 2 | 1 | 1.00 |
| 4540 | 0 | JP | H01L | H01L0021 | 8 | 1 | 0 | 1 | 0.00 |
| 4541 | 1 | US | F21L | F21L0004 | 15 | 6 | 21 | 4.444444444 | 0.53 |
| 4542 | 0 | US | H01L | H01L0023 | 30 | 10 | 5 | 3 | 1.67 |
| 4543 | 0 | TW | C30B | C30B0001 | 1 | 5 | 0 | 1 | 0.00 |
| 4544 | 0 | JP | H01L | H01L0033 | 11 | 21 | 0 | 1 | 0.00 |
| 4545 | 0 | AU | H01L | H01L0021 | 7 | 32 | 0 | 2 | 0.00 |
| 4546 | 0 | US | H01L | H01L0023 | 12 | 54 | 1 | 9 | 0.11 |
| 4547 | 0 | TW | H01L | H01L0033 | 10 | 4 | 2 | 1.25 | 0.40 |
| 4548 | 0 | JP | H01L | H01L0021 | 3 | 24 | 1 | 1.5 | 0.33 |
| 4549 | 0 | JP | C30B | C30B0025 | 18 | 20 | 1 | 2.75 | 0.09 |
| 4550 | 0 | DE | H01L | H01L0033 | 1 | 14 | 0 | 1.166666667 | 0.00 |
| 4551 | 0 | KR | H01L | H01L0033 | 3 | 5 | 1 | 1 | 0.50 |
| 4552 | 0 | US | H01L | H01L0021 | 14 | 21 | 8 | 1 | 1.14 |
| 4553 | 0 | JP | H01L | H01L0021 | 15 | 12 | 1 | 1 | 0.33 |
| 4554 | 0 | DE | H01L | H01L0021 | 17 | 21 | 6 | 1.666666667 | 0.60 |
| 4555 | 0 | JP | H01L | H01L0033 | 51 | 22 | 2 | 3.5 | 0.29 |
| 4556 | 0 | CA | H01L | H01L0033 | 23 | 11 | 6 | 2 | 1.00 |
| 4557 | 0 | JP | H01L | H01L0033 | 56 | 33 | 12 | 3 | 2.00 |
| 4558 | 0 | DE | H01L | H01L0033 | 6 | 19 | 0 | 1 | 0.00 |
| 4559 | 0 | US | H01L | H01L0033 | 24 | 4 | 10 | 7 | 1.43 |
| 4560 | 0 | NL | H01L | H01L0021 | 12 | 5 | 2 | 1 | 0.40 |
| 4561 | 0 | JP | H01L | H01L0031 | 24 | 50 | 1 | 1.5 | 0.33 |
| 4562 | 0 | JP | H01L | H01L0033 | 23 | 18 | 3 | 1.4 | 0.43 |
| 4563 | 0 | JP | H01L | H01L0021 | 26 | 15 | 1 | 1 | 0.33 |
| 4564 | 0 | US | H01L | H01L0021 | 38 | 146 | 7 | 18.83333333 | 0.06 |
| 4565 | 0 | JP | H01J | H01J0001 | 16 | 9 | 11 | 1 | 3.67 |
| 4566 | 0 | JP | H01J | H01J0001 | 14 | 9 | 6 | 1 | 6.00 |
| 4567 | 0 | US | H01L | H01L0021 | 17 | 168 | 24 | 4.333333333 | 1.85 |
| 4568 | 0 | JP | H01L | H01L0021 | 51 | 97 | 0 | 3.4 | 0.00 |
| 4569 | 0 | US | H01L | H01L0021 | 32 | 7 | 5 | 2 | 2.50 |
| 4570 | 0 | KR | H01L | H01L0021 | 11 | 6 | 1 | 1 | 0.33 |
| 4571 | 0 | JP | H01L | H01L0021 | 3 | 1 | 2 | 1.5 | 0.22 |
| 4572 | 0 | TW | H01L | H01L0021 | 15 | 7 | 0 | 1 | 0.00 |
| 4573 | 0 | JP | H01L | H01L0021 | 24 | 35 | 4 | 1 | 2.00 |
| 4574 | 0 | JP | H01L | H01L0029 | 31 | 24 | 12 | 1.333333333 | 3.00 |
| 4575 | 0 | JP | H01L | H01L0029 | 20 | 12 | 2 | 1 | 0.67 |
| 4576 | 0 | TW | H01L | H01L0027 | 18 | 4 | 4 | 2 | 1.00 |
| 4577 | 0 | US | G01T | G01T0001 | 26 | 30 | 22 | 1.2 | 3.67 |
| 4578 | 0 | US | H01L | H01L0033 | 13 | 32 | 4 | 1 | 2.00 |
| 4579 | 0 | FR | G02B | G02B0006 | 24 | 10 | 3 | 1 | 0.38 |
| 4580 | 0 | US | H01L | H01L0023 | 74 | 11 | 1 | 6 | 0.17 |
| 4581 | 0 | CA | G02B | G02B0005 | 53 | 71 | 29 | 1.25 | 5.80 |
| 4582 | 0 | JP | H01L | H01L0021 | 1 | 34 | 2 | 3 | 0.33 |
| 4583 | 0 | US | F21V | F21V0007 | 9 | 3 | 27 | 1 | 4.50 |
| 4584 | 0 | JP | H01L | H01L0021 | 11 | 7 | 1 | 1.5 | 0.33 |
| 4585 | 0 | JP | H01L | H01L0021 | 23 | 12 | 5 | 1 | 1.25 |
| 4586 | 0 | JP | H01L | H01L0033 | 37 | 5 | 2 | 1 | 1.00 |
| 4587 | 0 | US | H01L | H01L0021 | 5 | 46 | 0 | 9 | 0.00 |
| 4588 | 0 | JP | C30B | C30B0025 | 12 | 14 | 11 | 2 | 2.75 |
| 4589 | 0 | DE | H01L | H01L0021 | 16 | 28 | 3 | 2.285714286 | 0.19 |
| 4590 | 0 | US | H01L | H01L0021 | 76 | 22 | 15 | 1.222222222 | 1.36 |
| 4591 | 0 | JP | H01L | H01L0027 | 58 | 8 | 3 | 2 | 0.75 |
| 4592 | 0 | JP | H01J | H01J0001 | 20 | 4 | 15 | 1.333333333 | 3.75 |
| 4593 | 0 | JP | G09G | G09G0003 | 30 | 38 | 9 | 1.2 | 1.50 |
| 4594 | 0 | JP | H01S | H01S0005 | 7 | 873 | 8 | 5.5 | 0.73 |
| 4595 | 0 | US | G02B | G02B0006 | 7 | 15 | 1 | 1 | 1.00 |
| 4596 | 0 | TW | H01L | H01L0021 | 13 | 6 | 0 | 1 | 0.00 |
| 4597 | 0 | US | H01L | H01L0021 | 16 | 10 | 8 | 1.5 | 2.67 |
| 4598 | 0 | NL | G01J | G01J0001 | 20 | 8 | 6 | 1.142857143 | 0.75 |
| 4599 | 0 | JP | H01L | H01L0033 | 1 | 2 | 0 | 2 | 0.00 |
| 4600 | 0 | JP | H01L | H01L0021 | 20 | 10 | 4 | 1 | 1.33 |
| 4601 | 0 | DE | H05B | H05B0033 | 22 | 29 | 12 | 3 | 0.24 |
| 4602 | 0 | JP | H01J | H01J0001 | 18 | 5 | 2 | 1.5 | 0.67 |
| 4603 | 0 | US | G02B | G02B0006 | 28 | 86 | 4 | 1 | 4.00 |
| 4604 | 0 | JP | H01L | H01L0021 | 3 | 42 | 0 | 3.75 | 0.00 |
| 4605 | 0 | US | C23C | C23C0014 | 34 | 9 | 4 | 2 | 2.00 |
| 4606 | 0 | US | C23C | C23C0014 | 19 | 17 | 1 | 2 | 0.50 |
| 4607 | 0 | US | C23C | C23C0014 | 47 | 162 | 64 | 3 | 21.33 |
| 4608 | 0 | JP | H01L | H01L0027 | 10 | 4 | 0 | 1 | 0.00 |
| 4609 | 0 | JP | H01L | H01L0033 | 1 | 2 | 0 | 2 | 0.00 |
| 4610 | 0 | DE | H01J | H01J0001 | 3 | 12 | 12 | 2.375 | 0.63 |
| 4611 | 0 | US | C23C | C23C0016 | 18 | 11 | 2 | 3 | 0.67 |
| 4612 | 0 | TW | H01L | H01L0029 | 21 | 13 | 8 | 1 | 2.67 |
| 4613 | 0 | US | H01L | H01L0021 | 38 | 15 | 40 | 2 | 10.00 |
| 4614 | 0 | JP | H01L | H01L0033 | 14 | 36 | 2 | 2.333333333 | 0.29 |
| 4615 | 0 | JP | H01L | H01L0029 | 7 | 8 | 0 | 1 | 0.00 |
| 4616 | 0 | JP | H01L | H01L0021 | 16 | 134 | 1 | 2 | 0.25 |
| 4617 | 0 | US | H01L | H01L0021 | 23 | 45 | 5 | 5 | 1.00 |
| 4618 | 0 | US | F21V | F21V0033 | 33 | 5 | 4 | 2 | 0.50 |
| 4619 | 0 | JP | F21V | F21V0009 | 14 | 24 | 19 | 2 | 4.75 |
| 4620 | 0 | US | H01L | H01L0021 | 5 | 33 | 2 | 2 | 1.00 |
| 4621 | 0 | JP | F27B | F27B0005 | 17 | 14 | 2 | 1.75 | 0.29 |
| 4622 | 0 | US | H01L | H01L0033 | 4 | 59 | 16 | 1 | 2.00 |
| 4623 | 0 | CA | H01L | H01L0033 | 18 | 73 | 21 | 1.666666667 | 4.20 |
| 4624 | 0 | JP | H01L | H01L0033 | 23 | 14 | 3 | 1.2 | 0.50 |
| 4625 | 0 | US | G02B | G02B0006 | 31 | 1 | 1 | 1 | 1.00 |
| 4626 | 0 | JP | H05B | H05B0033 | 11 | 3 | 1 | 1.5 | 0.33 |
| 4627 | 0 | JP | H01L | H01L0029 | 9 | 10 | 2 | 1.333333333 | 0.50 |
| 4628 | 0 | TW | H01L | H01L0021 | 10 | 4 | 8 | 1 | 8.00 |
| 4629 | 0 | JP | H01L | H01L0029 | 18 | 8 | 6 | 1 | 3.00 |
| 4630 | 0 | US | H01L | H01L0021 | 25 | 35 | 5 | 2.5 | 0.33 |
| 4631 | 0 | JP | H01L | H01L0029 | 22 | 12 | 1 | 1 | 0.50 |
| 4632 | 0 | US | C23C | C23C0016 | 27 | 81 | 22 | 2.75 | 2.00 |
| 4633 | 0 | JP | B23K | B23K0031 | 11 | 7 | 0 | 1 | 0.00 |
| 4634 | 0 | US | H01S | H01S0005 | 81 | 59 | 3 | 14.25 | 0.03 |
| 4635 | 0 | US | H01L | H01L0021 | 53 | 105 | 19 | 2 | 9.50 |
| 4636 | 0 | JP | B32B | B32B0009 | 8 | 28 | 0 | 1 | 0.00 |
| 4637 | 0 | TW | H01L | H01L0021 | 13 | 1 | 1 | 1 | 1.00 |
| 4638 | 0 | KR | H01L | H01L0021 | 6 | 6 | 0 | 1.333333333 | 0.00 |
| 4639 | 0 | JP | H01L | H01L0021 | 7 | 12 | 4 | 1.25 | 0.80 |
| 4640 | 0 | JP | H01L | H01L0021 | 19 | 4 | 4 | 1 | 2.00 |
| 4641 | 0 | JP | H01L | H01L0029 | 33 | 26 | 4 | 2.5 | 0.80 |
| 4642 | 0 | JP | H01L | H01L0033 | 20 | 9 | 14 | 2 | 2.33 |
| 4643 | 0 | US | B23K | B23K0035 | 23 | 39 | 1 | 2 | 0.50 |
| 4644 | 0 | JP | H01L | H01L0029 | 15 | 8 | 0 | 1 | 0.00 |
| 4645 | 0 | JP | H01L | H01L0021 | 15 | 6 | 0 | 1.333333333 | 0.00 |
| 4646 | 0 | JP | H01L | H01L0021 | 5 | 8 | 0 | 1.5 | 0.00 |
| 4647 | 0 | DE | H01L | H01L0051 | 25 | 4 | 0 | 1 | 0.00 |
| 4648 | 0 | US | H01L | H01L0033 | 18 | 57 | 10 | 1 | 2.50 |
| 4649 | 0 | TW | C09K | C09K0011 | 17 | 3 | 5 | 1 | 2.50 |
| 4650 | 0 | DE | H01L | H01L0021 | 21 | 33 | 3 | 1.142857143 | 0.38 |
| 4651 | 0 | US | H01L | H01L0033 | 75 | 117 | 7 | 8.8 | 0.08 |
| 4652 | 0 | US | H01L | H01L0029 | 19 | 11 | 3 | 1 | 3.00 |
| 4653 | 1 | JP | H01J | H01J0001 | 7 | 180 | 21 | 5 | 0.23 |
| 4654 | 0 | JP | F21V | F21V0007 | 30 | 18 | 2 | 1 | 1.00 |
| 4655 | 0 | US | H01L | H01L0051 | 42 | 22 | 9 | 1 | 9.00 |
| 4656 | 0 | US | H01L | H01L0021 | 9 | 91 | 26 | 1.2 | 4.33 |
| 4657 | 0 | JP | H01L | H01L0021 | 13 | 16 | 4 | 1 | 1.00 |
| 4658 | 0 | JP | H01L | H01L0033 | 29 | 7 | 0 | 1.5 | 0.00 |
| 4659 | 0 | TW | H01L | H01L0033 | 24 | 6 | 12 | 1 | 6.00 |
| 4660 | 0 | JP | C23C | C23C0016 | 29 | 25 | 6 | 1.5 | 2.00 |
| 4661 | 0 | US | H01L | H01L0021 | 84 | 24 | 9 | 4 | 2.25 |
| 4662 | 0 | US | C30B | C30B0025 | 7 | 9 | 2 | 1 | 2.00 |
| 4663 | 0 | JP | H01L | H01L0021 | 12 | 6 | 12 | 2 | 3.00 |
| 4664 | 0 | US | H01L | H01L0023 | 24 | 28 | 3 | 3 | 1.00 |
| 4665 | 0 | TW | C23C | C23C0016 | 11 | 3 | 0 | 1 | 0.00 |
| 4666 | 0 | US | H01L | H01L0023 | 19 | 22 | 0 | 6 | 0.00 |
| 4667 | 0 | JP | H01L | H01L0021 | 11 | 12 | 2 | 1 | 0.67 |
| 4668 | 0 | JP | H01L | H01L0033 | 7 | 6 | 6 | 1 | 1.20 |
| 4669 | 0 | JP | H01L | H01L0033 | 7 | 6 | 1 | 1 | 0.50 |
| 4670 | 0 | JP | H01J | H01J0001 | 30 | 17 | 1 | 3.5 | 0.14 |
| 4671 | 0 | JP | G02B | G02B0006 | 16 | 3 | 0 | 1 | 0.00 |
| 4672 | 0 | JP | H01J | H01J0009 | 36 | 37 | 7 | 1.166666667 | 1.00 |
| 4673 | 0 | JP | H01L | H01L0021 | 12 | 12 | 10 | 1.2 | 1.67 |
| 4674 | 0 | TW | H01L | H01L0033 | 7 | 12 | 5 | 1 | 2.50 |
| 4675 | 0 | US | H01L | H01L0021 | 20 | 127 | 13 | 8.8 | 0.15 |
| 4676 | 0 | JP | H01L | H01L0029 | 10 | 3 | 1 | 1.666666667 | 0.20 |
| 4677 | 0 | JP | H01L | H01L0027 | 16 | 5 | 4 | 1 | 1.00 |
| 4678 | 0 | TW | H01L | H01L0023 | 20 | 2 | 1 | 1 | 0.50 |
| 4679 | 0 | CA | H01J | H01J0001 | 22 | 6 | 9 | 1.2 | 1.50 |
| 4680 | 0 | JP | H01L | H01L0051 | 32 | 18 | 2 | 2 | 0.50 |
| 4681 | 0 | US | H01L | H01L0021 | 19 | 27 | 3 | 1 | 0.43 |
| 4682 | 0 | JP | G02B | G02B0006 | 10 | 14 | 0 | 1.333333333 | 0.00 |
| 4683 | 0 | US | B22F | B22F0009 | 22 | 8 | 0 | 1 | 0.00 |
| 4684 | 0 | JP | H01L | H01L0021 | 19 | 7 | 5 | 1.5 | 1.67 |
| 4685 | 0 | JP | H01L | H01L0021 | 35 | 8 | 0 | 2.5 | 0.00 |
| 4686 | 0 | US | C08G | C08G0059 | 27 | 9 | 1 | 3 | 0.33 |
| 4687 | 0 | DE | H01L | H01L0027 | 16 | 9 | 1 | 1 | 0.33 |
| 4688 | 0 | TW | H01L | H01L0033 | 9 | 4 | 0 | 2 | 0.00 |
| 4689 | 0 | TW | H01L | H01L0029 | 10 | 2 | 1 | 1 | 1.00 |
| 4690 | 0 | KR | H01L | H01L0029 | 6 | 7 | 0 | 1.5 | 0.00 |
| 4691 | 0 | JP | H01L | H01L0021 | 4 | 22 | 0 | 1 | 0.00 |
| 4692 | 0 | JP | H01L | H01L0021 | 24 | 5 | 2 | 1 | 0.29 |
| 4693 | 0 | JP | H01J | H01J0001 | 20 | 9 | 6 | 1 | 3.00 |
| 4694 | 0 | TW | H01L | H01L0021 | 20 | 13 | 20 | 4.25 | 1.18 |
| 4695 | 0 | US | H01L | H01L0033 | 26 | 11 | 20 | 1 | 20.00 |
| 4696 | 0 | JP | H01L | H01L0029 | 9 | 61 | 2 | 3 | 0.33 |
| 4697 | 0 | US | H01L | H01L0023 | 19 | 3 | 6 | 1 | 6.00 |
| 4698 | 0 | KR | C09K | C09K0011 | 20 | 7 | 5 | 1 | 0.83 |
| 4699 | 0 | JP | G03B | G03B0027 | 3 | 20 | 2 | 1.5 | 0.67 |
| 4700 | 0 | JP | G03B | G03B0027 | 8 | 18 | 1 | 1.6 | 0.13 |
| 4701 | 0 | TW | H01L | H01L0029 | 8 | 9 | 5 | 1 | 2.50 |
| 4702 | 0 | TW | H01L | H01L0027 | 5 | 2 | 9 | 1 | 9.00 |
| 4703 | 0 | SG | H05B | H05B0033 | 13 | 22 | 7 | 2 | 1.75 |
| 4704 | 0 | JP | H01J | H01J0001 | 22 | 4 | 2 | 1 | 0.67 |
| 4705 | 0 | US | C09G | C09G0001 | 32 | 13 | 8 | 1 | 2.67 |
| 4706 | 0 | KR | H01L | H01L0021 | 9 | 11 | 6 | 1.166666667 | 0.86 |
| 4707 | 0 | KR | H01L | H01L0029 | 7 | 7 | 0 | 1 | 0.00 |
| 4708 | 0 | TW | H01L | H01L0033 | 16 | 7 | 0 | 1 | 0.00 |
| 4709 | 0 | JP | H01L | H01L0021 | 16 | 18 | 3 | 1.666666667 | 0.60 |
| 4710 | 0 | JP | H01L | H01L0029 | 2 | 27 | 0 | 4.333333333 | 0.00 |
| 4711 | 0 | KR | H01L | H01L0029 | 20 | 12 | 3 | 1 | 0.60 |
| 4712 | 0 | NL | H01L | H01L0029 | 11 | 7 | 15 | 1 | 2.14 |
| 4713 | 0 | US | H01L | H01L0033 | 26 | 20 | 0 | 1.333333333 | 0.00 |
| 4714 | 0 | TW | H01L | H01L0029 | 6 | 2 | 5 | 2 | 2.50 |
| 4715 | 0 | JP | H01L | H01L0023 | 16 | 6 | 3 | 1 | 1.50 |
| 4716 | 0 | JP | F21V | F21V0008 | 6 | 12 | 9 | 1.5 | 1.00 |
| 4717 | 0 | US | C23C | C23C0016 | 21 | 20 | 8 | 1 | 2.67 |
| 4718 | 0 | DE | H05B | H05B0033 | 23 | 16 | 5 | 3 | 0.10 |
| 4719 | 0 | KR | H01L | H01L0021 | 18 | 10 | 1 | 1 | 0.50 |
| 4720 | 0 | TW | H01L | H01L0027 | 6 | 27 | 9 | 1 | 2.25 |
| 4721 | 0 | JP | H01L | H01L0031 | 10 | 32 | 7 | 2 | 1.75 |
| 4722 | 0 | DE | H01L | H01L0027 | 14 | 6 | 2 | 1 | 1.00 |
| 4723 | 0 | TW | H01L | H01L0021 | 8 | 10 | 3 | 2 | 1.50 |
| 4724 | 0 | DE | C09K | C09K0011 | 7 | 20 | 14 | 1.571428571 | 1.27 |
| 4725 | 0 | US | H01L | H01L0021 | 26 | 38 | 1 | 30.25 | 0.00 |
| 4726 | 0 | JP | H01L | H01L0021 | 6 | 54 | 0 | 1.5 | 0.00 |
| 4727 | 0 | TW | H01L | H01L0021 | 5 | 10 | 4 | 2 | 2.00 |
| 4728 | 0 | KR | H01L | H01L0021 | 3 | 4 | 0 | 1.333333333 | 0.00 |
| 4729 | 0 | TW | H01L | H01L0033 | 4 | 4 | 1 | 1 | 0.50 |
| 4730 | 0 | KR | H01L | H01L0033 | 36 | 28 | 5 | 2 | 1.25 |
| 4731 | 0 | JP | G03B | G03B0027 | 8 | 9 | 2 | 1 | 1.00 |
| 4732 | 0 | JP | B32B | B32B0037 | 42 | 12 | 6 | 1.8 | 0.67 |
| 4733 | 0 | JP | H01L | H01L0021 | 10 | 17 | 3 | 5.5 | 0.27 |
| 4734 | 0 | JP | H01L | H01L0033 | 22 | 7 | 4 | 1 | 2.00 |
| 4735 | 0 | US | C30B | C30B0023 | 7 | 49 | 2 | 1.25 | 0.40 |
| 4736 | 0 | JP | H05B | H05B0033 | 15 | 173 | 20 | 5 | 0.22 |
| 4737 | 0 | JP | G02B | G02B0005 | 12 | 25 | 4 | 1.333333333 | 1.00 |
| 4738 | 0 | JP | B60Q | B60Q0001 | 20 | 16 | 2 | 1 | 0.33 |
| 4739 | 0 | US | H01L | H01L0021 | 27 | 26 | 1 | 1 | 0.33 |
| 4740 | 0 | US | H01L | H01L0021 | 31 | 14 | 5 | 6 | 0.83 |
| 4741 | 0 | US | G02F | G02F0001 | 45 | 19 | 62 | 2 | 31.00 |
| 4742 | 0 | US | G02B | G02B0006 | 66 | 20 | 3 | 1 | 0.50 |
| 4743 | 0 | US | C30B | C30B0025 | 7 | 20 | 0 | 1 | 0.00 |
| 4744 | 0 | JP | H01L | H01L0021 | 12 | 6 | 5 | 1.166666667 | 0.71 |
| 4745 | 0 | KR | H01L | H01L0021 | 8 | 7 | 0 | 1 | 0.00 |
| 4746 | 0 | US | H01L | H01L0033 | 33 | 3 | 7 | 4 | 1.75 |
| 4747 | 0 | KR | H01L | H01L0021 | 25 | 6 | 0 | 1 | 0.00 |
| 4748 | 0 | JP | H01L | H01L0023 | 19 | 10 | 3 | 1 | 1.50 |
| 4749 | 0 | JP | H01L | H01L0021 | 6 | 14 | 4 | 1.285714286 | 0.44 |
| 4750 | 0 | MY | C09K | C09K0011 | 16 | 18 | 5 | 1 | 1.67 |
| 4751 | 0 | MY | H05B | H05B0033 | 10 | 1 | 0 | 1 | 0.00 |
| 4752 | 0 | US | H01L | H01L0031 | 48 | 39 | 24 | 1 | 12.00 |
| 4753 | 0 | JP | H01L | H01L0033 | 2 | 3 | 0 | 1.5 | 0.00 |
| 4754 | 0 | DE | H01L | H01L0021 | 5 | 16 | 12 | 1.428571429 | 1.20 |
| 4755 | 0 | US | H01L | H01L0021 | 5 | 17 | 8 | 1.25 | 0.80 |
| 4756 | 0 | US | C23C | C23C0016 | 26 | 10 | 3 | 4 | 0.75 |
| 4757 | 0 | DE | H01L | H01L0035 | 24 | 2 | 3 | 1 | 3.00 |
| 4758 | 0 | US | F21S | F21S0008 | 38 | 13 | 24 | 1 | 4.00 |
| 4759 | 1 | DE | C09K | C09K0011 | 33 | 278 | 27 | 11.77777778 | 0.25 |
| 4760 | 0 | JP | H01L | H01L0029 | 19 | 8 | 3 | 1.666666667 | 0.60 |
| 4761 | 0 | JP | H01L | H01L0029 | 24 | 14 | 10 | 1.5 | 3.33 |
| 4762 | 0 | US | F21V | F21V0007 | 30 | 17 | 29 | 1 | 9.67 |
| 4763 | 0 | US | H01L | H01L0021 | 26 | 45 | 1 | 30.25 | 0.00 |
| 4764 | 0 | US | H01L | H01L0051 | 54 | 10 | 22 | 1 | 4.40 |
| 4765 | 0 | US | C30B | C30B0023 | 17 | 211 | 1 | 3.333333333 | 0.05 |
| 4766 | 0 | TW | H01L | H01L0029 | 9 | 5 | 17 | 1 | 8.50 |
| 4767 | 0 | TW | F21V | F21V0009 | 8 | 6 | 20 | 1 | 20.00 |
| 4768 | 0 | US | E01F | E01F0009 | 38 | 43 | 12 | 1 | 12.00 |
| 4769 | 0 | JP | G03C | G03C0001 | 5 | 11 | 1 | 1 | 0.50 |
| 4770 | 0 | US | H01L | H01L0021 | 10 | 46 | 1 | 5 | 0.20 |
| 4771 | 0 | JP | H01L | H01L0021 | 8 | 409 | 0 | 1.285714286 | 0.00 |
| 4772 | 0 | JP | H01L | H01L0029 | 26 | 5 | 5 | 1.5 | 1.67 |
| 4773 | 0 | JP | H01L | H01L0029 | 7 | 5 | 0 | 2 | 0.00 |
| 4774 | 0 | JP | H01L | H01L0027 | 5 | 7 | 5 | 1 | 1.67 |
| 4775 | 0 | US | C30B | C30B0025 | 34 | 40 | 3 | 2 | 0.30 |
| 4776 | 0 | JP | H01L | H01L0029 | 20 | 23 | 10 | 1.857142857 | 0.77 |
| 4777 | 0 | US | C30B | C30B0025 | 15 | 200 | 2 | 4.333333333 | 0.15 |
| 4778 | 0 | US | H01L | H01L0027 | 32 | 4 | 26 | 3 | 8.67 |
| 4779 | 0 | JP | H01L | H01L0021 | 4 | 15 | 1 | 1.8 | 0.11 |
| 4780 | 0 | US | H01L | H01L0021 | 71 | 45 | 2 | 30.25 | 0.01 |
| 4781 | 0 | US | H01L | H01L0031 | 36 | 8 | 49 | 1 | 16.33 |
| 4782 | 0 | US | H01L | H01L0021 | 13 | 19 | 14 | 1.166666667 | 2.00 |
| 4783 | 0 | TW | H01S | H01S0005 | 10 | 2 | 1 | 1 | 1.00 |
| 4784 | 0 | TW | H01L | H01L0029 | 6 | 1 | 1 | 1 | 0.50 |
| 4785 | 0 | JP | H01L | H01L0029 | 39 | 8 | 1 | 1 | 0.25 |
| 4786 | 0 | JP | H01L | H01L0029 | 10 | 3 | 3 | 1 | 1.00 |
| 4787 | 0 | JP | H01L | H01L0029 | 19 | 13 | 0 | 1 | 0.00 |
| 4788 | 0 | JP | H01L | H01L0029 | 9 | 14 | 0 | 2 | 0.00 |
| 4789 | 0 | US | H01L | H01L0031 | 13 | 16 | 2 | 1.166666667 | 0.29 |
| 4790 | 0 | US | H01L | H01L0021 | 15 | 17 | 1 | 1 | 1.00 |
| 4791 | 0 | US | C09K | C09K0011 | 13 | 21 | 10 | 1 | 2.00 |
| 4792 | 0 | US | H01L | H01L0033 | 7 | 24 | 46 | 1.666666667 | 4.60 |
| 4793 | 0 | JP | G02B | G02B0006 | 15 | 3 | 0 | 1 | 0.00 |
| 4794 | 0 | KR | H01L | H01L0021 | 22 | 2 | 0 | 1.333333333 | 0.00 |
| 4795 | 0 | JP | H01L | H01L0021 | 8 | 6 | 2 | 1.5 | 0.67 |
| 4796 | 0 | JP | H01L | H01L0023 | 12 | 3 | 1 | 1 | 0.17 |
| 4797 | 0 | US | F21V | F21V0019 | 4 | 3 | 39 | 2 | 3.90 |
| 4798 | 0 | US | H01L | H01L0021 | 20 | 14 | 0 | 1 | 0.00 |
| 4799 | 0 | KR | H01L | H01L0021 | 7 | 3 | 1 | 1.5 | 0.33 |
| 4800 | 0 | JP | H01L | H01L0021 | 3 | 17 | 0 | 1 | 0.00 |
| 4801 | 0 | KR | H01L | H01L0029 | 8 | 10 | 1 | 1.5 | 0.33 |
| 4802 | 0 | US | H01L | H01L0021 | 33 | 13 | 6 | 1.333333333 | 1.50 |
| 4803 | 0 | US | H01L | H01L0021 | 13 | 18 | 3 | 1.666666667 | 0.60 |
| 4804 | 0 | US | H05B | H05B0033 | 6 | 16 | 3 | 2.4 | 0.25 |
| 4805 | 0 | US | G02B | G02B0006 | 20 | 12 | 11 | 1 | 5.50 |
| 4806 | 0 | US | H01L | H01L0021 | 13 | 36 | 5 | 1 | 5.00 |
| 4807 | 0 | JP | H01L | H01L0033 | 7 | 11 | 1 | 1.285714286 | 0.11 |
| 4808 | 0 | BN | F21V | F21V0019 | 11 | 3 | 5 | 1 | 1.67 |
| 4809 | 0 | US | H01L | H01L0021 | 8 | 16 | 4 | 1 | 4.00 |
| 4810 | 0 | TW | H01L | H01L0029 | 35 | 3 | 0 | 1 | 0.00 |
| 4811 | 0 | DE | H01L | H01L0033 | 21 | 29 | 9 | 1.333333333 | 1.13 |
| 4812 | 0 | US | H01L | H01L0021 | 8 | 4 | 1 | 1.25 | 0.20 |
| 4813 | 0 | SG | H05B | H05B0033 | 25 | 16 | 3 | 1 | 0.60 |
| 4814 | 0 | JP | G11B | G11B0007 | 4 | 9 | 2 | 2 | 0.33 |
| 4815 | 0 | JP | F21V | F21V0029 | 15 | 8 | 16 | 1 | 8.00 |
| 4816 | 0 | JP | H01J | H01J0009 | 18 | 26 | 5 | 4.5 | 0.19 |
| 4817 | 0 | DE | H01L | H01L0029 | 42 | 74 | 9 | 1.571428571 | 0.82 |
| 4818 | 0 | DE | C03C | C03C0017 | 25 | 10 | 2 | 1.333333333 | 0.50 |
| 4819 | 0 | US | G01J | G01J0003 | 59 | 59 | 0 | 1.333333333 | 0.00 |
| 4820 | 0 | SG | H01L | H01L0031 | 20 | 5 | 11 | 2.166666667 | 0.85 |
| 4821 | 0 | KR | C23C | C23C0016 | 35 | 3 | 2 | 1 | 0.67 |
| 4822 | 0 | JP | H01L | H01L0033 | 15 | 4 | 7 | 1.25 | 1.40 |
| 4823 | 0 | DE | H01L | H01L0023 | 15 | 111 | 1 | 3 | 0.17 |
| 4824 | 0 | DE | H01L | H01L0023 | 15 | 13 | 15 | 1 | 2.14 |
| 4825 | 0 | DE | H01L | H01L0029 | 10 | 99 | 2 | 3.333333333 | 0.10 |
| 4826 | 0 | DE | H01L | H01L0021 | 38 | 43 | 0 | 1.285714286 | 0.00 |
| 4827 | 0 | JP | H01L | H01L0021 | 41 | 11 | 3 | 1.5 | 1.00 |
| 4828 | 0 | JP | C08F | C08F0297 | 23 | 27 | 6 | 2 | 0.60 |
| 4829 | 0 | TW | H01L | H01L0033 | 12 | 5 | 2 | 1 | 1.00 |
| 4830 | 0 | JP | H01L | H01L0029 | 2 | 12 | 1 | 1 | 0.25 |
| 4831 | 0 | TW | H01L | H01L0029 | 26 | 3 | 3 | 1 | 1.50 |
| 4832 | 0 | US | H01L | H01L0021 | 12 | 3 | 19 | 1 | 19.00 |
| 4833 | 0 | US | F21V | F21V0009 | 16 | 36 | 13 | 1.333333333 | 1.08 |
| 4834 | 0 | JP | G09G | G09G0003 | 15 | 65 | 5 | 3 | 0.83 |
| 4835 | 1 | DE | G01R | G01R0031 | 19 | 23 | 6 | 1.5 | 0.50 |
| 4836 | 0 | JP | G03B | G03B0027 | 9 | 13 | 0 | 1 | 0.00 |
| 4837 | 0 | US | H01L | H01L0033 | 20 | 11 | 8 | 1 | 2.67 |
| 4838 | 0 | JP | F21V | F21V0029 | 28 | 15 | 3 | 1 | 1.00 |
| 4839 | 0 | JP | H05B | H05B0033 | 14 | 47 | 0 | 1.428571429 | 0.00 |
| 4840 | 0 | US | C09K | C09K0011 | 19 | 3 | 7 | 1.166666667 | 1.00 |
| 4841 | 0 | KR | H01L | H01L0021 | 28 | 9 | 2 | 1.5 | 0.67 |
| 4842 | 0 | JP | H01L | H01L0021 | 10 | 13 | 8 | 1.333333333 | 2.00 |
| 4843 | 0 | US | H01L | H01L0029 | 55 | 12 | 7 | 1 | 7.00 |
| 4844 | 0 | DE | H01L | H01L0021 | 31 | 30 | 3 | 2.285714286 | 0.19 |
| 4845 | 0 | JP | H01L | H01L0029 | 9 | 98 | 13 | 2 | 1.63 |
| 4846 | 0 | JP | H01L | H01L0033 | 5 | 12 | 0 | 2 | 0.00 |
| 4847 | 0 | US | G01N | G01N0021 | 32 | 47 | 6 | 3.333333333 | 0.30 |
| 4848 | 0 | JP | H05B | H05B0033 | 16 | 21 | 4 | 1.2 | 0.67 |
| 4849 | 0 | JP | G02B | G02B0005 | 10 | 38 | 5 | 2.5 | 1.00 |
| 4850 | 0 | US | F21K | F21K0099 | 9 | 1 | 7 | 3.444444444 | 0.23 |
| 4851 | 0 | JP | F21V | F21V0005 | 17 | 8 | 27 | 1 | 13.50 |
| 4852 | 0 | US | F21V | F21V0007 | 8 | 2 | 4 | 1 | 0.67 |
| 4853 | 0 | JP | H05B | H05B0033 | 16 | 58 | 34 | 1.666666667 | 6.80 |
| 4854 | 0 | JP | H01L | H01L0029 | 20 | 9 | 0 | 2 | 0.00 |
| 4855 | 0 | US | C09K | C09K0011 | 20 | 39 | 13 | 1 | 6.50 |
| 4856 | 0 | US | C30B | C30B0025 | 28 | 49 | 0 | 30.25 | 0.00 |
| 4857 | 0 | US | H01L | H01L0021 | 20 | 8 | 5 | 1 | 5.00 |
| 4858 | 0 | US | H01L | H01L0029 | 26 | 65 | 2 | 30.25 | 0.01 |
| 4859 | 0 | DE | H05B | H05B0033 | 41 | 19 | 1 | 3 | 0.02 |
| 4860 | 0 | SG | H01L | H01L0021 | 3 | 6 | 0 | 1 | 0.00 |
| 4861 | 0 | US | H01L | H01L0027 | 41 | 7 | 6 | 1 | 2.00 |
| 4862 | 0 | TW | H01L | H01L0027 | 20 | 10 | 0 | 1 | 0.00 |
| 4863 | 0 | TW | H01L | H01L0021 | 13 | 4 | 1 | 1.5 | 0.33 |
| 4864 | 0 | KR | H01L | H01L0023 | 15 | 5 | 5 | 1 | 2.50 |
| 4865 | 0 | JP | H01S | H01S0005 | 16 | 35 | 1 | 3 | 0.17 |
| 4866 | 0 | US | H01L | H01L0033 | 35 | 32 | 23 | 1 | 11.50 |
| 4867 | 0 | US | H01J | H01J0007 | 22 | 23 | 5 | 4 | 1.25 |
| 4868 | 0 | US | C08L | C08L0063 | 6 | 10 | 7 | 3 | 2.33 |
| 4869 | 0 | JP | H01L | H01L0021 | 35 | 10 | 1 | 1 | 0.50 |
| 4870 | 0 | KR | H01L | H01L0021 | 34 | 14 | 0 | 1.2 | 0.00 |
| 4871 | 0 | US | H01L | H01L0021 | 20 | 64 | 0 | 30.25 | 0.00 |
| 4872 | 0 | CH | H01L | H01L0027 | 13 | 7 | 0 | 1 | 0.00 |
| 4873 | 0 | TW | H01L | H01L0023 | 5 | 5 | 21 | 1 | 21.00 |
| 4874 | 0 | US | F21V | F21V0021 | 7 | 6 | 4 | 1 | 0.57 |
| 4875 | 0 | US | H01L | H01L0031 | 20 | 65 | 0 | 30.25 | 0.00 |
| 4876 | 0 | US | H01L | H01L0021 | 17 | 18 | 8 | 2 | 4.00 |
| 4877 | 0 | US | C30B | C30B0009 | 100 | 26 | 6 | 1.375 | 0.55 |
| 4878 | 0 | US | F21V | F21V0029 | 34 | 24 | 10 | 3.333333333 | 0.50 |
| 4879 | 0 | JP | H01L | H01L0021 | 6 | 7 | 6 | 2 | 1.50 |
| 4880 | 0 | JP | H01L | H01L0021 | 29 | 7 | 1 | 1 | 0.50 |
| 4881 | 0 | US | C30B | C30B0025 | 8 | 1 | 1 | 1.5 | 0.33 |
| 4882 | 0 | US | H05B | H05B0033 | 26 | 10 | 0 | 1 | 0.00 |
| 4883 | 0 | JP | H01L | H01L0027 | 4 | 16 | 6 | 1 | 3.00 |
| 4884 | 0 | DE | H05B | H05B0033 | 16 | 19 | 6 | 2 | 0.50 |
| 4885 | 0 | JP | H05B | H05B0033 | 15 | 172 | 17 | 5 | 0.19 |
| 4886 | 0 | JP | F21V | F21V0003 | 12 | 11 | 4 | 1.142857143 | 0.50 |
| 4887 | 0 | DE | C30B | C30B0025 | 20 | 11 | 2 | 1 | 0.29 |
| 4888 | 0 | TW | H01L | H01L0029 | 12 | 8 | 1 | 2 | 0.25 |
| 4889 | 0 | DE | H01L | H01L0033 | 18 | 11 | 0 | 1.666666667 | 0.00 |
| 4890 | 0 | JP | H01L | H01L0023 | 10 | 10 | 3 | 1 | 1.50 |
| 4891 | 0 | US | H05B | H05B0033 | 28 | 15 | 2 | 1 | 2.00 |
| 4892 | 0 | KR | H01L | H01L0029 | 10 | 13 | 9 | 2 | 4.50 |
| 4893 | 0 | TW | H01L | H01L0033 | 26 | 24 | 6 | 1 | 2.00 |
| 4894 | 0 | DE | C09K | C09K0011 | 32 | 29 | 9 | 3 | 0.18 |
| 4895 | 0 | US | H01L | H01L0033 | 23 | 145 | 5 | 18.83333333 | 0.04 |
| 4896 | 0 | JP | C30B | C30B0029 | 12 | 7 | 0 | 1 | 0.00 |
| 4897 | 0 | JP | H01L | H01L0021 | 13 | 30 | 1 | 1 | 0.50 |
| 4898 | 0 | US | H01L | H01L0021 | 19 | 5 | 0 | 1.166666667 | 0.00 |
| 4899 | 0 | JP | B41J | B41J0002 | 12 | 6 | 2 | 1.333333333 | 0.50 |
| 4900 | 0 | DE | H01L | H01L0033 | 22 | 19 | 6 | 1 | 0.86 |
| 4901 | 0 | TW | H01L | H01L0021 | 7 | 10 | 3 | 2 | 0.75 |
| 4902 | 0 | JP | H01L | H01L0021 | 2 | 40 | 0 | 3.75 | 0.00 |
| 4903 | 0 | JP | H01L | H01L0033 | 27 | 7 | 5 | 1 | 2.50 |
| 4904 | 0 | US | C09K | C09K0011 | 19 | 7 | 2 | 2 | 1.00 |
| 4905 | 0 | JP | H05B | H05B0033 | 26 | 18 | 1 | 2 | 0.25 |
| 4906 | 0 | US | H01L | H01L0021 | 57 | 174 | 4 | 4.5 | 0.44 |
| 4907 | 0 | US | H01L | H01L0021 | 18 | 40 | 10 | 2 | 2.50 |
| 4908 | 0 | US | C30B | C30B0009 | 40 | 81 | 20 | 4.428571429 | 0.65 |
| 4909 | 0 | JP | H01L | H01L0021 | 10 | 10 | 0 | 1.5 | 0.00 |
| 4910 | 0 | JP | H01L | H01L0031 | 4 | 9 | 3 | 1.5 | 1.00 |
| 4911 | 0 | DE | H01L | H01L0033 | 12 | 27 | 0 | 1 | 0.00 |
| 4912 | 0 | JP | H01L | H01L0029 | 27 | 10 | 0 | 1.333333333 | 0.00 |
| 4913 | 0 | FR | H01L | H01L0029 | 20 | 21 | 0 | 1.428571429 | 0.00 |
| 4914 | 0 | JP | H01L | H01L0021 | 2 | 7 | 0 | 1 | 0.00 |
| 4915 | 0 | DE | C23C | C23C0016 | 15 | 40 | 2 | 1 | 0.29 |
| 4916 | 0 | US | H01L | H01L0021 | 11 | 6 | 0 | 1 | 0.00 |
| 4917 | 0 | JP | H01L | H01L0029 | 32 | 11 | 1 | 1.75 | 0.14 |
| 4918 | 0 | JP | H01L | H01L0023 | 8 | 152 | 12 | 3.125 | 0.48 |
| 4919 | 0 | JP | H01L | H01L0021 | 14 | 18 | 1 | 1 | 0.33 |
| 4920 | 0 | KR | H01L | H01L0027 | 8 | 20 | 2 | 1.333333333 | 0.50 |
| 4921 | 0 | JP | H01L | H01L0021 | 19 | 4 | 1 | 1.5 | 0.33 |
| 4922 | 0 | TW | H01L | H01L0033 | 9 | 2 | 0 | 2 | 0.00 |
| 4923 | 0 | KR | H01L | H01L0033 | 46 | 21 | 19 | 2.2 | 1.73 |
| 4924 | 0 | JP | H01L | H01L0029 | 20 | 15 | 1 | 1 | 0.25 |
| 4925 | 0 | JP | H01L | H01L0023 | 11 | 54 | 1 | 1 | 0.17 |
| 4926 | 0 | KR | H01L | H01L0021 | 20 | 8 | 1 | 1 | 0.25 |
| 4927 | 0 | TW | H01L | H01L0027 | 26 | 2 | 4 | 1 | 2.00 |
| 4928 | 1 | DE | C09K | C09K0011 | 36 | 404 | 5 | 11.77777778 | 0.05 |
| 4929 | 0 | TW | H01L | H01L0029 | 14 | 4 | 1 | 2 | 0.50 |
| 4930 | 0 | US | C23C | C23C0016 | 57 | 239 | 27 | 1.25 | 5.40 |
| 4931 | 0 | KR | F21V | F21V0003 | 20 | 6 | 38 | 1 | 19.00 |
| 4932 | 0 | US | H01L | H01L0021 | 45 | 386 | 58 | 3 | 19.33 |
| 4933 | 0 | TW | H01L | H01L0021 | 28 | 2 | 0 | 1 | 0.00 |
| 4934 | 0 | JP | H01L | H01L0021 | 1 | 11 | 2 | 2.5 | 0.40 |
| 4935 | 0 | US | F21L | F21L0004 | 21 | 8 | 9 | 1.166666667 | 1.29 |
| 4936 | 0 | TW | H01L | H01L0029 | 21 | 32 | 7 | 1 | 2.33 |
| 4937 | 0 | JP | H01L | H01L0029 | 20 | 4 | 0 | 1 | 0.00 |
| 4938 | 0 | JP | H01L | H01L0029 | 3 | 21 | 4 | 1.333333333 | 1.00 |
| 4939 | 0 | JP | H01L | H01L0033 | 17 | 5 | 0 | 2 | 0.00 |
| 4940 | 0 | TW | H01L | H01L0023 | 20 | 8 | 5 | 1 | 1.25 |
| 4941 | 0 | TW | H01L | H01L0029 | 10 | 9 | 0 | 2 | 0.00 |
| 4942 | 0 | KR | F21V | F21V0021 | 5 | 6 | 5 | 1 | 2.50 |
| 4943 | 0 | US | H01L | H01L0029 | 28 | 49 | 11 | 4 | 0.39 |
| 4944 | 0 | US | H01J | H01J0001 | 46 | 144 | 29 | 18.83333333 | 0.26 |
| 4945 | 0 | US | H01J | H01J0001 | 8 | 17 | 1 | 1 | 0.20 |
| 4946 | 0 | AU | H01L | H01L0021 | 12 | 19 | 0 | 2 | 0.00 |
| 4947 | 0 | TW | H01L | H01L0027 | 7 | 6 | 0 | 1 | 0.00 |
| 4948 | 0 | JP | H01L | H01L0029 | 21 | 16 | 7 | 2.333333333 | 1.00 |
| 4949 | 0 | JP | H01L | H01L0033 | 18 | 5 | 4 | 3 | 0.44 |
| 4950 | 0 | TW | H01L | H01L0027 | 17 | 3 | 11 | 1 | 5.50 |
| 4951 | 0 | JP | H01L | H01L0031 | 10 | 26 | 3 | 1 | 1.00 |
| 4952 | 0 | JP | C09K | C09K0011 | 12 | 13 | 0 | 1 | 0.00 |
| 4953 | 0 | US | H01L | H01L0029 | 65 | 139 | 22 | 18.83333333 | 0.19 |
| 4954 | 0 | DE | H01L | H01L0033 | 25 | 13 | 0 | 1.25 | 0.00 |
| 4955 | 0 | KR | H01L | H01L0029 | 14 | 14 | 1 | 1 | 0.33 |
| 4956 | 0 | JP | H01L | H01L0029 | 18 | 12 | 0 | 1 | 0.00 |
| 4957 | 0 | TW | H01L | H01L0027 | 9 | 4 | 3 | 2 | 1.50 |
| 4958 | 0 | US | F21V | F21V0001 | 21 | 8 | 0 | 1 | 0.00 |
| 4959 | 0 | JP | H01L | H01L0023 | 4 | 6 | 1 | 1.25 | 0.20 |
| 4960 | 0 | JP | F21S | F21S0008 | 19 | 6 | 2 | 1 | 0.29 |
| 4961 | 0 | US | F21V | F21V0021 | 33 | 100 | 25 | 4.333333333 | 1.92 |
| 4962 | 0 | JP | H01L | H01L0051 | 14 | 26 | 6 | 1.4 | 0.86 |
| 4963 | 0 | KR | H01L | H01L0035 | 12 | 39 | 1 | 1 | 0.20 |
| 4964 | 0 | US | H01L | H01L0029 | 112 | 41 | 7 | 1 | 1.00 |
| 4965 | 0 | US | H01L | H01L0021 | 22 | 67 | 1 | 14.25 | 0.01 |
| 4966 | 0 | US | H01L | H01L0029 | 35 | 28 | 12 | 2 | 3.00 |
| 4967 | 0 | US | H01L | H01L0021 | 25 | 58 | 39 | 3 | 13.00 |
| 4968 | 0 | JP | H01L | H01L0021 | 4 | 18 | 2 | 1 | 1.00 |
| 4969 | 0 | JP | H01L | H01L0029 | 3 | 15 | 1 | 1.5 | 0.33 |
| 4970 | 0 | JP | G02B | G02B0006 | 36 | 21 | 6 | 1 | 2.00 |
| 4971 | 0 | US | H01L | H01L0021 | 16 | 11 | 1 | 6 | 0.17 |
| 4972 | 0 | US | H01J | H01J0001 | 24 | 99 | 2 | 1 | 2.00 |
| 4973 | 0 | US | B41J | B41J0002 | 22 | 7 | 0 | 1 | 0.00 |
| 4974 | 0 | US | B32B | B32B0009 | 33 | 176 | 2 | 4.5 | 0.22 |
| 4975 | 0 | JP | H01L | H01L0029 | 7 | 15 | 2 | 1 | 1.00 |
| 4976 | 0 | US | C09K | C09K0019 | 91 | 156 | 0 | 1.142857143 | 0.00 |
| 4977 | 0 | KR | H01L | H01L0029 | 7 | 9 | 12 | 1 | 4.00 |
| 4978 | 0 | JP | H01L | H01L0023 | 21 | 5 | 4 | 1 | 1.33 |
| 4979 | 0 | JP | H01L | H01L0021 | 58 | 17 | 2 | 1 | 1.00 |
| 4980 | 0 | JP | H01L | H01L0029 | 13 | 60 | 2 | 1.5 | 0.67 |
| 4981 | 0 | JP | C30B | C30B0025 | 14 | 137 | 1 | 4.5 | 0.11 |
| 4982 | 0 | KR | H01L | H01L0029 | 16 | 7 | 0 | 1.25 | 0.00 |
| 4983 | 0 | US | H01L | H01L0027 | 30 | 33 | 10 | 4 | 0.36 |
| 4984 | 0 | KR | H01L | H01L0029 | 13 | 6 | 0 | 1 | 0.00 |
| 4985 | 0 | KR | H01J | H01J0001 | 25 | 16 | 0 | 1 | 0.00 |
| 4986 | 0 | JP | B29C | B29C0045 | 6 | 28 | 0 | 1.333333333 | 0.00 |
| 4987 | 0 | US | H01L | H01L0021 | 2 | 8 | 0 | 1.333333333 | 0.00 |
| 4988 | 0 | JP | H01L | H01L0021 | 42 | 77 | 4 | 1.25 | 0.80 |
| 4989 | 0 | TW | H01L | H01L0027 | 4 | 2 | 1 | 1 | 0.50 |
| 4990 | 0 | TW | H01L | H01L0027 | 4 | 3 | 1 | 2 | 0.50 |
| 4991 | 0 | JP | C09K | C09K0011 | 11 | 23 | 3 | 1 | 0.50 |
| 4992 | 0 | MI | H05K | H05K0007 | 10 | 9 | 7 | 1.5 | 2.33 |
| 4993 | 0 | JP | C30B | C30B0025 | 2 | 17 | 2 | 1.5 | 0.67 |
| 4994 | 0 | TW | H01L | H01L0029 | 11 | 1 | 2 | 1.25 | 0.40 |
| 4995 | 0 | JP | H01L | H01L0021 | 25 | 17 | 0 | 1 | 0.00 |
| 4996 | 0 | DE | H01L | H01L0023 | 14 | 101 | 7 | 3.333333333 | 0.35 |
| 4997 | 0 | DE | C08L | C08L0063 | 25 | 19 | 4 | 1.142857143 | 0.50 |
| 4998 | 0 | DE | H01J | H01J0001 | 8 | 11 | 1 | 2.375 | 0.05 |
| 4999 | 0 | SG | G02F | G02F0001 | 16 | 4 | 3 | 1 | 3.00 |
| 5000 | 0 | US | H01L | H01L0029 | 16 | 125 | 1 | 4 | 0.25 |
| 5001 | 0 | US | H01L | H01L0027 | 20 | 26 | 10 | 4 | 0.36 |
| 5002 | 0 | JP | C30B | C30B0029 | 13 | 3 | 1 | 1 | 0.50 |
| 5003 | 0 | KR | H01L | H01L0033 | 8 | 11 | 5 | 1.333333333 | 1.25 |
| 5004 | 0 | JP | H01L | H01L0031 | 10 | 6 | 3 | 1.5 | 1.00 |
| 5005 | 0 | JP | H01L | H01L0021 | 5 | 14 | 2 | 1.5 | 0.67 |
| 5006 | 0 | US | C30B | C30B0009 | 18 | 58 | 6 | 4.428571429 | 0.19 |
| 5007 | 0 | US | H01L | H01L0029 | 20 | 144 | 6 | 18.83333333 | 0.05 |
| 5008 | 0 | US | G01N | G01N0033 | 22 | 41 | 3 | 3.666666667 | 0.14 |
| 5009 | 0 | JP | C30B | C30B0029 | 33 | 19 | 6 | 1.25 | 1.20 |
| 5010 | 0 | US | H01L | H01L0031 | 32 | 142 | 3 | 18.83333333 | 0.03 |
| 5011 | 0 | JP | H01L | H01L0029 | 6 | 9 | 1 | 1.333333333 | 0.25 |
| 5012 | 0 | KR | H01L | H01L0021 | 7 | 4 | 2 | 1.333333333 | 0.50 |
| 5013 | 0 | JP | H01L | H01L0021 | 10 | 3 | 2 | 1 | 0.50 |
| 5014 | 0 | JP | H01L | H01L0033 | 162 | 65 | 3 | 1.428571429 | 0.30 |
| 5015 | 0 | US | H01L | H01L0023 | 16 | 65 | 5 | 1.25 | 1.00 |
| 5016 | 0 | US | G03C | G03C0001 | 38 | 10 | 2 | 1 | 0.29 |
| 5017 | 0 | TW | H01L | H01L0021 | 11 | 1 | 0 | 1.5 | 0.00 |
| 5018 | 0 | US | H01L | H01L0035 | 14 | 33 | 2 | 1 | 1.00 |
| 5019 | 0 | TW | H01L | H01L0021 | 20 | 3 | 4 | 1 | 1.33 |
| 5020 | 0 | DE | H01L | H01L0023 | 40 | 12 | 5 | 1 | 0.71 |
| 5021 | 0 | JP | H01L | H01L0051 | 22 | 50 | 0 | 1.428571429 | 0.00 |
| 5022 | 0 | SG | F21V | F21V0007 | 16 | 4 | 1 | 1 | 1.00 |
| 5023 | 0 | JP | G02B | G02B0006 | 6 | 122 | 0 | 4 | 0.00 |
| 5024 | 0 | JP | G01R | G01R0031 | 13 | 18 | 0 | 1 | 0.00 |
| 5025 | 0 | DE | H01L | H01L0021 | 34 | 74 | 1 | 3.125 | 0.04 |
| 5026 | 0 | DE | H01L | H01L0021 | 11 | 8 | 0 | 1 | 0.00 |
| 5027 | 0 | US | H05B | H05B0033 | 42 | 17 | 0 | 2.4 | 0.00 |
| 5028 | 0 | JP | H01L | H01L0029 | 2 | 18 | 1 | 1 | 0.50 |
| 5029 | 0 | JP | H01L | H01L0029 | 12 | 28 | 0 | 1.25 | 0.00 |
| 5030 | 0 | DE | H01L | H01L0033 | 24 | 22 | 6 | 1 | 0.86 |
| 5031 | 0 | JP | C30B | C30B0025 | 11 | 35 | 4 | 1 | 2.00 |
| 5032 | 0 | JP | H01L | H01L0029 | 18 | 22 | 0 | 1.5 | 0.00 |
| 5033 | 0 | JP | H01L | H01L0027 | 15 | 5 | 0 | 1 | 0.00 |
| 5034 | 0 | US | G02B | G02B0006 | 30 | 39 | 0 | 2.333333333 | 0.00 |
| 5035 | 0 | US | F21L | F21L0004 | 10 | 9 | 2 | 4.444444444 | 0.05 |
| 5036 | 0 | JP | C09K | C09K0011 | 20 | 13 | 1 | 1 | 0.17 |
| 5037 | 0 | US | H01R | H01R0033 | 20 | 50 | 8 | 1 | 1.60 |
| 5038 | 0 | US | H01L | H01L0021 | 46 | 30 | 5 | 1.285714286 | 0.56 |
| 5039 | 0 | JP | H01L | H01L0027 | 65 | 3 | 1 | 1.666666667 | 0.20 |
| 5040 | 0 | FI | H01L | H01L0027 | 17 | 5 | 0 | 1 | 0.00 |
| 5041 | 0 | JP | H01L | H01L0029 | 39 | 51 | 0 | 1.75 | 0.00 |
| 5042 | 0 | JP | H01L | H01L0029 | 4 | 6 | 0 | 1 | 0.00 |
| 5043 | 0 | NL | C03B | C03B0008 | 8 | 8 | 6 | 1 | 1.00 |
| 5044 | 0 | TW | H01J | H01J0001 | 5 | 3 | 0 | 1 | 0.00 |
| 5045 | 0 | US | C30B | C30B0025 | 54 | 29 | 36 | 2 | 9.00 |
| 5046 | 0 | JP | F21S | F21S0013 | 15 | 19 | 1 | 1.666666667 | 0.20 |
| 5047 | 0 | TW | H01L | H01L0027 | 35 | 5 | 1 | 1 | 0.50 |
| 5048 | 0 | TW | H01L | H01L0029 | 4 | 2 | 0 | 1 | 0.00 |
| 5049 | 0 | JP | H01L | H01L0033 | 8 | 13 | 0 | 1 | 0.00 |
| 5050 | 0 | DE | H01L | H01L0021 | 29 | 89 | 0 | 3.125 | 0.00 |
| 5051 | 0 | TW | H01L | H01L0027 | 5 | 1 | 0 | 2 | 0.00 |
| 5052 | 0 | TW | H01L | H01L0023 | 13 | 5 | 0 | 1 | 0.00 |
| 5053 | 0 | US | H01L | H01L0027 | 19 | 11 | 6 | 1 | 6.00 |
| 5054 | 1 | JP | H01J | H01J0001 | 14 | 38 | 4 | 1.666666667 | 0.80 |
| 5055 | 0 | US | G03C | G03C0001 | 12 | 15 | 1 | 1 | 0.50 |
| 5056 | 0 | US | H01L | H01L0031 | 35 | 35 | 5 | 2 | 2.50 |
| 5057 | 0 | TW | F21V | F21V0009 | 1 | 8 | 13 | 1.4 | 1.86 |
| 5058 | 0 | US | F21V | F21V0011 | 12 | 18 | 10 | 1 | 2.00 |
| 5059 | 0 | TW | H01L | H01L0021 | 21 | 16 | 0 | 4.25 | 0.00 |
| 5060 | 0 | DE | H01L | H01L0021 | 40 | 30 | 0 | 1.428571429 | 0.00 |
| 5061 | 0 | JP | H01L | H01L0021 | 4 | 6 | 4 | 1.5 | 1.33 |
| 5062 | 0 | TW | H01L | H01L0021 | 25 | 13 | 0 | 1 | 0.00 |
| 5063 | 0 | JP | H01L | H01L0029 | 28 | 16 | 0 | 1 | 0.00 |
| 5064 | 0 | US | H01L | H01L0029 | 18 | 20 | 10 | 1 | 10.00 |
| 5065 | 0 | KR | H01L | H01L0029 | 29 | 2 | 4 | 1 | 2.00 |
| 5066 | 0 | JP | H01L | H01L0029 | 19 | 9 | 2 | 1 | 1.00 |
| 5067 | 0 | US | H01L | H01L0027 | 25 | 206 | 3 | 1 | 3.00 |
| 5068 | 0 | JP | H01S | H01S0005 | 3 | 46 | 0 | 3 | 0.00 |
| 5069 | 0 | US | H01L | H01L0031 | 34 | 35 | 1 | 2 | 0.50 |
| 5070 | 0 | JP | F21V | F21V0009 | 13 | 7 | 7 | 1 | 1.40 |
| 5071 | 0 | US | H01L | H01L0033 | 35 | 18 | 5 | 1.5 | 0.42 |
| 5072 | 0 | US | B01J | B01J0020 | 8 | 41 | 5 | 3 | 1.67 |
| 5073 | 0 | JP | B32B | B32B0009 | 13 | 24 | 0 | 1.25 | 0.00 |
| 5074 | 0 | US | H01L | H01L0029 | 105 | 110 | 17 | 18.83333333 | 0.15 |
| 5075 | 0 | US | H01L | H01L0021 | 9 | 25 | 2 | 1.5 | 0.67 |
| 5076 | 0 | JP | F21V | F21V0007 | 16 | 5 | 6 | 1 | 3.00 |
| 5077 | 1 | US | H01L | H01L0033 | 24 | 114 | 13 | 8.8 | 0.15 |
| 5078 | 0 | JP | H01L | H01L0029 | 32 | 15 | 16 | 1.5 | 2.67 |
| 5079 | 0 | JP | H01L | H01L0033 | 21 | 22 | 0 | 1.333333333 | 0.00 |
| 5080 | 0 | TW | H01L | H01L0023 | 7 | 3 | 4 | 2 | 2.00 |
| 5081 | 0 | KR | H05B | H05B0033 | 33 | 15 | 0 | 1.333333333 | 0.00 |
| 5082 | 0 | US | H01L | H01L0029 | 36 | 119 | 7 | 18.83333333 | 0.06 |
| 5083 | 0 | SG | H01J | H01J0009 | 4 | 22 | 4 | 2 | 1.00 |
| 5084 | 0 | US | H01L | H01L0029 | 34 | 216 | 4 | 3 | 0.33 |
| 5085 | 0 | JP | H05B | H05B0033 | 5 | 173 | 26 | 5 | 0.29 |
| 5086 | 0 | US | H01L | H01L0021 | 25 | 33 | 7 | 1.5 | 0.78 |
| 5087 | 0 | TW | H01L | H01L0033 | 22 | 10 | 0 | 1 | 0.00 |
| 5088 | 0 | US | H01L | H01L0029 | 23 | 100 | 3 | 3 | 0.25 |
| 5089 | 0 | US | H01J | H01J0009 | 23 | 11 | 1 | 1 | 0.50 |
| 5090 | 0 | JP | C30B | C30B0009 | 29 | 13 | 2 | 2.5 | 0.40 |
| 5091 | 0 | JP | C30B | C30B0025 | 9 | 10 | 0 | 1.5 | 0.00 |
| 5092 | 0 | US | H01L | H01L0021 | 31 | 58 | 2 | 30.25 | 0.01 |
| 5093 | 0 | JP | H01L | H01L0021 | 1 | 14 | 0 | 2.5 | 0.00 |
| 5094 | 0 | JP | H01L | H01L0027 | 15 | 12 | 0 | 1.5 | 0.00 |
| 5095 | 0 | US | H01L | H01L0029 | 24 | 12 | 1 | 1 | 0.17 |
| 5096 | 0 | JP | H01L | H01L0029 | 7 | 25 | 3 | 1.5 | 1.00 |
| 5097 | 0 | JP | F21V | F21V0007 | 4 | 11 | 6 | 1 | 1.50 |
| 5098 | 0 | DE | H01L | H01L0021 | 26 | 21 | 0 | 1 | 0.00 |
| 5099 | 0 | US | H01J | H01J0005 | 38 | 94 | 9 | 9 | 0.14 |
| 5100 | 0 | US | H01L | H01L0033 | 5 | 17 | 7 | 2 | 0.50 |
| 5101 | 0 | JP | H01L | H01L0021 | 11 | 4 | 2 | 1.5 | 0.67 |
| 5102 | 0 | KR | H05B | H05B0033 | 25 | 3 | 0 | 2.5 | 0.00 |
| 5103 | 0 | KR | G02B | G02B0013 | 10 | 1 | 4 | 1 | 1.33 |
| 5104 | 0 | US | H01L | H01L0021 | 16 | 60 | 15 | 5.166666667 | 0.48 |
| 5105 | 0 | JP | C30B | C30B0001 | 20 | 25 | 0 | 1.333333333 | 0.00 |
| 5106 | 0 | US | H01J | H01J0001 | 37 | 168 | 3 | 18.83333333 | 0.03 |
| 5107 | 0 | JP | H01L | H01L0021 | 8 | 16 | 1 | 1.5 | 0.33 |
| 5108 | 0 | JP | C09K | C09K0011 | 18 | 24 | 10 | 1 | 1.25 |
| 5109 | 0 | DE | H01L | H01L0027 | 16 | 7 | 4 | 1.25 | 0.80 |
| 5110 | 0 | TW | H01L | H01L0029 | 39 | 3 | 0 | 1.25 | 0.00 |
| 5111 | 0 | US | H01L | H01L0021 | 41 | 164 | 3 | 4.5 | 0.33 |
| 5112 | 0 | SI | H01B | H01B0013 | 9 | 12 | 2 | 1 | 0.33 |
| 5113 | 0 | JP | H01L | H01L0021 | 2 | 18 | 0 | 1.333333333 | 0.00 |
| 5114 | 0 | US | H01L | H01L0029 | 10 | 1 | 0 | 1 | 0.00 |
| 5115 | 0 | US | H01J | H01J0001 | 40 | 153 | 0 | 18.83333333 | 0.00 |
| 5116 | 0 | US | F21V | F21V0021 | 18 | 21 | 0 | 4 | 0.00 |
| 5117 | 0 | KR | H01L | H01L0021 | 20 | 21 | 3 | 1.25 | 0.60 |
| 5118 | 0 | JP | H01L | H01L0033 | 12 | 7 | 1 | 1 | 0.33 |
| 5119 | 0 | JP | H01L | H01L0033 | 1 | 13 | 3 | 1.333333333 | 0.75 |
| 5120 | 0 | US | F21V | F21V0007 | 14 | 88 | 19 | 2.5 | 1.90 |
| 5121 | 0 | TW | H05B | H05B0033 | 8 | 4 | 0 | 1 | 0.00 |
| 5122 | 0 | US | G03B | G03B0021 | 20 | 7 | 6 | 1.333333333 | 1.50 |
| 5123 | 0 | JP | C30B | C30B0025 | 17 | 31 | 0 | 1 | 0.00 |
| 5124 | 0 | DE | C09K | C09K0011 | 17 | 364 | 9 | 11.77777778 | 0.08 |
| 5125 | 0 | US | F21V | F21V0007 | 18 | 6 | 6 | 1 | 1.20 |
| 5126 | 0 | FR | H01L | H01L0021 | 22 | 19 | 4 | 3.272727273 | 0.11 |
| 5127 | 0 | JP | H01L | H01L0029 | 2 | 63 | 6 | 5 | 0.60 |
| 5128 | 0 | KR | H01L | H01L0021 | 9 | 16 | 1 | 1 | 0.25 |
| 5129 | 0 | US | H01L | H01L0025 | 11 | 55 | 11 | 4 | 0.39 |
| 5130 | 0 | US | F21V | F21V0013 | 9 | 8 | 2 | 1 | 0.40 |
| 5131 | 0 | US | H01L | H01L0021 | 18 | 31 | 4 | 2.333333333 | 0.29 |
| 5132 | 0 | JP | C09K | C09K0011 | 14 | 15 | 1 | 1 | 0.50 |
| 5133 | 0 | JP | H01L | H01L0023 | 2 | 18 | 2 | 1.333333333 | 0.50 |
| 5134 | 0 | DE | C09K | C09K0011 | 1 | 25 | 0 | 1.571428571 | 0.00 |
| 5135 | 0 | US | H01L | H01L0021 | 19 | 6 | 2 | 4 | 0.25 |
| 5136 | 0 | DE | H01L | H01L0021 | 22 | 8 | 2 | 1 | 0.67 |
| 5137 | 0 | JP | H01L | H01L0027 | 12 | 35 | 0 | 2.5 | 0.00 |
| 5138 | 0 | DE | H01L | H01L0027 | 48 | 57 | 1 | 1 | 0.33 |
| 5139 | 0 | TW | H01L | H01L0029 | 44 | 3 | 0 | 1.5 | 0.00 |
| 5140 | 0 | JP | H01L | H01L0023 | 4 | 15 | 0 | 1.285714286 | 0.00 |
| 5141 | 0 | DE | H01L | H01L0029 | 19 | 16 | 0 | 2.25 | 0.00 |
| 5142 | 0 | KR | H01L | H01L0029 | 11 | 2 | 20 | 1 | 4.00 |
| 5143 | 0 | US | H01L | H01L0021 | 5 | 23 | 2 | 1.4 | 0.29 |
| 5144 | 0 | JP | H01L | H01L0029 | 20 | 11 | 4 | 1.333333333 | 1.00 |
| 5145 | 0 | JP | H01L | H01L0027 | 10 | 13 | 2 | 1.5 | 0.67 |
| 5146 | 0 | KR | H01L | H01L0029 | 9 | 7 | 4 | 1 | 1.33 |
| 5147 | 0 | KR | H01L | H01L0023 | 20 | 18 | 4 | 1 | 1.33 |
| 5148 | 0 | US | B23K | B23K0035 | 74 | 51 | 2 | 2.111111111 | 0.11 |
| 5149 | 0 | US | H01J | H01J0001 | 43 | 147 | 5 | 18.83333333 | 0.04 |
| 5150 | 0 | US | H01L | H01L0021 | 36 | 13 | 2 | 1 | 0.25 |
| 5151 | 0 | BE | B05D | B05D0005 | 26 | 11 | 0 | 6.666666667 | 0.00 |
| 5152 | 0 | CA | B05D | B05D0001 | 23 | 26 | 3 | 2.166666667 | 0.23 |
| 5153 | 0 | JP | H01L | H01L0051 | 23 | 6 | 0 | 1.5 | 0.00 |
| 5154 | 0 | CA | H01L | H01L0021 | 6 | 70 | 8 | 5.166666667 | 0.26 |
| 5155 | 0 | JP | H01L | H01L0027 | 33 | 11 | 10 | 1 | 5.00 |
| 5156 | 0 | DE | H01L | H01L0033 | 36 | 25 | 9 | 2.25 | 0.50 |
| 5157 | 0 | JP | H01J | H01J0001 | 8 | 7 | 1 | 1 | 0.33 |
| 5158 | 0 | JP | F21V | F21V0007 | 15 | 9 | 4 | 1.5 | 1.33 |
| 5159 | 0 | US | H01L | H01L0021 | 14 | 7 | 0 | 2 | 0.00 |
| 5160 | 0 | JP | H01L | H01L0021 | 14 | 13 | 0 | 2 | 0.00 |
| 5161 | 0 | US | F21V | F21V0023 | 25 | 36 | 19 | 2.764705882 | 0.40 |
| 5162 | 0 | US | H01L | H01L0021 | 42 | 13 | 8 | 1.571428571 | 0.73 |
| 5163 | 0 | US | H01L | H01L0021 | 10 | 206 | 6 | 1.428571429 | 0.60 |
| 5164 | 0 | JP | H01L | H01L0051 | 7 | 19 | 3 | 1.25 | 0.60 |
| 5165 | 0 | TW | H01L | H01L0021 | 20 | 1 | 0 | 1.5 | 0.00 |
| 5166 | 0 | US | H01L | H01L0029 | 20 | 66 | 0 | 11.33333333 | 0.00 |
| 5167 | 0 | JP | H01L | H01L0021 | 26 | 18 | 5 | 1 | 1.00 |
| 5168 | 0 | JP | H01L | H01L0033 | 8 | 14 | 5 | 1.5 | 0.83 |
| 5169 | 0 | US | H01L | H01L0029 | 35 | 109 | 7 | 1.285714286 | 0.78 |
| 5170 | 0 | TW | H01L | H01L0029 | 24 | 5 | 4 | 1 | 2.00 |
| 5171 | 0 | JP | H01L | H01L0029 | 7 | 5 | 6 | 1 | 3.00 |
| 5172 | 0 | KR | H01L | H01L0029 | 25 | 16 | 4 | 1 | 1.33 |
| 5173 | 0 | US | G02B | G02B0006 | 20 | 15 | 1 | 1 | 1.00 |
| 5174 | 0 | JP | C30B | C30B0029 | 15 | 12 | 5 | 1 | 2.50 |
| 5175 | 0 | DE | C30B | C30B0013 | 33 | 13 | 0 | 1 | 0.00 |
| 5176 | 0 | JP | C30B | C30B0029 | 26 | 27 | 1 | 1 | 0.50 |
| 5177 | 0 | US | H01J | H01J0063 | 25 | 33 | 0 | 1 | 0.00 |
| 5178 | 0 | FR | H01L | H01L0021 | 30 | 20 | 3 | 1.125 | 0.33 |
| 5179 | 0 | FR | H01L | H01L0021 | 25 | 14 | 0 | 1 | 0.00 |
| 5180 | 0 | JP | H01L | H01L0029 | 16 | 3 | 1 | 1 | 0.50 |
| 5181 | 0 | DE | B29D | B29D0011 | 13 | 28 | 7 | 1 | 1.75 |
| 5182 | 0 | JP | H01L | H01L0031 | 17 | 13 | 5 | 2 | 0.83 |
| 5183 | 0 | JP | B41J | B41J0002 | 11 | 18 | 0 | 1 | 0.00 |
| 5184 | 0 | JP | H01L | H01L0029 | 11 | 3 | 0 | 1.5 | 0.00 |
| 5185 | 0 | US | H01J | H01J0063 | 24 | 32 | 3 | 1 | 3.00 |
| 5186 | 0 | US | H01L | H01L0031 | 31 | 98 | 5 | 3.875 | 0.16 |
| 5187 | 0 | KR | H01L | H01L0023 | 7 | 2 | 2 | 1 | 0.50 |
| 5188 | 0 | SG | F21V | F21V0007 | 16 | 3 | 4 | 1 | 1.33 |
| 5189 | 0 | US | F21S | F21S0008 | 2 | 10 | 5 | 1 | 1.00 |
| 5190 | 0 | DE | H05B | H05B0033 | 34 | 33 | 0 | 3 | 0.00 |
| 5191 | 0 | US | H01L | H01L0029 | 11 | 53 | 0 | 2 | 0.00 |
| 5192 | 0 | US | H01L | H01L0029 | 3 | 59 | 3 | 14.25 | 0.03 |
| 5193 | 0 | TW | H01L | H01L0029 | 21 | 9 | 1 | 1 | 1.00 |
| 5194 | 0 | TW | H01L | H01L0027 | 17 | 1 | 0 | 1 | 0.00 |
| 5195 | 0 | DE | H01L | H01L0033 | 17 | 131 | 8 | 5.857142857 | 0.20 |
| 5196 | 0 | US | H01L | H01L0023 | 9 | 38 | 2 | 4 | 0.50 |
| 5197 | 0 | KR | H01L | H01L0021 | 19 | 6 | 4 | 1 | 1.00 |
| 5198 | 0 | JP | C09K | C09K0011 | 5 | 21 | 2 | 2 | 0.50 |
| 5199 | 0 | US | H01L | H01L0029 | 17 | 79 | 12 | 2.2 | 1.09 |
| 5200 | 0 | JP | H05B | H05B0033 | 32 | 66 | 1 | 1 | 0.20 |
| 5201 | 0 | SG | H01S | H01S0005 | 15 | 8 | 0 | 1.2 | 0.00 |
| 5202 | 0 | TW | H01L | H01L0021 | 12 | 2 | 0 | 1 | 0.00 |
| 5203 | 0 | TW | H01L | H01L0021 | 12 | 9 | 0 | 2 | 0.00 |
| 5204 | 0 | US | H01K | H01K0001 | 18 | 94 | 6 | 9 | 0.10 |
| 5205 | 0 | JP | B41J | B41J0002 | 9 | 3 | 6 | 1 | 1.50 |
| 5206 | 0 | JP | H01L | H01L0033 | 16 | 4 | 1 | 1 | 0.50 |
| 5207 | 0 | TW | H01L | H01L0029 | 27 | 7 | 1 | 1 | 0.33 |
| 5208 | 0 | GB | H05B | H05B0033 | 40 | 18 | 1 | 1 | 0.50 |
| 5209 | 0 | US | B65B | B65B0007 | 29 | 51 | 2 | 5.333333333 | 0.06 |
| 5210 | 0 | US | F21V | F21V0009 | 4 | 1 | 2 | 1 | 0.29 |
| 5211 | 0 | SG | H01L | H01L0021 | 12 | 6 | 0 | 2 | 0.00 |
| 5212 | 0 | JP | H01L | H01L0021 | 16 | 8 | 0 | 1 | 0.00 |
| 5213 | 0 | DE | C09K | C09K0011 | 20 | 428 | 4 | 11.77777778 | 0.04 |
| 5214 | 0 | US | H01L | H01L0033 | 17 | 78 | 3 | 1.666666667 | 0.60 |
| 5215 | 0 | KR | H01L | H01L0033 | 13 | 5 | 1 | 1 | 0.33 |
| 5216 | 0 | TW | H01L | H01L0027 | 1 | 2 | 0 | 2 | 0.00 |
| 5217 | 0 | US | H01J | H01J0001 | 72 | 153 | 2 | 18.83333333 | 0.02 |
| 5218 | 0 | TW | H01L | H01L0021 | 6 | 2 | 1 | 1.5 | 0.33 |
| 5219 | 0 | TW | H01L | H01L0029 | 17 | 2 | 0 | 1 | 0.00 |
| 5220 | 0 | SG | H01L | H01L0033 | 56 | 33 | 5 | 2 | 2.50 |
| 5221 | 0 | JP | H01L | H01L0023 | 18 | 3 | 4 | 1 | 2.00 |
| 5222 | 0 | JP | H01L | H01L0029 | 18 | 10 | 15 | 2 | 1.25 |
| 5223 | 0 | US | H01L | H01L0029 | 15 | 19 | 18 | 1.333333333 | 4.50 |
| 5224 | 0 | US | G02B | G02B0017 | 11 | 51 | 17 | 1 | 4.25 |
| 5225 | 0 | US | F21S | F21S0013 | 71 | 50 | 21 | 3 | 7.00 |
| 5226 | 0 | KR | H01L | H01L0027 | 15 | 4 | 2 | 1 | 0.67 |
| 5227 | 0 | US | H01L | H01L0029 | 30 | 9 | 4 | 1 | 1.00 |
| 5228 | 0 | JP | F21S | F21S0008 | 12 | 7 | 0 | 1 | 0.00 |
| 5229 | 0 | JP | H01L | H01L0023 | 20 | 25 | 7 | 1 | 2.33 |
| 5230 | 0 | CN | H04N | H04N0007 | 5 | 3 | 1 | 1 | 0.50 |
| 5231 | 0 | TW | H01L | H01L0027 | 9 | 7 | 3 | 1.333333333 | 0.75 |
| 5232 | 0 | JP | H01L | H01L0023 | 14 | 11 | 0 | 2 | 0.00 |
| 5233 | 0 | US | H01L | H01L0021 | 35 | 31 | 5 | 2.6 | 0.38 |
| 5234 | 0 | US | F21V | F21V0007 | 14 | 2 | 3 | 1 | 1.50 |
| 5235 | 1 | US | F21V | F21V0009 | 70 | 95 | 8 | 3.333333333 | 0.40 |
| 5236 | 0 | DE | B05D | B05D0005 | 13 | 10 | 0 | 1.166666667 | 0.00 |
| 5237 | 0 | JP | H01L | H01L0021 | 2 | 14 | 0 | 1 | 0.00 |
| 5238 | 0 | TW | H01L | H01L0029 | 17 | 4 | 1 | 1 | 1.00 |
| 5239 | 0 | US | F21V | F21V0007 | 7 | 1 | 1 | 1 | 1.00 |
| 5240 | 0 | TW | F21V | F21V0007 | 18 | 5 | 0 | 1 | 0.00 |
| 5241 | 0 | TW | G02B | G02B0006 | 14 | 3 | 0 | 1 | 0.00 |
| 5242 | 0 | US | H01L | H01L0021 | 25 | 47 | 4 | 1 | 0.57 |
| 5243 | 0 | US | C09K | C09K0011 | 11 | 8 | 0 | 2 | 0.00 |
| 5244 | 0 | JP | H01L | H01L0029 | 15 | 10 | 8 | 1.5 | 2.67 |
| 5245 | 0 | JP | H01L | H01L0029 | 20 | 16 | 2 | 1 | 0.67 |
| 5246 | 0 | JP | H01J | H01J0001 | 39 | 72 | 9 | 1.166666667 | 1.29 |
| 5247 | 0 | JP | H01J | H01J0033 | 14 | 14 | 1 | 1.75 | 0.14 |
| 5248 | 0 | JP | F21S | F21S0002 | 11 | 19 | 0 | 1 | 0.00 |
| 5249 | 0 | SG | F21V | F21V0007 | 18 | 8 | 9 | 1 | 1.80 |
| 5250 | 0 | JP | C30B | C30B0009 | 19 | 31 | 0 | 1 | 0.00 |
| 5251 | 0 | US | H01L | H01L0027 | 52 | 34 | 11 | 1 | 5.50 |
| 5252 | 0 | KR | H01L | H01L0021 | 39 | 7 | 4 | 2 | 0.40 |
| 5253 | 0 | KR | H01L | H01L0021 | 11 | 11 | 1 | 1 | 0.33 |
| 5254 | 0 | JP | H01J | H01J0001 | 6 | 21 | 1 | 1 | 0.17 |
| 5255 | 0 | CA | F21V | F21V0021 | 15 | 6 | 6 | 1 | 6.00 |
| 5256 | 0 | US | H01L | H01L0021 | 19 | 26 | 0 | 1.125 | 0.00 |
| 5257 | 0 | US | F21V | F21V0021 | 23 | 37 | 4 | 1 | 2.00 |
| 5258 | 0 | CN | F21V | F21V0007 | 2 | 7 | 3 | 1 | 1.50 |
| 5259 | 0 | JP | H01J | H01J0009 | 21 | 29 | 0 | 1 | 0.00 |
| 5260 | 0 | US | H01L | H01L0029 | 32 | 52 | 1 | 1.5 | 0.11 |
| 5261 | 0 | JP | H01L | H01L0029 | 7 | 9 | 6 | 1 | 2.00 |
| 5262 | 0 | US | C30B | C30B0023 | 35 | 18 | 2 | 1 | 0.67 |
| 5263 | 0 | US | H01J | H01J0061 | 30 | 50 | 2 | 19.83333333 | 0.01 |
| 5264 | 0 | US | C30B | C30B0029 | 42 | 23 | 1 | 1 | 1.00 |
| 5265 | 0 | JP | H01L | H01L0023 | 17 | 11 | 2 | 1 | 1.00 |
| 5266 | 0 | US | H01L | H01L0021 | 26 | 72 | 0 | 30.25 | 0.00 |
| 5267 | 0 | JP | H01S | H01S0005 | 12 | 6 | 0 | 1 | 0.00 |
| 5268 | 0 | US | H01L | H01L0021 | 20 | 12 | 4 | 1.5 | 1.33 |
| 5269 | 0 | TW | F21V | F21V0005 | 20 | 5 | 3 | 1 | 3.00 |
| 5270 | 0 | US | H01L | H01L0029 | 8 | 218 | 2 | 3.333333333 | 0.10 |
| 5271 | 0 | DE | H01L | H01L0021 | 4 | 32 | 0 | 1.142857143 | 0.00 |
| 5272 | 0 | JP | H01L | H01L0021 | 12 | 18 | 1 | 2 | 0.17 |
| 5273 | 0 | KR | H01L | H01L0021 | 13 | 4 | 0 | 1.333333333 | 0.00 |
| 5274 | 0 | US | C30B | C30B0025 | 18 | 11 | 2 | 2 | 1.00 |
| 5275 | 0 | DE | H01L | H01L0027 | 4 | 20 | 0 | 1 | 0.00 |
| 5276 | 0 | SG | F21V | F21V0007 | 10 | 9 | 0 | 1 | 0.00 |
| 5277 | 0 | US | H01L | H01L0021 | 23 | 108 | 3 | 1.285714286 | 0.33 |
| 5278 | 0 | JP | H01L | H01L0021 | 4 | 24 | 0 | 1.5 | 0.00 |
| 5279 | 0 | US | H01J | H01J0001 | 12 | 95 | 7 | 9 | 0.11 |
| 5280 | 0 | US | C30B | C30B0025 | 35 | 30 | 0 | 1.285714286 | 0.00 |
| 5281 | 0 | US | H05B | H05B0033 | 8 | 34 | 3 | 1 | 0.50 |
| 5282 | 0 | SG | H01J | H01J0001 | 8 | 6 | 0 | 3 | 0.00 |
| 5283 | 0 | JP | H01L | H01L0031 | 6 | 29 | 2 | 1.333333333 | 0.50 |
| 5284 | 0 | US | G08B | G08B0005 | 31 | 8 | 15 | 1 | 3.00 |
| 5285 | 0 | TW | H01L | H01L0021 | 6 | 7 | 0 | 1.25 | 0.00 |
| 5286 | 0 | US | F21V | F21V0007 | 20 | 86 | 3 | 1 | 3.00 |
| 5287 | 0 | DE | H01L | H01L0033 | 14 | 160 | 7 | 5.857142857 | 0.17 |
| 5288 | 0 | JP | H01J | H01J0021 | 15 | 11 | 7 | 1 | 3.50 |
| 5289 | 0 | KR | H01S | H01S0003 | 10 | 12 | 0 | 1 | 0.00 |
| 5290 | 0 | KR | H01L | H01L0025 | 10 | 16 | 1 | 1.25 | 0.20 |
| 5291 | 0 | US | C30B | C30B0025 | 43 | 23 | 3 | 1.5 | 0.50 |
| 5292 | 0 | US | F21V | F21V0007 | 36 | 173 | 13 | 1 | 6.50 |
| 5293 | 0 | US | H01L | H01L0021 | 24 | 7 | 0 | 1 | 0.00 |
| 5294 | 0 | KR | H01L | H01L0027 | 9 | 3 | 4 | 1 | 1.33 |
| 5295 | 0 | JP | H01L | H01L0027 | 10 | 14 | 0 | 1 | 0.00 |
| 5296 | 0 | US | H05B | H05B0033 | 29 | 43 | 6 | 2 | 3.00 |
| 5297 | 0 | US | H01L | H01L0023 | 4 | 10 | 1 | 2 | 0.50 |
| 5298 | 0 | US | H01L | H01L0023 | 25 | 11 | 0 | 2 | 0.00 |
| 5299 | 0 | US | H01L | H01L0029 | 34 | 168 | 6 | 1 | 1.00 |
| 5300 | 0 | JP | H01L | H01L0031 | 20 | 8 | 0 | 1.5 | 0.00 |
| 5301 | 0 | SG | H01L | H01L0029 | 12 | 19 | 3 | 1.5 | 0.50 |
| 5302 | 0 | JP | H01L | H01L0029 | 9 | 7 | 0 | 1 | 0.00 |
| 5303 | 0 | TW | H01L | H01L0031 | 3 | 18 | 5 | 1 | 5.00 |
| 5304 | 0 | JP | H05B | H05B0033 | 25 | 180 | 9 | 5 | 0.10 |
| 5305 | 0 | US | H01L | H01L0033 | 24 | 7 | 5 | 3 | 1.67 |
| 5306 | 0 | TW | F21V | F21V0001 | 19 | 16 | 10 | 1 | 2.50 |
| 5307 | 0 | US | H01J | H01J0001 | 43 | 151 | 1 | 18.83333333 | 0.01 |
| 5308 | 0 | JP | H01L | H01L0021 | 2 | 9 | 0 | 1.2 | 0.00 |
| 5309 | 0 | JP | H01L | H01L0029 | 2 | 37 | 0 | 3.75 | 0.00 |
| 5310 | 0 | JP | H01L | H01L0033 | 12 | 35 | 9 | 1 | 4.50 |
| 5311 | 0 | KR | H01J | H01J0001 | 10 | 5 | 0 | 1 | 0.00 |
| 5312 | 0 | DE | F21V | F21V0009 | 9 | 19 | 1 | 1 | 0.50 |
| 5313 | 0 | TW | H01L | H01L0021 | 22 | 13 | 1 | 1 | 0.50 |
| 5314 | 0 | TW | H01L | H01L0021 | 7 | 13 | 4 | 2 | 1.00 |
| 5315 | 0 | TW | H01L | H01L0021 | 19 | 11 | 0 | 1.25 | 0.00 |
| 5316 | 0 | US | H01L | H01L0031 | 10 | 42 | 4 | 1.333333333 | 0.33 |
| 5317 | 0 | KR | H01L | H01L0029 | 20 | 6 | 0 | 1.25 | 0.00 |
| 5318 | 0 | US | F21V | F21V0005 | 18 | 5 | 6 | 1.5 | 2.00 |
| 5319 | 0 | JP | H01L | H01L0033 | 9 | 23 | 2 | 1.5 | 0.22 |
| 5320 | 0 | TW | H01L | H01L0023 | 6 | 13 | 1 | 2 | 0.25 |
| 5321 | 0 | US | H01L | H01L0021 | 18 | 47 | 5 | 1.6 | 0.63 |
| 5322 | 0 | JP | H01L | H01L0021 | 18 | 16 | 0 | 2.2 | 0.00 |
| 5323 | 0 | TW | H01L | H01L0027 | 12 | 14 | 1 | 1.333333333 | 0.25 |
| 5324 | 1 | TW | H01L | H01L0025 | 25 | 12 | 0 | 1 | 0.00 |
| 5325 | 0 | TW | H01L | H01L0029 | 11 | 3 | 1 | 4 | 0.13 |
| 5326 | 0 | JP | H01L | H01L0033 | 14 | 27 | 0 | 1.166666667 | 0.00 |
| 5327 | 0 | TW | H01L | H01L0029 | 20 | 7 | 2 | 1 | 2.00 |
| 5328 | 0 | US | H05B | H05B0033 | 18 | 21 | 1 | 1 | 1.00 |
| 5329 | 0 | TW | H01L | H01L0021 | 13 | 1 | 0 | 1.333333333 | 0.00 |
| 5330 | 0 | TW | H01L | H01L0029 | 6 | 2 | 0 | 1 | 0.00 |
| 5331 | 0 | TW | H01L | H01L0021 | 15 | 2 | 0 | 1 | 0.00 |
| 5332 | 0 | US | F21V | F21V0005 | 26 | 11 | 12 | 1 | 6.00 |
| 5333 | 0 | US | G02B | G02B0027 | 28 | 24 | 23 | 4.285714286 | 0.77 |
| 5334 | 0 | KR | F21V | F21V0008 | 8 | 16 | 1 | 1 | 0.33 |
| 5335 | 0 | US | H01L | H01L0021 | 20 | 8 | 11 | 3 | 0.61 |
| 5336 | 0 | US | H01L | H01L0021 | 22 | 2 | 1 | 1.166666667 | 0.14 |
| 5337 | 0 | JP | H01L | H01L0021 | 13 | 10 | 0 | 1 | 0.00 |
| 5338 | 0 | JP | H01L | H01L0029 | 22 | 70 | 4 | 1 | 2.00 |
| 5339 | 0 | DE | H01L | H01L0033 | 19 | 17 | 4 | 1 | 1.00 |
| 5340 | 0 | TW | H01L | H01L0029 | 17 | 3 | 0 | 1.5 | 0.00 |
| 5341 | 1 | DE | F21K | F21K0002 | 43 | 430 | 6 | 11.77777778 | 0.06 |
| 5342 | 0 | JP | H01L | H01L0029 | 2 | 4 | 2 | 1 | 0.50 |
| 5343 | 0 | TW | H01L | H01L0029 | 24 | 2 | 1 | 1 | 1.00 |
| 5344 | 0 | JP | H01L | H01L 31 | 2 | 7 | 0 | 1.5 | 0.00 |
| 5345 | 0 | US | G02B | G02B0006 | 10 | 4 | 0 | 2 | 0.00 |
| 5346 | 0 | US | H01L | H01L0021 | 7 | 16 | 0 | 3 | 0.00 |
| 5347 | 0 | JP | F21V | F21V0007 | 25 | 50 | 6 | 1.555555556 | 0.43 |
| 5348 | 0 | US | H01L | H01L0021 | 20 | 7 | 2 | 2.8 | 0.14 |
| 5349 | 0 | JP | H01L | H01L0027 | 14 | 8 | 4 | 1 | 2.00 |
| 5350 | 0 | JP | H01L | H01L0029 | 9 | 30 | 0 | 1.375 | 0.00 |
| 5351 | 0 | US | F21V | F21V0023 | 25 | 372 | 14 | 23.28571429 | 0.04 |
| 5352 | 0 | DE | C09K | C09K0011 | 16 | 12 | 13 | 1.125 | 1.44 |
| 5353 | 0 | US | H01L | H01L0021 | 5 | 16 | 1 | 2 | 0.17 |
| 5354 | 0 | US | H01L | H01L0021 | 14 | 3 | 2 | 1.8 | 0.22 |
| 5355 | 0 | US | H01L | H01L0029 | 32 | 13 | 17 | 3 | 0.94 |
| 5356 | 0 | JP | H01L | H01L0033 | 3 | 36 | 0 | 2 | 0.00 |
| 5357 | 0 | JP | H01L | H01L0029 | 2 | 3 | 2 | 1 | 0.67 |
| 5358 | 0 | US | C01G | C01G0053 | 14 | 2 | 0 | 4.461538462 | 0.00 |
| 5359 | 0 | MI | H05K | H05K0007 | 16 | 25 | 2 | 1.5 | 0.67 |
| 5360 | 0 | US | H01L | H01L0021 | 14 | 25 | 1 | 3 | 0.33 |
| 5361 | 0 | US | H01L | H01L0021 | 18 | 22 | 0 | 1.375 | 0.00 |
| 5362 | 0 | US | H01L | H01L0051 | 32 | 28 | 1 | 1 | 0.20 |
| 5363 | 0 | JP | H01L | H01L0021 | 20 | 18 | 1 | 2 | 0.25 |
| 5364 | 0 | TW | H01L | H01L0027 | 11 | 6 | 5 | 2 | 2.50 |
| 5365 | 0 | TW | H01L | H01L0031 | 51 | 22 | 4 | 2.166666667 | 0.31 |
| 5366 | 0 | JP | H01L | H01L0023 | 8 | 5 | 2 | 1 | 1.00 |
| 5367 | 0 | JP | H01J | H01J0001 | 11 | 24 | 0 | 3.5 | 0.00 |
| 5368 | 0 | JP | H01S | H01S0005 | 17 | 3 | 1 | 1.5 | 0.33 |
| 5369 | 0 | JP | F21V | F21V0007 | 5 | 8 | 7 | 1 | 3.50 |
| 5370 | 0 | TW | H01L | H01L0021 | 8 | 8 | 0 | 1.5 | 0.00 |
| 5371 | 0 | SG | H01L | H01L0029 | 12 | 6 | 0 | 1 | 0.00 |
| 5372 | 0 | DE | H01L | H01L0029 | 28 | 6 | 0 | 1 | 0.00 |
| 5373 | 0 | TW | H01L | H01L0029 | 19 | 8 | 0 | 1 | 0.00 |
| 5374 | 0 | US | H01L | H01L0027 | 35 | 161 | 1 | 18.83333333 | 0.01 |
| 5375 | 0 | SG | H01L | H01L0029 | 20 | 16 | 0 | 1 | 0.00 |
| 5376 | 0 | JP | H01L | H01L0023 | 5 | 21 | 1 | 2.5 | 0.20 |
| 5377 | 0 | JP | H01L | H01L0021 | 4 | 4 | 2 | 1.666666667 | 0.40 |
| 5378 | 0 | US | H01K | H01K0003 | 10 | 10 | 0 | 2 | 0.00 |
| 5379 | 0 | US | H01L | H01L0027 | 25 | 15 | 18 | 2.333333333 | 1.29 |
| 5380 | 0 | KR | H01L | H01L0033 | 19 | 9 | 5 | 3.25 | 0.19 |
| 5381 | 0 | JP | H05B | H05B0033 | 15 | 184 | 13 | 5 | 0.14 |
| 5382 | 0 | US | F21V | F21V0007 | 17 | 4 | 3 | 1 | 0.50 |
| 5383 | 0 | JP | H05B | H05B0033 | 20 | 13 | 0 | 1 | 0.00 |
| 5384 | 0 | TW | H01L | H01L0021 | 20 | 4 | 0 | 1 | 0.00 |
| 5385 | 0 | JP | H01L | H01L0021 | 16 | 13 | 2 | 1 | 1.00 |
| 5386 | 0 | US | H01J | H01J0005 | 9 | 62 | 4 | 1 | 0.67 |
| 5387 | 0 | JP | C07F | C07F0005 | 2 | 20 | 2 | 1 | 1.00 |
| 5388 | 0 | JP | H01L | H01L0021 | 18 | 25 | 1 | 1.25 | 0.10 |
| 5389 | 0 | JP | H01L | H01L0029 | 12 | 5 | 0 | 1 | 0.00 |
| 5390 | 0 | JP | H01L | H01L0033 | 12 | 529 | 4 | 1.888888889 | 0.24 |
| 5391 | 0 | TW | G02B | G02B0027 | 20 | 6 | 1 | 2 | 0.25 |
| 5392 | 0 | US | H01L | H01L0029 | 19 | 27 | 6 | 1.333333333 | 0.75 |
| 5393 | 0 | SG | H01L | H01L0031 | 7 | 2 | 2 | 1.333333333 | 0.50 |
| 5394 | 0 | DE | H01L | H01L0021 | 12 | 34 | 5 | 2 | 0.42 |
| 5395 | 0 | US | H01L | H01L0033 | 23 | 25 | 3 | 1.222222222 | 0.27 |
| 5396 | 0 | JP | H01L | H01L0033 | 34 | 4 | 1 | 1.333333333 | 0.25 |
| 5397 | 0 | US | G02B | G02B0006 | 6 | 7 | 1 | 2 | 0.50 |
| 5398 | 0 | JP | H01L | H01L0021 | 4 | 24 | 0 | 1.333333333 | 0.00 |
| 5399 | 0 | KR | H01L | H01L0021 | 14 | 21 | 3 | 2.2 | 0.27 |
| 5400 | 0 | KR | H01L | H01L0021 | 9 | 4 | 0 | 1 | 0.00 |
| 5401 | 0 | TW | H01L | H01L0021 | 20 | 1 | 1 | 1 | 0.50 |
| 5402 | 0 | JP | H01L | H01L0021 | 24 | 52 | 2 | 3.75 | 0.13 |
| 5403 | 0 | JP | G02B | G02B0006 | 15 | 14 | 0 | 1 | 0.00 |
| 5404 | 0 | JP | H01L | H01L0029 | 16 | 21 | 1 | 1 | 0.17 |
| 5405 | 0 | DE | H01L | H01L0027 | 20 | 15 | 5 | 1 | 0.83 |
| 5406 | 0 | US | H01L | H01L0033 | 9 | 3 | 1 | 2.375 | 0.05 |
| 5407 | 0 | DE | H01L | H01L0027 | 22 | 36 | 0 | 1 | 0.00 |
| 5408 | 0 | US | H01L | H01L0035 | 14 | 9 | 2 | 1 | 0.40 |
| 5409 | 0 | JP | H01J | H01J0005 | 18 | 2 | 0 | 1 | 0.00 |
| 5410 | 0 | US | H01J | H01J0001 | 23 | 25 | 0 | 1 | 0.00 |
| 5411 | 0 | TW | H01L | H01L0033 | 20 | 1 | 1 | 1 | 0.50 |
| 5412 | 0 | US | H01J | H01J0005 | 57 | 94 | 3 | 9 | 0.05 |
| 5413 | 0 | US | H01L | H01L0021 | 51 | 78 | 7 | 1.6 | 0.88 |
| 5414 | 0 | US | H01L | H01L0021 | 7 | 64 | 2 | 1.571428571 | 0.18 |
| 5415 | 0 | TW | H01L | H01L0031 | 42 | 24 | 10 | 2.166666667 | 0.77 |
| 5416 | 0 | SG | H01L | H01L0029 | 14 | 4 | 7 | 1 | 3.50 |
| 5417 | 0 | JP | H01L | H01L0027 | 19 | 7 | 0 | 1.5 | 0.00 |
| 5418 | 0 | US | H01L | H01L0031 | 27 | 22 | 6 | 2 | 3.00 |
| 5419 | 0 | KR | G02F | G02F0001 | 20 | 19 | 4 | 1.5 | 0.67 |
| 5420 | 0 | US | G02F | G02F0001 | 25 | 180 | 5 | 2 | 2.50 |
| 5421 | 0 | JP | H01L | H01L0051 | 10 | 61 | 2 | 1 | 0.50 |
| 5422 | 0 | JP | H01L | H01L0033 | 18 | 3 | 0 | 1.666666667 | 0.00 |
| 5423 | 0 | JP | H01L | H01L0027 | 7 | 11 | 6 | 1 | 3.00 |
| 5424 | 0 | US | F21L | F21L0004 | 11 | 35 | 4 | 1 | 0.57 |
| 5425 | 0 | DE | F21V | F21V0005 | 13 | 9 | 0 | 1.333333333 | 0.00 |
| 5426 | 0 | US | C09K | C09K0011 | 28 | 21 | 2 | 1 | 0.33 |
| 5427 | 0 | JP | G02B | G02B0006 | 18 | 9 | 0 | 1 | 0.00 |
| 5428 | 0 | JP | H01L | H01L0031 | 1 | 62 | 8 | 5.4 | 0.30 |
| 5429 | 0 | TW | H01L | H01L0023 | 13 | 15 | 2 | 1 | 1.00 |
| 5430 | 0 | JP | H05B | H05B0033 | 28 | 36 | 10 | 1.75 | 1.43 |
| 5431 | 0 | US | H05B | H05B0033 | 17 | 9 | 2 | 1.5 | 0.67 |
| 5432 | 0 | TW | H01L | H01L0029 | 4 | 2 | 0 | 1 | 0.00 |
| 5433 | 0 | DE | H01L | H01L0023 | 32 | 105 | 1 | 1.888888889 | 0.06 |
| 5434 | 0 | US | F21V | F21V0005 | 7 | 175 | 10 | 4.285714286 | 0.33 |
| 5435 | 0 | US | C09K | C09K0011 | 21 | 4 | 0 | 1 | 0.00 |
| 5436 | 0 | US | C09K | C09K0011 | 31 | 7 | 0 | 1 | 0.00 |
| 5437 | 0 | KR | H01L | H01L0021 | 8 | 17 | 0 | 2 | 0.00 |
| 5438 | 0 | NL | G06F | G06F0017 | 22 | 37 | 3 | 1.555555556 | 0.21 |
| 5439 | 0 | JP | F21V | F21V0007 | 22 | 9 | 1 | 1 | 0.17 |
| 5440 | 0 | TW | H01L | H01L0021 | 5 | 2 | 1 | 1 | 0.50 |
| 5441 | 0 | US | C09K | C09K0011 | 16 | 24 | 0 | 1 | 0.00 |
| 5442 | 0 | JP | H01L | H01L0021 | 2 | 20 | 3 | 1 | 0.43 |
| 5443 | 0 | JP | H01L | H01L0021 | 15 | 64 | 3 | 1 | 1.00 |
| 5444 | 0 | JP | H01L | H01L0029 | 10 | 5 | 0 | 1 | 0.00 |
| 5445 | 0 | JP | H01L | H01L0033 | 11 | 2 | 0 | 1 | 0.00 |
| 5446 | 0 | TW | H01L | H01L0029 | 10 | 2 | 0 | 6 | 0.00 |
| 5447 | 0 | JP | H01L | H01L0031 | 15 | 11 | 0 | 1 | 0.00 |
| 5448 | 0 | US | H01L | H01L0051 | 33 | 27 | 1 | 1 | 0.25 |
| 5449 | 0 | US | G02B | G02B0007 | 16 | 56 | 0 | 1 | 0.00 |
| 5450 | 0 | JP | H01L | H01L0029 | 6 | 6 | 0 | 1 | 0.00 |
| 5451 | 0 | US | H01L | H01L0021 | 14 | 9 | 4 | 1 | 1.00 |
| 5452 | 0 | US | H01L | H01L0027 | 11 | 18 | 4 | 1 | 0.57 |
| 5453 | 0 | JP | H01L | H01L0023 | 16 | 20 | 0 | 1 | 0.00 |
| 5454 | 0 | JP | H01L | H01L0031 | 25 | 7 | 0 | 1.5 | 0.00 |
| 5455 | 0 | DE | H01L | H01L0023 | 13 | 2 | 2 | 1 | 2.00 |
| 5456 | 0 | JP | H01J | H01J0005 | 16 | 5 | 0 | 1 | 0.00 |
| 5457 | 0 | US | H01L | H01L0021 | 22 | 14 | 5 | 1 | 2.50 |
| 5458 | 0 | US | H01L | H01L0021 | 26 | 45 | 0 | 3 | 0.00 |
| 5459 | 0 | JP | H01L | H01L0021 | 25 | 24 | 1 | 1.666666667 | 0.20 |
| 5460 | 0 | JP | H01L | H01L0029 | 12 | 13 | 1 | 1 | 0.50 |
| 5461 | 0 | JP | H01L | H01L0027 | 14 | 8 | 2 | 1 | 0.50 |
| 5462 | 0 | DE | B60Q | B60Q0001 | 22 | 20 | 0 | 1.25 | 0.00 |
| 5463 | 0 | JP | C30B | C30B0011 | 5 | 6 | 0 | 2 | 0.00 |
| 5464 | 0 | DE | H01L | H01L0051 | 33 | 15 | 0 | 2 | 0.00 |
| 5465 | 0 | US | H01L | H01L0027 | 66 | 45 | 1 | 2.857142857 | 0.05 |
| 5466 | 0 | KR | H01L | H01L0021 | 11 | 4 | 1 | 1 | 0.25 |
| 5467 | 0 | US | B05B | B05B0013 | 37 | 32 | 3 | 5.333333333 | 0.09 |
| 5468 | 0 | JP | H01L | H01L0027 | 58 | 6 | 1 | 2 | 0.25 |
| 5469 | 0 | US | F28D | F28D0015 | 17 | 14 | 1 | 1 | 0.50 |
| 5470 | 0 | DE | H01L | H01L0033 | 13 | 3 | 0 | 1 | 0.00 |
| 5471 | 0 | US | F21V | F21V0021 | 10 | 92 | 4 | 3.333333333 | 0.20 |
| 5472 | 0 | JP | H01L | H01L0029 | 30 | 23 | 0 | 8.866666667 | 0.00 |
| 5473 | 0 | JP | G02B | G02B0006 | 22 | 12 | 0 | 1 | 0.00 |
| 5474 | 0 | KR | H01L | H01L0033 | 10 | 8 | 2 | 1 | 2.00 |
| 5475 | 0 | US | H01L | H01L0027 | 25 | 159 | 2 | 18.83333333 | 0.02 |
| 5476 | 0 | TW | F21V | F21V0029 | 18 | 3 | 5 | 1.5 | 1.67 |
| 5477 | 0 | JP | H01L | H01L0029 | 32 | 14 | 1 | 1.75 | 0.14 |
| 5478 | 0 | US | H01S | H01S0005 | 32 | 12 | 0 | 1 | 0.00 |
| 5479 | 0 | KR | H01L | H01L0027 | 13 | 18 | 3 | 1.25 | 0.60 |
| 5480 | 0 | KR | H01L | H01L0029 | 13 | 12 | 0 | 3 | 0.00 |
| 5481 | 0 | JP | H01L | H01L0033 | 19 | 12 | 1 | 1.4 | 0.14 |
| 5482 | 0 | KR | H01L | H01L0023 | 19 | 26 | 0 | 1.2 | 0.00 |
| 5483 | 0 | US | F21K | F21K0099 | 6 | 7 | 0 | 1 | 0.00 |
| 5484 | 0 | JP | H01L | H01L0027 | 20 | 13 | 3 | 2 | 0.75 |
| 5485 | 0 | JP | H01L | H01L0027 | 16 | 25 | 0 | 1.25 | 0.00 |
| 5486 | 0 | JP | B32B | B32B0009 | 1 | 40 | 0 | 1.5 | 0.00 |
| 5487 | 0 | US | H01J | H01J0005 | 3 | 5 | 1 | 1 | 0.20 |
| 5488 | 0 | US | H01L | H01L0027 | 29 | 18 | 2 | 1 | 1.00 |
| 5489 | 0 | TW | F21V | F21V0009 | 17 | 6 | 1 | 1 | 0.50 |
| 5490 | 0 | US | C09K | C09K0011 | 9 | 18 | 0 | 1 | 0.00 |
| 5491 | 0 | JP | H01L | H01L0051 | 4 | 15 | 0 | 1 | 0.00 |
| 5492 | 0 | US | H05B | H05B0033 | 9 | 16 | 2 | 1 | 1.00 |
| 5493 | 0 | DE | H01L | H01L0021 | 21 | 106 | 2 | 1.888888889 | 0.12 |
| 5494 | 0 | GB | H01L | H01L0021 | 18 | 42 | 2 | 1.5 | 0.67 |
| 5495 | 0 | KR | H01L | H01L0029 | 11 | 8 | 0 | 3 | 0.00 |
| 5496 | 0 | JP | H01L | H01L0033 | 17 | 6 | 1 | 1 | 0.25 |
| 5497 | 0 | US | H01L | H01L0033 | 6 | 127 | 0 | 8.8 | 0.00 |
| 5498 | 0 | TW | H01L | H01L0023 | 18 | 4 | 1 | 1 | 0.50 |
| 5499 | 0 | JP | G01D | G01D0011 | 10 | 6 | 3 | 1 | 0.60 |
| 5500 | 0 | JP | F21V | F21V0029 | 20 | 8 | 2 | 1 | 1.00 |
| 5501 | 0 | JP | F21V | F21V0033 | 6 | 16 | 2 | 1.25 | 0.40 |
| 5502 | 0 | UA | C30B | C30B0019 | 6 | 2 | 0 | 1 | 0.00 |
| 5503 | 0 | TW | H01L | H01L0021 | 14 | 17 | 1 | 2 | 0.25 |
| 5504 | 0 | FR | H01L | H01L0021 | 21 | 13 | 0 | 2.75 | 0.00 |
| 5505 | 0 | JP | H01L | H01L0029 | 40 | 82 | 0 | 1.5 | 0.00 |
| 5506 | 0 | US | B22F | B22F0009 | 63 | 180 | 3 | 19.83333333 | 0.01 |
| 5507 | 0 | KR | F21V | F21V0029 | 24 | 2 | 0 | 1.333333333 | 0.00 |
| 5508 | 0 | JP | H01L | H01L0021 | 11 | 23 | 0 | 1.8 | 0.00 |
| 5509 | 0 | JP | H01L | H01L0021 | 10 | 28 | 2 | 1.4 | 0.29 |
| 5510 | 0 | KR | H01L | H01L0029 | 13 | 5 | 0 | 1 | 0.00 |
| 5511 | 0 | JP | H01L | H01L0033 | 14 | 7 | 1 | 1 | 0.50 |
| 5512 | 0 | CA | B05D | B05D0005 | 38 | 196 | 2 | 1.555555556 | 0.14 |
| 5513 | 0 | US | H01L | H01L0021 | 19 | 33 | 2 | 1 | 0.29 |
| 5514 | 0 | SG | H01L | H01L0033 | 11 | 27 | 0 | 1.333333333 | 0.00 |
| 5515 | 0 | TW | H01L | H01L0029 | 13 | 13 | 0 | 2 | 0.00 |
| 5516 | 0 | TW | H01L | H01L0029 | 12 | 5 | 1 | 1 | 0.50 |
| 5517 | 0 | TW | H01L | H01L0021 | 18 | 3 | 1 | 4 | 0.13 |
| 5518 | 0 | JP | H01L | H01L0029 | 14 | 3 | 4 | 1.75 | 0.57 |
| 5519 | 0 | JP | H01L | H01L0027 | 10 | 6 | 0 | 1.25 | 0.00 |
| 5520 | 0 | JP | H01L | H01L0033 | 13 | 20 | 2 | 1.5 | 0.33 |
| 5521 | 0 | DE | H01L | H01L0033 | 15 | 28 | 8 | 2.333333333 | 1.14 |
| 5522 | 0 | US | H01L | H01L0033 | 15 | 111 | 1 | 1.833333333 | 0.09 |
| 5523 | 0 | KR | H01L | H01L0027 | 6 | 24 | 4 | 1 | 1.00 |
| 5524 | 0 | JP | H01L | H01L0021 | 16 | 5 | 0 | 1.666666667 | 0.00 |
| 5525 | 0 | US | H01L | H01L0033 | 17 | 27 | 4 | 1 | 4.00 |
| 5526 | 0 | JP | H01L | H01L0033 | 5 | 25 | 6 | 1 | 1.20 |
| 5527 | 0 | JP | H01J | H01J0001 | 10 | 111 | 6 | 2 | 1.00 |
| 5528 | 0 | TW | C30B | C30B0025 | 31 | 9 | 0 | 1.5 | 0.00 |
| 5529 | 0 | US | H01L | H01L0027 | 34 | 5 | 0 | 1 | 0.00 |
| 5530 | 0 | TW | H01L | H01L0021 | 14 | 8 | 0 | 3 | 0.00 |
| 5531 | 0 | DE | H01L | H01L0021 | 31 | 16 | 0 | 2.25 | 0.00 |
| 5532 | 0 | JP | C30B | C30B0029 | 15 | 15 | 1 | 1.5 | 0.33 |
| 5533 | 0 | US | H01L | H01L0021 | 18 | 4 | 0 | 1 | 0.00 |
| 5534 | 0 | KR | H01L | H01L0021 | 16 | 11 | 0 | 1 | 0.00 |
| 5535 | 0 | JP | H01L | H01L0027 | 3 | 13 | 1 | 2.5 | 0.20 |
| 5536 | 0 | JP | C09K | C09K0011 | 3 | 39 | 1 | 2 | 0.25 |
| 5537 | 0 | DE | H01L | H01L0021 | 34 | 32 | 0 | 1 | 0.00 |
| 5538 | 0 | KR | H01L | H01L0027 | 19 | 4 | 0 | 1.333333333 | 0.00 |
| 5539 | 0 | DE | H01L | H01L0033 | 41 | 69 | 6 | 1.333333333 | 0.75 |
| 5540 | 0 | JP | H01L | H01L0029 | 7 | 20 | 0 | 1 | 0.00 |
| 5541 | 0 | DE | H01L | H01L0023 | 7 | 2 | 0 | 1.333333333 | 0.00 |
| 5542 | 0 | US | F21V | F21V0013 | 10 | 16 | 2 | 1.5 | 0.67 |
| 5543 | 0 | US | H01L | H01L0021 | 20 | 37 | 3 | 1 | 0.50 |
| 5544 | 0 | TW | H01L | H01L0021 | 26 | 6 | 4 | 1 | 2.00 |
| 5545 | 0 | FR | H01L | H01L0021 | 20 | 133 | 1 | 1 | 0.14 |
| 5546 | 0 | US | H01L | H01L0029 | 27 | 14 | 3 | 1 | 1.50 |
| 5547 | 0 | FR | H01L | H01L0021 | 20 | 24 | 1 | 1.25 | 0.20 |
| 5548 | 0 | JP | H01L | H01L0021 | 6 | 15 | 0 | 2.2 | 0.00 |
| 5549 | 0 | JP | H01L | H01L0033 | 4 | 6 | 0 | 1 | 0.00 |
| 5550 | 0 | US | H01L | H01L0021 | 34 | 57 | 3 | 1.2 | 0.50 |
| 5551 | 0 | TW | F21V | F21V0029 | 20 | 4 | 1 | 1 | 0.33 |
| 5552 | 0 | JP | F21V | F21V0007 | 27 | 12 | 0 | 1 | 0.00 |
| 5553 | 0 | US | H01L | H01L0051 | 31 | 16 | 0 | 1 | 0.00 |
| 5554 | 0 | KR | H01L | H01L0021 | 15 | 10 | 0 | 1 | 0.00 |
| 5555 | 0 | TW | H01L | H01L0027 | 24 | 3 | 0 | 2 | 0.00 |
| 5556 | 0 | DE | H01L | H01L0033 | 10 | 11 | 5 | 1.666666667 | 1.00 |
| 5557 | 0 | JP | H01L | H01L0029 | 16 | 6 | 0 | 1 | 0.00 |
| 5558 | 0 | US | H01L | H01L0033 | 11 | 19 | 0 | 1.5 | 0.00 |
| 5559 | 0 | JP | F21V | F21V0001 | 5 | 4 | 11 | 1 | 3.67 |
| 5560 | 0 | TW | F21V | F21V0021 | 2 | 6 | 0 | 1 | 0.00 |
| 5561 | 0 | JP | C30B | C30B0025 | 11 | 6 | 0 | 1.5 | 0.00 |
| 5562 | 0 | FR | C30B | C30B0029 | 24 | 14 | 1 | 1 | 0.17 |
| 5563 | 0 | JP | G03C | G03C0001 | 39 | 31 | 1 | 3.5 | 0.14 |
| 5564 | 0 | US | F21V | F21V0005 | 26 | 151 | 4 | 1 | 2.00 |
| 5565 | 0 | US | H01J | H01J0009 | 18 | 4 | 5 | 1 | 5.00 |
| 5566 | 0 | DE | H01L | H01L0027 | 25 | 12 | 0 | 1 | 0.00 |
| 5567 | 0 | JP | H01L | H01L0027 | 7 | 4 | 1 | 1 | 0.33 |
| 5568 | 0 | US | H01L | H01L0029 | 20 | 14 | 4 | 1.571428571 | 0.36 |
| 5569 | 0 | US | H01L | H01L0033 | 42 | 24 | 5 | 1.25 | 1.00 |
| 5570 | 0 | DE | H01L | H01L0029 | 6 | 12 | 4 | 1.333333333 | 1.00 |
| 5571 | 0 | JP | G02F | G02F0001 | 4 | 36 | 1 | 1 | 0.17 |
| 5572 | 0 | AU | H01R | H01R0025 | 28 | 7 | 2 | 1 | 1.00 |
| 5573 | 0 | KR | C30B | C30B0035 | 7 | 2 | 0 | 1.5 | 0.00 |
| 5574 | 0 | US | C09K | C09K0011 | 16 | 16 | 0 | 1 | 0.00 |
| 5575 | 0 | US | H01L | H01L0021 | 24 | 31 | 0 | 1.285714286 | 0.00 |
| 5576 | 0 | JP | H01L | H01L0021 | 15 | 16 | 2 | 1.25 | 0.40 |
| 5577 | 0 | FR | H01L | H01L0021 | 19 | 63 | 2 | 3 | 0.10 |
| 5578 | 0 | JP | H01L | H01L0021 | 12 | 44 | 3 | 2 | 0.38 |
| 5579 | 0 | JP | H01L | H01L0033 | 10 | 5 | 2 | 1 | 0.67 |
| 5580 | 0 | JP | H01L | H01L0033 | 24 | 8 | 0 | 1 | 0.00 |
| 5581 | 0 | TW | H01L | H01L0033 | 3 | 6 | 0 | 1 | 0.00 |
| 5582 | 0 | JP | H01S | H01S0005 | 9 | 53 | 0 | 3 | 0.00 |
| 5583 | 0 | JP | H01S | H01S0003 | 12 | 13 | 0 | 1 | 0.00 |
| 5584 | 0 | US | H01L | H01L0021 | 16 | 21 | 7 | 3 | 0.39 |
| 5585 | 0 | TW | H01L | H01L0021 | 39 | 17 | 4 | 1.5 | 1.33 |
| 5586 | 0 | US | H01J | H01J0005 | 18 | 96 | 1 | 9 | 0.02 |
| 5587 | 0 | JP | H01L | H01L0021 | 17 | 9 | 0 | 1 | 0.00 |
| 5588 | 0 | JP | H01L | H01L0029 | 8 | 24 | 0 | 1 | 0.00 |
| 5589 | 0 | KR | H01L | H01L0027 | 9 | 1 | 0 | 1.666666667 | 0.00 |
| 5590 | 0 | JP | H01L | H01L0027 | 16 | 90 | 0 | 1 | 0.00 |
| 5591 | 0 | JP | H01L | H01L0027 | 18 | 39 | 1 | 1.5 | 0.33 |
| 5592 | 0 | KR | H01L | H01L0029 | 7 | 17 | 2 | 1 | 0.50 |
| 5593 | 0 | TW | H01L | H01L0033 | 6 | 1 | 3 | 1.666666667 | 0.60 |
| 5594 | 0 | JP | H01L | H01L0033 | 8 | 21 | 3 | 1 | 1.00 |
| 5595 | 0 | US | G09F | G09F0013 | 45 | 39 | 0 | 1 | 0.00 |
| 5596 | 0 | DE | G02B | G02B0006 | 40 | 30 | 0 | 1 | 0.00 |
| 5597 | 0 | JP | H01L | H01L0021 | 8 | 6 | 0 | 1.5 | 0.00 |
| 5598 | 0 | US | H01L | H01L0021 | 22 | 6 | 0 | 1 | 0.00 |
| 5599 | 0 | JP | H01L | H01L0027 | 4 | 15 | 0 | 1.25 | 0.00 |
| 5600 | 0 | TW | H01L | H01L0033 | 18 | 2 | 0 | 6 | 0.00 |
| 5601 | 0 | KR | B23K | B23K0031 | 4 | 6 | 0 | 1 | 0.00 |
| 5602 | 0 | JP | H01L | H01L0029 | 9 | 9 | 1 | 1 | 0.25 |
| 5603 | 0 | JP | H01J | H01J0005 | 17 | 4 | 0 | 1.4 | 0.00 |
| 5604 | 0 | DE | H01J | H01J0063 | 5 | 9 | 1 | 1.333333333 | 0.13 |
| 5605 | 0 | KR | F21V | F21V0007 | 12 | 9 | 17 | 1 | 5.67 |
| 5606 | 0 | JP | H01L | H01L0021 | 9 | 15 | 0 | 1.285714286 | 0.00 |
| 5607 | 0 | TW | H01L | H01L0021 | 10 | 7 | 1 | 1 | 1.00 |
| 5608 | 0 | US | H01L | H01L0029 | 3 | 50 | 11 | 1.666666667 | 1.10 |
| 5609 | 0 | DE | H01L | H01L0033 | 18 | 16 | 1 | 1 | 0.25 |
| 5610 | 0 | JP | G09F | G09F0009 | 27 | 45 | 1 | 1.166666667 | 0.14 |
| 5611 | 0 | TW | H01L | H01L0033 | 20 | 4 | 0 | 1 | 0.00 |
| 5612 | 0 | US | H01L | H01L0021 | 63 | 27 | 3 | 1.5 | 1.00 |
| 5613 | 0 | US | H01L | H01L0029 | 14 | 31 | 4 | 1.285714286 | 0.44 |
| 5614 | 0 | KR | H01L | H01L0027 | 10 | 1 | 0 | 1 | 0.00 |
| 5615 | 0 | KR | H01L | H01L0033 | 19 | 3 | 1 | 1 | 0.33 |
| 5616 | 0 | DE | H01L | H01L0033 | 8 | 17 | 0 | 1 | 0.00 |
| 5617 | 0 | JP | H01L | H01L0029 | 7 | 7 | 0 | 1 | 0.00 |
| 5618 | 0 | KR | H01C | H01C0007 | 20 | 66 | 1 | 4.333333333 | 0.04 |
| 5619 | 0 | KR | H01L | H01L0031 | 15 | 12 | 0 | 1 | 0.00 |
| 5620 | 0 | DE | H01L | H01L0023 | 15 | 26 | 0 | 1 | 0.00 |
| 5621 | 0 | SG | C09K | C09K0011 | 1 | 38 | 2 | 1 | 0.67 |
| 5622 | 0 | KR | F21S | F21S0004 | 22 | 12 | 1 | 1 | 0.25 |
| 5623 | 0 | TW | F21V | F21V0029 | 6 | 5 | 0 | 1 | 0.00 |
| 5624 | 0 | KR | H01L | H01L0021 | 19 | 7 | 1 | 1.2 | 0.17 |
| 5625 | 0 | JP | H01L | H01L0033 | 5 | 13 | 0 | 1 | 0.00 |
| 5626 | 0 | TW | H01L | H01L0029 | 19 | 76 | 0 | 53 | 0.00 |
| 5627 | 0 | TW | H01J | H01J0001 | 40 | 7 | 0 | 1 | 0.00 |
| 5628 | 0 | JP | C09K | C09K0011 | 20 | 12 | 2 | 2.5 | 0.40 |
| 5629 | 0 | JP | C08L | C08L0063 | 9 | 19 | 0 | 1 | 0.00 |
| 5630 | 0 | JP | H01L | H01L0029 | 8 | 29 | 0 | 1.5 | 0.00 |
| 5631 | 0 | DE | H01L | H01L0027 | 21 | 21 | 0 | 1 | 0.00 |
| 5632 | 0 | TW | H01L | H01L0033 | 9 | 2 | 2 | 1 | 2.00 |
| 5633 | 0 | US | H01L | H01L0021 | 25 | 34 | 4 | 1 | 2.00 |
| 5634 | 0 | DE | F21V | F21V0019 | 13 | 17 | 0 | 1 | 0.00 |
| 5635 | 0 | KR | H01L | H01L0021 | 25 | 10 | 0 | 1 | 0.00 |
| 5636 | 0 | DE | H01L | H01L0027 | 27 | 2 | 0 | 1 | 0.00 |
| 5637 | 0 | US | H01L | H01L0023 | 57 | 104 | 0 | 1 | 0.00 |
| 5638 | 0 | KR | H01L | H01L0029 | 8 | 17 | 3 | 1.2 | 0.50 |
| 5639 | 0 | TW | H01L | H01L0051 | 17 | 4 | 0 | 1 | 0.00 |
| 5640 | 0 | SG | H01L | H01L0029 | 2 | 2 | 0 | 1 | 0.00 |
| 5641 | 0 | US | F21S | F21S0013 | 13 | 9 | 2 | 2 | 0.50 |
| 5642 | 0 | KR | F21V | F21V0009 | 14 | 4 | 1 | 1 | 0.33 |
| 5643 | 0 | JP | C09K | C09K0011 | 20 | 89 | 0 | 1 | 0.00 |
| 5644 | 0 | JP | C09K | C09K0011 | 14 | 96 | 2 | 2.571428571 | 0.11 |
| 5645 | 0 | JP | C09K | C09K0011 | 25 | 94 | 4 | 2.333333333 | 0.57 |
| 5646 | 0 | US | H01L | H01L0021 | 11 | 42 | 1 | 1 | 0.50 |
| 5647 | 0 | JP | H01L | H01L0027 | 5 | 19 | 0 | 1.5 | 0.00 |
| 5648 | 0 | JP | H01L | H01L0029 | 12 | 13 | 3 | 1.25 | 0.60 |
| 5649 | 0 | TW | H01L | H01L0033 | 14 | 1 | 0 | 2 | 0.00 |
| 5650 | 0 | US | H01L | H01L0027 | 5 | 17 | 0 | 1.75 | 0.00 |
| 5651 | 0 | US | H01L | H01L0021 | 37 | 31 | 3 | 1.166666667 | 0.43 |
| 5652 | 0 | JP | H01J | H01J0063 | 19 | 110 | 0 | 1 | 0.00 |
| 5653 | 0 | TW | H05B | H05B0033 | 6 | 15 | 0 | 2 | 0.00 |
| 5654 | 0 | JP | G02B | G02B0006 | 10 | 4 | 0 | 1 | 0.00 |
| 5655 | 0 | US | H01L | H01L0021 | 91 | 161 | 2 | 18.83333333 | 0.02 |
| 5656 | 0 | JP | H01L | H01L0021 | 4 | 14 | 0 | 1 | 0.00 |
| 5657 | 0 | DE | F21V | F21V0033 | 14 | 33 | 1 | 2 | 0.50 |
| 5658 | 0 | TW | H01J | H01J0001 | 12 | 16 | 2 | 1.5 | 0.67 |
| 5659 | 0 | JP | G11B | G11B0007 | 4 | 9 | 0 | 2 | 0.00 |
| 5660 | 0 | TW | F21S | F21S0004 | 8 | 2 | 0 | 1 | 0.00 |
| 5661 | 0 | SG | F21V | F21V0007 | 17 | 10 | 2 | 1 | 2.00 |
| 5662 | 0 | US | H01L | H01L0051 | 13 | 12 | 0 | 1 | 0.00 |
| 5663 | 0 | DE | C09K | C09K0011 | 18 | 17 | 0 | 1 | 0.00 |
| 5664 | 0 | DE | H01L | H01L0021 | 18 | 22 | 0 | 1 | 0.00 |
| 5665 | 0 | US | H01L | H01L0021 | 34 | 42 | 1 | 2 | 0.25 |
| 5666 | 0 | US | H01L | H01L0031 | 19 | 71 | 0 | 1 | 0.00 |
| 5667 | 0 | US | H01L | H01L0021 | 18 | 78 | 1 | 1.571428571 | 0.09 |
| 5668 | 0 | US | H01L | H01L0021 | 6 | 20 | 5 | 2.666666667 | 0.31 |
| 5669 | 0 | JP | H01L | H01L0029 | 34 | 14 | 0 | 1.142857143 | 0.00 |
| 5670 | 0 | TW | H01L | H01L0033 | 7 | 3 | 0 | 6 | 0.00 |
| 5671 | 0 | US | H01L | H01L0029 | 21 | 17 | 1 | 1.333333333 | 0.25 |
| 5672 | 0 | JP | H01L | H01L0027 | 21 | 52 | 1 | 2 | 0.25 |
| 5673 | 0 | TW | H01L | H01L0029 | 8 | 3 | 1 | 1.5 | 0.33 |
| 5674 | 0 | KR | H01L | H01L0023 | 22 | 15 | 0 | 1 | 0.00 |
| 5675 | 0 | TW | H01L | H01L0021 | 10 | 1 | 0 | 2 | 0.00 |
| 5676 | 0 | KR | H01L | H01L0021 | 18 | 4 | 1 | 1 | 0.33 |
| 5677 | 0 | US | G03F | G03F0001 | 55 | 25 | 0 | 4 | 0.00 |
| 5678 | 0 | JP | H01L | H01L0027 | 21 | 5 | 1 | 1 | 0.50 |
| 5679 | 0 | JP | H01L | H01L0027 | 22 | 42 | 0 | 1.333333333 | 0.00 |
| 5680 | 0 | US | H01J | H01J0017 | 4 | 7 | 0 | 1 | 0.00 |
| 5681 | 0 | JP | B41J | B41J0002 | 5 | 15 | 1 | 1.333333333 | 0.25 |
| 5682 | 0 | FR | C30B | C30B0025 | 15 | 10 | 1 | 1.166666667 | 0.14 |
| 5683 | 0 | JP | C09K | C09K0011 | 13 | 18 | 2 | 2 | 0.50 |
| 5684 | 0 | US | H01L | H01L0021 | 8 | 29 | 0 | 1.285714286 | 0.00 |
| 5685 | 0 | US | H01L | H01L0021 | 31 | 79 | 3 | 2 | 1.50 |
| 5686 | 0 | JP | H01L | H01L0029 | 2 | 26 | 0 | 1.4 | 0.00 |
| 5687 | 0 | JP | H01L | H01L0021 | 18 | 37 | 1 | 1.25 | 0.10 |
| 5688 | 0 | JP | H01L | H01L0021 | 20 | 34 | 0 | 1 | 0.00 |
| 5689 | 0 | TW | H01J | H01J0001 | 14 | 15 | 1 | 1 | 0.50 |
| 5690 | 0 | DE | H01J | H01J0001 | 19 | 17 | 0 | 1 | 0.00 |
| 5691 | 0 | JP | H01J | H01J0001 | 12 | 3 | 0 | 1 | 0.00 |
| 5692 | 0 | JP | F21V | F21V0007 | 18 | 4 | 3 | 1 | 1.00 |
| 5693 | 0 | TW | F21V | F21V0007 | 16 | 5 | 0 | 1 | 0.00 |
| 5694 | 0 | KR | H01L | H01L0021 | 17 | 8 | 0 | 1.333333333 | 0.00 |
| 5695 | 0 | US | H01L | H01L0021 | 11 | 47 | 0 | 1.285714286 | 0.00 |
| 5696 | 0 | KR | H01L | H01L0033 | 7 | 3 | 0 | 1 | 0.00 |
| 5697 | 0 | JP | H01L | H01L0033 | 15 | 14 | 2 | 1 | 0.50 |
| 5698 | 0 | JP | H01L | H01L0033 | 13 | 16 | 1 | 1 | 0.50 |
| 5699 | 0 | US | F21V | F21V0007 | 12 | 95 | 1 | 2.5 | 0.10 |
| 5700 | 0 | US | G03B | G03B0021 | 25 | 4 | 3 | 1.333333333 | 0.75 |
| 5701 | 0 | US | F21V | F21V0021 | 23 | 1 | 1 | 1 | 0.17 |
| 5702 | 0 | DE | H01L | H01L0029 | 29 | 16 | 0 | 1 | 0.00 |
| 5703 | 0 | JP | H01L | H01L0021 | 17 | 21 | 0 | 1.285714286 | 0.00 |
| 5704 | 0 | US | H01L | H01L0021 | 31 | 173 | 7 | 2.8 | 0.50 |
| 5705 | 0 | JP | H01L | H01L0029 | 8 | 11 | 1 | 1 | 0.25 |
| 5706 | 0 | JP | H01L | H01L0031 | 6 | 12 | 0 | 1.5 | 0.00 |
| 5707 | 0 | JP | H01L | H01L0027 | 16 | 4 | 0 | 1 | 0.00 |
| 5708 | 0 | JP | H01L | H01L0021 | 3 | 37 | 4 | 1.166666667 | 0.57 |
| 5709 | 0 | JP | G01N | G01N0021 | 16 | 19 | 1 | 1.5 | 0.33 |
| 5710 | 0 | JP | H01S | H01S0003 | 8 | 446 | 2 | 2 | 0.14 |
| 5711 | 0 | KR | G02B | G02B0006 | 17 | 9 | 0 | 1.2 | 0.00 |
| 5712 | 0 | TW | C30B | C30B0025 | 14 | 9 | 0 | 1.2 | 0.00 |
| 5713 | 0 | US | H01L | H01L0021 | 19 | 19 | 1 | 1.5 | 0.33 |
| 5714 | 0 | US | C09K | C09K0011 | 6 | 9 | 0 | 1 | 0.00 |
| 5715 | 0 | US | C09K | C09K0011 | 23 | 28 | 2 | 1 | 0.67 |
| 5716 | 0 | CN | H01L | H01L0021 | 5 | 2 | 0 | 1 | 0.00 |
| 5717 | 0 | US | H01J | H01J0001 | 22 | 33 | 1 | 1.6 | 0.13 |
| 5718 | 0 | US | G03B | G03B0021 | 21 | 68 | 7 | 2 | 1.75 |
| 5719 | 0 | TW | H01L | H01L0029 | 24 | 20 | 1 | 1 | 0.25 |
| 5720 | 0 | JP | H01L | H01L0027 | 12 | 6 | 1 | 1 | 0.20 |
| 5721 | 0 | KR | H01L | H01L0033 | 9 | 5 | 2 | 1.25 | 0.40 |
| 5722 | 0 | JP | H01L | H01L0033 | 20 | 15 | 1 | 1 | 0.25 |
| 5723 | 0 | JP | H01L | H01L0033 | 12 | 14 | 0 | 1 | 0.00 |
| 5724 | 0 | JP | H01L | H01L0029 | 19 | 7 | 0 | 1 | 0.00 |
| 5725 | 0 | JP | H01J | H01J0063 | 10 | 27 | 7 | 1.333333333 | 1.75 |
| 5726 | 0 | DE | H01J | H01J0001 | 23 | 14 | 0 | 1 | 0.00 |
| 5727 | 0 | US | H01L | H01L0021 | 21 | 74 | 0 | 30.25 | 0.00 |
| 5728 | 0 | JP | C23C | C23C0014 | 9 | 19 | 8 | 1 | 1.60 |
| 5729 | 0 | US | H01L | H01L0029 | 25 | 58 | 0 | 30.25 | 0.00 |
| 5730 | 0 | KR | H01L | H01L0021 | 8 | 13 | 0 | 1 | 0.00 |
| 5731 | 0 | JP | H01L | H01L0033 | 10 | 9 | 0 | 1 | 0.00 |
| 5732 | 0 | KR | H01L | H01L0023 | 9 | 27 | 0 | 1.25 | 0.00 |
| 5733 | 0 | US | H01L | H01L0021 | 20 | 39 | 5 | 1 | 0.71 |
| 5734 | 0 | JP | H01S | H01S0003 | 7 | 12 | 0 | 1 | 0.00 |
| 5735 | 0 | JP | F21V | F21V0009 | 21 | 6 | 1 | 1 | 0.50 |
| 5736 | 0 | JP | G03C | G03C0001 | 11 | 5 | 0 | 1 | 0.00 |
| 5737 | 0 | JP | H01L | H01L0033 | 27 | 18 | 1 | 1 | 0.33 |
| 5738 | 0 | US | H01L | H01L0021 | 26 | 46 | 5 | 1 | 1.67 |
| 5739 | 0 | JP | H01L | H01L0033 | 3 | 26 | 0 | 1.5 | 0.00 |
| 5740 | 0 | DE | H05B | H05B0033 | 23 | 8 | 0 | 1 | 0.00 |
| 5741 | 0 | JP | H05B | H05B0033 | 4 | 8 | 3 | 1 | 0.60 |
| 5742 | 0 | JP | G03B | G03B0027 | 62 | 126 | 12 | 3.285714286 | 0.52 |
| 5743 | 0 | TW | H05K | H05K0007 | 15 | 18 | 0 | 1 | 0.00 |
| 5744 | 0 | US | H01L | H01L0021 | 28 | 5 | 0 | 1 | 0.00 |
| 5745 | 0 | US | H01J | H01J0001 | 72 | 155 | 4 | 18.83333333 | 0.04 |
| 5746 | 0 | JP | H01L | H01L0033 | 10 | 57 | 2 | 5.4 | 0.07 |
| 5747 | 0 | DE | H01L | H01L0027 | 14 | 103 | 0 | 3.333333333 | 0.00 |
| 5748 | 0 | JP | H01L | H01L0029 | 4 | 8 | 0 | 1 | 0.00 |
| 5749 | 0 | JP | H01L | H01L0021 | 11 | 19 | 0 | 1 | 0.00 |
| 5750 | 0 | TW | G02B | G02B0006 | 27 | 20 | 1 | 1.2 | 0.17 |
| 5751 | 0 | US | H01S | H01S0003 | 16 | 16 | 1 | 1 | 1.00 |
| 5752 | 0 | TW | H01L | H01L0021 | 13 | 54 | 2 | 1 | 2.00 |
| 5753 | 0 | KR | H01L | H01L0021 | 12 | 6 | 0 | 1 | 0.00 |
| 5754 | 0 | JP | H01L | H01L0033 | 20 | 13 | 0 | 1 | 0.00 |
| 5755 | 0 | TW | H01L | H01L0027 | 8 | 4 | 0 | 1 | 0.00 |
| 5756 | 0 | KR | H01L | H01L0029 | 8 | 7 | 1 | 1.333333333 | 0.25 |
| 5757 | 0 | JP | H01L | H01L0029 | 16 | 15 | 0 | 1 | 0.00 |
| 5758 | 0 | JP | H01J | H01J0001 | 21 | 1 | 1 | 1 | 0.50 |
| 5759 | 0 | JP | H01L | H01L0033 | 6 | 6 | 0 | 1 | 0.00 |
| 5760 | 0 | US | H01L | H01L0033 | 6 | 39 | 4 | 2.333333333 | 0.29 |
| 5761 | 0 | JP | G02F | G02F0002 | 15 | 5 | 0 | 1 | 0.00 |
| 5762 | 0 | US | H05K | H05K0007 | 4 | 18 | 6 | 1 | 3.00 |
| 5763 | 0 | JP | H01L | H01L0029 | 9 | 11 | 0 | 1 | 0.00 |
| 5764 | 0 | KR | H01L | H01L0027 | 20 | 4 | 1 | 1 | 0.25 |
| 5765 | 0 | US | H01L | H01L0023 | 12 | 10 | 0 | 2 | 0.00 |
| 5766 | 0 | TW | H01L | H01L0023 | 21 | 2 | 1 | 1 | 0.50 |
| 5767 | 0 | US | H01L | H01L0021 | 24 | 31 | 0 | 3 | 0.00 |
| 5768 | 0 | TW | G08B | G08B0005 | 20 | 5 | 0 | 1 | 0.00 |
| 5769 | 0 | JP | H01L | H01L0021 | 15 | 17 | 1 | 1 | 0.14 |
| 5770 | 0 | US | G01D | G01D0011 | 20 | 58 | 5 | 1 | 0.83 |
| 5771 | 0 | US | H01L | H01L0021 | 5 | 54 | 0 | 5 | 0.00 |
| 5772 | 0 | US | H01L | H01L0029 | 19 | 6 | 1 | 1.2 | 0.17 |
| 5773 | 0 | JP | H01L | H01L0033 | 18 | 11 | 2 | 1 | 1.00 |
| 5774 | 0 | JP | H01L | H01L0033 | 10 | 2 | 0 | 1 | 0.00 |
| 5775 | 0 | JP | H01L | H01L0027 | 22 | 6 | 0 | 1.25 | 0.00 |
| 5776 | 0 | US | H01L | H01L0021 | 21 | 6 | 2 | 1 | 1.00 |
| 5777 | 0 | KR | H01L | H01L0021 | 10 | 5 | 1 | 1.25 | 0.20 |
| 5778 | 0 | US | H01L | H01L0033 | 10 | 12 | 0 | 1 | 0.00 |
| 5779 | 0 | JP | H01L | H01L0021 | 6 | 20 | 0 | 1.333333333 | 0.00 |
| 5780 | 0 | TW | H01L | H01L0029 | 37 | 12 | 0 | 1 | 0.00 |
| 5781 | 0 | US | H01L | H01L0027 | 20 | 33 | 1 | 1 | 1.00 |
| 5782 | 0 | JP | H01L | H01L0021 | 21 | 15 | 0 | 1 | 0.00 |
| 5783 | 0 | JP | H01L | H01L0023 | 18 | 9 | 0 | 1 | 0.00 |
| 5784 | 0 | JP | C08L | C08L0083 | 7 | 31 | 2 | 1 | 0.67 |
| 5785 | 0 | US | H01L | H01L0029 | 11 | 24 | 3 | 1 | 3.00 |
| 5786 | 0 | DE | H01L | H01L0051 | 24 | 10 | 0 | 1 | 0.00 |
| 5787 | 0 | JP | H01J | H01J0001 | 10 | 3 | 1 | 1.5 | 0.33 |
| 5788 | 0 | US | H01L | H01L0021 | 25 | 3 | 0 | 5 | 0.00 |
| 5789 | 0 | US | H01L | H01L0023 | 14 | 125 | 0 | 18.83333333 | 0.00 |
| 5790 | 0 | US | C09K | C09K0011 | 23 | 119 | 2 | 1.833333333 | 0.18 |
| 5791 | 0 | US | H01L | H01L0021 | 23 | 58 | 1 | 3 | 0.33 |
| 5792 | 0 | KR | H01L | H01L0021 | 5 | 7 | 0 | 1.5 | 0.00 |
| 5793 | 0 | US | H01L | H01L0021 | 46 | 89 | 1 | 1 | 0.20 |
| 5794 | 0 | JP | H01L | H01L0027 | 3 | 16 | 0 | 1 | 0.00 |
| 5795 | 0 | US | H01L | H01L0021 | 22 | 133 | 5 | 3.375 | 0.19 |
| 5796 | 0 | JP | H01L | H01L0023 | 27 | 12 | 0 | 1.333333333 | 0.00 |
| 5797 | 0 | TW | H01L | H01L0023 | 16 | 2 | 0 | 1 | 0.00 |
| 5798 | 0 | JP | F21V | F21V0015 | 6 | 5 | 1 | 1 | 0.33 |
| 5799 | 0 | JP | H01L | H01L0033 | 8 | 93 | 0 | 1.166666667 | 0.00 |
| 5800 | 0 | US | H01L | H01L0033 | 24 | 11 | 1 | 1.8 | 0.11 |
| 5801 | 0 | JP | H01L | H01L0021 | 14 | 27 | 0 | 1.5 | 0.00 |
| 5802 | 0 | JP | H01L | H01L0029 | 19 | 37 | 1 | 1.166666667 | 0.14 |
| 5803 | 0 | TW | H01L | H01L0031 | 17 | 7 | 0 | 1 | 0.00 |
| 5804 | 0 | JP | H01L | H01L0021 | 36 | 46 | 0 | 1.285714286 | 0.00 |
| 5805 | 0 | US | H01L | H01L0027 | 3 | 158 | 0 | 18.83333333 | 0.00 |
| 5806 | 0 | TW | H01L | H01L0023 | 27 | 1 | 1 | 1 | 0.50 |
| 5807 | 0 | JP | H01L | H01L0035 | 11 | 10 | 1 | 1 | 0.25 |
| 5808 | 0 | JP | H05K | H05K0003 | 5 | 27 | 0 | 1.2 | 0.00 |
| 5809 | 0 | US | H01L | H01L0021 | 7 | 30 | 1 | 1.375 | 0.09 |
| 5810 | 0 | KR | H01L | H01L0021 | 17 | 1 | 0 | 1 | 0.00 |
| 5811 | 0 | US | H01L | H01L0031 | 28 | 42 | 0 | 2 | 0.00 |
| 5812 | 0 | TW | H01L | H01L0029 | 6 | 17 | 2 | 1.75 | 0.29 |
| 5813 | 0 | JP | H01L | H01L0029 | 4 | 17 | 0 | 1 | 0.00 |
| 5814 | 0 | TW | H01L | H01L0033 | 6 | 1 | 0 | 1 | 0.00 |
| 5815 | 0 | JP | H01J | H01J0063 | 14 | 32 | 3 | 1.333333333 | 0.38 |
| 5816 | 0 | US | G02B | G02B0006 | 22 | 19 | 0 | 2 | 0.00 |
| 5817 | 0 | KR | H01J | H01J0001 | 19 | 14 | 0 | 1 | 0.00 |
| 5818 | 1 | JP | H05B | H05B0033 | 23 | 186 | 10 | 5 | 0.11 |
| 5819 | 0 | JP | H05K | H05K0007 | 3 | 34 | 2 | 1 | 0.67 |
| 5820 | 0 | US | B23K | B23K0020 | 6 | 7 | 1 | 2 | 0.50 |
| 5821 | 1 | US | F21S | F21S0004 | 6 | 48 | 9 | 4 | 2.25 |
| 5822 | 0 | US | H01L | H01L0021 | 21 | 78 | 1 | 2 | 0.13 |
| 5823 | 0 | DE | H01L | H01L0021 | 23 | 32 | 0 | 1.333333333 | 0.00 |
| 5824 | 0 | JP | H01L | H01L0033 | 9 | 13 | 0 | 1 | 0.00 |
| 5825 | 0 | US | G01N | G01N0021 | 18 | 29 | 0 | 1.428571429 | 0.00 |
| 5826 | 0 | US | H01L | H01L0027 | 14 | 10 | 0 | 1.2 | 0.00 |
| 5827 | 0 | KR | H05B | H05B0033 | 4 | 5 | 0 | 1 | 0.00 |
| 5828 | 0 | US | H01J | H01J0013 | 26 | 19 | 0 | 1.25 | 0.00 |
| 5829 | 0 | US | H05H | H05H0001 | 7 | 31 | 0 | 1.25 | 0.00 |
| 5830 | 0 | US | H01L | H01L0029 | 17 | 45 | 1 | 3 | 0.33 |
| 5831 | 0 | US | H01J | H01J0001 | 23 | 9 | 0 | 1 | 0.00 |
| 5832 | 0 | JP | H01L | H01L0021 | 11 | 23 | 1 | 2 | 0.10 |
| 5833 | 0 | SG | H01L | H01L0031 | 8 | 4 | 0 | 1 | 0.00 |
| 5834 | 0 | TW | H01L | H01L0029 | 3 | 5 | 0 | 1 | 0.00 |
| 5835 | 0 | US | H01L | H01L0033 | 5 | 10 | 0 | 1 | 0.00 |
| 5836 | 0 | JP | H01L | H01L0027 | 18 | 25 | 1 | 1.333333333 | 0.25 |
| 5837 | 0 | JP | H01L | H01L0027 | 5 | 24 | 0 | 1 | 0.00 |
| 5838 | 0 | JP | H01L | H01L0023 | 4 | 13 | 1 | 1 | 0.33 |
| 5839 | 0 | US | H01L | H01L0021 | 26 | 15 | 0 | 1 | 0.00 |
| 5840 | 0 | JP | F21V | F21V0005 | 2 | 20 | 0 | 1.666666667 | 0.00 |
| 5841 | 0 | JP | C09K | C09K0011 | 20 | 9 | 1 | 1 | 0.20 |
| 5842 | 0 | TW | H01L | H01L0021 | 11 | 1 | 0 | 1.5 | 0.00 |
| 5843 | 0 | KR | H01L | H01L0021 | 15 | 15 | 0 | 1 | 0.00 |
| 5844 | 0 | US | H01L | H01L0031 | 21 | 8 | 0 | 2 | 0.00 |
| 5845 | 0 | JP | H01L | H01L0029 | 18 | 1 | 0 | 1 | 0.00 |
| 5846 | 0 | JP | H01L | H01L0033 | 7 | 29 | 0 | 1 | 0.00 |
| 5847 | 0 | JP | H01L | H01L0033 | 4 | 5 | 0 | 1 | 0.00 |
| 5848 | 0 | US | H01J | H01J0001 | 20 | 153 | 0 | 18.83333333 | 0.00 |
| 5849 | 0 | NL | C09K | C09K0011 | 13 | 11 | 2 | 1 | 0.29 |
| 5850 | 0 | US | H01L | H01L0021 | 25 | 34 | 0 | 1.4 | 0.00 |
| 5851 | 0 | TW | H01L | H01L0027 | 19 | 3 | 0 | 1 | 0.00 |
| 5852 | 0 | TW | F21V | F21V0003 | 13 | 19 | 1 | 1 | 0.33 |
| 5853 | 0 | JP | G03F | G03F0007 | 11 | 9 | 0 | 1 | 0.00 |
| 5854 | 0 | SG | H01L | H01L0021 | 6 | 2 | 4 | 1.333333333 | 1.00 |
| 5855 | 0 | US | H01L | H01L0029 | 28 | 38 | 0 | 1 | 0.00 |
| 5856 | 0 | DE | H01L | H01L0027 | 31 | 46 | 0 | 2.285714286 | 0.00 |
| 5857 | 0 | KR | H01L | H01L0029 | 3 | 2 | 3 | 1.25 | 0.60 |
| 5858 | 0 | US | H01L | H01L0021 | 20 | 169 | 0 | 18.83333333 | 0.00 |
| 5859 | 0 | US | H01R | H01R0013 | 15 | 43 | 2 | 1 | 0.50 |
| 5860 | 0 | HK | F21V | F21V0029 | 20 | 7 | 5 | 1 | 5.00 |
| 5861 | 0 | SG | F21V | F21V0029 | 26 | 44 | 1 | 1.25 | 0.20 |
| 5862 | 0 | KR | H01L | H01L0033 | 21 | 36 | 0 | 1 | 0.00 |
| 5863 | 0 | US | F21V | F21V0023 | 19 | 16 | 3 | 1.166666667 | 0.43 |
| 5864 | 0 | JP | H01L | H01L0021 | 12 | 8 | 0 | 1.714285714 | 0.00 |
| 5865 | 0 | US | G03F | G03F0007 | 19 | 42 | 0 | 1 | 0.00 |
| 5866 | 0 | JP | H01L | H01L0021 | 17 | 7 | 0 | 1.5 | 0.00 |
| 5867 | 0 | TW | H01L | H01L0021 | 9 | 6 | 0 | 1 | 0.00 |
| 5868 | 0 | US | H01L | H01L0021 | 19 | 40 | 2 | 2.333333333 | 0.14 |
| 5869 | 0 | JP | H01R | H01R0004 | 8 | 3 | 0 | 1 | 0.00 |
| 5870 | 0 | JP | H01L | H01L0033 | 10 | 10 | 0 | 1 | 0.00 |
| 5871 | 0 | JP | H01L | H01L0023 | 5 | 22 | 0 | 1.5 | 0.00 |
| 5872 | 0 | JP | H01L | H01L0031 | 7 | 7 | 2 | 1 | 1.00 |
| 5873 | 0 | KR | F21V | F21V0029 | 15 | 5 | 6 | 1 | 3.00 |
| 5874 | 0 | US | H01L | H01L0029 | 31 | 12 | 0 | 2 | 0.00 |
| 5875 | 0 | JP | H01L | H01L0021 | 18 | 24 | 0 | 1 | 0.00 |
| 5876 | 0 | JP | C08L | C08L0069 | 16 | 7 | 1 | 1 | 0.17 |
| 5877 | 0 | JP | H01L | H01L0035 | 4 | 4 | 0 | 1.4 | 0.00 |
| 5878 | 0 | JP | H01L | H01L0031 | 18 | 18 | 0 | 1 | 0.00 |
| 5879 | 0 | JP | H01L | H01L0033 | 40 | 167 | 0 | 1.5 | 0.00 |
| 5880 | 0 | US | C12Q | C12Q0001 | 9 | 104 | 2 | 10.83333333 | 0.02 |
| 5881 | 0 | JP | H01L | H01L0023 | 6 | 9 | 0 | 1.5 | 0.00 |
| 5882 | 0 | DE | H01J | H01J0001 | 45 | 4 | 1 | 1 | 0.33 |
| 5883 | 0 | JP | H05B | H05B0033 | 18 | 24 | 1 | 1 | 0.50 |
| 5884 | 0 | JP | F21V | F21V0009 | 17 | 24 | 2 | 1 | 1.00 |
| 5885 | 0 | DE | H01L | H01L0021 | 17 | 34 | 2 | 1.5 | 0.17 |
| 5886 | 0 | US | H01L | H01L0025 | 24 | 110 | 0 | 4 | 0.00 |
| 5887 | 0 | KR | B29C | B29C0043 | 14 | 19 | 0 | 1.25 | 0.00 |
| 5888 | 0 | JP | H01L | H01L0021 | 5 | 5 | 0 | 2 | 0.00 |
| 5889 | 0 | US | H05B | H05B0033 | 17 | 39 | 0 | 1 | 0.00 |
| 5890 | 0 | US | H01L | H01L0051 | 12 | 86 | 0 | 8 | 0.00 |
| 5891 | 0 | DE | H01L | H01L0033 | 46 | 53 | 1 | 1 | 0.13 |
| 5892 | 0 | KR | G02F | G02F0001 | 9 | 18 | 1 | 1.5 | 0.17 |
| 5893 | 0 | US | G03F | G03F0009 | 18 | 4 | 0 | 1 | 0.00 |
| 5894 | 0 | JP | H01J | H01J0001 | 13 | 37 | 1 | 1 | 0.25 |
| 5895 | 0 | IT | H01L | H01L0023 | 151 | 12 | 0 | 1.142857143 | 0.00 |
| 5896 | 0 | JP | H03H | H03H0009 | 8 | 15 | 2 | 1 | 0.67 |
| 5897 | 0 | TW | H01L | H01L0021 | 20 | 39 | 3 | 2.333333333 | 0.21 |
| 5898 | 0 | KR | H01L | H01L0051 | 41 | 75 | 4 | 4.333333333 | 0.15 |
| 5899 | 0 | TW | H01L | H01L0033 | 13 | 5 | 1 | 1 | 0.50 |
| 5900 | 0 | JP | H01L | H01L0033 | 16 | 2 | 2 | 1 | 0.67 |
| 5901 | 0 | US | H01L | H01L0033 | 24 | 53 | 5 | 1 | 2.50 |
| 5902 | 0 | US | F21S | F21S0013 | 21 | 169 | 2 | 1 | 0.33 |
| 5903 | 0 | US | G03F | G03F0007 | 20 | 23 | 0 | 1 | 0.00 |
| 5904 | 0 | JP | H01L | H01L0021 | 14 | 10 | 0 | 1.25 | 0.00 |
| 5905 | 0 | KR | H01L | H01L0021 | 11 | 25 | 0 | 1.666666667 | 0.00 |
| 5906 | 0 | JP | H01L | H01L0021 | 12 | 22 | 0 | 1.5 | 0.00 |
| 5907 | 0 | KR | H01L | H01L0033 | 17 | 4 | 1 | 1 | 0.25 |
| 5908 | 0 | JP | G02F | G02F0001 | 19 | 7 | 0 | 1 | 0.00 |
| 5909 | 0 | US | H01L | H01L0021 | 11 | 7 | 1 | 2 | 0.50 |
| 5910 | 0 | KR | H01L | H01L0021 | 14 | 23 | 0 | 1.25 | 0.00 |
| 5911 | 0 | KR | H01L | H01L0025 | 10 | 60 | 2 | 5.777777778 | 0.04 |
| 5912 | 0 | JP | H01L | H01L0029 | 16 | 53 | 1 | 2 | 0.25 |
| 5913 | 0 | KR | H01L | H01L0033 | 36 | 75 | 1 | 4.333333333 | 0.04 |
| 5914 | 0 | JP | H01J | H01J0001 | 7 | 11 | 2 | 1 | 0.50 |
| 5915 | 0 | US | H01L | H01L0021 | 15 | 45 | 1 | 3.666666667 | 0.09 |
| 5916 | 0 | KR | F21V | F21V0005 | 31 | 36 | 6 | 1.2 | 1.00 |
| 5917 | 0 | JP | H01J | H01J0001 | 38 | 167 | 1 | 2.714285714 | 0.05 |
| 5918 | 0 | KR | H01L | H01L0021 | 20 | 4 | 1 | 1 | 0.33 |
| 5919 | 0 | TW | H01L | H01L0033 | 2 | 3 | 0 | 1 | 0.00 |
| 5920 | 0 | US | G03B | G03B0021 | 25 | 69 | 1 | 1 | 0.17 |
| 5921 | 0 | US | H01L | H01L0029 | 10 | 72 | 1 | 2.333333333 | 0.07 |
| 5922 | 0 | JP | H01L | H01L0033 | 21 | 9 | 0 | 1 | 0.00 |
| 5923 | 0 | JP | H01L | H01L0029 | 18 | 19 | 0 | 1 | 0.00 |
| 5924 | 0 | TW | H01L | H01L0029 | 11 | 2 | 1 | 4 | 0.13 |
| 5925 | 0 | KR | H01C | H01C0007 | 32 | 76 | 1 | 4.333333333 | 0.04 |
| 5926 | 0 | JP | H01L | H01L0021 | 13 | 4 | 0 | 1.5 | 0.00 |
| 5927 | 0 | TW | H01L | H01L0021 | 18 | 10 | 0 | 1 | 0.00 |
| 5928 | 0 | JP | H01L | H01L0021 | 8 | 3 | 3 | 2 | 0.75 |
| 5929 | 0 | JP | H01L | H01L0021 | 9 | 35 | 1 | 1 | 0.25 |
| 5930 | 0 | US | G02B | G02B0006 | 20 | 15 | 1 | 1.5 | 0.33 |
| 5931 | 0 | JP | H01L | H01L0031 | 17 | 18 | 4 | 2.5 | 0.80 |
| 5932 | 0 | KR | F21V | F21V0005 | 7 | 4 | 0 | 1.666666667 | 0.00 |
| 5933 | 0 | US | H01L | H01L0029 | 15 | 470 | 1 | 1.166666667 | 0.14 |
| 5934 | 0 | US | H01J | H01J0001 | 18 | 17 | 0 | 1 | 0.00 |
| 5935 | 0 | JP | H01L | H01L0027 | 22 | 3 | 1 | 1 | 0.33 |
| 5936 | 0 | US | H01L | H01L0021 | 14 | 43 | 0 | 4 | 0.00 |
| 5937 | 0 | KR | H01L | H01L0021 | 21 | 48 | 2 | 2 | 0.50 |
| 5938 | 0 | TW | H01L | H01L0033 | 10 | 3 | 2 | 1 | 1.00 |
| 5939 | 0 | JP | H01L | H01L0031 | 19 | 6 | 4 | 1 | 1.33 |
| 5940 | 0 | US | H01L | H01L0021 | 22 | 6 | 0 | 1 | 0.00 |
| 5941 | 0 | JP | H01L | H01L0023 | 3 | 7 | 0 | 2 | 0.00 |
| 5942 | 0 | JP | H01L | H01L0023 | 16 | 4 | 0 | 1 | 0.00 |
| 5943 | 0 | US | H01L | H01L0051 | 5 | 67 | 4 | 1.875 | 0.27 |
| 5944 | 0 | US | H01S | H01S0005 | 33 | 44 | 1 | 1 | 0.20 |
| 5945 | 0 | JP | H01L | H01L0021 | 4 | 28 | 2 | 1 | 0.67 |
| 5946 | 0 | JP | H01L | H01L0021 | 13 | 18 | 0 | 1 | 0.00 |
| 5947 | 0 | US | H01L | H01L0021 | 20 | 36 | 0 | 3 | 0.00 |
| 5948 | 0 | TW | H01L | H01L0027 | 17 | 13 | 2 | 3 | 0.67 |
| 5949 | 1 | TW | H01L | H01L0023 | 22 | 17 | 0 | 1 | 0.00 |
| 5950 | 0 | JP | H01L | H01L0023 | 2 | 19 | 0 | 1.5 | 0.00 |
| 5951 | 0 | TW | H01L | H01L0023 | 5 | 5 | 0 | 1 | 0.00 |
| 5952 | 0 | DE | G09G | G09G0003 | 18 | 11 | 0 | 1 | 0.00 |
| 5953 | 0 | KR | F21V | F21V0029 | 9 | 4 | 0 | 1 | 0.00 |
| 5954 | 0 | US | F21V | F21V0029 | 24 | 8 | 3 | 1 | 0.75 |
| 5955 | 0 | US | H01L | H01L0033 | 5 | 27 | 9 | 1 | 1.50 |
| 5956 | 0 | US | H01L | H01L0021 | 19 | 10 | 0 | 1 | 0.00 |
| 5957 | 0 | KR | H01L | H01L0021 | 41 | 75 | 1 | 4.333333333 | 0.04 |
| 5958 | 0 | TW | H01L | H01L0021 | 14 | 6 | 0 | 1 | 0.00 |
| 5959 | 0 | TW | H01L | H01L0021 | 6 | 3 | 0 | 1 | 0.00 |
| 5960 | 0 | TW | H01L | H01L0027 | 12 | 3 | 0 | 1 | 0.00 |
| 5961 | 0 | TW | H01L | H01L0033 | 17 | 12 | 0 | 1 | 0.00 |
| 5962 | 0 | TW | H01L | H01L0027 | 7 | 27 | 0 | 1 | 0.00 |
| 5963 | 0 | JP | H01L | H01L0029 | 14 | 6 | 1 | 1 | 0.50 |
| 5964 | 0 | JP | H01L | H01L0033 | 3 | 61 | 0 | 3.75 | 0.00 |
| 5965 | 0 | US | H01L | H01L0033 | 4 | 32 | 1 | 1 | 0.33 |
| 5966 | 0 | KR | C09K | C09K0011 | 8 | 13 | 0 | 1 | 0.00 |
| 5967 | 0 | JP | H01L | H01L0021 | 28 | 68 | 0 | 1.5 | 0.00 |
| 5968 | 0 | JP | C08L | C08L0083 | 6 | 7 | 0 | 1 | 0.00 |
| 5969 | 0 | KR | H01L | H01L0031 | 4 | 8 | 1 | 1.5 | 0.17 |
| 5970 | 0 | KR | H01L | H01L0027 | 3 | 20 | 1 | 1.25 | 0.20 |
| 5971 | 0 | US | H01L | H01L0029 | 13 | 49 | 2 | 1.6 | 0.25 |
| 5972 | 0 | US | H01L | H01L0023 | 19 | 14 | 0 | 1 | 0.00 |
| 5973 | 0 | KR | H01L | H01L0033 | 20 | 6 | 0 | 3 | 0.00 |
| 5974 | 0 | JP | H01L | H01L0029 | 6 | 23 | 0 | 1.166666667 | 0.00 |
| 5975 | 0 | US | F21V | F21V0009 | 18 | 11 | 0 | 1 | 0.00 |
| 5976 | 0 | US | F21V | F21V0021 | 9 | 8 | 7 | 1 | 0.78 |
| 5977 | 0 | US | F21V | F21V0033 | 18 | 22 | 0 | 1.166666667 | 0.00 |
| 5978 | 0 | DE | H01J | H01J0009 | 22 | 23 | 0 | 2 | 0.00 |
| 5979 | 0 | JP | H01L | H01L0021 | 6 | 25 | 1 | 8.866666667 | 0.01 |
| 5980 | 0 | US | H01L | H01L0033 | 36 | 23 | 0 | 1 | 0.00 |
| 5981 | 0 | US | H01L | H01L0029 | 28 | 15 | 1 | 1.857142857 | 0.08 |
| 5982 | 0 | JP | H01L | H01L0029 | 16 | 17 | 0 | 1 | 0.00 |
| 5983 | 0 | JP | H01L | H01L0033 | 14 | 12 | 0 | 1 | 0.00 |
| 5984 | 0 | KR | H01L | H01L0021 | 10 | 6 | 2 | 1.333333333 | 0.25 |
| 5985 | 0 | SI | H01L | H01L0021 | 16 | 16 | 0 | 1 | 0.00 |
| 5986 | 0 | TW | H01L | H01L0021 | 23 | 3 | 1 | 3 | 0.33 |
| 5987 | 0 | US | H01L | H01L0029 | 27 | 159 | 0 | 18.83333333 | 0.00 |
| 5988 | 0 | GB | H01L | H01L0021 | 13 | 14 | 2 | 1 | 0.40 |
| 5989 | 0 | JP | H01L | H01L0051 | 13 | 26 | 0 | 3 | 0.00 |
| 5990 | 0 | KR | H01L | H01L0029 | 7 | 13 | 0 | 1.5 | 0.00 |
| 5991 | 0 | TW | H01L | H01L0029 | 10 | 5 | 1 | 1 | 0.50 |
| 5992 | 0 | JP | H01L | H01L0033 | 13 | 41 | 1 | 1.2 | 0.17 |
| 5993 | 0 | TW | H01L | H01L0031 | 20 | 18 | 0 | 1 | 0.00 |
| 5994 | 0 | TW | H01L | H01L0029 | 6 | 20 | 2 | 1 | 1.00 |
| 5995 | 0 | JP | H01L | H01L0023 | 5 | 10 | 0 | 1 | 0.00 |
| 5996 | 0 | TW | H05B | H05B0033 | 3 | 7 | 0 | 2 | 0.00 |
| 5997 | 0 | US | H05B | H05B0037 | 32 | 573 | 1 | 23.28571429 | 0.00 |
| 5998 | 0 | US | F21V | F21V0007 | 7 | 94 | 0 | 2.5 | 0.00 |
| 5999 | 0 | JP | C09K | C09K0011 | 19 | 12 | 0 | 2.5 | 0.00 |
| 6000 | 0 | KR | H01L | H01L0021 | 3 | 7 | 0 | 1 | 0.00 |
| 6001 | 0 | JP | H01L | H01L0029 | 37 | 14 | 1 | 1.166666667 | 0.14 |
| 6002 | 0 | KR | H01L | H01L0029 | 8 | 12 | 1 | 1 | 0.50 |
| 6003 | 0 | JP | H01L | H01L0023 | 9 | 11 | 0 | 1 | 0.00 |
| 6004 | 0 | US | H01L | H01L0029 | 5 | 10 | 1 | 1 | 0.50 |
| 6005 | 0 | JP | H01L | H01L0027 | 15 | 22 | 0 | 1 | 0.00 |
| 6006 | 0 | JP | H01L | H01L0023 | 13 | 12 | 0 | 1 | 0.00 |
| 6007 | 0 | US | H01L | H01L0027 | 14 | 31 | 2 | 1.333333333 | 0.25 |
| 6008 | 0 | JP | H01L | H01L0051 | 11 | 29 | 1 | 1 | 0.50 |
| 6009 | 0 | SG | F21V | F21V0008 | 17 | 5 | 3 | 1.5 | 1.00 |
| 6010 | 0 | JP | C09K | C09K0011 | 20 | 25 | 0 | 1 | 0.00 |
| 6011 | 0 | JP | H01L | H01L0021 | 8 | 9 | 0 | 1 | 0.00 |
| 6012 | 0 | JP | H01L | H01L0027 | 2 | 28 | 0 | 1 | 0.00 |
| 6013 | 0 | JP | C09K | C09K0011 | 8 | 21 | 0 | 1 | 0.00 |
| 6014 | 0 | KR | H01J | H01J0001 | 12 | 9 | 0 | 1 | 0.00 |
| 6015 | 0 | NL | H01L | H01L0033 | 8 | 6 | 0 | 1 | 0.00 |
| 6016 | 1 | US | H01L | H01L0021 | 13 | 109 | 0 | 8.8 | 0.00 |
| 6017 | 0 | US | H01L | H01L0021 | 29 | 28 | 0 | 1.25 | 0.00 |
| 6018 | 0 | KR | H01L | H01L0033 | 5 | 7 | 0 | 1.333333333 | 0.00 |
| 6019 | 0 | SG | H01L | H01L0023 | 13 | 12 | 0 | 1.25 | 0.00 |
| 6020 | 0 | JP | H01S | H01S0005 | 18 | 16 | 0 | 1.5 | 0.00 |
| 6021 | 0 | US | F21V | F21V0033 | 56 | 189 | 4 | 1.166666667 | 0.57 |
| 6022 | 0 | JP | F21V | F21V0007 | 23 | 8 | 0 | 1.5 | 0.00 |
| 6023 | 0 | TW | H01L | H01L0021 | 13 | 10 | 1 | 2 | 0.25 |
| 6024 | 0 | JP | H01L | H01L0021 | 16 | 12 | 0 | 1.333333333 | 0.00 |
| 6025 | 0 | KR | H01L | H01L0025 | 6 | 62 | 1 | 5.777777778 | 0.02 |
| 6026 | 0 | US | H01L | H01L0031 | 16 | 84 | 1 | 5 | 0.20 |
| 6027 | 0 | JP | H01L | H01L0031 | 1 | 67 | 1 | 5.4 | 0.04 |
| 6028 | 0 | JP | H01L | H01L0029 | 20 | 16 | 3 | 1 | 1.50 |
| 6029 | 0 | TW | H01L | H01L0033 | 10 | 7 | 0 | 1 | 0.00 |
| 6030 | 0 | US | H01L | H01L0023 | 27 | 497 | 1 | 1.333333333 | 0.25 |
| 6031 | 0 | JP | H01J | H01J0001 | 48 | 18 | 0 | 1.2 | 0.00 |
| 6032 | 0 | JP | F21V | F21V0029 | 20 | 9 | 0 | 1 | 0.00 |
| 6033 | 0 | US | F21V | F21V0009 | 3 | 16 | 0 | 1 | 0.00 |
| 6034 | 0 | JP | C09K | C09K0011 | 6 | 4 | 0 | 1 | 0.00 |
| 6035 | 0 | JP | G02F | G02F0001 | 19 | 22 | 0 | 1.25 | 0.00 |
| 6036 | 0 | JP | H01L | H01L0033 | 5 | 161 | 0 | 4.5 | 0.00 |
| 6037 | 0 | US | F21S | F21S0004 | 27 | 11 | 1 | 5.571428571 | 0.03 |
| 6038 | 0 | DE | F21V | F21V0005 | 23 | 17 | 1 | 1 | 0.14 |
| 6039 | 0 | CA | B05D | B05D0005 | 45 | 24 | 0 | 1 | 0.00 |
| 6040 | 0 | US | H01L | H01L0051 | 19 | 19 | 0 | 1 | 0.00 |
| 6041 | 0 | JP | H01L | H01L0021 | 25 | 68 | 2 | 1.142857143 | 0.25 |
| 6042 | 0 | KR | H01L | H01L0027 | 13 | 13 | 0 | 1.666666667 | 0.00 |
| 6043 | 0 | JP | H01L | H01L0033 | 20 | 11 | 1 | 1.25 | 0.20 |
| 6044 | 0 | JP | H01L | H01L0029 | 20 | 62 | 1 | 1.6 | 0.13 |
| 6045 | 0 | TW | H01L | H01L0023 | 4 | 10 | 1 | 2 | 0.50 |
| 6046 | 0 | JP | G02F | G02F0001 | 28 | 4 | 0 | 1.5 | 0.00 |
| 6047 | 0 | US | F21K | F21K0099 | 33 | 6 | 3 | 1 | 0.60 |
| 6048 | 0 | TW | H01L | H01L0021 | 9 | 3 | 0 | 2 | 0.00 |
| 6049 | 0 | US | H01L | H01L0029 | 24 | 3 | 0 | 1.2 | 0.00 |
| 6050 | 0 | KR | H01L | H01L0023 | 21 | 19 | 2 | 1.333333333 | 0.50 |
| 6051 | 0 | DE | H01L | H01L0021 | 38 | 8 | 1 | 1.5 | 0.33 |
| 6052 | 0 | JP | H01L | H01L0023 | 2 | 7 | 0 | 1 | 0.00 |
| 6053 | 0 | JP | B05D | B05D0005 | 16 | 105 | 0 | 1.5 | 0.00 |
| 6054 | 0 | BN | H01L | H01L0021 | 16 | 1 | 0 | 2 | 0.00 |
| 6055 | 0 | US | H01L | H01L0029 | 36 | 19 | 0 | 3 | 0.00 |
| 6056 | 0 | JP | H01L | H01L0033 | 13 | 13 | 0 | 1.2 | 0.00 |
| 6057 | 0 | US | H01L | H01L0051 | 8 | 15 | 0 | 1 | 0.00 |
| 6058 | 0 | US | H05B | H05B0037 | 24 | 12 | 0 | 1 | 0.00 |
| 6059 | 0 | NL | F21V | F21V0005 | 11 | 8 | 0 | 1 | 0.00 |
| 6060 | 0 | US | H05B | H05B0033 | 15 | 10 | 2 | 2 | 0.50 |
| 6061 | 0 | US | H01L | H01L0029 | 26 | 7 | 0 | 1 | 0.00 |
| 6062 | 0 | TW | H01L | H01L0029 | 2 | 1 | 0 | 2 | 0.00 |
| 6063 | 0 | NL | H01L | H01L0029 | 21 | 3 | 0 | 1 | 0.00 |
| 6064 | 0 | SG | H01L | H01L0027 | 17 | 1 | 0 | 1 | 0.00 |
| 6065 | 0 | US | H01L | H01L0023 | 24 | 7 | 0 | 1 | 0.00 |
| 6066 | 0 | MI | F21S | F21S0008 | 18 | 7 | 0 | 1 | 0.00 |
| 6067 | 0 | US | F21V | F21V0033 | 13 | 118 | 5 | 5 | 1.00 |
| 6068 | 0 | TW | H01L | H01L0027 | 8 | 12 | 0 | 1.5 | 0.00 |
| 6069 | 0 | TW | H01L | H01L0027 | 6 | 1 | 0 | 1 | 0.00 |
| 6070 | 0 | JP | H01L | H01L0029 | 6 | 33 | 0 | 1.333333333 | 0.00 |
| 6071 | 0 | TW | H01L | H01L0029 | 21 | 1 | 0 | 3 | 0.00 |
| 6072 | 0 | US | H01J | H01J0009 | 8 | 19 | 0 | 1 | 0.00 |
| 6073 | 0 | KR | H01L | H01L0021 | 12 | 8 | 0 | 1 | 0.00 |
| 6074 | 0 | US | H01L | H01L0023 | 18 | 8 | 0 | 1 | 0.00 |
| 6075 | 0 | JP | H01L | H01L0023 | 21 | 12 | 0 | 1 | 0.00 |
| 6076 | 0 | JP | H01J | H01J0029 | 19 | 5 | 0 | 1 | 0.00 |
| 6077 | 0 | SG | H05K | H05K0007 | 19 | 6 | 0 | 1 | 0.00 |
| 6078 | 0 | NL | G06K | G06K0009 | 31 | 20 | 0 | 1 | 0.00 |
| 6079 | 0 | US | H01L | H01L0031 | 13 | 3 | 0 | 2 | 0.00 |
| 6080 | 0 | US | H01L | H01L0021 | 10 | 53 | 0 | 2 | 0.00 |
| 6081 | 0 | US | H01L | H01L0021 | 12 | 31 | 0 | 1.285714286 | 0.00 |
| 6082 | 0 | TW | H01L | H01L0021 | 7 | 7 | 0 | 2 | 0.00 |
| 6083 | 0 | CN | H01L | H01L0033 | 12 | 9 | 0 | 1.428571429 | 0.00 |
| 6084 | 0 | JP | H01L | H01L0033 | 27 | 3 | 0 | 1 | 0.00 |
| 6085 | 0 | DE | H01L | H01L0033 | 22 | 18 | 1 | 2 | 0.17 |
| 6086 | 0 | KR | H01L | H01L0033 | 18 | 4 | 0 | 1.333333333 | 0.00 |
| 6087 | 0 | DE | H01L | H01L0033 | 11 | 31 | 0 | 1 | 0.00 |
| 6088 | 0 | TW | H01L | H01L0027 | 11 | 6 | 0 | 1 | 0.00 |
| 6089 | 0 | US | B32B | B32B0009 | 28 | 103 | 1 | 1.333333333 | 0.13 |
| 6090 | 0 | NL | C09K | C09K0011 | 6 | 5 | 0 | 1.2 | 0.00 |
| 6091 | 0 | US | F21S | F21S0008 | 49 | 9 | 1 | 1 | 1.00 |
| 6092 | 0 | JP | H01L | H01L0021 | 18 | 27 | 0 | 1.666666667 | 0.00 |
| 6093 | 0 | TW | H01L | H01L0033 | 16 | 12 | 1 | 3 | 0.33 |
| 6094 | 0 | JP | H01L | H01L0033 | 12 | 23 | 1 | 1 | 0.50 |
| 6095 | 0 | BN | H01L | H01L0033 | 18 | 2 | 0 | 2 | 0.00 |
| 6096 | 0 | KR | H01L | H01L0033 | 10 | 66 | 1 | 5.777777778 | 0.02 |
| 6097 | 0 | US | H01L | H01L0029 | 22 | 72 | 2 | 1.25 | 0.40 |
| 6098 | 0 | JP | H01L | H01L0033 | 20 | 8 | 0 | 1 | 0.00 |
| 6099 | 0 | JP | H01L | H01L0033 | 8 | 33 | 0 | 1 | 0.00 |
| 6100 | 0 | KR | H01J | H01J0001 | 26 | 23 | 0 | 1 | 0.00 |
| 6101 | 0 | US | H01L | H01L0021 | 16 | 13 | 0 | 1 | 0.00 |
| 6102 | 0 | JP | C30B | C30B0025 | 15 | 31 | 0 | 1 | 0.00 |
| 6103 | 0 | US | C09K | C09K0011 | 12 | 4 | 2 | 5 | 0.04 |
| 6104 | 0 | JP | H01L | H01L0029 | 7 | 11 | 1 | 1 | 0.25 |
| 6105 | 0 | KR | H01L | H01L0027 | 10 | 9 | 0 | 1 | 0.00 |
| 6106 | 0 | KR | H01L | H01L0029 | 37 | 38 | 1 | 7 | 0.14 |
| 6107 | 0 | JP | H01L | H01L0033 | 4 | 56 | 1 | 5.4 | 0.04 |
| 6108 | 0 | JP | H01L | H01L0033 | 16 | 17 | 0 | 1 | 0.00 |
| 6109 | 0 | KR | H01L | H01L0027 | 19 | 10 | 2 | 2.4 | 0.17 |
| 6110 | 0 | SG | H01L | H01L0027 | 17 | 3 | 0 | 1 | 0.00 |
| 6111 | 0 | BN | H01L | H01L0033 | 15 | 1 | 0 | 2 | 0.00 |
| 6112 | 0 | US | H01L | H01L0027 | 42 | 4 | 0 | 1 | 0.00 |
| 6113 | 0 | US | H01L | H01L0023 | 12 | 10 | 0 | 1 | 0.00 |
| 6114 | 0 | KR | H01L | H01L0033 | 5 | 1 | 0 | 1 | 0.00 |
| 6115 | 0 | KR | H05B | H05B0033 | 15 | 3 | 14 | 1 | 7.00 |
| 6116 | 0 | US | H01J | H01J0001 | 19 | 5 | 0 | 2 | 0.00 |
| 6117 | 0 | US | H01L | H01L0033 | 24 | 33 | 0 | 1 | 0.00 |
| 6118 | 0 | US | H01L | H01L0021 | 95 | 14 | 0 | 2 | 0.00 |
| 6119 | 0 | TW | H01L | H01L0033 | 20 | 10 | 1 | 1 | 1.00 |
| 6120 | 0 | JP | H01L | H01L0033 | 10 | 6 | 0 | 1 | 0.00 |
| 6121 | 0 | US | H01L | H01L0029 | 32 | 27 | 0 | 1.2 | 0.00 |
| 6122 | 0 | JP | H01L | H01L0033 | 16 | 4 | 0 | 1 | 0.00 |
| 6123 | 0 | JP | H01L | H01L0033 | 11 | 9 | 1 | 1 | 0.50 |
| 6124 | 0 | JP | G09F | G09F0009 | 19 | 7 | 0 | 1 | 0.00 |
| 6125 | 0 | JP | G09G | G09G0003 | 17 | 21 | 0 | 1 | 0.00 |
| 6126 | 0 | JP | G11B | G11B0007 | 19 | 8 | 0 | 5 | 0.00 |
| 6127 | 0 | JP | H01S | H01S0003 | 14 | 9 | 0 | 1 | 0.00 |
| 6128 | 0 | KR | H01S | H01S0005 | 12 | 1 | 0 | 1 | 0.00 |
| 6129 | 0 | JP | H01L | H01L0033 | 12 | 23 | 1 | 2 | 0.25 |
| 6130 | 0 | GB | H01L | H01L0051 | 28 | 38 | 0 | 1.166666667 | 0.00 |
| 6131 | 0 | TW | H01L | H01L0033 | 9 | 3 | 0 | 1.75 | 0.00 |
| 6132 | 0 | TW | F21V | F21V0011 | 20 | 1 | 1 | 1 | 1.00 |
| 6133 | 0 | US | H01L | H01L0021 | 7 | 38 | 0 | 2.333333333 | 0.00 |
| 6134 | 0 | JP | H01L | H01L0029 | 16 | 50 | 1 | 1 | 0.33 |
| 6135 | 0 | JP | H01L | H01L0051 | 18 | 58 | 1 | 1.4 | 0.14 |
| 6136 | 0 | US | H01L | H01L0033 | 15 | 38 | 0 | 1 | 0.00 |
| 6137 | 0 | JP | H01L | H01L0033 | 5 | 21 | 0 | 1 | 0.00 |
| 6138 | 0 | US | F21V | F21V0009 | 29 | 6 | 0 | 1 | 0.00 |
| 6139 | 0 | TW | H01L | H01L0031 | 17 | 6 | 0 | 1 | 0.00 |
| 6140 | 0 | DE | H01L | H01L0033 | 27 | 19 | 0 | 1.666666667 | 0.00 |
| 6141 | 0 | JP | H01L | H01L0033 | 3 | 41 | 0 | 2 | 0.00 |
| 6142 | 0 | KR | H01L | H01L0029 | 43 | 7 | 0 | 1.333333333 | 0.00 |
| 6143 | 0 | TW | H01L | H01L0021 | 8 | 28 | 0 | 4 | 0.00 |
| 6144 | 0 | JP | H01J | H01J0001 | 20 | 17 | 0 | 1 | 0.00 |
| 6145 | 0 | US | H01L | H01L0023 | 19 | 17 | 0 | 1 | 0.00 |
| 6146 | 0 | KR | H01L | H01L0033 | 13 | 81 | 1 | 5.777777778 | 0.02 |
| 6147 | 0 | SG | H01L | H01L0033 | 7 | 31 | 0 | 1.5 | 0.00 |
| 6148 | 0 | JP | H01L | H01L0023 | 13 | 13 | 0 | 1 | 0.00 |
| 6149 | 0 | SG | H01L | H01L0023 | 14 | 8 | 1 | 2 | 0.50 |
| 6150 | 0 | TW | H01J | H01J0001 | 15 | 11 | 2 | 1 | 1.00 |
| 6151 | 0 | US | H01J | H01J0017 | 9 | 6 | 0 | 1 | 0.00 |
| 6152 | 0 | JP | H01L | H01L0021 | 16 | 7 | 0 | 1.25 | 0.00 |
| 6153 | 0 | KR | H01L | H01L0021 | 20 | 12 | 0 | 1.25 | 0.00 |
| 6154 | 0 | US | H01J | H01J0009 | 11 | 29 | 0 | 1.285714286 | 0.00 |
| 6155 | 0 | JP | C08G | C08G0059 | 7 | 16 | 0 | 1 | 0.00 |
| 6156 | 0 | TW | H01L | H01L0029 | 17 | 6 | 1 | 1 | 1.00 |
| 6157 | 0 | TW | H01L | H01L0029 | 5 | 3 | 0 | 2 | 0.00 |
| 6158 | 0 | JP | H01L | H01L0033 | 16 | 22 | 0 | 1 | 0.00 |
| 6159 | 0 | JP | H01L | H01L0027 | 15 | 11 | 0 | 1.5 | 0.00 |
| 6160 | 0 | JP | H01L | H01L0051 | 10 | 13 | 0 | 1.5 | 0.00 |
| 6161 | 0 | US | G09G | G09G0003 | 30 | 7 | 0 | 1 | 0.00 |
| 6162 | 0 | JP | H01S | H01S0005 | 8 | 6 | 0 | 1 | 0.00 |
| 6163 | 0 | TW | G02B | G02B0006 | 8 | 21 | 0 | 1.2 | 0.00 |
| 6164 | 0 | DE | H05K | H05K0003 | 22 | 18 | 0 | 1 | 0.00 |
| 6165 | 0 | KR | H01L | H01L0021 | 10 | 24 | 0 | 1.666666667 | 0.00 |
| 6166 | 0 | JP | C30B | C30B0029 | 2 | 29 | 2 | 1.5 | 0.67 |
| 6167 | 0 | JP | H01L | H01L0027 | 44 | 24 | 0 | 1.666666667 | 0.00 |
| 6168 | 0 | JP | H01L | H01L0033 | 14 | 25 | 0 | 1 | 0.00 |
| 6169 | 0 | TW | H01L | H01L0033 | 26 | 12 | 0 | 1 | 0.00 |
| 6170 | 0 | JP | H01L | H01L0023 | 13 | 30 | 0 | 1 | 0.00 |
| 6171 | 0 | JP | H05K | H05K0007 | 13 | 5 | 0 | 1 | 0.00 |
| 6172 | 0 | SG | F21V | F21V0029 | 16 | 9 | 1 | 1 | 1.00 |
| 6173 | 0 | DE | B32B | B32B0001 | 17 | 23 | 0 | 1 | 0.00 |
| 6174 | 0 | KR | H01L | H01L0021 | 20 | 15 | 0 | 1.25 | 0.00 |
| 6175 | 0 | TW | H01L | H01L0027 | 10 | 11 | 2 | 1.5 | 0.67 |
| 6176 | 0 | JP | H01L | H01L0033 | 15 | 16 | 1 | 1 | 0.50 |
| 6177 | 0 | SG | H01L | H01L0027 | 20 | 7 | 0 | 1 | 0.00 |
| 6178 | 0 | SG | H01L | H01L0029 | 9 | 5 | 0 | 1 | 0.00 |
| 6179 | 0 | US | C09K | C09K0003 | 20 | 60 | 0 | 1 | 0.00 |
| 6180 | 0 | KR | H01L | H01L0027 | 4 | 5 | 0 | 1.5 | 0.00 |
| 6181 | 0 | JP | H01L | H01L0033 | 10 | 12 | 1 | 1 | 0.50 |
| 6182 | 0 | KR | H01L | H01L0033 | 18 | 2 | 2 | 1 | 0.67 |
| 6183 | 0 | US | F21V | F21V0029 | 7 | 6 | 0 | 1 | 0.00 |
| 6184 | 0 | TW | C09K | C09K0011 | 3 | 6 | 0 | 1 | 0.00 |
| 6185 | 0 | US | H01L | H01L0025 | 26 | 115 | 1 | 4 | 0.04 |
| 6186 | 0 | JP | H01L | H01L0021 | 2 | 215 | 0 | 5 | 0.00 |
| 6187 | 0 | US | H01L | H01L0021 | 18 | 13 | 1 | 1 | 0.17 |
| 6188 | 0 | JP | H01L | H01L0021 | 6 | 13 | 1 | 1.666666667 | 0.20 |
| 6189 | 0 | TW | H01L | H01L0021 | 15 | 8 | 0 | 2 | 0.00 |
| 6190 | 0 | JP | H01L | H01L0033 | 8 | 7 | 0 | 1 | 0.00 |
| 6191 | 0 | US | H01L | H01L0023 | 4 | 18 | 0 | 1 | 0.00 |
| 6192 | 0 | TW | H01L | H01L0029 | 19 | 1 | 1 | 1 | 1.00 |
| 6193 | 0 | US | F21V | F21V0003 | 20 | 35 | 0 | 1 | 0.00 |
| 6194 | 0 | JP | H01L | H01L0027 | 8 | 23 | 1 | 1 | 0.33 |
| 6195 | 0 | DE | H01L | H01L0033 | 12 | 21 | 0 | 1 | 0.00 |
| 6196 | 0 | KR | H01L | H01L0033 | 2 | 11 | 1 | 1.666666667 | 0.20 |
| 6197 | 0 | US | H01L | H01L0023 | 19 | 2 | 1 | 1 | 0.20 |
| 6198 | 0 | US | H01L | H01L0023 | 17 | 29 | 0 | 3 | 0.00 |
| 6199 | 0 | JP | H01J | H01J0001 | 32 | 111 | 0 | 4.5 | 0.00 |
| 6200 | 0 | KR | H01J | H01J0001 | 17 | 10 | 0 | 1 | 0.00 |
| 6201 | 0 | US | H01J | H01J0001 | 16 | 34 | 1 | 1 | 0.33 |
| 6202 | 0 | US | F21K | F21K0099 | 3 | 21 | 0 | 1 | 0.00 |
| 6203 | 0 | JP | F21V | F21V0007 | 14 | 7 | 0 | 1 | 0.00 |
| 6204 | 0 | KR | H01L | H01L0021 | 7 | 27 | 0 | 1.25 | 0.00 |
| 6205 | 0 | US | C09K | C09K0011 | 30 | 118 | 0 | 1.833333333 | 0.00 |
| 6206 | 0 | JP | H01L | H01L0021 | 8 | 6 | 0 | 1 | 0.00 |
| 6207 | 0 | KR | H01L | H01L0021 | 8 | 29 | 0 | 1 | 0.00 |
| 6208 | 0 | US | H01L | H01L0027 | 18 | 11 | 0 | 1 | 0.00 |
| 6209 | 0 | KR | H01L | H01L0021 | 14 | 7 | 0 | 1.666666667 | 0.00 |
| 6210 | 0 | US | G02B | G02B0006 | 12 | 9 | 1 | 1.75 | 0.14 |
| 6211 | 0 | KR | H01L | H01L0029 | 6 | 9 | 0 | 1.25 | 0.00 |
| 6212 | 0 | KR | H01L | H01L0031 | 13 | 13 | 1 | 1.25 | 0.20 |
| 6213 | 0 | US | C09K | C09K0011 | 26 | 8 | 1 | 5 | 0.02 |
| 6214 | 0 | KR | H01L | H01L0033 | 11 | 2 | 0 | 1 | 0.00 |
| 6215 | 0 | JP | H01L | H01L0033 | 2 | 72 | 0 | 1.75 | 0.00 |
| 6216 | 0 | JP | H01L | H01L0033 | 19 | 21 | 1 | 1 | 0.50 |
| 6217 | 0 | US | F21V | F21V0019 | 22 | 9 | 0 | 1 | 0.00 |
| 6218 | 0 | US | F21V | F21V0033 | 24 | 7 | 1 | 1 | 0.17 |
| 6219 | 0 | JP | H01L | H01L0051 | 14 | 61 | 0 | 1 | 0.00 |
| 6220 | 0 | KR | H01L | H01L0021 | 25 | 36 | 1 | 7 | 0.14 |
| 6221 | 0 | US | H01L | H01L0021 | 26 | 76 | 1 | 1.2 | 0.17 |
| 6222 | 0 | DE | H01L | H01L0021 | 29 | 186 | 0 | 5.857142857 | 0.00 |
| 6223 | 0 | TW | H01L | H01L0031 | 7 | 1 | 0 | 2 | 0.00 |
| 6224 | 0 | JP | H01L | H01L0033 | 19 | 4 | 0 | 1.666666667 | 0.00 |
| 6225 | 0 | CN | H01L | H01L0027 | 6 | 3 | 0 | 1 | 0.00 |
| 6226 | 0 | US | H01L | H01L0033 | 20 | 72 | 1 | 1.222222222 | 0.09 |
| 6227 | 0 | US | H01L | H01L0021 | 13 | 8 | 0 | 1 | 0.00 |
| 6228 | 0 | JP | H01L | H01L0023 | 23 | 21 | 0 | 1 | 0.00 |
| 6229 | 0 | KR | H01J | H01J0001 | 18 | 8 | 0 | 1 | 0.00 |
| 6230 | 0 | JP | H01L | H01L0051 | 37 | 33 | 0 | 2 | 0.00 |
| 6231 | 0 | JP | F21V | F21V0029 | 20 | 23 | 3 | 1.4 | 0.43 |
| 6232 | 0 | US | H01L | H01L0051 | 2 | 21 | 0 | 1 | 0.00 |
| 6233 | 0 | TW | H05B | H05B0033 | 9 | 15 | 0 | 2 | 0.00 |
| 6234 | 0 | TW | C03B | C03B0029 | 12 | 4 | 0 | 1 | 0.00 |
| 6235 | 0 | JP | H01L | H01L0021 | 7 | 16 | 0 | 1 | 0.00 |
| 6236 | 0 | TW | H01L | H01L0021 | 17 | 7 | 0 | 1.333333333 | 0.00 |
| 6237 | 0 | DE | H01L | H01L0021 | 14 | 19 | 0 | 1.25 | 0.00 |
| 6238 | 0 | JP | H01L | H01L0033 | 11 | 3 | 0 | 1 | 0.00 |
| 6239 | 0 | KR | H01L | H01L0033 | 13 | 12 | 0 | 2.5 | 0.00 |
| 6240 | 0 | JP | H01L | H01L0033 | 12 | 18 | 0 | 1.666666667 | 0.00 |
| 6241 | 0 | KR | H01L | H01L0021 | 8 | 8 | 0 | 1 | 0.00 |
| 6242 | 0 | FR | H01L | H01L0029 | 17 | 10 | 0 | 1 | 0.00 |
| 6243 | 0 | JP | H01L | H01L0023 | 11 | 19 | 0 | 1.166666667 | 0.00 |
| 6244 | 0 | US | G08B | G08B0005 | 30 | 20 | 8 | 1 | 1.60 |
| 6245 | 0 | US | H01S | H01S0005 | 35 | 19 | 1 | 1 | 0.20 |
| 6246 | 0 | US | C09K | C09K0011 | 21 | 6 | 1 | 1 | 0.17 |
| 6247 | 0 | KY | H01L | H01L0021 | 23 | 7 | 0 | 1 | 0.00 |
| 6248 | 0 | HK | H01L | H01L0021 | 10 | 9 | 0 | 1.25 | 0.00 |
| 6249 | 0 | JP | H01L | H01L0021 | 8 | 16 | 0 | 1 | 0.00 |
| 6250 | 0 | US | H01L | H01L0051 | 13 | 5 | 0 | 1.333333333 | 0.00 |
| 6251 | 0 | US | H01L | H01L0027 | 9 | 31 | 0 | 1 | 0.00 |
| 6252 | 0 | JP | H01L | H01L0033 | 34 | 78 | 2 | 1.333333333 | 0.50 |
| 6253 | 0 | TW | H01L | H01L0033 | 8 | 6 | 0 | 1 | 0.00 |
| 6254 | 0 | JP | H01L | H01L0029 | 3 | 10 | 0 | 1 | 0.00 |
| 6255 | 0 | TW | H01J | H01J0061 | 11 | 2 | 0 | 1.5 | 0.00 |
| 6256 | 0 | JP | H01L | H01L0033 | 32 | 45 | 0 | 2 | 0.00 |
| 6257 | 0 | US | H01L | H01L0033 | 28 | 15 | 0 | 1 | 0.00 |
| 6258 | 0 | JP | F21V | F21V0007 | 6 | 6 | 0 | 1 | 0.00 |
| 6259 | 0 | TW | H01L | H01L0021 | 27 | 18 | 0 | 3 | 0.00 |
| 6260 | 0 | CN | H01L | H01L0021 | 6 | 4 | 0 | 1 | 0.00 |
| 6261 | 0 | TW | H01L | H01L0021 | 10 | 4 | 0 | 1.25 | 0.00 |
| 6262 | 0 | KR | H01L | H01L0021 | 16 | 8 | 0 | 1.333333333 | 0.00 |
| 6263 | 0 | JP | H01L | H01L0021 | 9 | 2 | 0 | 1 | 0.00 |
| 6264 | 0 | US | H01L | H01L0023 | 11 | 6 | 0 | 1 | 0.00 |
| 6265 | 0 | CN | H01L | H01L0029 | 9 | 3 | 0 | 2 | 0.00 |
| 6266 | 0 | KR | H01L | H01L0029 | 9 | 5 | 0 | 1 | 0.00 |
| 6267 | 0 | TW | H01L | H01L0023 | 16 | 10 | 1 | 1 | 1.00 |
| 6268 | 0 | JP | H01L | H01L0023 | 10 | 14 | 0 | 1.333333333 | 0.00 |
| 6269 | 0 | JP | C30B | C30B0035 | 20 | 11 | 0 | 1 | 0.00 |
| 6270 | 0 | JP | B32B | B32B0015 | 11 | 34 | 0 | 1 | 0.00 |
| 6271 | 0 | US | F21S | F21S0004 | 17 | 80 | 0 | 1.666666667 | 0.00 |
| 6272 | 0 | JP | C09K | C09K0011 | 12 | 13 | 1 | 1 | 0.50 |
| 6273 | 0 | US | H01L | H01L0021 | 14 | 22 | 1 | 1.2 | 0.17 |
| 6274 | 0 | US | H01L | H01L0033 | 32 | 32 | 0 | 1.5 | 0.00 |
| 6275 | 0 | TW | H01L | H01L0029 | 21 | 11 | 0 | 1 | 0.00 |
| 6276 | 0 | JP | H01L | H01L0033 | 5 | 3 | 0 | 1 | 0.00 |
| 6277 | 0 | DE | H01L | H01L0029 | 17 | 38 | 0 | 1 | 0.00 |
| 6278 | 0 | JP | H01L | H01L0033 | 7 | 4 | 1 | 1 | 0.50 |
| 6279 | 0 | KR | H01L | H01L0033 | 10 | 4 | 1 | 1 | 0.33 |
| 6280 | 0 | JP | H01L | H01L0027 | 19 | 11 | 2 | 1 | 1.00 |
| 6281 | 0 | KR | H01L | H01L0023 | 15 | 12 | 0 | 1 | 0.00 |
| 6282 | 0 | NL | F21V | F21V0029 | 9 | 29 | 1 | 1 | 0.14 |
| 6283 | 0 | TW | H01L | H01L0021 | 9 | 7 | 0 | 2 | 0.00 |
| 6284 | 0 | JP | H01L | H01L0021 | 13 | 22 | 0 | 1.5 | 0.00 |
| 6285 | 0 | DE | G02B | G02B0006 | 8 | 35 | 0 | 1.666666667 | 0.00 |
| 6286 | 0 | JP | H01L | H01L0021 | 9 | 4 | 0 | 1 | 0.00 |
| 6287 | 0 | TW | H01L | H01L0029 | 3 | 5 | 1 | 1 | 1.00 |
| 6288 | 0 | JP | H01L | H01L0033 | 4 | 10 | 0 | 1 | 0.00 |
| 6289 | 0 | KR | H01L | H01L0021 | 13 | 5 | 0 | 1.5 | 0.00 |
| 6290 | 0 | KR | H01L | H01L0033 | 11 | 2 | 0 | 2.333333333 | 0.00 |
| 6291 | 0 | JP | H01L | H01L0033 | 6 | 3 | 1 | 1 | 0.33 |
| 6292 | 0 | JP | H01L | H01L0033 | 7 | 3 | 0 | 1.5 | 0.00 |
| 6293 | 0 | US | H01L | H01L0027 | 37 | 32 | 0 | 1 | 0.00 |
| 6294 | 0 | JP | H01L | H01L0029 | 16 | 6 | 0 | 1 | 0.00 |
| 6295 | 0 | KR | H01L | H01L0033 | 11 | 6 | 1 | 1.25 | 0.20 |
| 6296 | 0 | KR | C30B | C30B0001 | 9 | 9 | 0 | 1 | 0.00 |
| 6297 | 0 | TW | B32B | B32B0037 | 6 | 6 | 0 | 1 | 0.00 |
| 6298 | 0 | US | H01L | H01L0033 | 25 | 15 | 1 | 1 | 0.50 |
| 6299 | 0 | DE | H01L | H01L0021 | 14 | 34 | 0 | 2.25 | 0.00 |
| 6300 | 0 | US | H01L | H01L0021 | 19 | 281 | 57 | 2 | 5.70 |
| 6301 | 0 | JP | H01L | H01L0027 | 11 | 10 | 0 | 1 | 0.00 |
| 6302 | 0 | US | F21V | F21V0007 | 26 | 175 | 0 | 1 | 0.00 |
| 6303 | 0 | US | H01L | H01L0029 | 20 | 46 | 1 | 1.2 | 0.17 |
| 6304 | 0 | US | H01L | H01L0029 | 35 | 60 | 1 | 1.666666667 | 0.10 |
| 6305 | 0 | TW | H01L | H01L0023 | 22 | 5 | 0 | 2 | 0.00 |
| 6306 | 0 | JP | H01L | H01L0023 | 7 | 9 | 0 | 1 | 0.00 |
| 6307 | 0 | TW | F21V | F21V0007 | 19 | 6 | 1 | 2 | 0.50 |
| 6308 | 0 | US | H01L | H01L0029 | 8 | 60 | 3 | 1.666666667 | 0.30 |
| 6309 | 0 | JP | C09J | C09J0163 | 6 | 5 | 0 | 1 | 0.00 |
| 6310 | 0 | US | H01S | H01S0003 | 24 | 31 | 0 | 1 | 0.00 |
| 6311 | 0 | KR | H01L | H01L0021 | 20 | 13 | 0 | 1 | 0.00 |
| 6312 | 0 | US | H01L | H01L0021 | 12 | 8 | 0 | 1 | 0.00 |
| 6313 | 0 | KR | H01L | H01L0021 | 15 | 10 | 0 | 1 | 0.00 |
| 6314 | 0 | GB | C07C | C07C0013 | 8 | 25 | 0 | 1.333333333 | 0.00 |
| 6315 | 0 | TW | H01L | H01L0033 | 33 | 7 | 0 | 2 | 0.00 |
| 6316 | 0 | US | H01L | H01L0021 | 20 | 19 | 1 | 1 | 0.33 |
| 6317 | 0 | JP | H01L | H01L0051 | 11 | 7 | 1 | 1.2 | 0.17 |
| 6318 | 0 | JP | H01L | H01L0033 | 15 | 12 | 0 | 1 | 0.00 |
| 6319 | 0 | JP | H01L | H01L0033 | 7 | 5 | 0 | 1 | 0.00 |
| 6320 | 0 | KR | H01L | H01L0029 | 16 | 26 | 1 | 2.166666667 | 0.08 |
| 6321 | 0 | US | H01L | H01L0031 | 16 | 23 | 1 | 1.333333333 | 0.25 |
| 6322 | 0 | US | H01L | H01L0029 | 14 | 10 | 0 | 1.2 | 0.00 |
| 6323 | 0 | KR | H01L | H01L0051 | 11 | 17 | 0 | 1.5 | 0.00 |
| 6324 | 0 | JP | H01L | H01L0023 | 20 | 5 | 0 | 1 | 0.00 |
| 6325 | 0 | US | H01L | H01L0021 | 17 | 13 | 0 | 1 | 0.00 |
| 6326 | 0 | US | H01L | H01L0021 | 12 | 2 | 0 | 1.5 | 0.00 |
| 6327 | 0 | US | G06Q | G06Q0040 | 57 | 32 | 0 | 2 | 0.00 |
| 6328 | 0 | DE | H01L | H01L0027 | 17 | 33 | 0 | 1 | 0.00 |
| 6329 | 0 | US | C30B | C30B0029 | 47 | 45 | 3 | 1 | 0.33 |
| 6330 | 0 | KR | H01L | H01L0021 | 14 | 10 | 0 | 1.25 | 0.00 |
| 6331 | 0 | US | H01L | H01L0033 | 26 | 37 | 0 | 2 | 0.00 |
| 6332 | 0 | JP | H01L | H01L0021 | 14 | 60 | 0 | 1 | 0.00 |
| 6333 | 0 | JP | H01L | H01L0021 | 27 | 4 | 0 | 1.5 | 0.00 |
| 6334 | 0 | SG | H01L | H01L0021 | 19 | 5 | 0 | 2 | 0.00 |
| 6335 | 0 | JP | H01L | H01L0033 | 8 | 10 | 0 | 1 | 0.00 |
| 6336 | 0 | JP | H01L | H01L0051 | 4 | 9 | 0 | 1 | 0.00 |
| 6337 | 0 | JP | H01L | H01L0029 | 9 | 11 | 0 | 1 | 0.00 |
| 6338 | 0 | JP | H01L | H01L0031 | 29 | 25 | 0 | 1.142857143 | 0.00 |
| 6339 | 0 | JP | H01L | H01L0023 | 18 | 12 | 0 | 1 | 0.00 |
| 6340 | 0 | JP | H01L | H01L0023 | 20 | 50 | 1 | 1.25 | 0.20 |
| 6341 | 0 | JP | C01F | C01F0017 | 6 | 23 | 0 | 1.25 | 0.00 |
| 6342 | 0 | KR | H01J | H01J0001 | 10 | 24 | 2 | 1 | 0.50 |
| 6343 | 0 | JP | G01J | G01J0003 | 16 | 1 | 0 | 1 | 0.00 |
| 6344 | 0 | US | G02B | G02B0006 | 8 | 10 | 0 | 1 | 0.00 |
| 6345 | 0 | US | H01J | H01J0009 | 20 | 11 | 0 | 1 | 0.00 |
| 6346 | 0 | US | H01L | H01L0021 | 13 | 9 | 0 | 1.5 | 0.00 |
[truncated: 51,038 more chars]
